# Supplementary material for: Phase 1 double-blind randomized safety trial of the Janus kinase inhibitor tofacitinib in systemic lupus erythematosus
Source: Nat Commun. 2021 Jun 7;12:3391. doi: 10.1038/s41467-021-23361-z (PMC8185103; doi:10.1038/s41467-021-23361-z)
Supplement: Supplementary file 1 — Supplementary Information [file 41467_2021_23361_MOESM1_ESM.pdf]

Supplemental Material

This supplement contains the following items:

1. Supplementary methods
2. Supplementary tables
3. Supplementary figures
4. Supplementary references
5. Original protocol, final protocol, summary of changes.
6. Original statistical analysis plan. No changes were made to the original statistical plan.

Supplementary Methods:

STAT phosphorylation (pSTAT) in immune cell subsets: Peripheral blood mononuclear cells (PBMCs) were isolated by Ficoll-Paque gradient centrifugation (GE Healthcare, Chicago, IL) with Leucosep centrifuge tubes (VWR, Radnor, PA) according to manufacturer's instructions. Cells were frozen in medium containing 90% FCS and 10% dymethyl-sulfoxide (DMSO, Sigma-Aldrich, St. Louis, MO, USA) and stored in liquid nitrogen until use. Cryopreserved PBMCs were thawed in a water bath at 37°C, using media containing the Anti-Aggregate (CTL) wash buffer (Cellular Technology Limited, Shaker Heights, OH), and viability assessed using acridine orange/propidium iodide. PBMCs (1.5 x 10^6) were left untreated or stimulated for 20 min at 37°C with recombinant human IFN-α (Cell Signaling Technology; Danvers, MA) at 200 ng/mL for pSTAT1 or recombinant human IL-2 (Hoffmann-La Roche Inc; Nutley, NJ) at 200 U/mL for pSTAT5, or recombinant IL-10 (PeproTech; Rocky Hill, NJ) at 200 ng/mL for pSTAT3(data not shown). Untreated PBMCs were processed following the same protocol and used as negative control. The pSTAT1, pSTAT3and pSTAT5 expression levels were measured on $CD3^+$, $CD4^+$, $CD8^+$, $CD20^+$, and $CD14^+$ cells. Sample acquisition was carried out by high-throughput multiplexed fluorescent cell barcoding on a LSR Fortessa cytometer (BD Biosciences) equipped with 6 lasers (355 nm, 405 nm, 488 nm, 552 nm, 592 nm, and 628 nm wavelengths) and BD FACSDiva software (v.8.0.1, BD Biosciences). Data were analyzed using Prism (v.8.0.1; GraphPad software, Inc., La Jolla, CA). Fluorescence values were reported as median fluorescence intensity (MFI), Coefficient of variance (CV=SD/mean of population), and MFI fold change of unstimulated cell population vs stimulated cell population.

Quantification of LDGs: PBMCs were isolated by Ficoll-Paque density gradient and red blood cells were lysed with hypertonic solution. PBMCs were resuspended in 2% FBS/PBS, blocked for

15 minutes with Human TruStain Fc Receptor Blocking Solution (BioLegend; San Diego, CA), then resuspended in FACS buffer and incubated with fluorochrome-conjugated mouse anti–human CD10 (clone H10A, catalog 312209), –CD15 (clone HI98, catalog 301906), and –CD14 (clone HCD14, catalog 325610) antibodies (BioLegend; San Diego, CA) or isotype control for 15 minutes in the dark. Cells were fixed with 2% PFA. Data was collected using a BD FACSCanto RUO and analyzed using FlowJo Software Version 10. Cutoff values for positive staining were determined using compensation controls for each fluorophore.

RNA isolation and RNA sequencing analysis: Peripheral blood was collected by venipuncture in PAXgene Blood RNA tubes (BD Diagnostics; Franklin Lakes, NJ) and stored at –80°C until use. RNA was isolated using PAXgene Blood RNA Kit (Qiagen; Germantown, MD) following manufacturer's instructions. cDNA libraries were prepared using the TruSeq Stranded mRNA NeoPrep Kit (Illumina; San Diego, CA). RNA-Seq data were generated with Illumina's HiSeq 2500 or 3000 system. Raw sequencing data were processed with CASAVA 1.8.2 to generate FastQ files. Reads of 50 bases were mapped to the human transcriptome and genome hg19 using TopHat 2.1.1. Reads mapping to hemoglobin genes (*HBA1*, *HBA2*, *HBB*, *HBD*) were removed from the TopHat–generated BAM files using BEDTools and customized Bash scripts. Hemoglobin-removed BAM files were used for downstream analysis including reads per kilobase exon per million mapped reads (RPKM) calculations.

Type I IFN NanoString: Total RNA was extracted from whole blood using Paxgene blood RNA isolation kit (PreAnalytiX; Homberchtikon, Switzerland) that included DNAse treatment. RNA concentration was measured by NanoDrop (Thermo Fisher Scientific; Waltham, MA). The nCounter Element prep kit (NanoString Technologies; Seattle, WA) was used for Nanostring assay. A custom NanoString Elements CodeSet consisting of fluorescently labeled specific

Reporter Tags and a biotinylated universal Capture Tag were supplied by NanoString. Target-specific oligonucleotide probe pairs (synthesized by IDT, Coralville, IA) contained 37 IFN stimulated genes (ISGs), previously identified as discriminative of the IFN signature, and 4 housekeeping genes (*ALAS1*, *HPRT1*, *TBP*, *TUBB*). A total of 100 ng of RNA were used for hybridization at 67° C for 16-21 h on thermocycler. The hybridized samples were inserted into the nCounter Prep Station, where they were purified and immobilized onto the internal surface of a sample cartridge for 2-3 h. Finally, the sample cartridge was transferred to the nCounter Digital Analyzer where color codes were counted and tabulated for each target molecule. The resulting data were processed with nSolver software (NanoString Technologies; Seattle, WA), which included assessment of quality of the runs.

Neutrophil extracellular trap (NET) complex ELISA: In brief, 96-well ELISA plates were coated overnight at 4°C with rabbit anti–human HNE (Calbiochem; San Diego, CA). Plates were blocked in 1% BSA and incubated overnight with plasma in blocking buffer. After washing, plates were incubated for 1 hour at room temperature with anti-dsDNA (clone BV16-13, MilliporeSigma; Burlington, MA). Plates were washed and incubated for 1 hour with anti–mouse IgG-HRP conjugate (Bio-Rad), followed by a wash and the addition of TMB substrate (MilliporeSigma; Burlington, MA) and stop reagent (MilliporeSigma; Burlington, MA). Absorbance was measured at 450 nm, and values were calculated as an OD Index.

Immunofluorescence analysis of cell subsets by flow cytometry: Flow cytometry was performed as previously described (1, 2). Briefly, PBMCs were isolated by Ficoll separation and cryopreserved at -120ºC according to NIH Center for Human Immunology (CHI) protocols (https://chi.niaid.nih.gov/web/new/our-research/SOP-Isolation.pdf). Thawed cells were washed and resuspended in PBS, viability assessed using LIVE/DEAD Aqua fixable viability dye (Life

Sciences, Carlsbad, CA), followed by a wash in FACS staining buffer (PBS supplemented with 1% normal mouse serum, 1% goat serum, 0.02% sodium azide) (Gemini Bioproducts, West Sacramento, CA). Cells were stained with three 15-color panels from the previously described comprehensive leukocyte immunophenotyping panel shown in Supplemental Table 3 (1, 2). Acquisition was performed using a Becton Dickinson LSR Fortessa (BD, San Jose, CA) equipped with five lasers (355 nm, 407 nm, 488 nm, 532 nm, 633 nm wavelengths) with 22 PMT detectors. Data were acquired using DIVA 6.1.2 software (BD) and we ensured a minimum of 50,000 $CD4^+$ T cells was recorded to be able to accurately assess minor cell populations.

Serum cytokines: Luminex assays were performed as previously described (2). Briefly, serum was collected and stored at −120°C per NIH CHI protocols (https://chi.niaid.nih.gov/web/new/our-research/SOP-Serum-SSTTubes.pdf). Cytokines were quantified using ProcartaPlex 60-plex kits from eBioscience, with analytes tested listed in Supplemental Table 4. Median fluorescence intensities were collected on a Luminex-100 instrument (Luminex, Bio-Rad), using Bio-Plex Manager software version 6. Standard curves were generated for each cytokine using lyophilized standards. Cytokine concentrations were determined from standard curves using a five point 5-parameter logistic regression. Samples were run in duplicate and averages used for analysis.

High density lipoprotein (HDL) cholesterol efflux capacity: Briefly, 3 x 10^5 J774 cells/well were seeded in 24-well plate and radiolabeled with 2 μCi of ^{3}H-cholesterol/mL in RPMI-1640 media containing 1% FBS for 24-hours. Cells were incubated for 16-hours in RPMI media containing 2% BSA in the presence or absence of 0.3 mmol/L 8-(4-chlorophenylthio)-cAMP to upregulate ATP-binding cassette transporter A1 (ABCA1). This was followed by addition of 2.8% apoB-depleted plasma to the efflux medium for 4 hours. A liquid scintillation counter was used to quantify the efflux of radioactive cholesterol from cells using the formula: (μCi of ^{3}H-cholesterol

in media containing 2.8% apoB-depleted subject plasma-μCi of ^{3}H-cholesterol in plasma-free media / μCi of ^{3}H-cholesterol in media containing 2.8% apoB-depleted pooled control plasma-μCi of ^{3}H-cholesterol in pooled control plasma-free media). Pooled plasma was obtained from five healthy adult volunteers. All assays were performed in duplicate.

Vascular function assessment: Subjects were asked to fast for at least 6 hours prior to these tests and to refrain from smoking or drinking caffeinated beverages for 24 hours prior to the studies. Subjects were asked to hold vasodilators, anti-hypertensives and statins on the morning of the test. During testing, subjects were placed in a temperature-controlled quiet room in the supine position.

1)CAVI**.** CAVI was measured using VaSera-1500A (Fukuda Denshi Co. Redmond, WA). After placing blood pressure (BP) cuffs around both arms and ankles and attaching electrocardiogram (EKG) electrodes to the upper arms, a microphone was placed on the sternal angle to record heart sounds. Measurements were automatically calculated using the VaSera VS-1000 software. The principle underlying CAVI has been discussed previously (3).

2) PAT. Microvascular endothelial function was evaluated using PAT with an EndoPAT 2000 device (Itamar Medical Ltd. Caesarea, Israel) as previously described (4). Finger probes were placed on symmetric fingers bilaterally, and a BP cuff was placed on one arm, with the other arm serving as control. PAT was continuously measured for 20 minutes. In between, for 5 minutes, BP cuff was inflated to supra systolic pressure in the test arm. At the end of the occlusion and dilatation periods, reactive hyperemia was captured as an increase in the PAT signal amplitude and compared with the control arm. A postocclusion to preocclusion ratio was calculated by EndoPAT software, providing a reactive hyperemia index (RHI). Augmentation index (AI) was calculated from PAT pulses at the baseline period. The result was further normalized to heart rate of 75 bpm (AI@75), as previously described (4).

3)SphygmoCor pulse wave analysis and velocity system: Central aortic BP and stiffness were quantified using SphygmoCor CP system (AtCor Medical Pty Ltd.; New South Wales, Australia). The central aortic pressure PWV was determined by using the pressure tonometer and an EKG signal was used simultaneously to visualize ventricular-vascular interactions. Standard algorithm and procedures, as described elsewhere, were used to quantify results (5).

Supplemental Table 1. Longitudinal follow-up of laboratory values during the trial stratified according the *STAT4* risk allele status.

	**Tofacitinib (n=20)**						**Placebo (n=10)**					
	**STAT4 Positive(n=10)**			**STAT4 Negative(n=10)**			**STAT4 Positive(n=5)**			**STAT4 Negative (n=5)**		
**Outcome variable**	**Day 1**	**Day 56**	**Day 84**	**Day 1**	**Day 56**	**Day 84**	**Day 1**	**Day 56**	**Day 84**	**Day 1**	**Day 56**	**Day 84**
	**mean ± SD**	**mean ± SD**	**mean ± SD**	**mean ± SD**	**mean ± SD**	**mean ± SD**	**mean ± SD**	**mean ± SD**	**mean ± SD**	**mean ± SD**	**mean ± SD**	**mean ± SD**
**WBC, K/mcL**	6.2 ± 1.7	5.9 ± 1.9	5.4 ± 1.8	4.5 ± 0.8	4.5 ± 1.1	4.1 ± 0.8	4.9 ± 0.8	5.8 ± 1.6	5 ± 1.3	4.6 ± 1.4	4.8 ± 1.6	4.8 ± 1
**Absolute Neutrophil Counts, K/uL**	3.8 ± 1.2	3.4 ± 1.4	3.2 ± 1.3	2.3 ± 0.7	2.3 ± 1.1	2.2 ± 0.6	2.9 ± 0.9	3.8 ± 1.3	3 ± 0.8	2.5 ± 0.9	2.6 ± 1.1	2.5 ± 0.7
**Hemoglobin, g/dL**	13.1 ± 1.2	13 ± 1.3	13 ± 1.2	12.5 ± 1.3	12.1 ± 1.1	11.8 ± 1.3	12.8 ± 1.4	12.6 ± 1.4	12.2 ± 1.5	13.1 ± 1.8	13.4 ± 2.4	13.3 ± 2.3
**Platelet, K/mcL**	223.4 ± 67.8	218.9 ± 80.5	227.8 ± 64.8	224.7 ± 81.5	224.2 ± 73.1	235.1 ± 75.2	201.2 ± 73.7	220 ± 77.4	199.6 ± 57.8	225.6 ± 41.3	231.8 ± 63.6	227.8 ± 52.7
**ESR, mm/hr**	22.4 ± 22.2	15.6 ± 17.6	21.2 ± 19.8	21.5 ± 17	23.1 ± 16.9	24 ± 16.1	32.4 ± 23.5	33 ± 26.3	34.6 ± 27.5	19.4 ± 25.8	18.4 ± 26.8	16.8 ± 22.8
**CRP, mg/L**	6.9 ± 10.3	1.2 ± 1	3.4 ± 3.7	2.4 ± 2.5	3.3 ± 4.3	3 ± 3.2	0.9 ± 0.5	3.2 ± 4.9	1.1 ± 0.8	4.4 ± 5.2	3.4 ± 5.9	6.8 ± 11.4
**C3,mg/dL**	100.5 ± 24	96.2 ± 29.2	101.5 ± 21.8	108.7 ± 22.3	107.7 ± 19.1	109.7 ± 21	98.7 ± 12.2	98.2 ± 21.1	110.6 ± 41.2	111.5 ± 32.8	117.9 ± 41.1	120.5 ± 35.3
**C4, mg/dL**	18 ± 6.4	15.9 ± 7.6	17.7 ± 6.8	24.9 ± 12.6	24.5 ± 11.5	23.5 ± 11.1	16.9 ± 6.5	16.7 ± 7.3	20.1 ± 11.4	20.3 ± 8.5	21.1 ± 9	21.5 ± 8.5
**Anti-ds-DNA, IU/mL***	149 ± .	160 ± .	188 ± .	86 ± 88.5	119.3 ± 96.9	149.5 ± 131.9	42.5 ± 2.1	50 ± 12.7	102.5 ± 99.7	80 ± .	84 ± .	94 ± .
**AST, u/L**	23.4 ± 5.4	23.7 ± 7.4	23.9 ± 5.8	17.7 ± 3.6	21 ± 4.2	19 ± 4.3	22.4 ± 1.7	26.2 ± 11.9	50.6 ± 64	21 ± 6.4	18.2 ± 2.8	19.6 ± 2.9
**ALT, u/L**	22.9 ± 9.7	24.8 ± 14.1	25.3 ± 11.9	12.4 ± 4.1	15.3 ± 5.5	13.8 ± 4.8	23.6 ± 10.9	26.4 ± 18.8	30 ± 15.4	15.8 ± 3.1	14.2 ± 3.3	16.2 ± 4.8
**Cholesterol, mg/dL**	158.7 ± 33.5	175.1 ± 53.3	161.8 ± 38.6	182.2 ± 25.4	194.6 ± 35.9	179.4 ± 27.2	137.2 ± 23	132.2 ± 18.8	131 ± 25.6	159.6 ± 19.1	162.2 ± 24.8	168.2 ± 27
**Triglycerides, mg/dL**	111.3 ± 52.9	142.8 ± 147.5	119.4 ± 88.6	106.1 ± 42	88.8 ± 33.5	94.6 ± 30.2	94.6 ± 36.4	79.2 ± 30.1	83.6 ± 25.3	65 ± 11.9	89.8 ± 57	97.8 ± 60.2
**HDL, mg/dL**	51.8 ± 15.5	56.8 ± 14	51.9 ± 18.5	61.1 ± 18.9	71.5 ± 20.8	61 ± 20.8	44.6 ± 11.3	44.6 ± 11.4	43 ± 12.3	59.8 ± 18.2	58.2 ± 17.5	60 ± 22.5
**LDL, mg/dL**	84.4 ± 33.7	81.8 ± 26.6	86.1 ± 31.5	100 ± 25	105.4 ± 32.8	99.5 ± 23.3	73.6 ± 17.6	72 ± 12.7	71.4 ± 14.9	86.8 ± 28.5	86 ± 33.8	89 ± 30.1
**HDL Particle Number, mcmol/L**	31.6 ± 7.4	33.9 ± 7.1	30.5 ± 7.2	32.6 ± 6	35.5 ± 5.2	31.9 ± 5.7	26.4 ± 4.8	26.2 ± 4.8	25.5 ± 5.2	31.4 ± 5.6	30.7 ± 5.9	30.7 ± 8
**HDL Size, nm**	9.5 ± 0.5	9.5 ± 0.5	9.5 ± 0.5	9.4 ± 0.5	9.6 ± 0.6	9.5 ± 0.5	9.4 ± 0.3	9.5 ± 0.3	9.5 ± 0.4	9.4 ± 0.8	9.5 ± 0.8	9.6 ± 0.6
**LDL Particle Number, nmol/L**	980.5 ± 463.8	1074.2 ± 692.7	1045.4 ± 545.2	1069 ± 336.2	1036.8 ± 416.2	1061.1 ± 313.7	818.6 ± 70.5	747.4 ± 197.6	787.4 ± 140.6	978.2 ± 405.2	990.6 ± 373.9	1074.8 ± 491.4
**LDL Size, nm**	20.5 ± 0.5	20.7 ± 0.6	20.4 ± 0.7	20.9 ± 0.4	21.1 ± 0.7	20.9 ± 0.7	20.5 ± 0.7	20.7 ± 0.6	20.5 ± 0.7	21 ± 0.4	20.7 ± 0.4	20.5 ± 0.7
**VLDL Particle Number, nmol/L**	47.7 ± 21.7	57.9 ± 54	55 ± 53.9	40.1 ± 18.5	43 ± 18.9	33.2 ± 11.4	42 ± 21.3	29.2 ± 11.6	34.4 ± 6.2	20.9 ± 4.5	40.1 ± 36.9	44 ± 36.8

**VLDL Size, nm**	49.6 ± 4.9	50.1 ± 6.2	49.2 ± 4.5	48.3 ± 4.9	45.4 ± 4.1	49 ± 10.1	46 ± 8.8	46.3 ± 3.8	44.8 ± 5	46 ± 6.1	48.2 ± 5.4	46.4 ± 4.9
**Glucose, mmol/l**	4.9 ± 0.7	4.9 ± 0.7	5.1 ± 0.8	5.1 ± 0.7	4.9 ± 0.8	5.3 ± 1.2	4.8 ± 0.4	5.1 ± 0.7	4.9 ± 0.6	4.9 ± 0.5	4.8 ± 0.6	5.1 ± 1.6
**Insulin, Pmol/L**	98.4 ± 37.7	97.2 ± 37	97.3 ± 41.7	98.9 ± 47.9	79.5 ± 34.3	95.6 ± 37.7	171.7 ± 72.4	167.1 ± 132.6	177 ± 84.6	114.6 ± 114.3	95.4 ± 61	231.6 ± 356.9

WBC, K/mcL= White blood cell, thousand cells per cubic microliter
ANC, K/uL= Absolute neutrophil count, thousand cells per microliter
ESR, mm/hr = Erythrocyte sedimentation rate, millimeters per hour
CRP, mg/L = C-reactive protein, milligram per liter
C3, mg/dl=Complement component 3, milligram per deciliter
C4, mg/dl= Complement component 4, milligram per deciliter
Anti-ds-DNA, IU/mL= Anti double-stranded DNA antibody, International units per milliliter
AST, u/L = Aspartate aminotransferase, units per liter
ALT, u/L = Alanine aminotransferase units per liter
HDL, mg/dL = High density lipoprotein, milligram per deciliter
LDL, mg/dL = Low density lipoprotein, milligram per deciliter
VLDL = Very low-density lipoprotein
mcmol/L = Micromoles per liter
nm = nanometer
Pmol/L = Picomole per liter
HOMA2 IR = Homeostatic Model Assessment Index for Insulin Resistance

*standard deviation were not calculated due to small numbers in each group after removing subjects with normal values

Supplemental Table 2: Disease activity and patient reported outcome measures stratified according to the *STAT4* risk allele status.

	Tofacitinib (n=20)						Placebo (n=10)					
	STAT4 positive (n=10)			***S**TAT4* negative (n=10)			*STAT4* positive (n=5)			*STAT4* negative (n=5)		
outcome variable	Day 1	Day 56	Day 84	Day 1	Day 56	Day 84	Day 1	Day 56	Day 84	Day 1	Day 56	Day 84
	mean±SD	mean ± SD	mean±SD	mean ± SD	mean ± SD	mean ± SD	mean ± SD	mean ± SD	mean ± SD	mean ± SD	mean ± SD	mean ± SD
SF36 TOTAL	111 ± 9.5	114.2 ± 10.2	113 ± 7.6	109.6 ± 10.1	112.3 ± 9.2	110.9 ± 11.1	107.4 ± 7.8	115 ± 6.7	113.2 ± 7.2	107.8 ± 9.8	108 ± 8.5	102.8 ± 9.9
FAT Average	1.1 ± 1.3	1.4 ± 1.8	1.4 ± 1.7	2.1 ± 2	2.4 ± 2.6	2.7 ± 2.6	4 ± 2.8	1.5 ± 1.4	1.6 ± 1.7	4.8 ± 2.8	2.8 ± 1.6	2.9 ± 2.7
SLEDAI-2K	5.2 ± 2.7	4.2 ± 2	3.9 ± 1.5	4.9 ± 1.8	4.2 ± 2.6	3.9 ± 2.3	5.6 ± 4.3	4.8 ± 3	5.4 ± 4	5.4 ± 3.4	4.2 ± 1.8	4.4 ± 1.7
PGA	0.9 ± 0.8	0.7 ± 0.8	0.8 ± 0.7	0.6 ± 0.9	0.9 ± 1	1 ± 0.7	1.2 ± 0.9	0.8 ± 0.8	0.6 ± 0.6	1.1 ± 0.9	1 ± 0.9	1.1 ± 1
DAS28-ESR	2.6 ± 1.5	2.4 ± 1.4	2.3 ± 0.8	2.7 ± 1.2	2.5 ± 1	2.8 ± 1	3.6 ± 2.1	3.2 ± 1.6	3.1 ± 1.3	2.7 ± 1.9	2.6 ± 1.6	2.5 ± 1.5
CLASI Total ACT	3.1 ± 2.1	2.1 ± 2.2	2.5 ± 2.3	2 ± 1.5	1.7 ± 1.1	1.6 ± 1.1	2 ± 1.2	2 ± 1.2	2 ± 1	2.8 ± 2.2	2.2 ± 2.2	2.4 ± 2.1
CLASI Total Damage	0.6 ± 1.3	0.6 ± 1.3	0.6 ± 1.3	1.4 ± 2.7	1.5 ± 2.9	1.5 ± 2.9	0.2 ± 0.4	0.2 ± 0.4	0 ± 0	1.8 ± 2.9	1.6 ± 3	1.8 ± 2.9
BILAG 2004	9.6 ± 2.7	8.2 ± 5.2	7 ± 3.5	5.5 ± 5.3	4.2 ± 3.6	3.9 ± 3.8	11.4 ± 3.6	11.6 ± 4.5	10.2 ± 3.8	7.2 ± 4.1	4.6 ± 4.2	5.6 ± 3.9

SF 36= Short Form Health Survey
MD-Fatigue = Multidimensional Assessment of Fatigue questionnaire
SLEDAI 2K = Systemic Lupus Erythematosus Disease Activity Index 2000
PGA = Physician Global Assessment
DAS 28-ESR = Disease Activity Score of the 28 joints with erythrocyte sedimentation rate
CLASI = Cutaneous Lupus Erythematosus Disease Area and Severity Index
BILAG 2004 = British Isles Lupus Assessment Group disease activity index

Supplemental Table 3

Flow cytometry panels used to phenotype peripheral blood immune cell subsets.

			T lineage		NK
Excitation	Fluorochrome name	PMT name	T_1 Treg	T_4 TFh	NK_2
407 Excitation	V450 Pac blue	V450	CD4 *RPA-T4*	CD4 *RPA-T4*	CD56 *B159*
	Aquablue	V545	Viability	Viability	Viability
	BV605	V605	CD8 *3B5*	CD8 *3B5*	CD8 *3B5*
	BV650	V655	CD27 *CLB-27/1*	CD27 *CLB-27/1*	NKp46 *900*
	BV785	V800	CD45 *HI30*	CD45 *HI30*	CD45 *HI30*
488 Excitation	FITC	B515	CD39 *A1*	CD183 *1C6/CXCR3*	CD57 *TB01*
	PcPcy5.5	B710	CD38 *HIT2*	CD196 *11A7*	IFNg *4S.B3*
532 Excitation	PE	G560	Foxp3 *PCH101*	CD278 *ISA-3*	Perforin *6G9*
	PE-TR	G610	CD45-RA *2H4LDH11LDB9*	CD45-RA *2H4LDH11LDB9*	CD25 *B1.49.9*
	PEcy5	G660	CD103 *LF61*	CD40L *TRAP1*	CD16 *3G8*
	PEcy5.5 /PE-A700	G710	HLA-DR *Tu36*	CD25 *3G10*	CD69 *CH/4*
	PEcy7	G780	CD25 *M-A251*	CD279 *EH12.1*	CD5 *L17F12*
633 Excitation	APC/eFluor660	R660	CD127 *hIL-7R-M21*	CD185 *RF8B2*	CD127 *hIL-7R-M21*
	Alexa700/APC Cy5.5	R710	CD197 *150503*	CD197 *150503*	CD158e1 *DX9*
	APC cy7	R780	CD3 *Sk7*	CD3 *Sk7*	CD3 *Sk7*

Supplemental Table 4.

Analytes measured in Luminex assay.

BDNF (57)	IL-5 (21)
b-NGF (55)	IL-6 (25)
EGF (56)	IL-7 (26)
Eotaxin (33)	IL-8 (27)
FGF-2 (75)	IL-9 (52)
G-CSF (59)	IP-10 (22)
GM-CSF (44)	Leptin (79)
GROa (61)	LIF (15)
HGH (46)	MCP-1 (51)
IFNa (48)	MCP-3 (68)
IFNb (30)	M-CSF (67)
IFNg (43)	MIG (69)
IL-1a (62)	MIP-1a (12)
IL-1b (18)	MIP-1b (47)
IL-1Ra (38)	PAI-1 (80)
IL-10 (28)	PDGF-bb (77)
IL-12p40 (64)	RANTES (42)
IL-12p70 (34)	Resistin (70)
IL-13 (35)	SCF (39)
IL-15 (65)	SDF-1a (13)
IL-17A (36)	sFAS-L (08)
IL-17F (07)	sICAM-1 (73)
IL-18 (66)	TGFa (09)
IL-2 (19)	TGFb (49)
IL-21 (72)	TNFa (45)
IL-22 (76)	TNFb (54)
IL-23 (63)	TRAIL (58)
IL-27 (14)	VCAM-1 (74)
IL-31 (37)	VEGF-A (78)
IL-4 (20)	VEGF-D (53)

Supplemental Figure 1

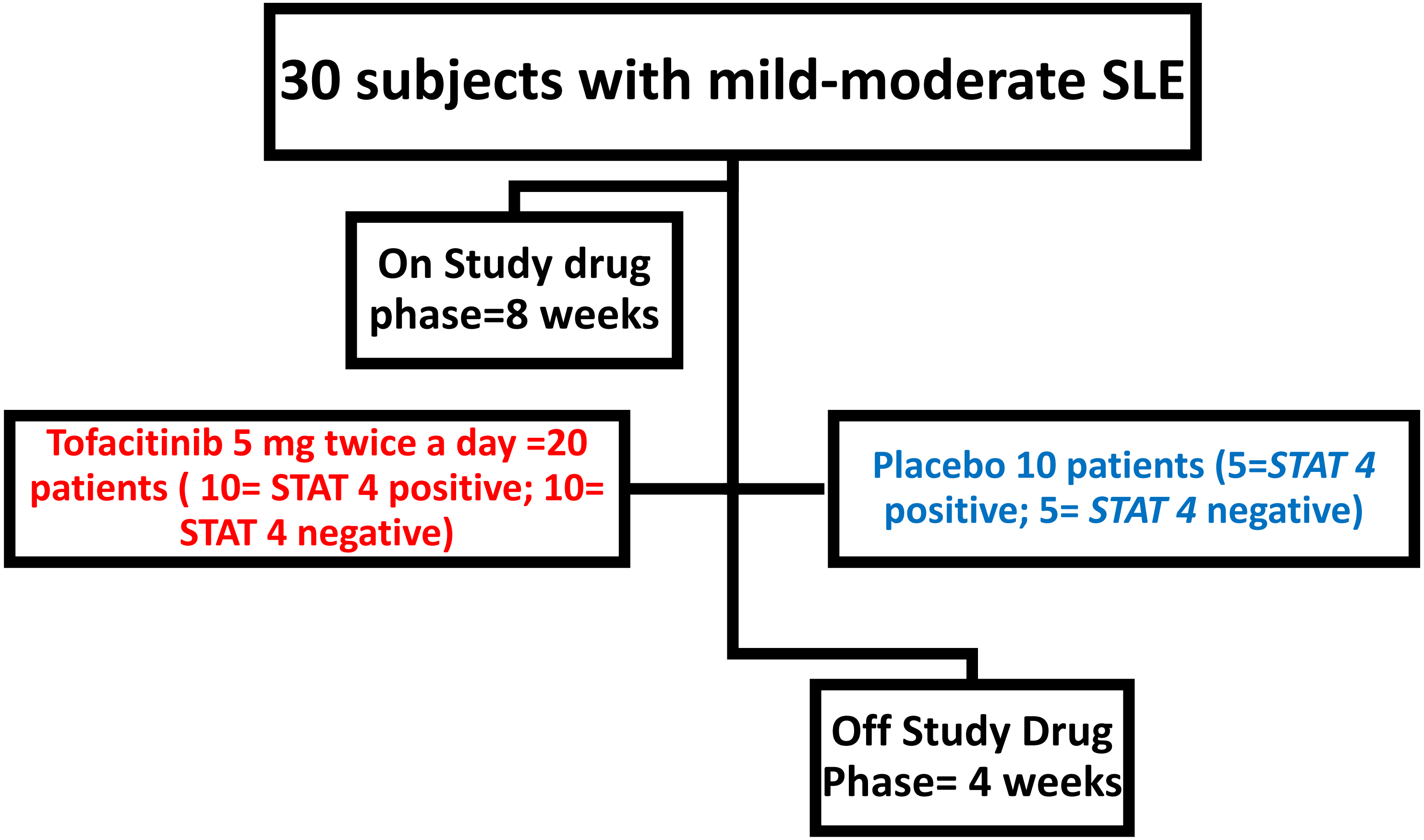

Supplemental Figure 1. Study design. After determining eligibility, 30 subjects were randomized in 2:1 ratio to receive either tofacitinib or placebo for 8 weeks. Half of the patients in each group were *STAT4*-risk allele positive. Subjects were followed for another 4 weeks off study medication.

Supplemental Figure 2

a

Tofacitinib

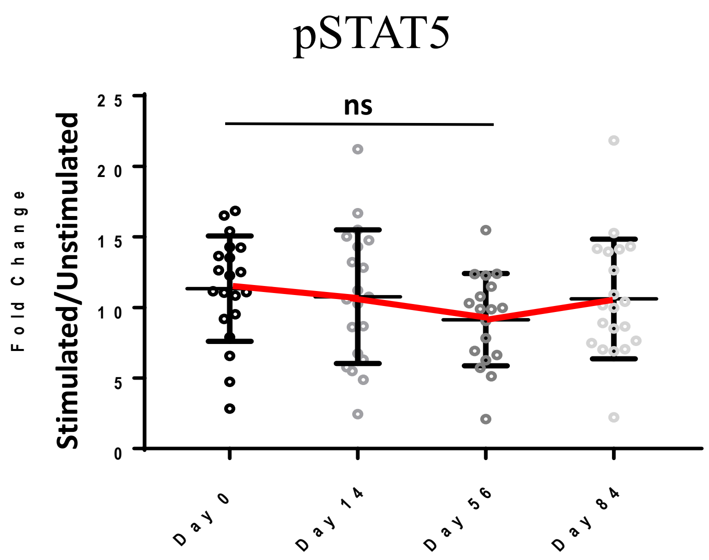

b

Placebo

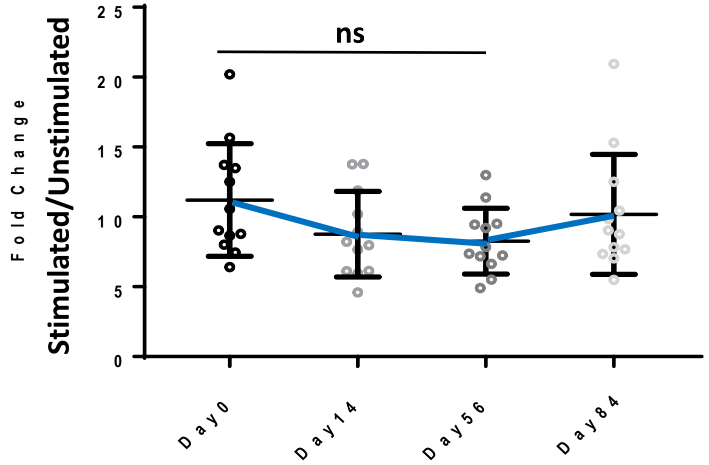

Supplemental Figure 2. Phosphorylation of STAT5 in $CD4^{+}$ T cells. The inhibition of pSTAT5 was not significant in subjects on a) tofacitinib n=20 biologically independent samples or b) placebo n=10 biologically independent samples. All data are presented as mean values +/- SEM.

A mixed linear model for repeated measures was used. Two tailed tests were used where appropriate.

No adjustments were made for multiple comparisons.

Supplemental Figure 3

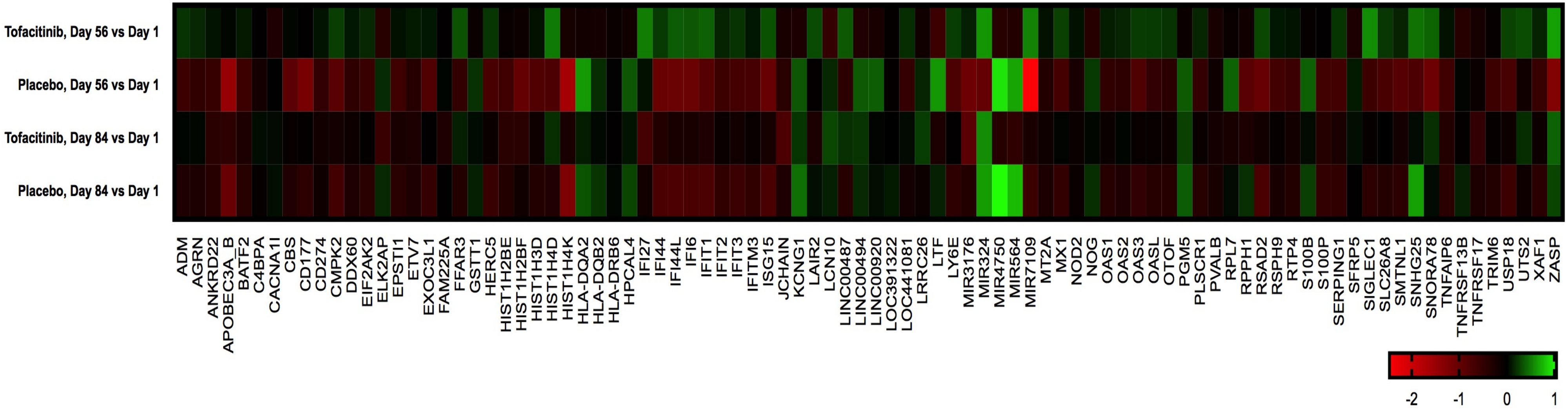

Supplemental Figure 3. Gene expression in peripheral blood by RNA sequencing. A total of 90 genes were found to be two-fold different (22 up and 68 down) by ANOVA comparison between tofacitinib and placebo treatment on day 56. Heat map shows Log2 fold change for these genes between day 56 vs day 1 and day 84 vs day 1 for Tofacitinib and Placebo treated patients.

Supplemental Figure 4

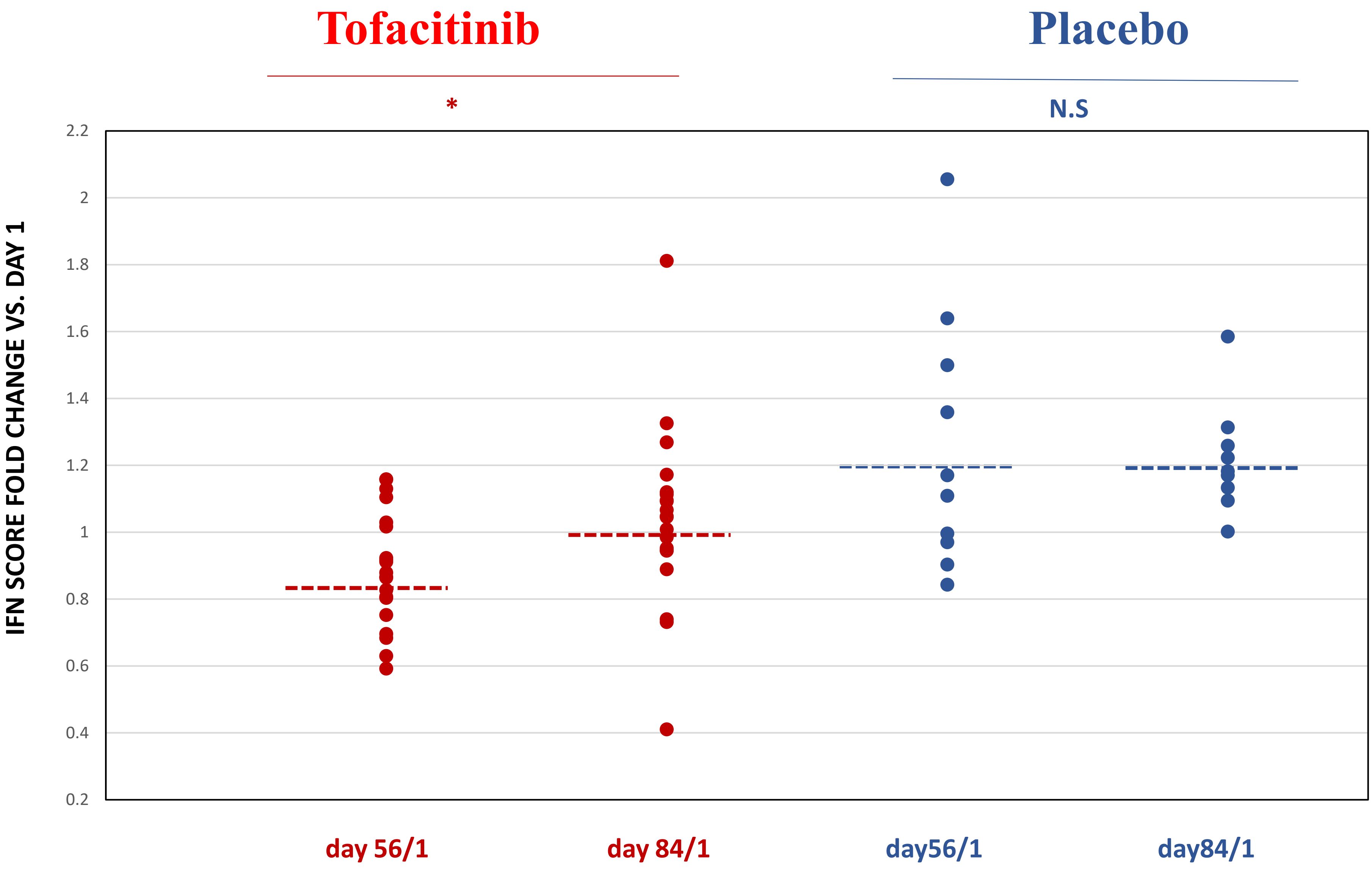

Supplemental Figure 4. IFN responsive gene score. Patients on tofacitinib had a significant reduction (p=0.01) in IFN score at day 56 in comparison to placebo and this difference reverted to baseline at day 84 off study drug period (p=0.02). The two tailed t tests was used.

No adjustments were made for multiple comparisons.

Supplemental Figure 5

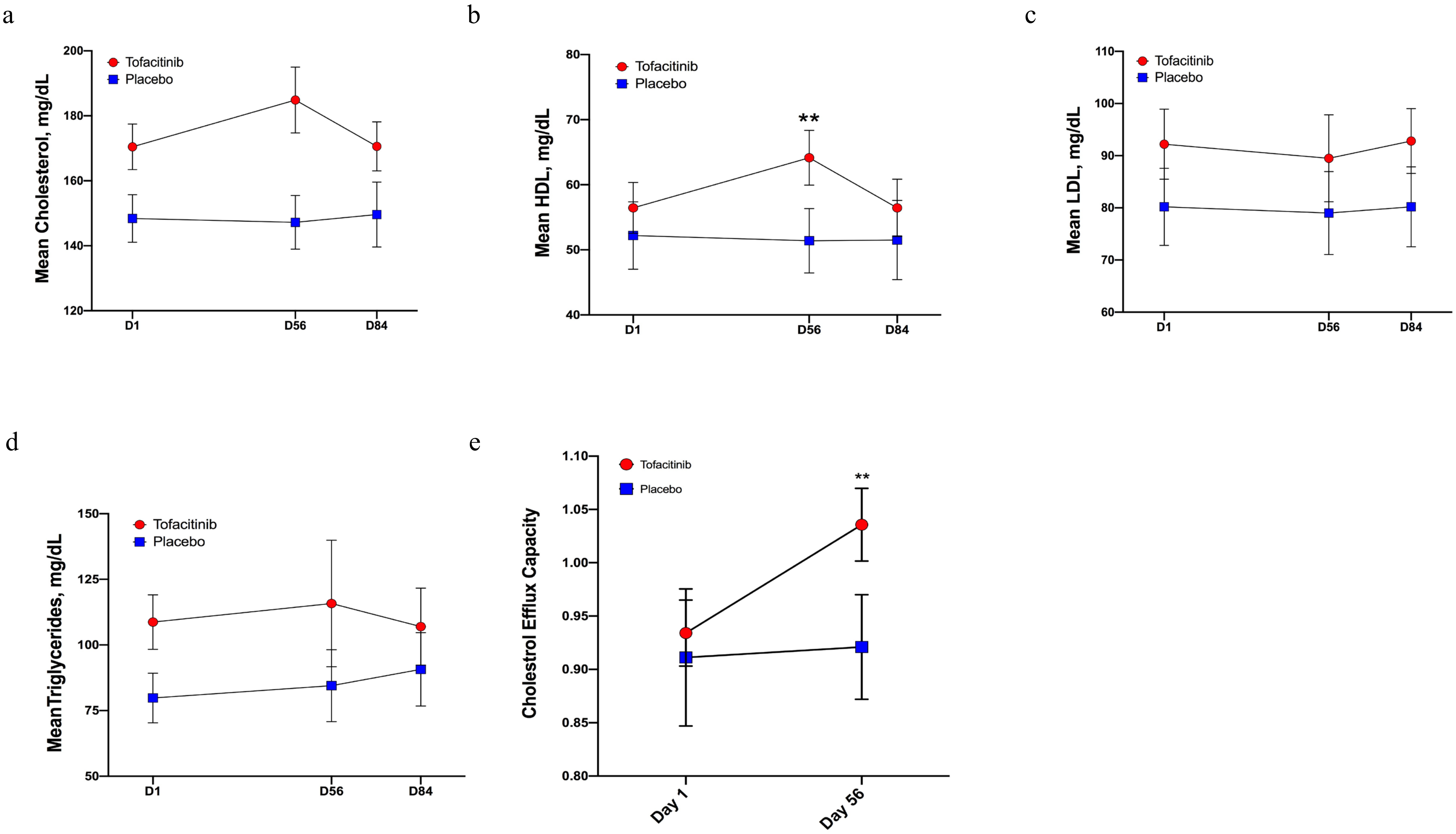

Supplemental Figure 5. Tofacitinib modulates lipoprotein levels and function in SLE. Results represent changes in a) Serum total cholesterol levels b) HDL-C **p=0.006 c) LDL-C d) triglyceride levels during the trial. e) Cholesterol efflux capacity: Results represent percentage change in efflux capacity in subjects on tofacitinib as compared to placebo at baseline and on day 56 **p=0.002. All results represent mean ± SEM, * p:<0.05 ** p:<0.01, and are based on tofacitinib n=20, placebo n=10.

Paired two tailed t- tests were used where appropriate. In addtion, Mann-Whitney u or ANOVA were used for comparison where appropriate based on normality of distribution.

No adjustments were made for multiple comparisons.

Supplemental Figure 6

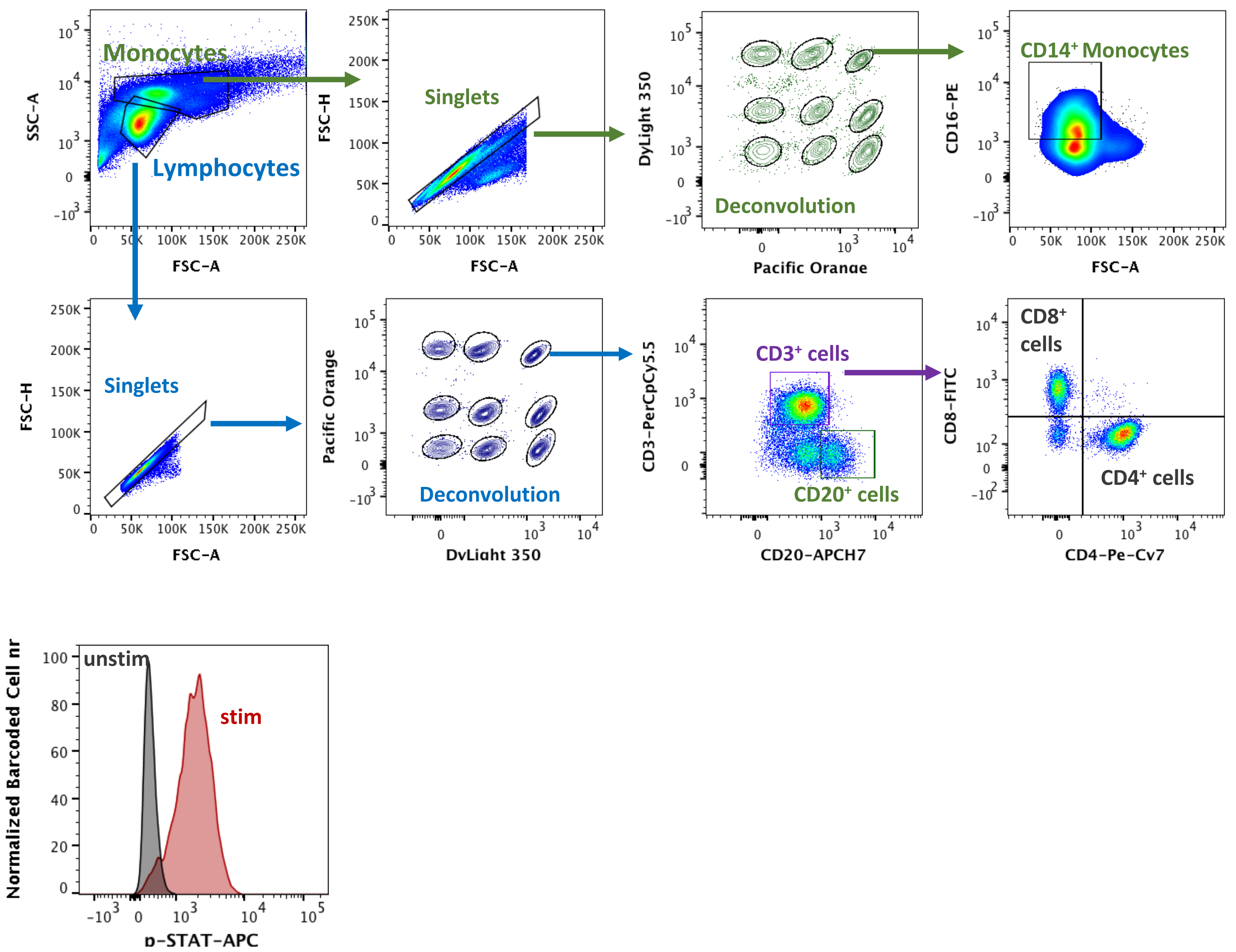

Supplemental Figure 6. Gating strategy. Lymphocytes and monocytes were identified using linear parameters (FSC-A vs SSC-A), double cells excluded (FSC-A vs FSC-H), and deconvolution carried out on each population using FCB dye channels (DyLight 350 vs Pacific Orange). On barcoded monocytes, $CD14^+$ cells were identified using linear parameter and corresponding fluorochrome channel (CD14-PE vs FSC-A). On barcoded lymphocytes, $CD3^+$ and $CD20^+$ cells were first gated and CD4 and CD8 expression was further investigated on $CD3^+$ cells. Then, on $CD4^+$, $CD8^+$, $CD20^+$ and $CD14^+$ populations, pSTATs expression was studied by calculating MFI values from stimulated and unstimulated samples. pSTAT expression is displayed by normalized cell count histograms using unstimulated samples (grey) and matched stimulated specimens (red).

Supplementary References:

1. Biancotto A, McCoy JP. Studying the human immunome: the complexity of comprehensive leukocyte immunophenotyping. Current topics in microbiology and immunology. 2014;377:23-60.
2. Olnes MJ, Kotliarov Y, Biancotto A, Cheung F, Chen J, Shi R, et al. Effects of Systemically Administered Hydrocortisone on the Human Immunome. Scientific reports. 2016;6:23002.
3. Ichihara A, Yamashita N, Takemitsu T, Kaneshiro Y, Sakoda M, Kurauchi-Mito A, et al. Cardio-ankle vascular index and ankle pulse wave velocity as a marker of arterial fibrosis in kidney failure treated by hemodialysis. American journal of kidney diseases : the official journal of the National Kidney Foundation. 2008;52(5):947-55.
4. Marder W, Khalatbari S, Myles JD, Hench R, Lustig S, Yalavarthi S, et al. The peroxisome proliferator activated receptor-gamma pioglitazone improves vascular function and decreases disease activity in patients with rheumatoid arthritis. J Am Heart Assoc. 2013;2(6):e000441.
5. Doupis J, Papanas N, Cohen A, McFarlan L, Horton E. Pulse Wave Analysis by Applanation Tonometry for the Measurement of Arterial Stiffness. The open cardiovascular medicine journal. 2016;10:188-95.

CLINICAL RESEARCH PROTOCOL

NATIONAL INSTITUTE OF ARTHRITIS AND MUSCULOSKELETAL AND SKIN DISEASES

PROTOCOL NUMBER: Initial Protocol
PROTOCOL VERSION: 1.2

PROTOCOL TITLE: Safety of tofacitinib, an oral Janus kinase inhibitor, in Systemic Lupus Erythematosus; a Phase Ib clinical trial and associated mechanistic studies

SHORT TITLE: JAK-IN-LUPUS

IDENTIFYING WORDS: JAK-IN-LUPUS

Date: August 2, 2015

Principal Investigator:

Name, Degree	Branch/Institute	Bldg/Rm	Phone	E-mail
Sarfaraz Hasni, M.D.	SAB/NIAMS	10/3-2340	301-451-1599	hasnisa@mail.nih.gov

Associate Investigators:

Name, Degree	Branch/Institute	Bldg/Rm	Phone	E-mail
Mariana Kaplan, M.D.	SAB/NIAMS	10/6D47C	301-496-0517	Mariana.kaplan@nih.gov
John J. O'Shea, M.D.	MIIB, NIAMS	10/13C103	301-496-6026	osheajo@mail.nih.gov
James Balow, M.D	NIDDK	10/5-2551	301-496-4181	jmb@mail.nih.gov
Massimo Gadina, Pharm D, Ph.D.	TIS/OST/NIAMS	10/6D47A	301-443-5457	gadinama@mail.nih.gov
Yasuko Furumoto, Ph.D.	IRP/OST/TIS/NIAMS	10/10C211	301-594-6386	furumoty@ep.niams.nih.gov
Meryl Waldman, M.D.	NIDDK	10/ 5-2551	301-451-6990	waldmanm@niddk.nih.gov
Betty Diamond M.D.	FIMR	-	516-562-3830	BDiamond@NSHS.edu
Meggan MacKay M.D.	FIMR	-	516- 562-3838	mmackay@nshs.edu
Peter Gregersen M.D.	FIMR	-	516-562-1542	pgregers@nshs.edu
Cynthia Aranow M.D.	FIMR	-	516-562-3837	caranow@nshs.edu
Nehal Mehta M.D.	NHLBI	10/5-3142	301-827-0483	nehal.mehta@nih.gov
Alan Remaley M.D.	NHLBI	10/2C433	301-402-9796	Alan.remaley@nih.gov

Daniella Schwartz M.D.	OCD/NIAMS	10/13C10 3	301-443-8544	Daniella.schwartz@nih.gov
Shubhasree Choudhury M.D.	OCD/NIAMS	10/6N226	301-451-7616	Shubhasree.choudhury2@nih.gov
Elizabeth Joyal, MSN, RN	OCD/NIAMS	10/3-2340	301-435-4489	ejoyal@mail.nih.gov
Alice Fike, MSN, CRNP	OCD/NIAMS	10/3-2340	301-496-4644	fikeam@mail.nih.gov
Yenealem Temesgen-Oyelakin, BSN,RN,	OCD/NIAMS	10/3-2340	301-451-4990	Yenealem.temesgen-oyelakin@nih.gov
Simantini Sakhardande, MD	SAB/NIAMS	10/6D44	301-594-2120	Simantini.sakhardande@nih.gov
Ann Biehl Pharm D.	CC/ODDCC/Pharm	10/1C240	301-496-4363	Ann.biehl@nih.gov

Key Research Personnel:

Name, Degree	Branch/Institute	Bldg./Rm	Phone	E-mail
Zerai Manna, MSc	SAB/NIAMS	10/4-1341	301-827-0546	zerai.manna@nih.gov
Yinghui Shi	SAB/NIAMS	10/6D46	301-435-8044	yinghui.shi@nih.gov

Research Contact:

Name, Degree	Branch/Institute	Bldg/Rm	Phone	E-mail
Yenealem Temesgen-Oyelakin, BSN,RN	OCD/NIAMS	10/3-2340	301-451-4990	Yenealem.temesgen-oyelakin@nih.gov

ESTIMATED DURATION OF STUDY: 5 years

START DATE: pending END DATE:

NUMBER AND TYPE OF PATIENTS: 30 Lupus Patients
Accrual Ceiling: 38

	Number	Sex	Age Range
Lupus Patients:	30	Females & Males	≥18 ages

PROJECT USES IONIZING RADIATION:
__X__ Medically indicated:

IND/IDE: IND exempt
Sponsor: NIAMS
Signature Page:

The signature below constitutes approval of this protocol and attachments, and provides the necessary assurances that this study will be conducted according to all stipulations of the protocol, including all statements regarding confidentiality, and according to local laws and regulatory requirements, applicable U.S. federal regulations, and guidelines established by the International Conference on Harmonization.

____

Principal Investigator (Signature) Date

Table of Contents

CLINICAL RESEARCH PROTOCOL 1
NATIONAL INSTITUTE OF ARTHRITIS AND MUSCULOSKELETAL AND SKIN DISEASES 1
TABLE OF CONTENTS 4
ABBREVIATIONS 8
PRÉCIS 10
INTRODUCTION/SCIENTIFIC RATIONALE: 12
1.1 Overview: 12
1.2 Systemic lupus erythematosus; description and epidemiology: 12
1.3 Current Treatment Paradigms in SLE: 13
1.4 Disease pathogenesis; cytokines in SLE: 13
Tofacitinib; description and summary of clinical studies: 15
2.1 Tofacitinib; mechanism of action: 17
2.2 Rationale for treatment with tofacitinib: 17
3. STUDY OBJECTIVES: 21
3.1 *Primary Objective:* 21
3.2 *Secondary Objectives:* 21
4. SUBJECTS: 22
4.1 Inclusion Criteria: 22
4.2 Exclusion Criteria: 23
4.3 Subject Completion and Replacement: 25
5 STUDY DESIGN AND METHODS: 25
5.1 Description of Study Design: 25
5.2 Description of Endpoints: 26
5.2.1 Primary Endpoint 26
5.2.2 Secondary Endpoints 26
5.3 Recruitment: 28
5.4 Screening Methods 28
5.5 Study Phases 28
5.6 Duration of Study Participation 29
5.7 Clinical Assessments 29
Vital Signs Measurement 29
5.8 Adverse event assessment 30
5.9 Assessment of lupus disease activity 30
5.10 Subject questionnaires 30
5.11 Clinical Laboratory Assessments 30
5.12 Laboratory Research Studies 31
Mechanistic studies 31
5.13 Visit Procedures: 32
5.13.2 Screening Period (—30 days to Day 1): 33
5.13.3 Day 1 +/- 7 days Study Visit: 34

5.13.4 Day 7+/- 7days, Day 21+/- 7days, Day 35 +/- 7days and Day 49 +/- 7days Telephone Calls ±7days : 36
5.13.5 Day 14 +/- 7days Study Visit: 36
5.13.6 Day 28 +/- 7 days Study Visit: 37
5.13.7 Day 42 +/- 7 days Study Visit: 38
5.13.8 Day 56 +/- 7 days Study Visit (end of treatment period): 39
5.13.9 Day 84 +/- 7 days (follow up visit, end of study) : 40
5.13.10 Unscheduled Visits: 42
5.14 Follow-up/Termination Procedures: 43
5.15 Study Drug, Dosing and Administration: 44
5.15.1 Tofacitinib: 44
5.15.2 Tofacitinib dosing: 44
5.15.3 Placebo: 44
5.16 Treatment Compliance: 44
5.17 Treatment Allocation: 45
5.17.1 Genotyping and Blinding: 45
5.18 Concomitant medications: 46
5.18.1 Restricted medications: 46
5.18.2 Disallowed medications: 47
5.18.3 Corticosteroids: 47
5.19 Treatment of flares: 47
5.20 Withdrawal criteria: 48
6 Risks and Discomforts: 49
6.1 Unexpected Adverse Events: 49
6.2 Risks/discomfort associated with tofacitinib: 49
6.3 Flare of SLE: 52
6.4 Risks associated with study procedures: 53
6.4.1 Blood draws 53
6.4.2 Electrocardiogram (ECG) 53
6.4.3 Vascular Function Studies 53
7 Safety Monitoring (see section 15) : 55
8 OUTCOME MEASURES: 55
9 STATISTICAL ANALYSIS 57
9.1 Analysis Populations: 57
9.1.1 Safety Population: 57
9.1.2 Efficacy Population: 57
9.2 Data Analysis: 57
Demographic and Baseline Characteristics 57
9.3 Primary outcome: 57
9.4 Secondary outcomes: 57
9.5 Exploratory Analyses: 58
9.6 Criteria for Significance: 58
9.7 Power Analysis: 58
9.8 Interim Analysis: 58
9.9 Accrual Number Request: 59
10 HUMAN SUBJECTS PROTECTION 59

10.1 Ethics and Good Clinical Practice, Data Collection and Data Quality Assurance:......... 59
10.4 Storage of Data and Samples:... 60
10.4.1 Samples: 60
10.4.2 Data: 61
10.4.3 Record Retention:... 61
10.4.4 Access to Source Data and Documents:.. 61
10.4.5 Data Collection, Quality Control and Quality Assurance Monitoring:..................... 62
10.5 Institutional Review Board:.. 64
10.6 Recruitment of women, children and minorities:.. 64
10.7 Subject selection:... 65
10.8 Qualification of Investigators:.. 65
11 BENEFITS:.. 67
12 SIGNIFICANCE TO BIOMEDICAL RESEARCH: 67
13 SUMMARY/CLASSIFICATION OF RISK:.. 67
14 INFORMED CONSENT DOCUMENTS AND PROCESS: 67
15 GCP COMPLIANCEAND DATA AND SAFETY MONITORING AND REPORTING: 68
15.1 Clinical Monitoring: 69
15.2 Data and Safety Monitoring Committee Oversight:.. 70
15.3 Suspension Guidelines: 70
16 ADVERSE EVENT AND UNANTICIPATED PROBLEM REPORTING:...................... 71
16.1 Definitions:... 71
16.1.1 Adverse Event: 71
16.1.2 Unanticipated Problem:... 71
16.1.3 Serious Adverse Event: 73
16.1.4 Medical Events Not Qualifying as Adverse Events or Serious Adverse Events: 73
16.1.5 Clinical Laboratory Test Results Not Qualifying as Adverse Events or Serious Adverse Events:.. 74
16.1.6 Lupus Flare:... 74
16.1.7 Reporting of Adverse Events, Unanticipated Problems and Protocol Deviations:........ 74
16.1.7.1 Intensity of Adverse Event:.. 74
16.1.7.2 Relationship to Study Drug and Procedures:... 74
16.1.7.3 Expectedness of Adverse Events:.. 75
16.1.8 Procedures for Reporting... 76
16.1.9 Reporting Timeline: 76
16.2 Adverse Event, Protocol deviation and Unanticipated Problem Assessment and Follow-up: 78
16.2.1 Adverse Event Recording:.. 78
16.2.2 Clinically Significant Laboratory Abnormalities:... 79
16.2.3 Pregnancy Reporting and Follow-up:... 79
16.2.4 Lost to follow up patient reporting:.. 80
17 ALTERNATIVES TO PARTICIPATION OR ALTERNATIVE THERAPIES:............... 81
18 CONFIDENTIALITY: 81
19 CONFLICT OF INTEREST/TECHNOLOGY TRANSFER:... 81
20 RESEARCH AND TRAVEL COMPENSATION:... 81
21 REFERENCES: 83

22 APPENDICES: ... 89
22.1 Schedule of Events ... 89
22.2 American College of Rheumatology Revised Classification Criteria for Systemic Lupus Erythematosus ... 90
22.3 SLEDAI 2K ... 91
SLEDAI-2K: DATA COLLECTION SHEET ... 91
22.4 BILAG 2004: ... 93
22.5 Disease Activity Score-28 (DAS-28): ... 94
22.6 The Cutaneous Lupus Erythematosus Disease Area and Severity Index (CLASI): ... 95
22.7 36-item Short Form Survey: ... 96
22.8 Multidimensional Assessment of Fatigue: ... 101
55 101
22.9 NIH Clinical Center guidelines for the management of allergic reactions: ... 105
22.10 XELJANZ (tofacitinib) patient prescribing information: ... 107
22.11 NCI Common Toxicity Criteria, Version 4.0: ... 107
22.12 Physician Global Assessment (PGA): ... 108

Abbreviations

AE	adverse event
AU	arbitrary unit
Anti-ds-DNA	Anti-double stranded DNA antibody
Anti-Sm	Anti-Smith antibody
Anti-SSA	Anti-Sjögren’s Syndrome A antibody
Anti-SSB	Anti-Sjögren’s Syndrome B antibody
ANA	Antinuclear Antibody
CBC	complete blood count
C3	Complement component 3
CD	Cluster of Differentiation
C4	Complement component 4
CTDB	Clinical Trials Data Base
CTSS	Clinical Trials Survey System
CRP	C-reactive protein
DAS-28	Disease Activity Score of the 28 joints
DSMC	Data and Safety Monitoring Committee
ESSDAI	EULAR Sjögren’s Syndrome Disease Activity Index
ESSPRI	EULAR Sjögren’s Syndrome Patient Reported Index
eCRF	electronic case report form
EKG	Electrocardiogram
EDC	electronic data capture
ESR	Erythrocyte sedimentation rate
FDA	Food and Drug Administration
GCP	Good Clinical Practice
HLA	Human Leukocyte Antigen
Ig G	Immunoglobulin subtype G
IgE	Immunoglobulin subtype E
IL-4	Interleukin 4

IU	International Units
ICF	Informed Consent Form
ICH	International Conference on Harmonization
IRB	Institutional Review Board
MHC	Major Histocompatibility Complex
MI	Myocardial Infarction
NIAMS	National Institute of Arthritis and Musculoskeletal and Skin Diseases
NIH	National Institutes of Health
NSAID	nonsteroidal anti-inflammatory drug
PK	Pharmacokinetics
PD	Pharmacodynamics
PT/PTT	Prothrombin time/Partial thromboplastin time
BILAG 2004	British Isles Lupus Assessment Group index
SLENA-SLEDAI	Safety of Estrogen in Lupus Erythematosus National Assessment-modification of Systemic Lupus Erythematosus Disease Activity Index
SLEDAI 2K	Systemic Lupus Erythematosus Disease Activity Index 2000
SAE	serious adverse event
SD	standard deviation
SF-36	Short Form-36
SLE	Systemic Lupus Erythematosus
SOP	Standard Operating Procedure
TBNK	T lymphocytes, B lymphocytes and Natural Killer cells
TNF-α	Tumor Necrosis Factor alpha
WBC	White Blood Cells

Précis

Background:

Systemic Lupus Erythematosus (SLE) is an autoimmune disease with variegated clinical presentation resulting from involvement of multiple biologic pathways. The pathways that lead to loss of tolerance in SLE include: multiple autoreactive cell types (B, T, dendritic, Th17 and regulatory T cells) and abnormal cytokine milieus, genetic factors, environmental and hormonal influences, all of which can influence cell differentiation patterns and reset tolerance checkpoints (1, 2). In addition, recent studies indicate a putative role for neutrophils in lupus pathogenesis and associated end-organ damage(3). Currently available therapeutics are frequently inadequate to treat disease flares and simultaneously expose patients to potentially serious toxicities. Further, premature cardiovascular disease not explained by the Framingham risk equation has become one of the most important causes of morbidity and mortality in this patient population. To date, no treatment used in lupus appears to significantly decrease cardiovascular risk. Identifying a drug that has immunomodulatory effects and is also vasculoprotective is an unmet need in this disease.

Tofacitinib is an orally administered Janus kinase (JAK) inhibitor that has recently been approved by the Food and Drug Administration for the treatment of moderate to severe rheumatoid arthritis (RA).

The JAKs are a family of intracellular enzymes (JAK1, 2 and 3 and TYK2) that mediate signaling via a broad range of cytokine receptors (4, 5). Targeting JAKs is an attractive therapeutic possibility for SLE for many reasons. Many of the inflammatory cytokines implicated in the pathogenesis of SLE signal via the JAK-STAT pathways. JAK inhibitors have been found to have efficacy in various murine models of lupus (6). Mice treated with a JAK2 inhibitor exhibited reduced serum levels of IL-6, and IL-17 along with reduced numbers of long-lived autoantibody producing plasma cells in the spleen and bone marrow (7). Furthermore, we have found that administration of tofacitinib reduced serum levels of ANA, IL-6, and IFN-γ; and ameliorated glomerulonephritis (unpublished data).

This study therefore represents an innovative investigative measure of the safety and efficacy of JAK inhibition in SLE that is predicted by genetic predisposition. We will also

investigate effects of tofacitinib on vascular function in SLE subjects and identify biomarkers that may be useful as endpoints in future studies.

Primary Objective:

To determine the safety and tolerability of tofacitinib in patients with SLE and mild to moderate disease activity.

Study Design:

This is a Phase Ib, randomized, double blind, placebo controlled clinical trial of orally administered tofacitinib, 5 mg twice daily, for the treatment of SLE subjects with mild to moderate disease activity stratified by the presence or absence of STAT4 risk alleles.

INTRODUCTION/SCIENTIFIC RATIONALE**:**

1.1 *Overview:*

The proposed research is an exploratory Phase Ib clinical trial, the primary focus of which will be to test the safety of tofacitinib, a JAK inhibitor, in SLE. A secondary goal will be to do exploratory mechanistic studies as a prologue to future studies and to identify candidate surrogate markers that might relate to clinical efficacy. The patient population for this trial will be stratified by the presence or absence of STAT4 risk alleles to investigate the effect(s) of these genetic haplotypes on response to tofacitinib. While the numbers of patients may be too small to discern significant effects, STAT4 genotyping will be performed as an exploratory effort. This clinical trial seeks to address an important medical problem: SLE is a chronic autoimmune disease that has no cure and current therapeutic strategies are limited in their efficacy and by their significant toxicities.

1.2 *Systemic lupus erythematosus; description and epidemiology:*

SLE is characterized by anti-nuclear antibody production and pathological findings of inflammation, vasculitis, vasculopathy and immune complex deposition in multiple target organs. Lupus occurs throughout the world and susceptibility is clearly modulated by ethnicity and gender. Although it affects both males and females of all age groups, it most commonly presents in women of reproductive age with a striking female to male ratio of approximately 9:1 (8). This ratio is approximately 2-3:1 in younger and older populations, supporting a role for hormonal factors in the induction of disease. Incidence rates reported during the last 25 years in North America vary from 2 to 7 per 100,000; rates in African-American, Afro-Caribbean, Hispanic and Asian populations are approximately three times greater than in Caucasian populations. The worldwide prevalence of lupus ranges from 17 to 48 per 100,000, but has been reported as high as 207/100,000 in an Afro-Caribbean population in the United Kingdom. While a precise etiology of SLE is not known, combinations of genetic, hormonal and environmental factors are thought to contribute to the loss of self-tolerance. In rare individuals, single gene mutations play a major role, e.g., 98% of individuals with complete deficiency of C1q develop SLE. For the vast majority of individuals with SLE the genetic contribution appears to be polygenetic. The importance of non-genetic contributors to disease factors (i.e., hormonal and environmental factors) is also apparent since disease discordance in monozygotic twins is at least 60%.

1.3 *Current Treatment Paradigms in SLE:*

The current management of patients with SLE is usually stratified by the degree of internal organ involvement; however, most treatment strategies include a variety of immunosuppressive medications that are limited both in their efficacy and by their potential toxicities (reviewed in (9)). FDA-approved treatments for SLE include only hydroxychloroquine, corticosteroids, aspirin, and most recently, belimumab. NSAIDs are relatively contraindicated in patients with renal disease and potential adverse effects on photosensitivity, aseptic meningitis and the gastrointestinal tract also limit their use in SLE. Potentially devastating side effects of corticosteroids are well-known and include infection, avascular necrosis, weight gain, osteoporosis, cataracts and development of diabetes. The use of immunosuppressive drugs for musculoskeletal symptoms refractory to NSAIDs or requiring continued steroid therapy has become “standard of care” by the rheumatologic community. Although often beneficial for treatment of active disease, these immunosuppressive medications (ex: azathioprine, methotrexate, mycophenolic acid, leflunomide, cyclophosphamide, cyclosporine) are associated with multiple toxicities; most commonly infection (with potentially fatal outcomes), hepatic and renal impairment and infertility. Despite the addition of these potentially toxic agents, SLE patients usually require continued treatment with corticosteroids. Thus, lupus patients are typically dependent indefinitely on corticosteroids and/or immunosuppressive agents for disease control even while developing cumulative toxicities from exposure to these drugs. Clearly there is an unmet need for improved treatment of inflammation in this patient population. Further, premature cardiovascular disease has become one of the most important causes of morbidity and mortality in this patient population and it is not explained by the Framingham risk equation. To this date, no drug used in lupus appears to significantly decrease cardiovascular risk. Therefore, identifying a drug that has both immunomodulatory and vasculoprotective effects would fill an important unmet need in this disease.

1.4 *Disease pathogenesis; cytokines in SLE:*

End organ damage in SLE is the consequence of a series of events characterized by loss of tolerance resulting in autoantibody production and ending in an inflammatory cascade that leads

to tissue destruction. Auto-reactive B and T cells as well as abnormally primed dendritic cells all contribute to the abnormal immune response in SLE. However, at a cellular level, much of the tissue destruction appears to be cytokine mediated. A variety of cytokines, including: IL-1, IL-2, IL-6, IL-10, IL-18, IL-21, IFN α, IFN γ, and TNF α, have been implicated in the immunopathogenesis of SLE. These molecules mediate tissue destruction and contribute to breaking tolerance through effects on B cell activation, immunoglobulin production and expression of costimulatory molecules on lymphocytes.(10-12) Immune complexes from SLE stimulate peripheral blood mononuclear cells (PBMCs) to express the highly pro-inflammatory cytokines TNF α and IFNα via Fc gamma receptor and TLR 9 mechanisms(13-15). Interestingly, deficiencies in Fc gamma receptors or the inability to produce high levels of IFN γ are protective against acute glomerulonephritis in murine models of SLE.(16, 17) IFN γ, IL-1β, TNF α, TGFβ, IL-18 and IL-6 are all expressed at high levels in the kidneys of mice with active glomerulonephritis leading to increased inflammation, hypercellularity, fibrosis and renal dysfunction (18-20). The same array of cytokines has also been identified in kidney biopsy tissue from SLE patients with active glomerulonephritis (11, 21, 22) and some of these cytokines have also been found in the urine of patients with active nephritis (11, 23, 24). Skin biopsies from SLE patients have yielded increased expression of IFN α and IL-6 in active sites (25-27). There are conflicting reports on circulating levels of all of these cytokines in SLE patients; it is assumed that the tissue levels (understandably difficult to measure) are truly reflective of the pathologic process and reports of cytokine levels in tissue specimens in murine models and humans have been reasonably consistent.

The idea of uncoupling autoantibody/immune complex formation from cytokine production and directing therapy at known cytokines is not without precedent; TNF α blockade has revolutionized the treatment of rheumatoid arthritis. Thus far, in SLE, anti- IL-10 (28, 29) and anti-TNF α (30-32) have been used successfully in murine models. Clinical trials of TNFα inhibitors in human SLE have been halted due to safety concerns. In addition, recent reports from clinical trials indicate that anti-IFNα and anti-IL-10 therapy (33), though well-tolerated, may not be effective in human disease. These data suggest that a multi-targeted approach to this disease using a JAK inhibitor may be beneficial.

Tofacitinib; description and summary of clinical studies:

Tofacitinib (CP-690550/XELJANZ; Pfizer) is an orally administered JAK inhibitor that has recently been approved for the treatment of rheumatoid arthritis (RA) and is currently being developed for use in inflammatory bowel disease, psoriasis, ankylosing spondylitis, juvenile arthritis and renal allograft transplantation. Clinical trials of tofacitinib in RA demonstrate rapid onset of drug efficacy with an acceptable safety profile (34-38). These clinical trials have used tofacitinib as 1) monotherapy in patients failing non-biologic or biologic disease modifying drugs (DMARDS), 2) in combination with methotrexate in patients failing methotrexate or TNF inhibitors and the combined results are shown in Table 1.

Table 1: Proportion of Patients with an ACR Response Percent of Patients									
Monotherapy in Nonbiologic or Biologic DMARD Inadequate Responders[c]				MTX Inadequate Responders[d]			TNF Inhibitor Inadequate Responders[e]		
Study I				Study IV			Study V		
N[a]	PBO 122	XELJANZ 5 mg Twice Daily 243	XELJANZ 10 mg Twice Daily 245	PBO + MTX 160	XELJANZ 5 mg Twice Daily + MTX 321	XELJANZ 10 mg Twice Daily + MTX 316	PBO + MTX 132	XELJANZ 5 mg Twice Daily + MTX 133	XELJANZ 10 mg Twice Daily + MTX 134
ACR20 Month 3 Month 6	26% NA[b]	59% 69%	65% 70%	27% 25%	55% 50%	67% 62%	24% NA	41% 51%	48% 54%
ACR50 Month 3 Month 6	12% NA	31% 42%	36% 46%	8% 9%	29% 32%	37% 44%	8% NA	26% 37%	28% 30%
ACR70 Month 3 Month 6	6% NA	15% 22%	20% 29%	3% 1%	11% 14%	17% 23%	2% NA	14% 16%	10% 16%

[a] N is number of randomized and treated patients.
[b] NA Not applicable, as data for placebo treatment is not available beyond 3 months in Studies I and V due to placebo advancement.
[c] Inadequate response to at least one DMARD (biologic or nonbiologic) due to lack of efficacy or toxicity.
[d] Inadequate response to MTX defined as the presence of sufficient residual disease activity to meet the entry criteria.
[e] Inadequate response to a least one TNF inhibitor due to lack of efficacy and/or intolerance.

Improvement with doses of 5 mg twice daily was noted as early as two weeks, with ACR 20 responses reported in 41-59% and 51-69% of subjects at 3 and 6 months respectively. Similarly, ACR 50 responses were reported in 26-31% and 32-42% of subjects at 3 and 6 months respectively. In general, tofacitinib was well tolerated; the most common toxicity reported was infection with an overall frequency of 20% in the patients who received tofacitinib 5 mg twice daily and 18% in the patients who received placebo. The most commonly reported infections were upper respiratory infections, throat infections and urinary tract infections.

Serious infections, pneumonia, cellulitis, herpes zoster, and urinary tract infections due to bacterial or opportunistic organisms (Herpes simplex, Cytomegalovirus, Cryptococcus, Pneumocystis and Candida) were observed in 0.4% of subjects who received drug for 3 months or less. Other toxicities, including malignancy and gastrointestinal perforation were rare. Laboratory abnormalities, including lymphopenia, neutropenia, anemia, increased transaminases and elevated high density lipoprotein (HDL) and low density lipoprotein (LDL) were also considered mild and reversible. The lymphopenia (absolute lymphocyte count <500 cells/mm3) was associated with an increased risk of infection but the neutropenia was not. Pooled data from 2 open-label long-term extension studies involving 4102 patients treated for 5963 patient-years reported a cardiovascular events incidence rate range from 0.05-0.3 per 100 patient-years (39) . Increase in serum creatinine of more than 50% from baseline was observed in 3.3 % of the patients(39).

2.1 Tofacitinib; mechanism of action:

The JAKs are a family of intracellular enzymes (JAK1, 2 and 3 and TYK2) that facilitate signaling between inflammatory cytokines bound to surface receptors and subsequent gene transcription that regulates immune response (4). Specifically, JAKs phosphorylate cytoplasmic tails of cytokine receptors once the receptor has been bound by its cognate cytokine. This leads to recruitment of appropriate Signal Transduction and Transcription (STAT) proteins, which are in turn phosphorylated. This leads to dimerization and disassociation from the receptor-JAK complex. STATs translocate to the nucleus where they regulate gene transcription. Specific cytokine receptor subunits selectively associate with different JAKS and STAT (Fig. 1). JAKs play a critical role in mediating inflammatory responses and pharmacologic intervention that modulates JAK function represents a novel approach to the treatment of autoimmune disease.

Figure 1. Associations between JAK kinases, cytokines and STAT proteins

γ chain cytokines; IL-2, 4, 7, 9, 15, 21	→	JAK1<br>JAK3	→	STAT 1, 3, 5, 6
IL-6, IFNγ	→	JAK1<br>JAK2	→	STAT 1, 2, 3
IL-23, 12, IFNα	→	TYK2<br>JAK2	→	STAT 4, 3
EPO, TPO	→	JAK2<br>JAK2	→	STAT 3, 5

Tofacitinib has been shown to inhibit JAK1, JAK3 and JAK2, however, cellular assays that measure cytokine-induced STAT phosphorylation have demonstrated partial selectivity of tofacitinib for JAK 1 and 3 resulting in greater inhibition of JAK 1 and 3 compared to JAK 2(40). JAK1 and JAK3 mediate intracellular responses for cytokines that use the gamma chain, which are critical for the development and function of T, B, and NK cells. Other in vitro studies suggest that the effectiveness of tofacitinib in RA may be attributable to its suppressive effects on the generation of Th1 and pathogenic Th17 cells (41-43). Tofacitinib also inhibits cytokines that provide B cell help such as IL-4, IL-6, and IL-21. In addition, tofacitinib inhibits the effect of IL-6 and IFN-α on innate immune cells.

2.2 Rationale for treatment with tofacitinib:

2.2.1 Cytokine signaling: Targeting JAKs is an attractive therapeutic possibility for SLE for many reasons. Many of the inflammatory cytokines implicated in SLE

pathogenesis, in particular IFNα, IL-6, IL-23, IL-12, IL-21 and IFNγ, signal through JAK/STAT pathways. These molecules mediate tissue destruction and contribute to breaking tolerance through effects on B cell activation, immunoglobulin production and expression of costimulatory molecules on lymphocytes (10-12). Immune complexes from SLE stimulate peripheral blood mononuclear cells (PBMCs) to express the highly pro-inflammatory cytokines TNFα and IFNα via Fc gamma receptor and TLR 9 mechanisms (13-15). Interestingly, deficiencies in Fc gamma receptors or the inability to produce high levels of IFNγ are protective against acute glomerulonephritis in murine models of SLE (16, 17) and IFN γ genetic polymorphisms are associated with disease susceptibility (44). IFNγ, IL-1β, TNFα, TGFβ, IL-18 and IL-6 are all expressed at high levels in the kidneys of mice with active glomerulonephritis leading to increased inflammation, hypercellularity, fibrosis and renal dysfunction (18-20). The same array of cytokines has also been identified in kidney biopsy tissue from SLE patients with active glomerulonephritis (11, 21, 22) and some of these cytokines have also been found in the urine of patients with active nephritis (11, 23, 24). Skin biopsies from SLE patients show increased expression of IFNα and IL-6 in active sites (25-27). Following engagement of its receptor, IFNα activates JAKs and STATs leading to increased expression of MHC Class I, dendritic cell maturation, T cell survival and autoantibody production (45). There are currently several monoclonal antibodies directed against different isoforms of IFNα in clinical trials for SLE treatment. Known functions of IL-6 are to enhance B cell differentiation into immunoglobulin secreting plasma cells and promote antibody production through stimulation of IL-21 by CD4+ T cells (46, 47). IL-6 is an important factor that induces naïve CD4+ cells to differentiate into Th17 cells through activation of STAT3 and induction of RORγt (48). Additionally, anti-IL-6 monoclonal antibodies are currently being tested in clinical trials for a number of autoimmune diseases including SLE. Positive anti-inflammatory effects of anti-IL-6 therapy are attributable, in part, to diminished support of long lived plasma cells by IL-6. IL-17 is recognized as an important contributor to lupus pathogenesis through its effects on neutrophils and monocytes and T cell

migration into tissues. Increased numbers of IL-17-producing T cells have been identified in SLE patients and in renal biopsies from lupus nephritis patients. Positive correlations between disease activity and IL-17 producing T cells have also been reported (49-51). A selective JAK2 inhibitor has been used to successfully treat established SLE and nephritis in murine models of SLE. These mice exhibited reduced serum levels of IL-6 and IL-17 along with reduced numbers of long-lived autoantibody producing plasma cells in the spleen and bone marrow (at higher doses) (7). IL-23 is mainly secreted by antigen presenting cells and signals through JAKs and STATs to promote expansion of IL-17 producing cells (including Th17 cells), increase IFNγ production, activate memory T cell responses and increase production of pro-inflammatory cytokines (rev in (52)). Elevated mRNA expression of IL-23 has been correlated with disease activity and renal disease in human SLE (53-55) and IL-23 receptor-deficient MRL/lpr mice do not develop renal disease (56). Given the importance of these pro-inflammatory cytokines that signal through JAKs and STAT proteins to the inflammatory response in lupus, use of tofacitinib has the potential to block effects of several cytokines known to exacerbate inflammatory responses in SLE rather than singling out one cytokine target at a time.

2.2.2 T cell pathology in SLE and JAK inhibition: The importance of T cell contributions to pathology in SLE is well established (57). The MHC locus remains the strongest genetic association with SLE, supporting the role of T cell-driven auto-reactivity. The plethora of high affinity, class-switched IgG autoantibodies characteristic of SLE reflect the role of follicular T helper (T_{fh}) cells in B cell proliferation and differentiation giving rise to autoantibody producing plasma cells (58). T_{fh} cells are generated from naïve CD4+ T cells in the presence of IL-6, IL-21 and ICOS stimulation. Inflammation is locally driven in target organ tissue in response to cytokines that regulate vascular permeability and enhance local extravasation of inflammatory cells into tissue. Th17 cells are generated from naïve CD4+ T cells in the presence of TGFβ, IL-1β, IL-6, IL-21 and IL-23 and they moderate inflammatory responses by releasing the pro-inflammatory cytokines IL-17 and IL-21. Engagement of the IL-17 receptor on

target cells leads to chemokine production and results in leukocyte recruitment and production of other inflammatory cytokines such as IL-1β and TNFα. Th17-related pathology is described in multiple autoimmune diseases including RA, psoriasis, inflammatory bowel disease, and SLE (59). High levels of IL-17 from Th17 cells and CD4-, CD8- (double negative, DN) T cells correlate with disease activity in SLE (60) and both of these cell types have been identified within inflammatory infiltrates in human lupus nephritis (49, 61) and murine models of lupus nephritis. DN T cells, though rare in healthy individuals, are expanded in SLE, produce pro-inflammatory cytokines (IFNγ, IL-17, IL-1β) and target organ tissue.

Ghoreschi et al have demonstrated, through a series of in vitro cellular assays and in vivo animal models, the range of tofacitinib's effects on T cell function and inflammatory cytokines (41). Tofacitinib inhibits JAK3 dependent γ_c cytokine receptor signaling and other JAK1 dependent cytokine receptor signaling. These blocking effects manifest as decreased production of inflammatory IL-23 dependent Th17 cells. Additionally, tofacitinib blockade of JAK1 and JAK3 inhibited Th1 and Th2 differentiation and blocked IL-6 signaling. In the animal model of collagen-induced arthritis, mice experienced a significant reduction in established arthritis within 48 hours of treatment with tofacitinib and circulating inflammatory markers decreased within 4 hours; demonstrating a remarkably fast onset of action. These immediate results were not associated with leukocyte depletion; histologic confirmation of leukocyte depletion took 7 days. Others have demonstrated that treatment of human subjects with tofacitinib for prevention of graft rejection after kidney transplant results in a differential effect of tofacitinib on Treg cells and Th1 effector cells where the Th1 effector cells proved more susceptible to JAK blockade (62). These studies have direct implications for the use of JAK inhibition in SLE where, similar to the positive results demonstrated in RA, we predict that inhibition of signaling molecules with broad effects on inflammatory responses will prove to be beneficial.

The reason for a Phase 1b study of tofacitinib in SLE is that tofacitinib also inhibits cytokines that may be protective in SLE. IL-2 is an important immunoregulatory cytokine that promotes immune responses but it is also important for peripheral tolerance. IL-2 promotes expression of

Foxp3 in regulatory T (Treg) cells. It also inhibits IL-17 production and Bcl6 expression; Bcl6 is important for T_{fh} cells and germinal center formation. While there is no evidence in mouse models or in humans treated with tofacitinib that this drug promotes autoimmunity, this remains a theoretical possibility. Regulatory T cells (Tregs), identified by the presence of the FOXP3 intracellular transcription factor, help modulate the duration and intensity of inflammatory responses. Decreased numbers of circulating Tregs have been correlated with disease activity in human SLE (63, 64) although other studies report normal levels in active disease (65). It is not clear whether Tregs are intrinsically less effective at suppression of inflammation in SLE or whether target cells are more resistant. Some studies have demonstrated inhibition of immune complex-mediated glomerulonephritis in murine models with adoptive transfer of Tregs (66, 67).

In addition, tofacitinib can inhibit JAK2 and thereby interfere with the action of erythropoietin, GM-CSF and IL-7. Consequently, tofacitinib could theoretically exacerbate the anemia and lymphopenia associated with SLE.

Tofacitinib treatment is also associated with higher rates of BK virus nephropathy. Patients with SLE were found to be asymptomatic career of BK virus in urine (32%) in one cross sectional cohort study. Hence there is a possibility of BK virus activation leading to clinically significant disease after starting patients on tofacitinib. We will include assessment of BK virus infection at baseline and throughout the study for an additional safety measure.

3. STUDY OBJECTIVES:

3.1 *Primary Objective:*

- To determine the safety and tolerability of tofacitinib in patients with Systemic Lupus Erythematosus and mild to moderate disease activity.

3.2 *Secondary Objectives:*

- To assess clinical improvement after treatment with tofacitinib as measured by improvement in the Systemic Lupus Erythematosus Disease Activity Index (SLEDAI 2K) and no worsening on the physician’s global assessment scale.
- To demonstrate that treatment with tofacitinib is more effective clinically and biologically in a subset of SLE subjects with STAT4 risk alleles than those without.

- To demonstrate that treatment with tofacitinib is more effective clinically and biologically in a subset of SLE subjects with mild to moderate disease as compared to placebo.
- To demonstrate that oral administration of tofacitinib does not result in increased disease activity as measured by the SLEDAI 2K disease activity index.
- To investigate the effects of tofacitinib on intracellular signaling molecules, serum cytokines and peripheral blood gene expression as a measure of biological effects that can be used as outcome measures to power a Phase II Clinical Trial.
- To investigate the effects of tofacitinib on patient quality of life measures as assessed by the patients' global assessment of disease activity, the Multidimensional Assessment of Fatigue questionnaire and the Short Form 36 health survey.
- To investigate the effects of tofacitinib in modulation of endothelial responses and markers of vascular risk in SLE.

4. SUBJECTS:

Adult SLE patients with mild to moderate disease activity will be eligible for the study. These patients can be naïve or failed immunosuppressive therapy beyond anti-malarials and glucocorticoids. We plan to enroll immunosuppressive therapy naïve patients as well as patients who have failed past therapies so as not to bias the cohort to patients with more recalcitrant disease. We expect that tofacitinib is a potential second line therapy, in addition to anti-malarials and glucocorticoids, depending on the patient's initial presentation and response and thus would like to include such patients in this study. Patients on the study will be followed weekly and thus any indication of worsening disease will be assessed promptly and patients will be withdrawn from the study accordingly for standard of care treatment as detailed in study withdrawal criteria. The following are the inclusion and exclusion criteria:

4.1 Inclusion Criteria:

Subjects who meet all of the following criteria are eligible for enrollment into the study:

1. Subject is capable of providing written informed consent.
2. Subject is ≥ 18 years old.

3. Meets at least 4 of 11 modified American College of Rheumatology (ACR) (1997) Revised Criteria for the Classification of Systemic Lupus Erythematosus
4. Has mild to moderate disease activity defined as a SLEDAI 2K score between 4-14.
5. If on glucocorticoids, the dose must be ≤20 mg daily and stable for the 4 weeks prior to screening visit.
6. If on hydroxychloroquine, the dose must have been stable for the 12 weeks prior to screening visit. The maximum allowed dose is hydroxychloroquine up to 400 mg/day or 6.5 mg/kg/day if more than 400 mg/day.
7. Males and females with potential for reproduction must agree to practice effective birth control measures (2 approved methods of contraception).

4.2 Exclusion Criteria:

Subjects who meet any of the following criteria are disqualified from enrollment in the study:

1. Pregnant or lactating women. Women of childbearing potential are required to have a negative pregnancy test at screening.
2. Women of childbearing potential and fertile men who are not practicing or who are unwilling to practice two forms of birth control during and for a period of 3 months after the completion of the study. Acceptable forms of birth control include abstinence, barrier methods, implantable intrauterine devices and oral, transdermal patch or injectable contraceptives.
3. Current or prior treatment with rituximab, belimumab or any other biologic agent in the 12 months prior to screening.
4. Current treatment with immunosuppressive drugs (methotrexate, azathioprine, mycophenolate mofetil, cyclosporine, tacrolimus). Glucocorticoids are allowed as per the inclusion criteria. At the investigator’s discretion, glucocorticoids may be tapered during the study.
5. Patients previously on azathioprine, mycophenolate mofetil, cyclosporine or tacrolimus should have stopped it for at least 8 weeks at the time of screening.
6. Treatment with cyclophosphamide, pulse methylprednisolone or IVIG within the 6 months prior to screening.

7. Increase in glucocorticoid dose within 4 weeks of screening.
8. A history of drug or alcohol abuse within the 6 months prior to screening.
9. History of chronic liver disease or elevated LFTs:
   - ALT or AST ≥ 2x upper limit of normal at screening
   - serum unconjugated bilirubin > 2mg/dL at screening
10. Dialysis or serum creatinine >1.5mg/dL.
11. Protein to creatinine ratio of more than 1000 mg/mg or 24 hours urine protein of more than 1000 mg.
12. Active urinary sediment (WBC, RBC or mixed cellular casts 1+ or more /hpf).
13. Hypercholesterolemia: Values after an 8-12 hour fasting blood specimen: total cholesterol >250 mg/dL or LDL >180 mg/dl or hypertriglyceridemia (triglyceride >300 mg/dL) at screening visit.
14. Active infection that requires the use of oral or intravenous antibiotics and has not resolved at least 2 weeks prior to the administration of the first dose of study medication.
15. Active chronic infections including but not limited to HIV, Hepatitis B, Hepatitis C, and BK viremia at screening visit.
16. History of cancer, excluding skin cancers (squamous cell or basal cell that have been treated).
17. Known active tuberculosis or untreated latent tuberculosis.
18. History of opportunistic infections.
19. Subjects with active renal or central nervous system disease or a BILAG A in any organ system.
20. WBC <2500/µL or ANC <1,000/µL, Hgb <9.0 g/dL or platelets <70,000/µL or absolute lymphocyte count < 500/µL.
21. Current treatment with potent inhibitors of Cytochrome P450 3A4 (CYP3A4) (e.g., ketoconazole) or receiving one or more concomitant medications that result in both moderate inhibition of CYP3A4 and potent inhibition of CYP2C19 (e.g., fluconazole) that would increase serum availability of tofacitinib. Past treatment with the above mentioned agent is allowed if it was more than a week prior to the administration of the first dose of study medication.

22. Significant impairment of major organ function (lung, heart, liver, kidney) or any condition that, in the opinion of the Investigator, would jeopardize the subject's safety following exposure to the study drug.

4.3 Subject Completion and Replacement:

A subject is considered to have completed the study when he/she has completed 56 days of treatment with tofacitinib and the Day 84 follow-up assessments.

Enrolled subjects who withdraw from the trial prior to starting on study drug will be replaced. Subjects who discontinue study treatment and/or withdraw from the trial for any reason other than drug related adverse events or lack of efficacy after initiating the first dose of tofacitinib and before completing 38 doses of tofacitinib will also be replaced in order to maintain the sample size of 10 subjects per group and a total of 30 subjects. Based on experience with studies in SLE patients, we expect a withdrawal rate of approximately 25% and therefore plan for a recruitment ceiling of 38 subjects.

5 STUDY DESIGN AND METHODS:

5.1 Description of Study Design:

This is a Phase Ib, randomized, double blinded, placebo controlled clinical trial of orally administered tofacitinib, 5 mg twice daily, for treatment of SLE subjects with mild to moderate disease activity stratified by the presence or absence of STAT4 risk alleles. There will be 3 arms in the study with 10 subjects in each arm and a recruitment ceiling of 38 subjects. Twenty subjects will be randomized to receive treatment with tofacitinib 5 mg twice daily; 10 of these subjects will be heterozygous or homozygous for the STAT 4 risk alleles and 10 will not. An additional 10 SLE subjects with variable genotypes will receive placebo twice daily. Both the investigators and study subjects will be blinded to the treatment allocation. The study duration is a maximum of 16 weeks; with 30 days screening period followed by an 8 week treatment period and a 4 week follow-up period. The proposed clinical trial is an exploratory study designed to yield preliminary data about the safety, clinical and biologic efficacy of tofacitinib in SLE subjects as well as information about the influence of genotype on drug efficacy. Provided the

drug is well tolerated in the SLE subjects, the data can be used to design and power a larger Phase II study of clinical efficacy and safety.

5.2 Description of Endpoints:

5.2.1 Primary Endpoint

The primary endpoint is safety of tofacitinib in SLE subjects. In order to assess safety, toxicity is defined as any Grade 3 adverse event or higher (as measured by the National Cancer Institute (NCI), Common Terminology Criteria for Adverse Events (CTCAE), Version 4.0). Grade 3 adverse events will include measures of standard laboratory tests including serum chemistries, urinalysis, complete blood counts and lipid profiles at screening, baseline and conclusion of the treatment period. Included in the lipid profiles will be measurements of lipoproteins such as; proinflammatory HDL and oxidized HDL that has been associated with increased atherosclerotic risk in SLE (68). Secondary Endpoints

- Preliminary assessments of clinical response will be measured by:
  - the change in SLEDAI 2K between baseline and week 8 (end of treatment)
  - the change in the Physicians Global Assessment scores between baseline and week 8 (end of treatment).
- Changes in anti-dsDNA autoantibody titers, complement proteins C3 and C4, markers of systemic inflammation such as ESR and CRP between baseline and Day 56/week 8 (end of treatment).
- Assessment of biologic effect(s) will be measured by changes in the following mechanistic endpoints between baseline and Day 56/ week 8 (end of treatment):
  - Alteration in peripheral blood immune cell populations by phosphoflow cytometry with special attention to NK, CD8+, CD4+, CD25+, Foxp3+ regulatory subsets and Th17 cells.
  - Ligand-induced STAT phosphorylation in circulating T cells and monocytes using phosphoflow assessment of single cells.
  - Expression of STAT-related target genes; expression of pro-inflammatory and immunoregulatory cytokines in T cells and monocytes using RNAseq.
  - Alteration in the “interferon signature” and the “granulocyte signature” in PBMCs using nanostring; measures of CD3+ T cell expression of IFN regulatory factor-related genes using RNAseq and nanostring.

- Alteration in peripheral blood immune cell populations with special attention to a subset of aberrant neutrophils present in lupus patients (low-density granulocytes-LDG)(17). With regards to the latter, assessments will include quantification of LDG levels in peripheral blood and their propensity to form neutrophil extracellular traps (NETs) in the absence of added exogenous stimuli.
- Measures of serum cytokines (IL-2, 4, 6, 7, 9, 15, 12,17, 23, IFNα and IFNγ)
- Changes in vascular function, as assessed by the reactive hyperemia index (RHI) using Endopat device, arterial stiffness using cardio-ankle vascular index (CAVI), and by using SphygmoCor device to determine central blood pressures and arterial stiffness. These are surrogate markers of vascular damage and future atherosclerosis development which are amenable to change within this treatment timeframe.
- Measurements of lipoproteins, including proinflammatory HDL and oxidized LDL that has been associated with increased atherosclerotic risk in SLE (68). Additional studies may be performed to assess modifications in the proteome of HDL, reverse cholesterol transport as well as lipoprotein particles.

- Assessment of the effect of STAT4 risk alleles on drug efficacy will be measured by a comparison of changes in all of the above clinical and biologic measures between Days 1 and 56 between the SLE subjects with the STAT4 risk alleles and those without.
- Assessment of durability of the clinical and biologic effects will be measured by changes in all of the above clinical and biologic measures between Day 56/week 8 (end of treatment) and Day 84/week 12 (end of study).
- Patient reported outcomes for clinical efficacy will be measured by changes in the SF-36, the Multidimensional Assessment of Fatigue questionnaire and the Patient Global Assessment scores between baseline and Day 56/week 8 (end of treatment).

5.3 Recruitment:

Patients may be recruited:

- From the Outpatient Rheumatology Clinic of the Clinical Center at the NIH or the NIAMS Community Health Center;
- From the Lupus Clinics associated with the Feinstein Institute for Medical Research (FIMR), Manhasset, NY;
- From patients referred for treatment and/or second opinion;
- From local area rheumatology and nephrology practices and university hospital clinics;
- By advertising the study to the rheumatologists and nephrologists;
- By direct advertising to patients through publications of patient advocate organizations, such as the Arthritis Foundation, the Lupus Foundation, Lupus Research Institute, Alliance for Lupus Research, and in the news outlets.

5.4 Screening Methods

All potential subjects will have preliminary screening done under the "Studies of the Pathogenesis and Natural History of Systemic Lupus Erythematosus" protocol (94-AR-0066) at the NIH Clinical Research Center. Subjects who are eligible for the study will be asked to sign the informed consent document.

Potentially eligible patients identified in the Lupus Clinics at the FIMR will be approached by the investigators at FIMR to discuss the study. If interested, an appointment will be made at the NIH Clinical Research Center to review and sign the informed consent document. A subject is considered enrolled in the study when a signed informed consent is obtained.

5.5 Study Phases

5.1.1 Pre-Screening and Screening:

Subjects will be pre-screened on the "Natural History of SLE" protocol (94-AR-0066). Those with mild-moderate active lupus and no obvious exclusion criteria will be offered the chance to enroll in the study. Interested subjects will sign the consent form and undergo formal screening. This will take place 30 days of the first treatment. Routine laboratory data obtained within 7 days of the screening

visit can be used for screening. All patients will have their screening visit at the NIH Clinical Research Center.

5.1.2 Treatment Period:

All subjects who fulfill eligibility criteria will be randomized to one of 2 dosing arms; tofacitinib 5 mg twice daily or placebo twice daily for 56 days. Each subject will be seen at the NIH Clinical Research Center Day hospital or Outpatient clinic at the NIH at baseline. After this baseline visit each subject will be seen in the either at the Clinical Research Center Day Hospital or outpatient clinic at the NIH for days 14, 28, 42, 56 and 84 (all visits are +/- 7 days). They will have a physical exam, blood draw and be assessed for adverse events and compliance with medications and study drug at each of these visits (see Appendix 21.1 for the schedule of events). Each subject will receive a telephone call on days 7, 21, 35 and 49(all days are +/-7 days) to assess adverse events and compliance. Day 56 (+/-7 days) will be the end of the treatment period.

5.1.3 Follow-Up Period:

There will be a follow up safety visit at Day 84(+/- 7 days). End of follow up (EFU) end-points will be collected at this visit.

5.6 Duration of Study Participation

Study participation will be a maximum of 16 weeks, including within 30 days screening followed by 8 weeks in the treatment and 4 weeks in follow up phase. All study time periods are inclusive of a window of +/- 7 days to accommodate any potential variability in subject scheduling (see Appendix 21.1; Schedule of Events).

5.7 Clinical Assessments

Clinical assessment will take place in the outpatient clinics or the day hospital of the Clinical Research Center of the NIH. Vascular assessments will be performed in the Vascular Lab at the NIH Clinical Research Center.

Vital Signs Measurement

Vital signs measurements will include pulse rate, systolic and diastolic blood pressure, respiratory rate, and temperature. These measurements will be performed at all study visits.

5.8 Adverse event assessment

Adverse events will be assessed by reviewing the interim medical history, performing a physical evaluation, asking open-end question of how the patient's feeling and reviewing laboratory results at each visit. Adverse events will be collected after the subjects receive their first dose of study medication until their last study visit. Abnormalities detected at the screening visit will be noted as baseline but not considered adverse events.

5.9 Assessment of lupus disease activity

Lupus disease activity will be assessed by completing the following measures:

- SLEDAI 2K (Appendix 22.3): This validated disease activity measure reflects clinical events that occurred within 28 days, and includes physical findings and laboratory measures.
- BILAG 2004
- Physician Global Assessment (PGA) (Appendix 21.4): Global disease activity will be rated by the PGA
- Joint count: A 28tender and swollen joint count (DAS-28) (Appendix 22.5) will be done.
- Cutaneous Lupus Erythematosus Disease Area and Severity Index (CLASI) (Appendix 22.6): The severity of cutaneous involvement (when applicable) will be documented on the CLASI

5.10 Subject questionnaires

The following assessments will be utilized to assess patient reported outcomes:

1. Fatigue will be measured by the Multidimensional Assessment of Fatigue questionnaire (Appendix 21.8).
2. Quality of life by the Short Form 36 (SF36) (Appendix 21.7) questionnaire.
   Both of these tools are validated and widely used in SLE.
3. Subjects will also be asked to indicate overall assessment of disease activity using the Patient Global Assessment Scale.

5.11 Clinical Laboratory Assessments

The CLIA certified clinical laboratories at the NIH Clinical Center (Department of Laboratory Medicine) will be the central laboratories for all routine clinical laboratory parameters including CBC, chemistries, urinalysis, PT/PTT, ESR, CRP, hepatitis B surface antigen, hepatitis C

antibody, HIV antibody, Quantiferon Gold tuberculosis testing, complement components 3 and 4, anti-ds DNA, ANA and anti-ENA (Sm, RNP, SSA, SSB), anticardiolipin antibodies, lupus anticoagulant, and anti-beta2-glycoprotein antibodies. A serum or urine pregnancy test will be performed for female subjects at screening, before dosing at baseline and again on days 56 and 84, unless they are postmenopausal (no menstrual period for 2 years or more), had a previous hysterectomy or removal of the ovaries. Testing for BK virus quantitative PCR serum and urine levels will be performed at Screening, day 28, day 56 and day 84. Subjects with positive serum BK virus PCR results at screening will be excluded from the study. Subjects with positive BK virus in urine with undetectable BK virus in serum will be allowed to participate in the study. However, an increase in BK viureia by one log or development of BK viremia at any of the post randomization visits will lead to study medication discontinuation. Subjects will be asked to complete safety visits and further follow-up will be done under the Natural History Protocol or by their local physicians.

The hematology, chemistry, and urine parameters will be assayed (see Appendix 21.1; Schedule of Events) and are described in detail under Section 4.9 Study Visit Procedures.

5.12 Laboratory Research Studies

Mechanistic studies

Mechanistic studies will be conducted to investigate effects of tofacitinib on intracellular signaling molecules, cytokine expression, circulating immune cell types and autoreactive B cells. Results of these mechanistic studies will be used to compare responses to tofacitinib in those patients with the STAT4 risk alleles to those without STAT4 risk alleles; as well as to the SLE subjects receiving placebo.

All samples for the mechanistic assays will be collected at baseline (prior to treatment), on Day 14, on Day 56 (end of treatment), on Day 84 (end of study) and Unscheduled Visits. These assays will be performed in different laboratories at the NIH and FIMR. To diminish confounding from variability between multiple assays, all of the samples will be treated appropriately, frozen and stored until all samples can be run simultaneously for each assay at the end of the study.

- The following is a list of the planned mechanistic studies:

- Ligand-induced STAT phosphorylation in circulating T cells and monocytes using phosphoflow assessment of single cells;
- Expression of STAT-related target genes; expression of pro-inflammatory and immunoregulatory cytokines in T cells and monocytes using RNA seq;
- Alteration in the "interferon signature" and the "granulocyte signature" in PBMCs using nanostring; measures of CD3+ T cell expression of IFN regulatory factor-related genes using RNAseq and/or NanoString;
- Alteration in peripheral blood immune cell populations with special attention to CD4+, CD25+, Foxp3+ regulatory subsets,Th17 cells, and a subset of aberrant neutrophils present in lupus patients (low-density granulocytes)(69). With regards to the latter, assessments will include quantification of LDG levels in peripheral blood and their propensity to form neutrophil extracellular traps (NETs) in the absence of added exogenous stimuli;
- Measures of serum cytokines (IL-2, 4, 6, 7, 9,12, 15, 17, 23, IFNα and IFNγ);
- Measurement of pro-inflammatory HDL (piHDL) will be identified with a cell free assay based on the ability of HDL to prevent oxidation.
- Changes in vascular function, as assessed by the reactive hyperemia index (RHI) using Endopat device, and arterial stiffness using cardio-ankle vascular index (CAVI). Both are surrogate markers of vascular damage and future atherosclerosis development which are amenable to change within this duration of treatment.
- Additional biomarker studies: Left over whole blood, plasma and serum from the planned mechanistic studies will be frozen and stored for future use.

5.13 Visit Procedures:

5.13.1 ***<u>Pre-screening:</u>***

NIH subjects with SLE who are followed in Protocol 94-AR-0066 will be offered participation in the study if they are found to have mild to moderate disease activity and no obvious exclusion criteria. Pre-screening assessments will be done during routine clinical follow up or initial evaluations and will include review of medical history, physical examination and laboratory

assessment and concomitant medications. All of the subjects referred to this study will be enrolled in protocol 94-AR-0066 first.

Patients followed in the Lupus Clinics associated with the FIMR will be offered participation in the study if they are found to have mild to moderate disease activity and no obvious exclusion criteria. These patients will be referred to the NIH for their initial screening and subsequent study-related visits. A summary of the study procedures is in Appendix 21.1.

5.13.2 Screening Period (—30 days to Day 1):

Subjects may be scheduled for screening visits at the NIH Outpatient 9 Clinic or the 5SWS Day Hospital. After informed consent is provided, subjects will undergo screening assessments that include:

- review of inclusion and exclusion criteria,
- vital sign measurements,
- medical history and physical examination,
- EKG,
- Concomitant medications review.
- Laboratory studies:
- complete blood count with differential,
- serum chemistry panel (creatinine, glucose, urea nitrogen, , total bilirubin, alanine aminotransferase, aspartate aminotransferase, alkaline phosphatase),
- pregnancy test (serum or urine; for females with reproductive potential only),
- urinalysis and random urine protein/creatinine ratio,
- lipid panel
- Lipoprotein Profile

- ANA and anti-ENA (Sm, RNP, SSA, SSB), anticardiolipin antibodies, lupus anticoagulant, and anti-beta2-glycoprotein antibodies,
- screening serologies for, anti-dsDNA antibodies, , C_3, C_4,
- quantitative Immunoglobulins, screening serologies for hepatitis B, hepatitis C and HIV,
- tuberculosis screening using the Quantiferon Gold test.
- BK virus serum and urine quantitative PCR level
- Assessment of lupus activity (SLEDAI 2K,BILAG 2004 , PGA)

After confirmation of eligibility, the Clinical Center Research Pharmacy at the NIH will be notified by the investigator of the subject's eligibility. The NIH Research Pharmacy will be responsible for randomization of all subjects based on their communication with FIMR for the presence or absence of STAT 4 risk allele.

5.13.3 Day 1 +/- 7 days Study Visit:

All subjects will be seen in the Clinical Center outpatient clinic or the Clinical Center Day Hospital at the NIH for their visits.

The following procedures will be performed at the Day 1(+/-7 days) visit:

- review of inclusion and exclusion criteria
- concomitant medication review
- Drug Accountability- Dispensing Study Drug 35 days of supply
- vital sign assessments
- Presenting symptoms, abbreviated medical history and
- physical examination
- laboratory studies
- complete blood count with differential,

- serum chemistry panel (,creatinine, glucose, urea nitrogen, , total bilirubin, alanine aminotransferase, aspartate aminotransferase, alkaline phosphatase),
    - pregnancy test (urine or serum; for females of reproductive potential only),
    - urinalysis, random urine protein/creatinine ratio,
    - lipid panel
    - Lipoprotein Profile
    - Lymphocyte pheno-TBNK
    - inflammatory markers (hs-CRP, ESR),
  - anti-dsDNA anibodies, serum complement components C_3 and C_4,
- quantitative Immunoglobulins,
- Research labs
- assessment of SLE disease activity (SLEDAI 2K, PGA),
- cutaneous Lupus Erythematosus Disease Area and Severity Index (CLASI), ,
- DAS-28
- vascular function studies,
- patient questionnaires (Multidimensionnel fatigue, SF-36, Patient Global Assessment),
- Study drug administration: A 35day supply of study drug will be dispensed to subjects on Day 1 with careful instructions regarding the twice daily dosing regimen and a review of potential side effects. Subjects will take the first dose of study drug in the Clinical Research Center at the NIH. No drug will be dispensed to a female subject of reproductive potential until negative pregnancy test results (serum or urine) have been obtained within 24 hours.

5.13.4 Day 7+/- 7days, Day 21+/- 7days, Day 35 +/- 7days and Day 49 +/- 7days Telephone Calls ±7days :

All subjects will receive a telephone call from the study coordinator on Days 7, 21, 35 and 49 (all +/- 7days). The purpose of the calls will be to ascertain any adverse events, review compliance with study drug and with concomitant medications.

5.13.5 Day 14 +/- 7days Study Visit:

All subjects will return to the Clinical Center at the NIH on Day 14(+/- 7 days)for a brief visit and they will be asked to bring their bottles of study drug with them. The following procedures will be performed at this visit:

- adverse event assessment
- concomitant medication review
- vital sign measurements
- Presenting symptoms abbreviated medical history and
- physical examination
- Research labs
- laboratory studies:
    - complete blood count with differential,
    - serum chemistry panel (creatinine, glucose, urea nitrogen, total bilirubin, alanine aminotransferase, aspartate aminotransferase, alkaline phosphatase),
    - anti-dsDNA antibodies, serum complement components C3 and C4,
    - quantitative Immunoglobulins,
    - pregnancy test (urine or serum; for females of reproductive potential only),
    - urinalysis, random urine protein/creatinine ratio,

- assessment of SLE disease activity:
  - SLEDAI 2K, PGA,
  - Cutaneous Lupus Erythematosus Disease Area and Severity Index (CLASI),
  - DAS-28
  - Patient questionnaires (Multidimensionnel fatigue, SF-36, Patient Global Assessment)

5.13.6 Day 28 +/- 7 days Study Visit:

All subjects will return to the Clinical Center at the NIH on Day 28(+/- 7 days) for a brief visit and they will be asked to bring their bottles of study drug with them. The following procedures will be performed at this visit:

- adverse event assessment
- concomitant medication review
- Drug accountability-Returning used drug bottle
- Drug accountability- Dispensing Study Drug 35 days of supply
- vital sign measurements
- Presenting symptoms abbreviated medical history
- physical examination
- laboratory studies:
  - complete blood count with differential,
  - serum chemistry panel (creatinine, glucose, urea nitrogen, total bilirubin, alanine aminotransferase, aspartate aminotransferase, alkaline phosphatase),

        - pregnancy test (urine or serum; for females of reproductive potential only),
        - urinalysis, random urine protein/creatinine ratio,
        - Anti-dsDNA antibodies, serum complement components C_3 and C_4
        - quantitative Immunoglobulins,
        - BK virus serum and urine quantitative PCR level
- assessment of SLE disease activity:
    - SLEDAI 2K, BILAG 2004, PGA,
    - cutaneous Lupus Erythematosus Disease Area and Severity Index (CLASI),
    - DAS-28
    - patient questionnaires (Multidimensionnel fatigue, SF-36, Patient Global Assessment).

5.13.7 Day 42 +/- 7 days Study Visit:

All subjects will return to the Clinical Center at the NIH on Day 42(+/- 7 days) for a brief visit. The following procedures will be performed at this visit:

- adverse event assessment
- concomitant medication review
- vital sign measurements
- Presenting symptoms abbreviated medical history
- physical examination
- laboratory studies:
    - complete blood count with differential,
    - serum chemistry panel (creatinine, glucose, urea nitrogen, total bilirubin, alanine aminotransferase, aspartate aminotransferase, alkaline phosphatase),

    - pregnancy test (urine or serum; for females of reproductive potential only),
    - urinalysis, random urine protein/creatinine ratio,
    - anti-dsDNA antibodies, serum complement components C3 and C4,
    - quantitative Immunoglobulins,

- assessment of SLE disease activity:
  - SLEDAI 2K, PGA,
  - cutaneous Lupus Erythematosus Disease Area and Severity Index (CLASI),
  - DAS-28
  - patient questionnaires (Multidimensionnel fatigue, SF-36, Patient Global Assessment)

5.13.8 Day 56 +/- 7 days Study Visit (end of treatment period):

All subjects will be seen again in the Clinical Center Day hospital or outpatient clinic at the NIH for this visit and they will be asked to bring their bottles of study drug with them. Subjects will be asked to fast after midnight the night previously for the fasting lipid profile and the following procedures will be performed at this visit:

- adverse event assessment
- concomitant medication review
- Drug accountability-Returning used drug bottle
- vital sign measurements
- Presenting symptoms abbreviated medical history
- physical examination
- laboratory studies:
    - complete blood count with differential,

    - serum chemistry panel (creatinine, glucose, urea nitrogen, , total bilirubin, alanine aminotransferase, aspartate aminotransferase, alkaline phosphatase),
    - pregnancy test (urine or serum; for females of reproductive potential only),
    - urinalysis, random urine protein/creatinine ratio,
    - inflammatory markers (hsCRP, ESR),
    - Anti-dsDNA antibodies, serum complement components C_3 and C_4,
    - quantitative Immunoglobulins,
    - lipid panel,
    - Lipoprotein Profile
    - Lymphocyte pheno-TBNK
    - BK virus serum and urine quantitative PCR level

- EKG,
- Research labs,
- assessment of SLE disease activity
    - SLEDAI 2K, BILAG 2004, PGA,
    - cutaneous Lupus Erythematosus Disease Area and Severity Index (CLASI),
    - DAS-28
    - Patient questionnaires (Multidimensionnel fatigue, SF-36, Patient Global Assessment)
- Vascular function studies.

5.13.9 Day 84 +/- 7 days (follow up visit, end of study):

All subjects will return to the Clinical Center outpatient clinic or Day hospital at the NIH for the Day 84(+/- 7 days) visit that will serve as the final visit to evaluate for any toxicity and/or

increased disease activity after discontinuation of study drug. Subjects will be asked to fast after midnight the previous night for the fasting lipid profile and the following procedures will be performed at this visit:

- adverse event assessment
- concomitant medication review
- vital sign measurements
- presenting symptoms abbreviated medical history
- physical examination
- laboratory studies:
    - complete blood count with differential,
    - serum chemistry panel (creatinine, glucose, urea nitrogen, total bilirubin, alanine aminotransferase, aspartate aminotransferase, alkaline phosphatase),
    - pregnancy test (urine or serum; for females of reproductive potential only),
    - urinalysis, random urine protein/creatinine ratio,
    - inflammatory markers (hsCRP, ESR),
    - Anti-dsDNA antibodies, serum complement components C_3 and C_4,
    - quantitative Immunoglobulins,
    - lipid panel,
    - Lipoprotein Profile
    - Lymphocyte pheno-TBNK
    - BK virus serum and urine quantitative PCR level

- EKG,
- research labs,
- assessment of SLE disease activity
    - SLEDAI 2K, BILAG 2004, PGA,
    - cutaneous Lupus Erythematosus Disease Area and Severity Index (CLASI),
    - DAS-28
    - patient questionnaires (Multidimensionnel fatigue, SF-36, Patient Global Assessment).
- Vascular function studies.

5.13.10 Unscheduled Visits:

In the event of an adverse event or other reason that results in an unscheduled visit, reasonable measures should be taken to try and obtain the following information:

- adverse event assessment (AE)
- concomitant medication review
- vital sign measurements
- Presenting symptoms abbreviated medical history
- physical examination
- laboratory studies:
    - complete blood count with differential,
    - serum chemistry panel (creatinine, glucose, urea nitrogen, total bilirubin, alanine aminotransferase, aspartate aminotransferase, alkaline phosphatase),
    - pregnancy test (urine or serum; for females of reproductive potential only),

    - urinalysis, random urine protein/creatinine ratio,
    - inflammatory markers (hsCRP, ESR),
    - Anti-dsDNA antibodies, serum complement components C_3 and C_4,
    - quantitative Immunoglobulins,
    - lipid panel,
    - Lipoprotein Profile
    - Lymphocyte pheno-TBNK
- EKG,
- research labs,
- assessment of SLE disease activity
    - SLEDAI 2K, BILAG 2004, PGA,
    - cutaneous Lupus Erythematosus Disease Area and Severity Index (CLASI),
    - DAS-28
    - patient questionnaires (Multidimensionnel fatigue, SF-36, Patient Global Assessment).

5.14 Follow-up/Termination Procedures:

During the study, subjects will continue to receive regular medical care from their primary physicians. Any AEs reported will be followed by the investigators as described in Section 16.1.8. Following completion of the study, subjects will return to the care of their referring physicians. Subjects who received treatment under protocol 94-AR-0066 may continue to be managed under that protocol. A summary of the subjects' course in the study will be sent to the referring physician, if applicable.

Subjects who withdraw or are withdrawn from the study after receiving study treatment will be asked to complete the Day 56 (end of treatment) and the Day 84 follow-up assessments.

Subjects who withdraw or are withdrawn before Day 1 will resume care with their referring physician with no further evaluation in this protocol.

5.15 Study Drug, Dosing and Administration:

5.15.1 Tofacitinib:

Tofacitinib (XELJANZ®; Pfizer) is a Janus kinase (JAK) inhibitor. JAKs are intracellular enzymes that transmit signals arising from cytokine or growth factor-receptor interactions on immune cell membranes to influence cellular processes of hematopoiesis and immune cell function. Within the signaling pathway, JAKs phosphorylate and activate Signal Transducers and Activators of Transcription (STATs) which modulate intracellular activity including gene expression. Tofacitinib modulates the signaling pathway at the point of JAKs, preventing the phosphorylation and activation of STATs. XELJANZ® is the citrate salt of tofacitinib. Tofacitinib citrate is a white to off-white powder with the following chemical name: (3R,4R)-4-methyl-3-(methyl-7H-pyrrolo [2,3-d]pyrimidin-4-ylamino)-β-oxo-1-piperidinepropanenitrile, 2-hydroxy-1,2,3-propanetricarboxylate (1:1) It is freely soluble in water and has a molecular weight of 504.5 Daltons. XELJANZ® is supplied for oral administration as 5 mg tofacitinib (equivalent to 8 mg tofacitinib citrate) white round, immediate-release film-coated tablet. Each tablet of XELJANZ® contains the appropriate amount of XELJANZ ®as a citrate salt and the following inactive ingredients: microcrystalline cellulose, lactose monohydrate, croscarmellose sodium, magnesium stearate, HPMC 2910/Hypromellose 6cP, titanium dioxide, macrogol/PEG3350, and triacetin.

5.15.2 Tofacitinib dosing:

The package insert with the recommended dosing is attached as Appendix 22.10. Each subject will be dosed with 5 mg (1 tablet) twice daily for 56 days as is recommended for the treatment of rheumatoid arthritis.

5.15.3 Placebo:

Placebo tablets will be supplied by the NIH Clinical Center Research Pharmacy.

5.16 Treatment Compliance:

Compliance with study drug dosing will be assessed at the Day 28, 56(+/- 7 days) visits. Subjects will be asked to bring their bottles for a pill count that will be recorded. Subjects who have demonstrated less than 80% compliance (missed more than 6 total days of treatment) will be

withdrawn from the study. Subjects withdrawn from the study after receiving study treatment will be asked to follow up to complete the Day 56 (+/-7 days) and Day 84(+/-7 days) follow-up assessments.

5.17 Treatment Allocation:

5.17.1 Genotyping and Blinding:

Most of the SLE patients followed in the Lupus Clinics at the FIMR have agreed to participate in the “Rheumatology Clinical Database and Specimen Bank” protocol at the FIMR. As part of this protocol, blood samples have been collected and stored for DNA analysis. Similarly, NIH subjects followed in the "Studies of the Pathogenesis and Natural History of Systemic Lupus Erythematosus" protocol (94-AR-0066) have also provided consent for DNA analysis. These blood samples will be analyzed for STAT4 risk alleles in Dr. Peter Gregersen’s laboratory at the FIMR. The results of presence or absence of STAT 4 risk allele will be communicated to the NIH Clinical Center Research Pharmacy. As subjects who are followed in these observational cohorts experience disease flares and become eligible for participation in this study, investigators will contact NIH Clinical Center Research Pharmacy during the screening process to determine eligibility based on STAT4 risk alleles. NIH Clinical Center Research Pharmacy will ensure that a minimum of 10 eligible subjects are either heterozygous or homozygous for the STAT4 risk allele and that a minimum of 10 eligible subjects do not have the risk allele. The unblinded coordinator at FIMR will communicate with the NIH Clinical Center Research Pharmacist prior to randomization so that 10 STAT4 allele positive and 10 STAT4 allele negative subjects receive tofacitinib. The placebo group may or may not have STAT4 risk alleles. This will help to maintain the blind for the investigators and study subjects.

5.17.2 Randomization and Blinding:

Randomization will be done by the NIH Clinical Research Center Pharmacy. This is a two arm study and subjects will be randomized to either tofacitinib or placebo in a 2:1 ratio so that 10 subjects with STAT 4 risk alleles and 10 subjects without STAT 4 risk alleles are in the tofacitinib treatment group. The genotype for the placebo group (n=10) is not pertinent to the randomization as these subjects may or may not have the risk alleles. This randomization will necessitate communication between the unblinded coordinator at the FIMR who holds the genotype information and the NIH Clinical Center Research Pharmacist responsible for

randomization. Both subjects and investigators will be blinded to treatment allocation during the first phase of the study. Unblinding will occur for the analyses after the last subject has completed Day 84 of the study. We will inform the subjects of their treatment allocation after the last subject has completed Day 84 of the study.

The blind will be held by the NIH Clinical Center Research Pharmacy. In cases where breaking the blind is necessary to provide clinical care to the patient (as determined by the physician involved in patient's care and principal investigator), the principal investigator will contact the NIH Clinical Center Research Pharmacy and provide the treatment allocation information to the treating physician. Such subject/s would be withdrawn from the study and followed up as described in section 4.10 above. The Data and Safety Monitoring Committee (DSMC) may also request unblinding of treatment allocation or group assignment. These requests will be transmitted to the NIH Clinical Center Research Pharmacy by the principal investigator. The data will be provided directly to the Chair of DSMC without unblinding the investigators before the DSMC determines a plan of action.

5.18 Concomitant medications:

5.18.1 Restricted medications:

The following medications are allowed provided that they are administered at stable doses during the study:

- Antimalarial (hydroxychloroquine 400 mg/day or ≤6.5 mg/kg/day, if more than 400 mg/day); must already be on this stable dose of this medication 12 weeks prior to study entry.
- Prednisone or equivalent steroid dose up to 20 mg /day must already be on this stable dose of this medication 4 weeks prior to study entry.
- ACE inhibitors or ARB medications, must already be on this stable dose of this medication 4 weeks prior to study entry.
- Lipid lowering medications if initiated at least 6 months prior to the screening visit. The dose of any of these medications can be decreased (temporally or for the duration of the study) if clinically indicated for toxicity.

5.18.2 Disallowed medications:

The medications and vaccines listed below will not be allowed during treatment. Any clinical indication requiring treatment with medications listed below will lead to cessation of study treatment. Withdrawn subjects will be asked to return according to the follow up phase of the study and to complete all study procedures.

- Rituximab.
- Any other biologic agent including TNF-α blockers, IL-1 blocking agents, anti-IL-6 agents, belimumab, abatacept.
- Cyclophosphamide.
- Azathioprine.
- Mycophenolate mofetil.
- Rapamycin.
- Cyclosporine.
- Tacrolimus.
- Methotrexate.
- Administration of any live virus vaccines.

5.18.3 Corticosteroids:

Subjects may be on prednisone ≤ 20 mg/day at study entry. Prednisone may be increased as indicated after the end of the treatment period on Day 56(+/- 7 days). At the investigator's discretion, glucocorticoids may be tapered during the study.

5.19 Treatment of flares:

Subjects entering the study will have mild to moderate disease activity. Based on the known response to tofacitinib in RA, animal studies and in cellular assays, we expect a similar rapid response in SLE subjects. Nonetheless, flares that escalate despite treatment with tofacitinib to merit treatment with corticosteroids, increasing current dose of corticosteroid, and/or the addition of another DMARD will meet criteria for subject termination from the study.

5.20 Withdrawal criteria:

Subjects will be informed that they may withdraw or be excluded from the study at any time. The following conditions will require the discontinuation of study drug:

1. Request by subject to be withdrawn from the study.
2. Flares not responding to the treatment above or if, in the opinion of the responsible Investigator, the subject needs immediate immunosuppressive therapy that is not allowed in the protocol.
3. Any Grade 4 adverse event that is unexpected and at least possibly related to study drug.
4. More than 2 weeks delay in treatment after the screening visit.
5. More than 1 "no-show" for a visit.
6. Persistent elevation of LFTs of > 2 times upper limit of normal persistently present on repeated samples 2 weeks +/- 7 days apart.
7. >30% increase in serum creatinine from baseline at enrollment, persistently present on repeated samples 2 weeks +/- 7 days apart.
8. Increase in proteinuria at the time of screening visit to protein to creatinine ratio of more than 1000 mg/mg or more than 1000 mg in a 24 hours urine collection, persistently present on repeated samples 2 weeks +/- 7 days apart.
9. Cellular casts ≥ 2+ persistently present on repeated samples 2 weeks +/- 7 days apart, (Criteria #7,8,and 9 were chosen as withdrawal criteria as they would be indicative of active lupus nephritis requiring aggressive immunosuppressive therapy).
10. Any other reason that, in the opinion of the responsible Investigator, poses unacceptable risk to the subject.
11. Infections requiring intravenous antibiotic treatment.
12. Development of any malignancy.
13. More than one log increase in urine BK virus level by quantitative PCR or detection of BK virus in serum by quantitative PCR.

6 Risks and Discomforts:

6.1 Unexpected Adverse Events:

Unforeseen adverse effects are a risk with every new drug treatment, including new indications for approved drugs. The risks of participating in this study are reasonable relative to the potential health benefits and generalizable medical knowledge that may be obtained.

6.2 Risks/discomfort associated with tofacitinib:

Tofacitinib is approved by the Food and Drug Administration (FDA) for treatment of rheumatoid arthritis. It is also being tested for use in other diseases such as psoriasis, inflammatory bowel disease and to prevent transplant rejection. Though generally well-tolerated in patients with Rheumatoid Arthritis, tofacitinib has potential risks associated with its use. The data on adverse events associated with exposure to tofacitinib are gathered from two Phase II and 5 Phase III multi-center, double-blind, placebo controlled clinical trials where subjects were randomized to tofacitinib 5 mg twice daily, 10 mg twice daily or placebo, with or without concomitant disease modifying drugs (DMARDS); most commonly methotrexate. Adverse events associated with tofacitinib included the following:

6.2.1 Infections

In the rheumatoid arthritis (RA) studies, the overall frequency of infections was 20% in the patients who received tofacitinib 5 mg twice daily and 18% in the patients who received placebo. The most commonly reported infections were upper respiratory infections, throat infections and urinary tract infections.

The most serious side effects reported with tofacitinib in RA patients were serious infections due to bacterial, viral or fungal organisms. These occurred in 11 out of 2685 patients (0.4%) who received the drug for 3 months or less. For patients who experienced 0-12 months of exposure to tofacitinib, serious infections were reported in 34 patients on the 5 mg twice daily dose and in 33 subjects on the 10 mg twice daily dose. The most common serious infections were pneumonia, cellulitis, herpes zoster, and urinary tract infections. Tuberculosis was not reported in patients on any dose of tofacitinib for less than 3 months. During 0-12 months of exposure to tofacitinib, tuberculosis cases were reported in 0 patients receiving the 5 mg twice daily dose and 6 patients who received the 10 mg twice daily dose. Opportunistic infections were not reported in patients

receiving any dose of tofacitinib for less than 3 months. During 0-12 months of exposure, opportunistic infections were reported in 4 patients receiving 5 mg twice daily dosing and 4 patients receiving 10 mg twice daily dosing; the median range for tofacitinib exposure prior to diagnosis of an opportunistic infection was 8 months. These opportunistic infections included cryptococcus, pneumocystis, herpes virus, cytomegalovirus (CMV) and BK virus and most occurred in patients that were also taking corticosteroids and methotrexate.

6.2.2 Gastrointestinal Perforation

Rare events of gastrointestinal perforation have been reported in clinical studies of tofacitinib in RA patients although the role of JAK inhibition in these events is not known.

6.2.3 Malignancy

Malignancies were observed in clinical studies of tofacitinib. In the seven controlled RA clinical studies, 11 solid cancers and one lymphoma were diagnosed in 3328 patients receiving tofacitinib with or without methotrexate, compared to 0 solid cancers and 0 lymphomas in 809 patients in the placebo with or without methotrexate group during the first 12 months of exposure. Lymphomas and solid cancers have also been observed in the long-term extension studies in RA patients treated with tofacitinib. Two of these 11 malignancies occurred in the first 3 months of exposure to tofacitinib. The most common types of malignancies were lung and breast cancer followed by gastric, colorectal, renal cell, prostate cancer and malignant melanoma. In Phase 2B, controlled dose-ranging trials in de-novo renal transplant patients, all of whom received induction therapy with basiliximab, high dose corticosteroids, and mycophenolic acid products, Epstein Barr virus-associated post-transplant lymphoproliferative disorder was observed in 5 out of 218 patients treated with tofacitinib (2.3%) compared to 0 out of 111 patients treated with cyclosporine.

6.2.4 Laboratory Abnormalities

6.2.4.1 **Lymphopenia:** Lymphopenia below 500 cells/mm^3 occurred in0.04 % of patients in both dosing groups of tofacitinib within the first 3 months. Low lymphocyte counts (<500 cells/mm^3) were associated with increased risk for serious infections.

6.2.4.2 Neutropenia: Neutropenia with an ANC less than 1000 cells/mm^3 occurred in 0.07% of patients in both dosing groups of tofacitinib during the first 3 months of exposure. No ANCs less than 500 cells/mm3 were reported and there was no clear association between neutropenia and risk of infection. The neutropenia was dose related and reversible.

6.2.4.3 Anemia: Although JAK inhibition has potential to affect hematopoiesis through interruption of cytokine-induced regulation of hematopoiesis, minimal effects of tofacitinib 5 mg twice daily have been observed in the clinical trials in RA. Mild fluctuations in hemoglobin were not different from those observed in the placebo group.

6.2.4.4 Liver Function Tests: Increased liver enzyme tests greater than 3 times the upper limit of normal were seen in approximately 1.2% of patients overall. No differences in the incidence of AST or ALT elevations were observed between the placebo, 5 mg and 10 mg twice daily groups in the first three months.

6.2.4.5 Lipids: Dose related elevations of lipid parameters were observed at one month of exposure and remained stable thereafter. These were:

- a mean increase of LDL by 15% in the 5 mg twice daily arm and 19% in the 10 mg twice daily arm, and
- a mean increase in HDL by 10 % in the 5 mg twice daily arm and 12% in the 10 mg twice daily arm.

However, mean LDL/HDL ratios were unchanged in patients treated with tofacitinib.

6.2.4.6 Serum Creatinine: In a pooled analysis of 5 phase 3 and 2 long term extension studies of tofacitinib in subjects with RA increase in serum creatinine were seen predominantly in the first 3 months of treatment. The serum creatinine increases at Month 3 were 0.07 and 0.08 mg/dl for 5 and 10 mg BID tofacitinib doses, respectively, compared with 0.04 mg/dl in the placebo. In the tofacitinib 5 mg BID group, 17/1,220 (1.4%) patients had a confirmed

serum creatinine increase of ≥33% from baseline in Months 0 to 3. Of these patients only 2 patients had serum creatinine elevation above the reference range for normal. In the tofacitinib 10 mg BID group, 23/1,217 (1.9%) patients had a confirmed serum creatinine increase ≥33% from baseline in Months 0 to 3. Of these 23 patients only 4 had serum creatinine above the reference range for normal. No continued worsening of serum creatinine was noted during the long term follow-up studies. The published data revealed that these changes plateaued or reversed in long term follow up.

6.2.5 Other adverse reactions:

Other adverse reactions reported with tofacitinib at doses of 5 mg twice a day include diarrhea (4%), nasopharyngitis 3.8%), upper respiratory infections (4.5%, headache (4.3%), high blood pressure (1.6%). None of these occurred significantly more frequently than in the patients who were treated with placebo.

6.2.6 Medication interactions

Tofacitinib exposure is increased when tofacitinib is co-administered with moderate and potent inhibitors of cytochrome P450 (CYP) 3A4 (e.g., ketoconazole) or CYP2C19 (e.g., fluconazole). Potent CYP3A4 inducers (e.g., rifampin) will decrease exposure to tofacitinib.
The risk of increased immunosuppression is enhanced if tofacitinib is co-administered with potent immunosuppressive drugs such as azathioprine, tacrolimus, cyclosporine.

6.3 Flare of SLE:

There are no data about the use of tofacitinib in patients with SLE. Tofacitinib may be ineffective or may even worsen lupus. The potential impact of flares is minimized in this study by close monitoring of subjects and strict withdrawal criteria for flares.

6.4 Risks associated with study procedures:

6.4.1 Blood draws

Subjects may experience discomfort, bleeding, or bruising at the venipuncture site, which should resolve with time. There is also a small risk of fainting. Infection at the site of needle insertion may also occur but is rare with the use of sterile disposable needles and aseptic technique.

The amount of blood drawn for clinical care indication and research purposes will be kept within the NIH guidelines of 10.5 mL/kg or 550 mL, whichever is smaller, over any 8 week period for adults.

6.4.2 Electrocardiogram (ECG)

There is no clinically significant risk associated with this procedure. There may be minor discomfort, when the ECG electrodes taped to chest are removed. Rarely, a reaction to the electrodes may cause redness or swelling of the skin.

6.4.3 Vascular Function Studies

Explanation of procedures and risks:

6.4.3.1 Pulse wave analysis (SphygmoCor):

SphygmoCor is a set of non-invasive tools used to determine central blood pressures and arterial stiffness. It (1) derives the pressure wave from the ascending aorta to the carotid artery and (2) gives an accurate measurement of pressure at the heart, brain, and kidneys. However, it cannot be used on patients who may suffer from heart arrhythmias or arterial stenosis. The SphygmoCor consists of the following:

SphygmoCor Px Pulse Wave Analysis System – a diagnostic tool to measure central blood pressure

SphygmoCor Pulse Wave Analysis System – an algorithm used to determine central aortic pressure and visualize ventricular- vascular interactions

SphygmoCor Pulse Wave Velocity System- a tool that derives a pressure pulse waveform using the pressure tonometer and an ECG signal simultaneously. Arterial

tonometry uses a pressure sensor to detect the speed of a pulse wave and may indicate a problem in the arteries.

SphygmoCor Pulse Wave Monitoring System– a tool that provides an estimated pressure waveform from the ascending aorta.

6.4.3.2 **Cardio-ankle vascular index (CAVI):**

For this procedure, placement of ECG electrodes on both wrists and a microphone for phonocardiograph on second intercostal space and 4 blood pressure cuffs wrapped around 4 extremities. Arterial stiffness is calculated followed specified formulas. Main advantage of this procedure versus other procedures to measure arterial stiffness is that it is not altered by blood pressure. In addition, it is simple to perform. All vasodilators, antihypertensives and statins will be held the morning of the test and will be restarted after the procedures are completed. This is in order to avoid having additional variables interfering with the readings of the vascular measurements. The test takes approximately 20 minutes to be performed.

6.4.3.3 **Peripheral arterial tonometry (Endopat):**

EndoPAT™ quantifies the endothelium-mediated changes in vascular tone, elicited by a 5-minute occlusion of the brachial artery (using a standard blood pressure cuff). When the cuff is released, the surge of blood flow causes an endothelium-dependent. Flow Mediated Dilatation (FMD). The dilatation, manifested as Reactive Hyperemia, is captured by EndoPAT as an increase in the PAT Signal amplitude. A post-occlusion to pre-occlusion ratio is calculated by the EndoPAT software, providing the EndoPAT index. The test takes approximately 15 minutes to complete, is very easy to perform, and is both operator and interpreter independent. It is a noninvasive test, providing automatic analysis, office-based procedure. The five-minute blood pressure cuff inflation is an accepted standard test to cause reactive hyperemia (the

increase of blood flow after a temporary restriction in blood supply) for the assessment of endothelial function. While the occlusion may cause some minor discomfort and tingling in the fingers, the test is absolutely harmless. It is recommended that the patient fast 3 to 8 hours before the test. Subjects will be requested to hold all medications including but not limited to all vasodilator, antihypertensive and statin on the morning of the test. Subjects will be requested to bring their medication with them on the day of the procedure they will resume their medications right after the completion of procedures. This is in order to avoid any possible confounding variables interfering with the readings of the vascular measurements.

Vascular function studies risk (SphygmoCor, CAVI and Endopat): These procedures are very well tolerated. Other than potential transient minimal discomfort with blood pressure cuff, no side effects are expected.

7 Safety Monitoring (see section 15) :

All subjects will be followed from the time of informed consent until the end of study. At each study visit, subjects will be evaluated for AEs and will be followed until resolution or stabilization of any AEs. All AEs will be graded according to a descriptive severity scale based on the National Cancer Institute Common Toxicity Criteria Version 4.0 (Appendix 21.15). Abnormal findings at the screening visit will be recorded but not considered as adverse events. Concomitant medications will be recorded at the screening visit and assessed at each subsequent visit and telephone monitoring calls.

8 OUTCOME MEASURES:

- Ligand-induced STAT phosphorylation in circulating T cells and monocytes using phosphoflow assessment of single cells measured at baseline, study days 56 and 84.
- Reduced expression of STAT-related target genes; expression of pro-inflammatory and immunoregulatory cytokines in PBMCs, T cells, and monocytes using RNAseq and/or NanoString measured at baseline, study days 7, 56 and 84.
- Alteration in the “interferon signature”; measures of CD3+ T cell expression of IFN regulatory factor-related genes using RNAseq and NanoString between subjects with

STAT4 risk allele present and subjects with STAT4 risk allele absent measured at baseline, study days 56 and 84.

- Flow cytometry immunophenotyping to analyze alteration in peripheral blood immune cell populations with special attention to CD4+, CD25+, Foxp3+ regulatory subsets and Th17 cells between subjects with STAT4 risk allele present and subjects with STAT4 risk allele absent at baseline, study days 56 and 84.
- Measures of serum cytokines (IL-2, 4, 6, 7, 9, 12, 15,17, 23, IFNα and IFNγ) at baseline, study days 56 and 84.
- Reduced IgG autoantibody levels: a statistically significant difference in the change in IgG autoantibody (ANA, anti-dsDNA, anti-Ro, anti-La, anti-Sm, anti-RNP and anticardiolipin antibodies levels) between the subjects with STAT4 risk allele present and subjects with STAT4 risk allele absent at study days 56 and 84.
- Clinical Efficacy: the difference in scores on patient reported outcome measures (SF-36, Fatigue scale and Patient Global Assessment) between subjects on study medications vs. subjects receiving placebo at study days 56 (8 weeks) and 84 (12 weeks).
- Clinical response will be defined as:
    - An improvement in SLEDAI 2K scores of 4 or more from baseline without a worsening (defined as an increase > 0.3) in PGA (physician's global assessment).
- SLE disease flares will be defined as:
    - Mild to moderate flare:
        - an increase in SLEDAI 2K score ≥ 3 but the total score is < 12 or
        - an increase in the PGA > 1 but the total score is < 2.5
    - Severe Flare:
        - a SLEDAI 2K score > 12
        - a PGA>2.5

9 STATISTICAL ANALYSIS

9.1 Analysis Populations:

9.1.1 Safety Population:

The Safety Population will consist of all enrolled subjects receiving at least one dose of study treatment.

9.1.2 Efficacy Population:

The Efficacy Population (EP) will consist of subjects who receive at least 3 doses of study treatment.

9.2 Data Analysis:

Demographic and Baseline Characteristics

Demographic and baseline characteristics will be summarized in tables. Continuous demographic and baseline variables will be summarized as means, medians, standard deviations, minimum values, and maximum values. Categorical demographic (e.g., race) and baseline variables will be summarized as frequencies and percentages.

9.3 Primary outcome:

The primary outcome is to evaluate the safety and tolerance of tofacitinib in patients with SLE. This analysis will include a comparison of rates of adverse events (serious adverse events, Grade 3 and 4 toxicities not fulfilling the criteria for SAE, and non-serious adverse events) and rates of SLE disease flares between the tofacitinib group and the placebo group.

9.4 Secondary outcomes:

Secondary outcomes will be analyzed by comparisons between the treatment (all SLE subjects receiving tofacitinib) and placebo groups and by comparisons between the STAT4 + and STAT4 – groups once the last subject completes the 84 days of study medication.

- Reduced expression of STAT-related target genes; expression of pro-inflammatory and immunoregulatory cytokines in T cells and monocytes using microarray analysis.
- A change in clinical efficacy will be analyzed using the chi-square test comparing the proportions of subjects in the treatment/placebo and STAT4+/STAT4- groups that achieve clinical response at Week 12.

- Differences between the treatment/placebo groups and the STAT4+/STAT4- groups in changes in individual disease activity measures (SLEDAI 2K, PGA) at the end of week 12 will be analyzed as repeated measures with change from baseline as the dependent variable.

9.5 Exploratory Analyses:

Data generated from the exploratory mechanistic studies may not have sufficient power for significance, given the limited number of subjects in this study. Nonetheless, the data generated from differential expression of STAT4 regulated genes in the subjects with STAT4 risk allele present and STAT4 risk allele absent as well as interferon signature in the two groups will be significant. This approach is likely to be hypothesis generating and serves to seed future studies toward a mechanistic understanding of SLE and the effect of JAK inhibition treatment on the dysregulation of the immune system seen in people with SLE.

9.6 Criteria for Significance:

The number of subjects in this study is unlikely to lead to a statistically significant difference in adverse events between subjects on study medication vs. placebo. Therefore, no formal statistical analysis will be performed for the primary outcome.

Hypothesis tests and confidence intervals of secondary analyses will be either 1- or 2-sided. Results will be considered significant at the $\alpha = 0.05$ level.

9.7 Power Analysis:

This is a pilot, Phase Ib study intended to study predominantly the tolerability/toxicity of tofacitinib. The number of subjects for this study is arbitrary and is based primarily on the conventional numbers in Phase I studies and our experience with similar studies in the past. The primary endpoint is safety and tolerability and 15-20 subjects are commonly used as the sample size for open label safety studies. Our plan to have 20 subjects dosed with tofacitinib to evaluate for safety and a placebo group of additional 10 subjects for comparison is consistent with Phase 1 studies.

9.8 Interim Analysis:

No interim analysis will be performed.

9.9 Accrual Number Request:

We plan to treat 30 subjects with at least 112 doses of study medication each (56 days). Subjects withdrawn before receiving 38 doses of study drug (19 days) for reasons other than drug related adverse events or lack of efficacy will be replaced. Assuming a 25% attrition rate we would like to accrue up to 38 subjects.

10 HUMAN SUBJECTS PROTECTION

10.1.1 Ethics and Good Clinical Practice, Data Collection and Data Quality Assurance:

The study will be conducted according to Good Clinical Practice (GCP) guidelines, the Manual of Procedures (MOP), U.S. 21 CFR Part 50 – Protection of Human Subjects, 21CFR312 subpart D and Part 56 – Institutional Review Boards.

10.1.2 Compliance with Good Clinical Practices:

This trial will be conducted in compliance with the protocol, current GCPs recommended by the International Conference on Harmonization (ICH) and the applicable regulatory requirements for participating institutions. These include the tenets of the Declaration of Helsinki and review and approval by the appropriate ethics review committee or IRBs of participating organizations.

10.2 Data Collection:

Study staff will complete electronic case report forms (eCRFs) that will be compiled and stored in a computerized central database Clinical Trial Data Base (CTDB). Subject electronic medical records in the Clinical Research Information System (CRIS) at the NIH will be used as source documents for these eCRFs. These source documents will be made available upon request for review during site monitoring visits. The eCRF data will be validated via a series of manual edit checks, and all relevant data queries will be raised and resolved on an ongoing basis. Complete, clean data will be locked to prevent further inadvertent modifications. All discrepancies will be reviewed and any resulting queries will be resolved with the investigators and amended in the database. All elements of data entry (i.e., time, date, verbatim text, and the person performing the

data entry) will be recorded in an electronic audit trail to allow all data changes in the database to be monitored and maintained in accordance with federal regulations. Security of the database system is maintained through an application firewall, military grade encryption and SSL certificates, with removal of personal identifiers consistent with HIPAA requirements (45 C.F.R. 164.514(a),(b)&(c)).

10.3 Storage of Data and Samples:

10.3.1 Samples:

Research samples collected from subjects consenting to this protocol will be stored in locked secure freezers belonging to NIAMS. The freezers are located in Building 10 at the NIH. The freezers for DNA samples in Dr. Gregersen's lab are located on the 2nd floor of the FIMR and freezers for the Clinical Research Center at the FIMR are located on the first floor. Only study investigators and participating research personnel will have access to the samples. Samples will be kept indefinitely unless there is a significant justification for destroying them. The Principal Investigator will report the loss or destruction of samples collected under this protocol to the Institutional Review Board (IRB).

All samples will be coded and will not have personal identifiers. The codes for identifiers will be contained in a secure electronic database (CTDB) and a subject code log that is maintained in secure research files. An electronic record log with identifiers of all collected research specimens will be kept. These will be stored in secure NIH computers.

Coded samples may be shared with collaborators within and outside the NIH and FIMR. Any remaining samples will be stored in the NIAMS and FIMR locked freezers. Stored samples may be used for studies related to systemic lupus erythematosus. Approval from the IRB will be obtained prior to any research use of stored samples beyond the scope of this study.

All patient samples will be coded and used for research purposes without sharing identifying information and all collaborators will follow federal rules for clinical research.

10.3.2 Data:

Research records and all source documents will be kept in locked cabinets or rooms, and computer research databases will be stored in a secure, password-protected environment, per standard NIH policies. Only study investigators and participating research personnel will have access to the data. The studies done at FIMR will be done on coded samples with no identifiable patient information. Results of the tests will be transmitted to NIH and any data analysis on de-identified samples will be done only at the NIH.

If applicable:

In the future, other investigators (at the NIH) may wish to study these samples and/or data. In that case, IRB approval must be sought prior to any sharing of samples. Any clinical information shared about the sample without patient identifiers would similarly require prior IRB approval.

If applicable:

The research use of stored, unlinked or unidentified samples may be exempt from the need for prospective IRB review and approval. Exemption requests will be submitted in writing to the NIH Office of Human Subjects Research, which is authorized to determine whether a research activity is exempt.

If applicable:

At the completion of the protocol (termination), samples will be retained, and after IRB approval, may be transferred to another existing protocol or a repository.

10.3.3 Record Retention:

The investigators will retain all study-related records for at least 3 years after discontinuation of the study. Some of the research data might be maintained indefinitely for research purposes.

10.3.4 Access to Source Data and Documents:

Medical and research records will be maintained in the strictest confidence. However, as a part of the quality assurance and legal responsibilities of an investigation, the Principal Investigator must permit authorized representatives of the IND sponsor (NIAMS), Leidos Biomedical Research, Inc., CTDB at the NIH, and health authorities to examine (and when required by

applicable law, to copy) clinical records for the purposes of quality assurance reviews, audits, and evaluation of the study safety and progress. Unless required by the laws permitting copying of records, only the coded identity associated with documents or other subject data may be copied (obscuring any personally identifying information). Authorized representatives as noted above are bound to maintain the strict confidentiality of medical and research information that may be linked to identifiable individuals. Participating sites will normally be notified in advance of auditing visits.

All subject records and study documentation will be maintained after the protocol is completed. This will include all documentation of AEs, records of study drug receipt and dispensation, and all IRB correspondence. All study records will be kept for at least 3 years after the investigation is completed.

10.3.5 Data Collection, Quality Control and Quality Assurance Monitoring:

The site investigators are required to keep accurate records to ensure the conduct of the study is fully documented. The period of record retention should be consistent with the record retention policies of the sponsoring agency or applicable regulatory agencies.

The site investigators will report all protocol deviations (including those found by study monitors) to their local IRBs as per their policies. The Principal Investigator will review each protocol deviation for potential impact on evaluations of safety and efficacy and subsequent reports will be submitted to the NIAMS/NIDDK IRB as appropriate.

Leidos Biomedical Research, Inc., will provide clinical research monitoring services for all subjects to be enrolled in this protocol at all participating sites. Leidos Biomedical Research-Fredrick Inc., is responsible for monitoring the conduct of the trial, for verifying adherence to the protocol, and data quality through confirming the completeness, consistency and accuracy of all documented data. Monitoring services will include:

- Good Clinical Practice regulator review at all performance sites
- Patient Case Review at all performance sites to include review of case report form completion and protocol and informed consent compliance
- protocol drug accountability at each site's pharmacy

In addition the Leidos Biomedical Research Inc., will provide regulatory consultative services to the central study coordinator and training in Good Clinical Practice requirements for all participating site personnel. Monitoring services by the Leidos Biomedical Research Inc., at each participating site will be performed according to the established monitoring plan.

Study staff will complete electronic case report forms (eCRFs) via a web-based electronic data capture (EDC) system (Clinical Trials Database, CTDB) that is compliant with Part 11 Title 21 of the Code of Federal Regulations. Subject electronic medical records in the Clinical Research Information System (CRIS) will be used as source documents for these eCRFs.

Subjects will also complete periodic questionnaires as outlined in the study procedures through the Clinical Trial Survey System (CTSS) – an ancillary web-based application associated with CTDB. These patient completed questionnaires in CTSS will be used as source documents for the study. The CTSS is accessible outside the NIH and allows subjects to remotely respond to clinical questionnaires with secure passwords administered by CTDB. Security of the database system is maintained through an application firewall, military grade encryption and SSL certificates, removal of personal identifiers consistent with HIPAA requirements (45 C.F.R. 164.514(a),(b)&(c)), and the incorporation of audit trails. The servers are physically located in a secured NIH data center with controlled limited access.

CRFs and patient questionnaire data will be kept in the CTDB database.

The data will be further validated via a series of manual edit checks, and all relevant data queries will be raised and resolved on an ongoing basis. Complete, clean data will be frozen to prevent further inadvertent modifications. All discrepancies will be reviewed and any resulting queries will be resolved with the investigators and amended in the database. All elements of data entry (i.e., time, date, verbatim text, and the person performing the data entry) will be recorded in an electronic audit trail to allow all data changes in the database to be monitored and maintained in accordance with federal regulations.

10.3.6 **<u>Review Schedule:</u>**

The Leidos Biomedical Research, Inc., will provide monitoring services to assess performance at a minimum of 3 visits (1-2 days per visit):

- Initial site monitoring visit: Prior to study start-up for GCP and protocol overview

- Follow-up monitoring visits: One year post enrollment of the first subject and every year thereafter as long as the site continues to enroll subjects. However, additional visits may be performed for targeted reviews as required or for high enrolling sites.
- Final monitoring visit: Study close out

10.3.7 Reporting:

Regular monitoring reports will be generated by the Leidos Biomedical Research, Inc., after each visit and will be provided to Principal Investigator (PI). Resolution of queries and outstanding issues or concerns will be the responsibility of the PI.

The PI will be responsible for reporting incidents of IRB non-compliance to the NIH IRB (in compliance with regulations on the protection of human subjects and institutional policy and procedures) and responsible for securing compliance.

10.4 Institutional Review Board:

The principal investigator must provide for the review and approval of this protocol and associated informed consent documents by NIAMS/NIDDK IRB at the NIH. Any amendments to the protocol or consent materials must be approved by the NIAMS/NIDDK IRB before they are placed into use.

The principal investigator will inform the IRB of serious or unexpected AEs that might occur during the study and are likely to affect the safety of the subjects, or the conduct of the study according to the NIH HRPP SOP 16 v2(10/11/13): "Reporting Requirements for Unanticipated Problems, adverse Events and Protocol Deviations". The principal investigator will comply fully with all IRB requirements for both the reporting of AEs, protocol or consent form changes, as well as any new information pertaining with the use of the study medication that might affect the conduct of the study.

10.5 Recruitment of women, children and minorities:

Minors will not be included in this study because this is a Phase Ib study that involves greater than a minor increment over minimal risk without the prospect of direct benefit to subjects based on currently available data.

The sex distribution (female:male) for SLE is 9:1. Lupus is more prevalent in African-Americans and Hispanics. We expect to recruit subjects indicative of this population distribution. If subject enrollment is not indicative of the Lupus population of the United States, then alternative methods for recruitment will be considered such as:

- contacting our referring physicians and requesting the appropriate study subjects and/or
- contacting health professionals in urban tertiary referral centers for African-, Hispanic-, or Asian-American subject recruitment.

10.6 Subject selection:

Pregnant and lactating women will be excluded from the study because the safety of tofacitinib during pregnancy/lactation has not been established. All subjects of childbearing potential will be required to use an effective method of contraception during the study. A pregnancy test will also be performed at pre-screening, during the Screening Period, before each study treatment, and on the follow up visits. Other vulnerable populations (minors and prisoners) will also be excluded from participating in this study.

10.7 Qualification of Investigators:

- Dr. Sarfaraz Hasni MD, will be the Principal Investigator (PI) for the NIH site. He is board certified in internal medicine and rheumatology. He is involved with clinical research at the NIH and is currently PI for another SLE treatment protocol.
- Dr. Meggan Mackay MD, will be the Associate Investigator. She is a clinical investigator with experience in the design and conduct of clinical trials and translational studies in lupus.
- Dr. John O'Shea MD, will be the Associate Investigator. He has made significant contributions to the discovery of JAK-STAT pathway and subsequent development of JAK inhibitors.
- Dr. Betty Diamond MD, will be the Associate Investigator. She is a physician-scientist with significant experience in clinical research involving SLE patients.

- Dr. Mariana Kaplan MD, will be lead medical Investigator. She is a physician-scientist with significant experience in clinical research involving SLE patients. She will be one of the investigators responsible for informed consent process.
- Dr. Nehal Mehta, cardiologist at the NHLBI and will be evaluating and interpreting vascular studies.
- Dr. Yasuko Furumoto, staff scientist at the NIH and will be responsible for the mechanistic studies
- Dr. Alan Remaley, is a senior investigator at the NHLBI and will be respponsible for the lipoprotein assays and analysis
- Dr. Peter Gregerson, MD is a collaborator at the FIMR and head of the Center for Genomics. He will supervise the genotyping and microarray studies.
- Dr. Cynthia Aranow will be the associate investigator at the FIMR. She is a clinical investigator with experience in the design and conduct of clinical trials and translational studies.
- Daniela Schwartz M.D. will be the associate investigator at the NIAMS. She will work on the mechanistic studies in the lab of Dr. John O'Shea and will also perform protocol related visits requiring a medical evaluation. She will be one of the investigators responsible for informed consent process.
- Shubhasree Choudhury M.D., will be the associate investigator at the NIAMS. She will perform protocol related visits requiring medical evaluation. She will be one of the investigators responsible for informed consent process.
- Yenealem Temesgen-Oyelakin, BSN, RN, will be the study coordinator. She will be one of the investigators responsible for informed consent process.
- Elizabeth Joyal, BSN, RN, will be the back–up study coordinator. She will be one of the investigators responsible for informed consent process.
- Massimo Gadina, PhD, will be supervising the mechanistic studies.

- Simantini Sakhardande, will be performing vascular function studies.
- Alice Fike, MSN will be the nurse practitioner on the study and will perform the majority of protocol related visits requiring a medical evaluation. She will be one of the investigators responsible for informed consent process.

11 BENEFITS:

There may be no direct benefits to participants. Subjects may benefit from a thorough evaluation by experts in SLE.

12 SIGNIFICANCE TO BIOMEDICAL RESEARCH:

If this regimen is shown to be devoid of any significant toxicities, further efficacy studies will be planned. This agent is not expected to be associated with the most common toxicities of therapies commonly used in the treatment of SLE, such as severe immunosuppression, myelosuppression, amenorrhea and osteoporosis. This study will provide important preliminary information about the effect of JAK-STAT pathway inhibition in SLE patients and may contribute to better understanding of the pathophysiology of SLE.

13 SUMMARY/CLASSIFICATION OF RISK:

This study involves more than minimal risk without the prospect of direct benefit to subjects based on currently available data. There is the prospect of generalizable knowledge and better understanding of the disease process.

14 INFORMED CONSENT DOCUMENTS AND PROCESS:

The principles of informed consent in the current edition of the Declaration of Helsinki, as well as compliance with all IRB requirements, will be implemented in the study, before any protocol-specified procedures are carried out. A standard consent form for subject participation will be provided with the protocol to the IRB and Office of Protocol Services (OPS) of the NIH. Any modifications to the standard information in the template will require review and approval by the IRB. All subjects will receive a consent form that will include the purposes, procedures, benefits, and potential hazards of the study. This information will be reviewed with the subject by either

the principal or a qualified associate investigator. All prospective subjects will be given ample time to read the consent form, and ask questions, before signing.

The consent documents will be translated into Spanish. For Spanish speaking subjects the consent will be explained by the PI/AI through an interpreter. All subjects will be informed of their right to withdraw from the study. Translated documents must be certified to contain the complete descriptions provided in the English version of the document.

For inclusion in the study, each subject will be required to sign the consent form. The original forms will become part of the permanent medical record and kept on file in the subject's study chart, available for inspection by regulatory authorities, both federal and institutional. Copies will be provided to the subjects. The fact that informed consent was obtained prior to the initiation of study procedures will be documented in the subject's medical records.

The principal investigator (PI) will be responsible to ensure that informed consent is obtained consistent with the requirements as outlined in the document HRPP SOP 12, *REQUIREMENTS FOR INFORMED CONSENT.* The informed consent will be obtained by the PI or designated investigators as listed under section 10.8 Qualification of Investigators .
All the relevant research information necessary to make an informed decision will be disclosed to the prospective subjects. The individual obtaining the informed consent will facilitate the understanding of what has been discussed. The individual obtaining the informed consent will make every effort to minimize any possibility of coercion and undue influence.
All subjects must provide written informed consent to participate and consent will be documented using the most current IRB-approved consent form. For our Spanish speaking subjects, we will use the Spanish translation of our Informed Consent Document.

All subjects must provide written informed consent to participate and consent will be documented using the most current IRB-approved consent form. For our Spanish speaking subjects, we will use the Spanish translation of our Informed Consent Document. This study does not target or enroll any vulnerable populations.

15 GCP COMPLIANCEAND DATA AND SAFETY MONITORING AND REPORTING:

This section defines the types of safety data that will be collected under this protocol and outlines the procedures for appropriately collecting, grading, recording and reporting that data. Serious

adverse events must be reported promptly to the IND sponsor and NIAMS/NIDDK Institutional Review Board (IRB). IRB reporting of AEs will be done as mandated by NIH policies ~~NIAMS/NIDDK IRB~~ Information in this section complies with ICH Guideline E2A: Clinical Safety Data Management: Definitions and Standards for Expedited Reporting, ICH Guideline E-6: Guideline for Good Clinical Practice and applies the standards set forth in the NCI CTCAE, version 4 and NIH HRPP SOP 16 v2 (10/11/13): "Reporting Requirements for Unanticipated Problems, adverse Events and Protocol Deviations". Data for this study will be collected on electronic Case Report Forms (eCRFs) that will be transmitted to the NIH CTDB. The Principal Investigator, Dr. Hasni, will oversee the conduct of the study.

15.1 <u>Clinical Monitoring:</u>

Study procedures will be subject to audits and/or monitoring visits to ensure compliance with the protocol and applicable regulatory requirements consistent with the NIAMS quality assurance program plan. Audit and/or monitoring visit results will be reported to the Principal Investigator for further reporting as appropriate. Study documents and pertinent hospital or clinical records will be reviewed to verify that the conduct of the study is consistent with the protocol plan. The clinical monitoring plan will be developed by the Leidos Biomedical Research, Inc. Clinical Monitoring Research Program (CMRP), Clinical Trials Management Team (CTM) in collaboration with the Principal Investigator. The purposes of the clinical monitoring activities are:

1. to verify the existence of signed informed consent documents and documentation of the ICF process for each monitored subject;
2. to verify the prompt and accurate recording of all monitored data points, and prompt reporting of all SAEs;
3. to compare abstracted information in the electronic database with individual subjects' records and source documents (subjects' charts, laboratory analyses and test results, physicians' progress notes, nurses' notes, and any other relevant original subject information);
4. to help ensure investigators are in compliance with the protocol.

The monitors will also inspect the clinical site regulatory files to ensure that regulatory requirements (Office for Human Research Protections-OHRP) and applicable guidelines (ICH-GCP) are being followed. Some monitoring activities may be performed remotely (e.g.,

review of regulatory documents), while others will take place on site (e.g., verification of study databases against source documentation). Staff from Leidos Biomedical Research Inc., will conduct the monitoring activities and provide follow-up letters describing the findings. The frequency of reporting for monitoring activities will be specified in the monitoring plan.

15.2 Data and Safety Monitoring Committee Oversight:

The NIAMS Data and Safety Monitoring Committee (DSMC) will have safety oversight responsibilities for the study. A separate committee will be formed specifically for this protocol and will include members with expertise in a broad range of areas, including human subjects' protection, research ethics, clinical trial implementation, immunology, and rheumatology. Approximately semi-annually, the DSMC will review data related to enrollment progress, study implementation, subject safety, and protocol violations. The DSMC will primarily review blinded data but the committee chair may also request un-blinding of treatment allocation or group assignment of individual subjects on as needed basis. These requests will be transmitted to the NIH Clinical Center Research Pharmacy by the principal investigator. The data will be provided directly to the Chair of DSMC without un-blinding the investigators before the DSMC determines a plan of action.

The CTDB will generate reports that compile all newly submitted and accumulated AEs, SAEs, toxicities, pregnancies, and concomitant medications. Subsequent review of periodic reports will be performed by the Principal Investigator.

The DSMC will also consider current information from other sources on the biology of the disease and the subject population under study. Based on these reviews, the DSMC will make recommendations to the Principal Investigator and the NIAMS Clinical Director concerning the continuation, modification, or termination of the study. The DSMC will also meet ad hoc if relevant issues arise that require committee review. The roles and responsibilities of committee members and meeting procedures are formally described in the NIAMS Charter.

15.3 Suspension Guidelines:

The DSMC chair will be alerted if any of the following situations occur:

- An unexpected fatal or life-threatening event assessed as related to the use of study drug.
- Three or more subjects with similar severe AEs assessed as related to the use of study drug.

While the DSMC has ultimate authority to establish safety, we have established additional pre-determined stopping rules as defined by the number and type of AEs (Table 2). The Principal Investigator will be responsible for monitoring the accruing safety data related to suspension guidelines and for alerting the DSMC chair when a criterion is met. The DSMC chair will be alerted by email within 7 calendar days of determination that a criterion has been met. The DSMC will issue a recommendation on study continuation to the NIAMS Clinical Director after reviewing data related to the suspension guideline. If the study is stopped, subjects will receive conventional care for any study-related AEs and continue to be followed for clinical and safety outcomes. Otherwise, the study will continue per the DSMC recommendations. The Principal Investigator and the Clinical Director will provide the recommendations of the DSMC to the IRB.

Table 2: Suspension Guidelines for DSMC

First 5 subjects	Three grade 3 or 4 AEs of same type<br>Five grade 3 or 4 AEs of any type
First 10 subjects	Four grade 3 or 4 AEs of same type<br>Five grade 3 or 4 AEs of any type
All 20 subjects	Eight grade 3 or 4 AEs of same type<br>Ten grade 3 or 4 AEs of any type

16 ADVERSE EVENT AND UNANTICIPATED PROBLEM REPORTING:

The Principal Investigator will be responsible for detecting, documenting, and reporting AEs and SAEs in accordance with the protocol, IRB requirements, and federal regulations.

16.1 Definitions:

16.1.1 Adverse Event:

An adverse event (AE) is any unfavorable and unintended diagnosis, symptom, sign (including an abnormal laboratory finding), syndrome, or disease that either occurs during the study, having been absent at baseline, or if present at baseline, appears to worsen. All AEs will be graded for intensity (severity) and relationship to study drug.

16.1.2 Unanticipated Problem:

The Office for Human Research Protections considers unanticipated problems to be any incident, experience, or outcome that meets all of the following criteria:

- Is unexpected in terms of nature, severity, or frequency given a) the research procedures that are described in the IRB-approved research protocol and informed consent, and b) the characteristics of the subject population being studied;

- Is related or possibly related to participation in the research (possibly related means there is a reasonable possibility that the incident, experience, or outcome may have been caused by the procedures involved in the research); and

- Places subjects or others at a greater risk for physical, psychological, economic, or social harm than was previously known or recognized.

An incident, experience, or outcome that meets the 3 criteria above will generally warrant consideration of substantive changes in order to protect the safety, welfare, or rights of subjects or others. Examples of corrective actions or substantive changes that might need to be considered in response to an unanticipated problem include the following:

- Changes to the research protocol initiated by the investigator prior to obtaining IRB approval to eliminate apparent immediate hazards to subjects.

- Modification of inclusion or exclusion criteria to mitigate the newly identified risks.

- Implementation of additional procedures for monitoring subjects.

- Suspension of enrollment of new subjects.

- Suspension of research procedures in currently enrolled subjects.

- Modification of informed consent documents to include a description of newly recognized risks.

- Provision of additional information about newly recognized risks to previously enrolled subjects.

Per the definition, only a subset of AEs would be further characterized as unanticipated problems. Additionally, there are other sorts of events that, while not AEs, would also be characterized as unanticipated problems (e.g., contaminated study drug).

16.1.3 <u>Serious Adverse Event:</u>

A serious adverse event (SAE) is defined as any untoward medical occurrence that:

- Results in death,
- Is life-threatening (defined as a subject at immediate risk of death at the time of the event; it does not apply to an AE which hypothetically might have caused the death if it were more severe),
- Requires or prolongs hospitalization (i.e. the AE required at least a 24-hour inpatient hospitalization or prolonged a hospitalization beyond the expected length of stay; hospitalizations for elective medical/surgical procedures, scheduled treatments, or routine check-ups are not SAEs by this criterion),
- Results in a congenital anomaly or birth defect (i.e., an adverse outcome in a child or fetus of a patient exposed to the trial drug prior to conception or during pregnancy),
- Causes a persistent or significant disability/incapacity (i.e. the AE resulted in a substantial disruption of the patient's ability to carry out normal life functions), or
- Is any other condition that, in the judgment of the investigator, represents a significant hazard or it does not meet any of the above serious criteria but may jeopardize the patient and may require medical or surgical intervention to prevent one of the outcomes listed above.

16.1.4 <u>Medical Events Not Qualifying as Adverse Events or Serious Adverse Events:</u>

Signs and symptoms of pre-existing medical conditions will not be recorded or reported as AEs or SAEs, unless they represent a clinically significant change from the baseline disease status documented at the Pre-screening Visit. In addition, hospitalization for elective procedures or surgeries will not be considered SAEs, nor will inpatient hospitalizations for convenience.

16.1.5 Clinical Laboratory Test Results Not Qualifying as Adverse Events or Serious Adverse Events:

A clinically significant laboratory result that is present at baseline and does not change significantly during the study will not be reported as an AE or SAE. The clinical significance of a change in a laboratory result will be determined by the investigator.

16.1.6 Lupus Flare:

A lupus flare is any significant worsening of the signs, symptoms and laboratory test abnormalities associated with lupus. Any increase in the SLEDAI 2K index of 3 or more will be considered as a SLE flare.

16.1.7 Reporting of Adverse Events, Unanticipated Problems and Protocol Deviations:

16.1.7.1 Intensity of Adverse Event:

The intensity (severity) of AEs and SAEs will be graded according to a descriptive scale based on the National Cancer Institute CTC Version 4.0. (Appendix 22.11) and includes the grades of 1) mild, 2) moderate, 3) severe, and 4) potentially life threatening.

16.1.7.2 Relationship to Study Drug and Procedures:

For all AEs and SAEs, the investigator will provide his best estimate of the causal relationship between the event and study drug, and the causal relationship between the event and study procedures. The degree of certainty about causality will be graded according to the criteria in Table .

Table 3. Relatedness of Adverse Event to Intervention

Causality	Description
Not Related Category	
Unrelated	Adverse event is clearly due to extraneous causes (e.g., underlying disease, environment)
Related Category	
Unlikely (must have at least 2)	1) does not have temporal relationship to intervention<br>2) could readily have been produced by the subject's clinical state<br>3) could have been due to environmental or other interventions<br>4) does not follow a known pattern of response to intervention<br>5) does not reappear or worsen with reintroduction of intervention
Possible (must have at least 2)	1) has a reasonable temporal relationship to intervention<br>2) could not readily have been produced by the subject's clinical state<br>3) could not readily have been due to environmental or other interventions<br>4) follows a known pattern of response to intervention
Probable (must have at least 3)	1) has a reasonable temporal relationship to intervention<br>2) could not readily have been produced by the subject's clinical state or have been due to environmental or other interventions<br>3) follows a known pattern of response to intervention<br>4) disappears or decreases with reduction in dose or cessation of intervention
Definite (must have all 4)	1) has a reasonable temporal relationship to intervention<br>2) could not readily have been produced by the subject's clinical state or have been due to environmental or other interventions<br>3) follows a known pattern of response to intervention<br>4) disappears or decreases with cessation of intervention and recurs with re-exposure

16.1.7.3 <u>Expectedness of Adverse Events:</u>

For purposes of regulatory reporting, the medically responsible investigator will determine whether an AE or SAE is expected or unexpected. Expected adverse events are those adverse events that are listed or characterized in the Package Insert or in the Physicians' Desk Reference.

Unexpected adverse events are those not listed in the Package Insert (P.I.) or Physicians' Desk Reference (PDR), published medical literature, protocol, informed consent document or not identified. This includes adverse events for which the specificity or severity is not

consistent with the description in the P.I. or PDR. For example, under this definition, hepatic necrosis would be unexpected if the P.I. or I.B. only referred to elevated hepatic enzymes or hepatitis.

For consistency of labeling and categorizing adverse events, the National Cancer Institute (NCI) Common Terminology Criteria for Adverse Events, Version 4.0 will be used in this study. NCI CTC Version .0, DCTD, NCI, NIH, DHHS; June 14, 2010, publish date: May 28, 2009 (http://ctep.cancer.gov).

16.1.8 Procedures for Reporting

Serious adverse events, unexpected AEs, and unanticipated problems will be reported to the IRB, NIAMS Clinical Director, DSMC and FDA according to the NIH-OHSRP SOP 16, "Reporting Requirements for Unanticipated Problems, Adverse Events and Protocol Deviations.". All patients who receive at least one dose or part of a dose of the trial medication (tofacitinib) and complete a safety follow-up, whether withdrawn prematurely or not, will be included in the safety analyses. All data relating to safety will be listed and summarized separately for the treatment period and for the entire study. All safety reports will be reviewed by the Principal Investigator.

16.1.9 Reporting Timeline:

Adverse events, protocol deviations, unanticipated problems (UP), Unanticipated Adverse Device Effects (UADEs), serious adverse events, sponsor and serious, are defined as described in NIH HRPP SOP 16 ("Reporting Requirements for Unanticipated Problems, Adverse Events and Protocol Deviations."). All adverse events occurring during the study, including those observed by or reported to the research team, will be recorded. Serious unanticipated problems and serious protocol deviations, will be reported to the IRB and CD as soon as possible but not more than 7 days after the PI first learns of the event. Not serious unanticipated problems will be reported to the IRB and CD as soon as possible but not more than 14 days after the PI first learns of the event. Not serious protocol deviations will be reported to the IRB as soon as possible but not more than 14 days after the PI first learns of the event.

Deaths will be reported to the Clinical Director and IRB within 7 days after the PI first learns of the event.

16.1.10 Reporting of Non-Serious Protocol Deviations:

Non-serious protocol deviations will only be reported to the IRB (within 14 days after the PI first learns of the event) if they represent a departure from NIH policies for the conduct of human subjects research, adversely affect the health care of the subject(s) or compromise the interpretation or integrity of the research. Non-serious protocol deviations that result from normal subject scheduling variations or technical issues associated with sampling that does not impact the health of the subject or the interpretation of the study data will not be reported.

16.1.11 Reporting of Adverse Events:

The PI is responsible for summarizing all serious adverse events and adverse events at least possibly related to the research procedure and interventions at the time of Continuing Review.

16.1.12 Reporting of Deaths:

All deaths that have occurred among study participants since the previous review will be summarized at the time of continuing review.

16.1.13 Reporting Waivers:

Waiver of Reporting to the IRB of anticipated minor protocol deviations, adverse events and deaths due to underlying disease or population under study unless determined to be an Unanticipated Problem.

- The following anticipated minor deviations in the conduct of the protocol will not be reported to the IRB unless they occur at a rate greater than that which is anticipated to occur: Deviation in blood draw or visit dates due to any unexpected closure of federal government due to inclement weather or otherwise, a single missed time-point for blood draw in the mechanistic studies. If the rate of these events exceeds the rate specified by the protocol, the events will be classified and reported as though they are Unanticipated Problems.
- The anticipated non-UP adverse events will not be reported to the IRB unless they occur at a rate greater than that known to occur in patients with SLE. If events are occurring substantially more frequent that would be anticipated in typically treated patients with SLE, they will also be reported to IRB. The following anticipated adverse events will not be reported to the IRB unless they occur at a severity greater than that known to occur in patients taking tofacitinib: mild infections of upper respiratory tract, oropharynx

and urinary tract treated with or without oral antibiotics, lymphopenia with absolute lymphocyte count of more than 500 cells/mm^3, neutropenia with an absolute neutrophil count of more than 1000 cells/mm^3, mild anemia with drop in hemoglobin of less than 1.5g/dl, increased liver enzyme tests which are not greater than 2 times the upper limit of normal, a mean increase in LDL by less than 15 %, and a mean increase in HDL by less than 10%. If the rate of these events exceeds the rate specified in the protocol or investigator's brochure the events will be classified and reported as though they are Unanticipated Problems.

16.2 Adverse Event, Protocol deviation and Unanticipated Problem Assessment and Follow-up:

In the event of an adverse event, protocol deviation and unanticipated problem the first concern will be for the safety of the patients. Investigators are required to collect and document all adverse events (AEs), protocol deviations and unanticipated problems. At each study visit, the Principal Investigator will inquire about the occurrence of AE/SAEs since the last visit, and review any protocol deviations and unanticipated problems. Adverse events (including SAEs), protocol deviations, and unanticipated problems may be discovered through any of these methods:

- Observing the subject.
- Questioning the subject in an objective manner.
- Receiving an unsolicited complaint from the subject.
- Review of all source documentation related to study procedures; abnormal values or results from clinical or laboratory evaluations (including, but not limited to, radiographs, ultrasounds, or electrocardiograms) can also indicate adverse events.

Events will be followed for outcome information until they return to baseline or stabilize. Study-related AEs will be followed and/or treated at the NIH until resolution or stabilization of the AE, after which the subject will be referred to a physician(s) outside of the NIH for care and follow-up.

16.2.1 Adverse Event Recording:

Adverse events will be monitored throughout this study, and these events will be recorded on the appropriate AE eCRF at each visit. The record for each event will include the following information:

- Description of the event.
- Onset and stop dates of the event.
- Seriousness of event.
- Intensity (or severity) of the event.
- Action taken because of the event.
- Relationship of the event to study drug and/or study procedure.
- Outcome of the event.
- Expectedness of the event.

16.2.2 Clinically Significant Laboratory Abnormalities:

The Principal Investigator or designated AI will evaluate all clinical laboratory and imaging results for clinically significant abnormalities and document the evaluation in the medical record and case report form. A laboratory abnormality will be documented as an adverse event using the following criteria:

- The abnormality is not already encompassed by a reported adverse event (e.g., elevated AST need not be reported as an AE if Liver Failure has already been reported as an AE).
- The abnormality is considered clinically significant by the Investigator. Clinically significant lab abnormality is defined as meeting the following:
  - Necessitates study drug dosing modification (i.e., dose reduction, interruption or discontinuation)~~;~~ and/or
  - Requires a therapeutic intervention (e.g., concomitant medication, blood transfusion or dialysis)~~;~~ and
  - Is unexplainable by the patient’s current and past medical conditions

The Principal Investigator will follow significant abnormalities until they return to baseline or stabilize.

16.2.3 Pregnancy Reporting and Follow-up:

This study includes pregnancy information as safety data and pregnancies will be recorded if they begin any time after enrollment. Information about any pregnancy should be reported

promptly to the NIH NIAMS/NIDDK IRB, NIAMS Clinical Director, and DSMC on the same timeline as a SAE. All pregnancies identified during the study must be followed to conclusion and the outcome of each must be reported. The investigator should be informed immediately of any pregnancy in a study subject or a partner of a study subject. A pregnant subject should be instructed to stop taking study medication. The investigator should counsel the subject and discuss the risks of continuing with the pregnancy and the possible effects on the fetus. Tofacitinib has a pregnancy risk factor Category C; there are no adequate and well controlled studies in pregnant women. Tofacitinib is fetocidal and teratogenic in rats and rabbits when given at exposures 146 times and 13 times, respectively, the maximum recommended human dose. Monitoring of the pregnant subject should continue until the conclusion of the pregnancy, and a follow-up Pregnancy Monitoring form detailing the outcome of the pregnancy should be submitted to the IRB. When possible, similar information should be obtained for a pregnancy occurring in a partner of a study subject. Information requested about the delivery will include:

- Subject's enrollment ID
- Gestational age at delivery
- Birth weight, length, and head circumference
- Gender
- Appearance, pulse, grimace, activity, and respiration (APGAR) score at 1 minute, 5 minutes, and 24 hours after birth, if available
- Any abnormalities.

  Should the pregnancy result in a congenital abnormality or birth defect, an SAE also must be submitted to the NIH NIAMS/NIDDK IRB using the SAE reporting procedures described above.

16.2.4 Lost to follow up patient reporting:

After three attempts to contact the patient via phone, a certified letter will be sent to notify him or her that they have been withdrawn from the study.

17 ALTERNATIVES TO PARTICIPATION OR ALTERNATIVE THERAPIES:

The alternative to participating in this study is to receive conventional treatment. There are several treatment options available for SLE patients with mild to moderate disease activity. The options include: higher doses of corticosteroids; switching to or adding another immunosuppressant; or using a biologic, such as belimumab, which was recently approved for SLE.

18 CONFIDENTIALITY:

A unique coded study number will be assigned to each subject for data collection. The number will not contain any personal information (dates, age) to further ensure protection. Research records will be kept in locked cabinets or rooms, and computer research databases will be stored on NIH computers, which are password protected and encrypted. Only members of the study staff and monitors will have access to study samples and data. Clinical data will be stored in CRIS at the NIH and will be protected by standard measures.

19 CONFLICT OF INTEREST/TECHNOLOGY TRANSFER:

The NIH guidelines on conflict of interest have been distributed to all investigators.

The NIH and Dr. John O'Shea have a patent related to JAK inhibitors and receive royalties. The NIH and Dr. O'Shea have had a collaborative agreement and development award (CRADA) with Pfizer that pertains to JAK inhibition and tofacitinib. The NIH and Dr. O'Shea have an ongoing CRADA for new JAK inhibitors. None of the other investigators have any financial conflicts of interest to report.

The Principal Investigator will seek prospective and continuing NIH IRB review and approval for research collaborations in which coded samples (for which the investigators maintain the key) are sent to non-NIH investigator(s). He will identify the names of the collaborating researchers and their affiliated institutions.

20 RESEARCH AND TRAVEL COMPENSATION:

Travel reimbursement will be provided in accordance with NIH regulations. Subjects will receive financial compensation for time required to participate in the research as per the guidelines provided in HRPP SOP 13, RECRUITMENT, SELECTION AND

COMPENSATION OF RESEARCH SUBJECTS. The subjects will be paid $ 60 at each outpatient visit. In addition subjects will be paid an additional $100 at the end of study visit Day 84 as a study completion bonus. Any subject who drops off the study or withdrawn from the study will be paid for the number of visits completed.

21 REFERENCES:

1. GC T. - Systemic lupus erythematosus. N Engl J Med. 2011;365(22):2110-21. - eng.

2. PJ M. - The JAK-STAT signaling pathway: input and output integration. J Immunol. 2007;178(5):2623-9. - eng.

3. MJ K. - Neutrophils in the pathogenesis and manifestations of SLE. Nat Rev Rheumatol. 2011;7(12):691-9. - eng.

4. Murray PJ. The JAK-STAT signaling pathway: input and output integration. J Immunol. 2007 Mar 1;178(5):2623-9. PubMed PMID: 17312100. Epub 2007/02/22. eng.

5. JJ OS, Holland Sm Fau - Staudt LM, LM S. - JAKs and STATs in immunity, immunodeficiency, and cancer. N Engl J Med. 2013;368(2):161-70. - eng.

6. S W, Yang N Fau - Zhang L, Zhang L Fau - Huang B, Huang B Fau - Tan H, Tan H Fau - Liang Y, Liang Y Fau - Li Y, et al. - Jak/STAT signaling is involved in the inflammatory infiltration of the kidneys in MRL/lpr mice. Lupus. 2010;19(10):1171-80. - eng.

7. Lu LD, Stump KL, Wallace NH, Dobrzanski P, Serdikoff C, Gingrich DE, et al. Depletion of autoreactive plasma cells and treatment of lupus nephritis in mice using CEP-33779, a novel, orally active, selective inhibitor of JAK2. J Immunol. 2011 Oct 1;187(7):3840-53. PubMed PMID: 21880982. Epub 2011/09/02. eng.

8. O'Neill S, Cervera R. Systemic lupus erythematosus. Best Pract Res Clin Rheumatol. 2010 Dec;24(6):841-55. PubMed PMID: 21665130. Epub 2011/06/15. eng.

9. Lo MS, Tsokos GC. Treatment of systemic lupus erythematosus: new advances in targeted therapy. Ann N Y Acad Sci. 2012 Jan;1247:138-52. PubMed PMID: 22236448. Epub 2012/01/13. eng.

10. Aringer M, Smolen JS. Tumour necrosis factor and other proinflammatory cytokines in systemic lupus erythematosus: a rationale for therapeutic intervention. Lupus. 2004;13(5):344-7. PubMed PMID: 15230290. Epub 2004/07/03. eng.

11. Aringer M, Smolen JS. Cytokine expression in lupus kidneys. Lupus. 2005;14(1):13-8. PubMed PMID: 15732282. Epub 2005/03/01. eng.

12. Dean GS, Tyrrell-Price J, Crawley E, Isenberg DA. Cytokines and systemic lupus erythematosus. Ann Rheum Dis. 2000 Apr;59(4):243-51. PubMed PMID: 10733469. Pubmed Central PMCID: PMC1753117. Epub 2000/03/25. eng.

13. Banchereau J, Steinman RM. Dendritic cells and the control of immunity. Nature. 1998 Mar 19;392(6673):245-52. PubMed PMID: 9521319. Epub 1998/04/01. eng.

14. Bennett L, Palucka AK, Arce E, Cantrell V, Borvak J, Banchereau J, et al. Interferon and granulopoiesis signatures in systemic lupus erythematosus blood. J Exp Med. 2003 Mar 17;197(6):711-23. PubMed PMID: 12642603. Pubmed Central PMCID: PMC2193846. Epub 2003/03/19. eng.

15. Vallin H, Blomberg S, Alm GV, Cederblad B, Ronnblom L. Patients with systemic lupus erythematosus (SLE) have a circulating inducer of interferon-alpha (IFN-alpha) production acting on leucocytes resembling immature dendritic cells. Clin Exp Immunol. 1999

Jan;115(1):196-202. PubMed PMID: 9933442. Pubmed Central PMCID: PMC1905199. Epub 1999/02/05. eng.

16. Balomenos D, Rumold R, Theofilopoulos AN. Interferon-gamma is required for lupus-like disease and lymphoaccumulation in MRL-lpr mice. J Clin Invest. 1998 Jan 15;101(2):364-71. PubMed PMID: 9435308. Pubmed Central PMCID: PMC508575. Epub 1998/02/07. eng.

17. Clynes R, Dumitru C, Ravetch JV. Uncoupling of immune complex formation and kidney damage in autoimmune glomerulonephritis. Science. 1998 Feb 13;279(5353):1052-4. PubMed PMID: 9461440. Epub 1998/03/07. eng.

18. Boswell JM, Yui MA, Burt DW, Kelley VE. Increased tumor necrosis factor and IL-1 beta gene expression in the kidneys of mice with lupus nephritis. J Immunol. 1988 Nov 1;141(9):3050-4. PubMed PMID: 3262676. Epub 1988/11/01. eng.

19. Brennan DC, Yui MA, Wuthrich RP, Kelley VE. Tumor necrosis factor and IL-1 in New Zealand Black/White mice. Enhanced gene expression and acceleration of renal injury. J Immunol. 1989 Dec 1;143(11):3470-5. PubMed PMID: 2584702. Epub 1989/12/01. eng.

20. Lemay S, Mao C, Singh AK. Cytokine gene expression in the MRL/lpr model of lupus nephritis. Kidney Int. 1996 Jul;50(1):85-93. PubMed PMID: 8807576. Epub 1996/07/01. eng.

21. Herrera-Esparza R, Barbosa-Cisneros O, Villalobos-Hurtado R, Avalos-Diaz E. Renal expression of IL-6 and TNFalpha genes in lupus nephritis. Lupus. 1998;7(3):154-8. PubMed PMID: 9607638. Epub 1998/06/02. eng.

22. Malide D, Russo P, Bendayan M. Presence of tumor necrosis factor alpha and interleukin-6 in renal mesangial cells of lupus nephritis patients. Hum Pathol. 1995 May;26(5):558-64. PubMed PMID: 7750940. Epub 1995/05/01. eng.

23. Chan RW, Lai FM, Li EK, Tam LS, Chow KM, Li PK, et al. The Effect of immunosuppressive therapy on the messenger RNA expression of target genes in the urinary sediment of patients with active lupus nephritis. Nephrol Dial Transplant. 2006 Jan 31. PubMed PMID: 16449281.

24. Iwano M, Dohi K, Hirata E, Kurumatani N, Horii Y, Shiiki H, et al. Urinary levels of IL-6 in patients with active lupus nephritis. Clin Nephrol. 1993 Jul;40(1):16-21. PubMed PMID: 8358870. Epub 1993/07/01. eng.

25. Blomberg S, Eloranta ML, Cederblad B, Nordlin K, Alm GV, Ronnblom L. Presence of cutaneous interferon-alpha producing cells in patients with systemic lupus erythematosus. Lupus. 2001;10(7):484-90. PubMed PMID: 11480846. Epub 2001/08/02. eng.

26. Nurnberg W, Haas N, Schadendorf D, Czarnetzki BM. Interleukin-6 expression in the skin of patients with lupus erythematosus. Exp Dermatol. 1995 Feb;4(1):52-7. PubMed PMID: 7757333. Epub 1995/02/01. eng.

27. Pelton BK, Hylton W, Denman AM. Activation of IL-6 production by UV irradiation of blood mononuclear cells from patients with systemic lupus erythematosus. Clin Exp Immunol. 1992 Aug;89(2):251-4. PubMed PMID: 1638768. Pubmed Central PMCID: PMC1554444. Epub 1992/08/01. eng.

28. Ishida H, Muchamuel T, Sakaguchi S, Andrade S, Menon S, Howard M. Continuous administration of anti-interleukin 10 antibodies delays onset of autoimmunity in NZB/W F1

mice. J Exp Med. 1994 Jan 1;179(1):305-10. PubMed PMID: 8270873. Pubmed Central PMCID: PMC2191319. Epub 1994/01/01. eng.

29. Llorente L, Zou W, Levy Y, Richaud-Patin Y, Wijdenes J, Alcocer-Varela J, et al. Role of interleukin 10 in the B lymphocyte hyperactivity and autoantibody production of human systemic lupus erythematosus. J Exp Med. 1995 Mar 1;181(3):839-44. PubMed PMID: 7869046. Pubmed Central PMCID: PMC2191898. Epub 1995/03/01. eng.

30. Edwards CK, 3rd, Zhou T, Zhang J, Baker TJ, De M, Long RE, et al. Inhibition of superantigen-induced proinflammatory cytokine production and inflammatory arthritis in MRL-lpr/lpr mice by a transcriptional inhibitor of TNF-alpha. J Immunol. 1996 Aug 15;157(4):1758-72. PubMed PMID: 8759766. Epub 1996/08/15. eng.

31. Segal R, Dayan M, Zinger H, Mozes E. Suppression of experimental systemic lupus erythematosus (SLE) in mice via TNF inhibition by an anti-TNFalpha monoclonal antibody and by pentoxiphylline. Lupus. 2001;10(1):23-31. PubMed PMID: 11243506. Epub 2001/03/13. eng.

32. Su X, Zhou T, Yang P, Edwards CK, 3rd, Mountz JD. Reduction of arthritis and pneumonitis in motheaten mice by soluble tumor necrosis factor receptor. Arthritis Rheum. 1998 Jan;41(1):139-49. PubMed PMID: 9433879. Epub 1998/01/20. eng.

33. Llorente L, Richaud-Patin Y, Garcia-Padilla C, Claret E, Jakez-Ocampo J, Cardiel MH, et al. Clinical and biologic effects of anti-interleukin-10 monoclonal antibody administration in systemic lupus erythematosus. Arthritis Rheum. 2000 Aug;43(8):1790-800. PubMed PMID: 10943869. Epub 2000/08/16. eng.

34. Fleischmann R, Kremer J, Cush J, Schulze-Koops H, Connell CA, Bradley JD, et al. Placebo-controlled trial of tofacitinib monotherapy in rheumatoid arthritis. N Engl J Med. 2012 Aug 9;367(6):495-507. PubMed PMID: 22873530. Epub 2012/08/10. eng.

35. Kremer JM, Cohen S, Wilkinson BE, Connell CA, French JL, Gomez-Reino J, et al. A phase IIb dose-ranging study of the oral JAK inhibitor tofacitinib (CP-690,550) versus placebo in combination with background methotrexate in patients with active rheumatoid arthritis and an inadequate response to methotrexate alone. Arthritis Rheum. 2012 Apr;64(4):970-81. PubMed PMID: 22006202. Epub 2011/10/19. eng.

36. Riese RJ, Krishnaswami S, Kremer J. Inhibition of JAK kinases in patients with rheumatoid arthritis: scientific rationale and clinical outcomes. Best Pract Res Clin Rheumatol. 2010 Aug;24(4):513-26. PubMed PMID: 20732649. Epub 2010/08/25. eng.

37. van Vollenhoven RF, Fleischmann R, Cohen S, Lee EB, Garcia Meijide JA, Wagner S, et al. Tofacitinib or adalimumab versus placebo in rheumatoid arthritis. N Engl J Med. 2012 Aug 9;367(6):508-19. PubMed PMID: 22873531. Epub 2012/08/10. eng.

38. JJ OS, Laurence A Fau - McInnes IB, IB M. - Back to the future: oral targeted therapy for RA and other autoimmune diseases. Nat Rev Rheumatol. 2013;9(3):173-82. - eng.

39. J W, Silverfield J Fau - Lee EB, Lee Eb Fau - Curtis JR, Curtis Jr Fau - Wood SP, Wood Sp Fau - Soma K, Soma K Fau - Nduaka CI, et al. - Safety and Efficacy of Tofacitinib, an Oral Janus Kinase Inhibitor, for the Treatment of Rheumatoid Arthritis in Open-label, Longterm Extension Studies. J Rheumatol. 2014;41(5):837-52. - eng.

40. Meyer DM, Jesson MI, Li X, Elrick MM, Funckes-Shippy CL, Warner JD, et al. Anti-inflammatory activity and neutrophil reductions mediated by the JAK1/JAK3 inhibitor, CP-690,550, in rat adjuvant-induced arthritis. Journal of inflammation (London, England). 2010;7:41. PubMed PMID: 20701804. Pubmed Central PMCID: PMC2928212. Epub 2010/08/13. eng.

41. Ghoreschi K, Jesson MI, Li X, Lee JL, Ghosh S, Alsup JW, et al. Modulation of innate and adaptive immune responses by tofacitinib (CP-690,550). J Immunol. 2011 Apr 1;186(7):4234-43. PubMed PMID: 21383241. Pubmed Central PMCID: PMC3108067. Epub 2011/03/09. eng.

42. Maeshima K, Yamaoka K, Kubo S, Nakano K, Iwata S, Saito K, et al. The JAK inhibitor tofacitinib regulates synovitis through inhibition of interferon-gamma and interleukin-17 production by human CD4+ T cells. Arthritis Rheum. 2012 Jun;64(6):1790-8. PubMed PMID: 22147632. Epub 2011/12/08. eng.

43. Tanaka Y, Maeshima K, Yamaoka K. In vitro and in vivo analysis of a JAK inhibitor in rheumatoid arthritis. Ann Rheum Dis. 2012 Apr;71 Suppl 2:i70-4. PubMed PMID: 22460142. Epub 2012/04/04. eng.

44. Kim K, Cho SK, Sestak A, Namjou B, Kang C, Bae SC. Interferon-gamma gene polymorphisms associated with susceptibility to systemic lupus erythematosus. Ann Rheum Dis. 2010 Jun;69(6):1247-50. PubMed PMID: 19919944. Epub 2009/11/19. eng.

45. Ronnblom L. The type I interferon system in the etiopathogenesis of autoimmune diseases. Ups J Med Sci. 2011 Nov;116(4):227-37. PubMed PMID: 22066971. Pubmed Central PMCID: PMC3207297. Epub 2011/11/10. eng.

46. Dienz O, Eaton SM, Bond JP, Neveu W, Moquin D, Noubade R, et al. The induction of antibody production by IL-6 is indirectly mediated by IL-21 produced by CD4+ T cells. J Exp Med. 2009 Jan 16;206(1):69-78. PubMed PMID: 19139170. Pubmed Central PMCID: PMC2626667. Epub 2009/01/14. eng.

47. Naka T, Nishimoto N, Kishimoto T. The paradigm of IL-6: from basic science to medicine. Arthritis Res. 2002;4 Suppl 3:S233-42. PubMed PMID: 12110143. Pubmed Central PMCID: PMC3240141. Epub 2002/07/12. eng.

48. Acosta-Rodriguez EV, Napolitani G, Lanzavecchia A, Sallusto F. Interleukins 1beta and 6 but not transforming growth factor-beta are essential for the differentiation of interleukin 17-producing human T helper cells. Nat Immunol. 2007 Sep;8(9):942-9. PubMed PMID: 17676045. Epub 2007/08/07. eng.

49. Crispin JC, Oukka M, Bayliss G, Cohen RA, Van Beek CA, Stillman IE, et al. Expanded double negative T cells in patients with systemic lupus erythematosus produce IL-17 and infiltrate the kidneys. J Immunol. 2008 Dec 15;181(12):8761-6. PubMed PMID: 19050297. Pubmed Central PMCID: PMC2596652. Epub 2008/12/04. eng.

50. Dolff S, Quandt D, Wilde B, Feldkamp T, Hua F, Cai X, et al. Increased expression of costimulatory markers CD134 and CD80 on interleukin-17 producing T cells in patients with systemic lupus erythematosus. Arthritis Res Ther. 2010;12(4):R150. PubMed PMID: 20653937. Pubmed Central PMCID: PMC2945048. Epub 2010/07/27. eng.

51. Yang J, Chu Y, Yang X, Gao D, Zhu L, Wan L, et al. Th17 and natural Treg cell population dynamics in systemic lupus erythematosus. Arthritis and rheumatism. 2009 May;60(5):1472-83. PubMed PMID: 19404966. Epub 2009/05/01. eng.

52. Kastelein RA, Hunter CA, Cua DJ. Discovery and biology of IL-23 and IL-27: related but functionally distinct regulators of inflammation. Annu Rev Immunol. 2007;25:221-42. PubMed PMID: 17291186. Epub 2007/02/13. eng.

53. Edelbauer M, Kshirsagar S, Riedl M, Billing H, Tonshoff B, Haffner D, et al. Activity of childhood lupus nephritis is linked to altered T cell and cytokine homeostasis. J Clin Immunol. 2012 Jun;32(3):477-87. PubMed PMID: 22228566. Epub 2012/01/10. eng.

54. Mok MY, Wu HJ, Lo Y, Lau CS. The relation of interleukin 17 (IL-17) and IL-23 to Th1/Th2 cytokines and disease activity in systemic lupus erythematosus. J Rheumatol. 2010 Oct;37(10):2046-52. PubMed PMID: 20682672. Epub 2010/08/05. eng.

55. Rana A, Minz RW, Aggarwal R, Anand S, Pasricha N, Singh S. Gene expression of cytokines (TNF-alpha, IFN-gamma), serum profiles of IL-17 and IL-23 in paediatric systemic lupus erythematosus. Lupus. 2012 Sep;21(10):1105-12. PubMed PMID: 22759859. Epub 2012/07/05. eng.

56. Kyttaris VC, Zhang Z, Kuchroo VK, Oukka M, Tsokos GC. Cutting edge: IL-23 receptor deficiency prevents the development of lupus nephritis in C57BL/6-lpr/lpr mice. J Immunol. 2010 May 1;184(9):4605-9. PubMed PMID: 20308633. Pubmed Central PMCID: PMC2926666. Epub 2010/03/24. eng.

57. Tsokos GC. Systemic lupus erythematosus. N Engl J Med. 2011 Dec 1;365(22):2110-21. PubMed PMID: 22129255. Epub 2011/12/02. eng.

58. Crotty S. Follicular helper CD4 T cells (TFH). Annu Rev Immunol. 2011;29:621-63. PubMed PMID: 21314428. Epub 2011/02/15. eng.

59. Waite JC, Skokos D. Th17 response and inflammatory autoimmune diseases. Int J Inflam. 2012;2012:819467. PubMed PMID: 22229105. Pubmed Central PMCID: PMC3249891. Epub 2012/01/10. eng.

60. Doreau A, Belot A, Bastid J, Riche B, Trescol-Biemont MC, Ranchin B, et al. Interleukin 17 acts in synergy with B cell-activating factor to influence B cell biology and the pathophysiology of systemic lupus erythematosus. Nat Immunol. 2009 Jul;10(7):778-85. PubMed PMID: 19483719. Epub 2009/06/02. eng.

61. Shah K, Lee WW, Lee SH, Kim SH, Kang SW, Craft J, et al. Dysregulated balance of Th17 and Th1 cells in systemic lupus erythematosus. Arthritis Res Ther. 2010;12(2):R53. PubMed PMID: 20334681. Pubmed Central PMCID: PMC2888202. Epub 2010/03/26. eng.

62. Sewgobind VD, Quaedackers ME, van der Laan LJ, Kraaijeveld R, Korevaar SS, Chan G, et al. The Jak inhibitor CP-690,550 preserves the function of CD4CD25FoxP3 regulatory T cells and inhibits effector T cells. Am J Transplant. 2010 Aug;10(8):1785-95. PubMed PMID: 20626385. Epub 2010/07/16. eng.

63. Crispin JC, Martinez A, Alcocer-Varela J. Quantification of regulatory T cells in patients with systemic lupus erythematosus. J Autoimmun. 2003 Nov;21(3):273-6. PubMed PMID: 14599852. Epub 2003/11/06. eng.

64. Lee JH, Wang LC, Lin YT, Yang YH, Lin DT, Chiang BL. Inverse correlation between CD4+ regulatory T-cell population and autoantibody levels in paediatric patients with systemic lupus erythematosus. Immunology. 2006 Feb;117(2):280-6. PubMed PMID: 16423064. Pubmed Central PMCID: PMC1782210. Epub 2006/01/21. eng.

65. Lourenco EV, La Cava A. Natural regulatory T cells in autoimmunity. Autoimmunity. 2011 Feb;44(1):33-42. PubMed PMID: 21091291. Pubmed Central PMCID: PMC3057884. Epub 2010/11/26. eng.

66. La Cava A. T-regulatory cells in systemic lupus erythematosus. Lupus. 2008 May;17(5):421-5. PubMed PMID: 18490420. Epub 2008/05/21. eng.

67. La Cava A, Ebling FM, Hahn BH. Ig-reactive CD4+CD25+ T cells from tolerized (New Zealand Black x New Zealand White)F1 mice suppress in vitro production of antibodies to DNA. J Immunol. 2004 Sep 1;173(5):3542-8. PubMed PMID: 15322219. Epub 2004/08/24. eng.

68. McMahon M, Grossman J, Skaggs B, Fitzgerald J, Sahakian L, Ragavendra N, et al. Dysfunctional proinflammatory high-density lipoproteins confer increased risk of atherosclerosis in women with systemic lupus erythematosus. Arthritis Rheum. 2009 Aug;60(8):2428-37. PubMed PMID: 19644959. Pubmed Central PMCID: PMC2753974. Epub 2009/08/01. eng.

69. E V, Yalavarthi S Fau - Berthier CC, Berthier Cc Fau - Hodgin JB, Hodgin Jb Fau - Khandpur R, Khandpur R Fau - Lin AM, Lin Am Fau - Rubin CJ, et al. - Netting neutrophils induce endothelial damage, infiltrate tissues, and expose immunostimulatory molecules in systemic lupus erythematosus. J Immunol. 2011;187(1):538-52. - eng.

22 APPENDICES:

22.1 Schedule of Events

Study Procedures and Visits

Procedures	SCR 30 days	D1	D7 t/call	D14	D21 t/call	D28	D35 t/call	D42	D49 t/call	D56 -Last dosing day	D84 Final f/u	UNSCH visit for AE or other reason
Informed Consent	X											
Review of inclusion and exclusion criteria,	X	X										
vital sign measurements	X	X		X		X		X		X	X	X
Medical history	X											
Randomization	X											
Presenting Symptoms-Abbreviated Medical History		X		X		X		X		X	X	X
Physical examination	X	X		X		X		X		X	X	X
EKG,	X									X	X	X
Concomitant medications review.	X	X	X	X	X	X	X	X	X	X	X	X
Study Drug Administration<br>D1-D56, Dose BID. 1st dose at NIH on D1		X	X	X	X	X	X	X	X	X		
Drug Accountability-Dispensing Study Drug (35 days' supply)		x				x						
Compliance with study drug- Returning bottle.						X				X		
Adverse event review (assessed by reviewing medical hx, Physical exam, lab results)<br>from D1-D84.		X	X	X	X	X	X	X	X	X	X	X
Screening serologies for hepatitis B, hepatitis C and HIV,	X											
Screening Tuberculosis using the Quantiferon Gold test.	X											
BK virus quantitative PCR- urine and blood	X					X				X	X	
**Serologies:** antinuclear antibodies, anti-ENA panel (anti-RNP, anti-SmRNP, anti-SSA, anti-SSB **)** anti-dsDNA antibodies, anticardiolipin antibodies, lupus anticoagulant, anti-beta2-glycoprotein antibodies, C3 complement, C4 complement, Quantitative immunoglobulin IgA, IgG, and IgM	X											
anti-dsDNA antibodies, C3 complement, C4 complement, Quantitative immunoglobulin IgA, IgG, and IgM		x		x		x		x		x	x	x
**complete blood count** with differential	X	X		X		X		X		X	X	X
**serum chemistry panel (**creatinine, glucose, urea nitrogen, total bilirubin, alanine aminotransferase, aspartate aminotransferase, alkaline phosphatase)	X	X		X		X		X		X	X	X
**Pregnancy test** (serum or urine; for females with reproductive potential only)	X	X		X		X		X		X	X	X
**Urinalysis and Random urine Protein/Creatinine ratio**	X	X		x		X		x		X	X	X
**Lipid Panel** (low density lipoprotein (LDL), high density lipoprotein (HDL), triglycerides (TG) and total cholesterol.	X	X								X	X	X
**Lipoprotein Profile**	X	X								X	X	X
**Lymphocyte pheno-TBNK**		X								X	X	X
**Inflammatory markers:**<br>(hs-CRP, ESR)		X								X	X	X
Assessment of biologic effect(s)		*x*								*x*		
Assessment of the effect of STAT4 risk alleles on drug efficacy.		*x*								*x*		
Assessment of durability of the clinical and biologic effects.										*x*	*x*	
Mechanistic studies: (intracellular signaling molecules, cytokine expression, circulating immune cell types and autoreactive B cells.) All samples can be run simultaneously for each assay at the end of the study.		x								x	x	
Assessment of lupus activity (SLEDAI-2K , BILAG 2004, PGA)	X											
6. Patient questionnaires<br>1. SF-36,<br>2. Multidimensional Assessment of Fatigue questionnaire and<br>3. Patient Global Assessment Scale. P.16, 31,39		X		X		X		X		X	X	X
**Assessment of lupus disease activity**<br>1. SLEDAI-2K<br>2. Physician Global Assessment (PGA)<br>3. Joint count: A 28 tender and swollen joint count (DAS-28)<br>4. Cutaneous Lupus Erythematosus Disease Area and Severity Index (CLASI)		X		X		X		X		X	X	X
5. BILAG 2004	X					X				X	X	X
Vascular Function Studies		X								X	X	
Research labs		X		X						X	X	X

22.2 American College of Rheumatology Revised Classification Criteria for Systemic Lupus Erythematosus

Criteria	Definition
Malar rash	Fixed erythema, flat or raised, over the malar eminences, tending to spare the nasolabial folds
Discoid rash	Erythematous raised patches with adherent keratotic scaling and follicular plugging; atrophic scarring occurs in older lesions
Photosensitivity	Skin rash as a result of unusual reaction to sunlight, by patient history or physician observation
Oral ulcers	Oral or nasopharyngeal ulceration, usually painless, observed by a physician
Arthritis	Nonerosive arthritis involving two or more peripheral joints, characterized by tenderness, swelling, or effusion
Serositis	a. Pleuritis—convincing history of pleuritic pain or rub heard by a physician or evidence of pleural effusion or<br>b. Pericarditis—documented by ECG or rub or evidence of pericardial effusion
Renal disorder	a. Persistent proteinuria >0.5 g/day >3+ if quantitation is not performed or<br>b. Cellular casts—may be red blood cell, hemoglobin, granular tubular, or mixed
Neurologic disorder	a. Seizures—in the absence of offending drugs or known metabolic derangements (e.g., uremia, acidosis, or electrolyte imbalance) or<br>b. Psychosis—in the absence of offending drugs or known metabolic derangements (e.g., uremia, acidosis, or electrolyte imbalance)
Hematologic disorder	a. Hemolytic anemia with reticulocytosis, or<br>b. Leukopenia—$<4000/mm^3$, or<br>c. Lymphopenia—$<1500/mm^3$, or<br>d. Thrombocytopenia—$<100,000/mm^3$ in the absence of offending drugs
Immunologic disorder	a. Anti-DNA—antibody to native DNA in abnormal titer, or<br>b. Anti-Sm—presence of antibody to Sm nuclear antigen, or<br>c. Positive finding of antiphospholipid antibodies based on (1) abnormal serum concentration of IgG or IgM anticardiolipin antibodies, (2) positive test result for lupus anticoagulant using a standard method, or (3) false-positive serologic test for syphilis

Criteria	Definition
	known to be positive for at least 6 mo and confirmed by Treponema pallidum immobilization or fluorescent treponemal antibody absorption test
ANA	Abnormal titer of ANA by immunofluorescence or equivalent assay at any point in time and in the absence of drugs known to be associated with drug-induced lupus syndrome

Adapted from Hochberg MC: Updating the American College of Rheumatology revised criteria for the classification of systemic lupus erythematosus. Arthritis Rheum 40:1725, 1997.

22.3 SLEDAI 2K

SLEDAI-2K: DATA COLLECTION SHEET

SLEDAI 2K Weight	SLEDAI 2K SCORE	Descriptor	Definition
8	________	Seizure	Recent onset, exclude metabolic, infectious or drug causes.
8	________	Psychosis	Altered ability to function in normal activity due to severe disturbance in the perception of reality. Include hallucinations, incoherence, marked loose associations, impoverished thought content, marked illogical thinking, bizarre, disorganized, or catatonic behavior. Exclude uremia and drug causes
8	________	Organic brain syndrome	Altered mental function with impaired orientation, memory, or other intellectual function, with rapid onset and fluctuating clinical features, inability to sustain attention to environment, plus at least 2 of the following: perceptual disturbance, incoherent speech, insomnia or daytime drowsiness, or increased or decreased psychomotor activity. Exclude metabolic, infectious, or drug causes.
8	________	Visual disturbance	Retinal changes of SLE. Include cytoid bodies, retinal hemorrhages, serous exudate or hemorrhages in the choroid, or optic neuritis. Exclude hypertension, infection, or drug causes.
8	________	Cranial nerve disorder	New onset of sensory or motor neuropathy involving cranial nerves.
8	________	Lupus headache	Severe, persistent headache; may be migrainous, but must be nonresponsive to narcotic analgesia.
8	________	CVA	New onset of cerebrovascular accident(s). Exclude arteriosclerosis.

8	_______	Vasculitis	Ulceration, gangrene, tender finger nodules, periungual infarction, splinter hemorrhages, or biopsy or angiogram proof of vasculitis.
4	_______	Arthritis	$\geq$ 2 joints with pain and signs of inflammation (i.e., tenderness, swelling or effusion).
4	_______	Myositis	Proximal muscle aching/weakness, associated with elevated creatine phosphokinase/aldolase or electromyogram changes or a biopsy showing myositis.
4	_______	Urinary casts	Heme-granular or red blood cell casts.
4	_______	Hematuria	>5 red blood cells/high power field. Exclude stone, infection or other cause.
4	_______	Proteinuria	>0.5 gram/24 hours
4	_______	Pyuria	>5 white blood cells/high power field. Exclude infection.
2	_______	Rash	Inflammatory type rash.
2	_______	Alopecia	Abnormal, patchy or diffuse loss of hair.
2	_______	Mucosal ulcers	Oral or nasal ulcerations.
2	_______	Pleurisy	Pleuritic chest pain with pleural rub or effusion, or pleural thickening.
2	_______ P	Pericarditis	Pericardial pain with at least 1 of the following: rub, effusion, or electrocardiogram or echocardiogram confirmation.
2	_______	Low complement	Decrease in CH50, C3, or C4 below the lower limit of normal for testing laboratory
2	_______	Increased DNA binding	Increased DNA binding by Farr assay above normal range for testing laboratory.
1	_______	Fever	>38° C. Exclude infectious cause.
1	_______	Thrombocytopenia	<100,000 platelets / x10^{9}/L, exclude drug causes.
1	_______	Leukopenia	< 3,000 white blood cells / x10^{9}/L, exclude drug causes.

TOTAL SCORE: ______________

22.4 BILAG 2004:

BILAG-2004 INDEX **Centre:** **Date:** **Initials/Hosp No:**

♦ **Only record manifestations/items <u>due to SLE Disease Activity</u>**
♦ **Assessment refers to <u>manifestations occurring in the last 4 weeks (compared with the previous 4 weeks)</u>**
♦ **TO BE USED WITH THE GLOSSARY**

Record: **ND** **Not Done**
0 **Not present**
1 **Improving**
2 **Same**
3 **Worse**
4 **New**
Yes/No OR Value (where indicated)
***Y/N Confirm this is <u>due to SLE activity</u> (Yes/No)**

<u>CONSTITUTIONAL</u>
1. Pyrexia - documented > 37.5ºC ( )
2. Weight loss - unintentional > 5% ( )
3. Lymphadenopathy/splenomegaly ( )
4. Anorexia ( )

<u>MUCOCUTANEOUS</u>
5. Skin eruption - severe ( )
6. Skin eruption - mild ( )
7. Angio-oedema - severe ( )
8. Angio-oedema - mild ( )
9. Mucosal ulceration - severe ( )
10. Mucosal ulceration - mild ( )
11. Panniculitis/Bullous lupus - severe ( )
12. Panniculitis/Bullous lupus - mild ( )
13. Major cutaneous vasculitis/thrombosis ( )
14. Digital infarcts or nodular vasculitis ( )
15. Alopecia - severe ( )
16. Alopecia - mild ( )
17. Peri-ungual erythema/chilblains ( )
18. Splinter haemorrhages ( )

<u>NEUROPSYCHIATRIC</u>
19. Aseptic meningitis ( )
20. Cerebral vasculitis ( )
21. Demyelinating syndrome ( )
22. Myelopathy ( )
23. Acute confusional state ( )
24. Psychosis ( )
25. Acute inflammatory demyelinating polyradiculoneuropathy ( )
26. Mononeuropathy (single/multiplex) ( )
27. Cranial neuropathy ( )
28. Plexopathy ( )
29. Polyneuropathy ( )
30. Seizure disorder ( )
31. Status epilepticus ( )
32. Cerebrovascular disease (not due to vasculitis) ( )
33. Cognitive dysfunction ( )
34. Movement disorder ( )
35. Autonomic disorder ( )
36. Cerebellar ataxia (isolated) ( )
37. Lupus headache - severe unremitting ( )
38. Headache from IC hypertension ( )

<u>MUSCULOSKELETAL</u>
39. Myositis - severe ( )
40. Myositis - mild ( )
41. Arthritis (severe) ( )
42. Arthritis (moderate)/Tendonitis/Tenosynovitis ( )
43. Arthritis (mild)/Arthralgia/Myalgia ( )

**Weight (kg):**	**Serum urea (mmol/l):**
**African ancestry: Yes/No**	**Serum albumin (g/l):**

<u>CARDIORESPIRATORY</u>
44. Myocarditis - mild ( )
45. Myocarditis/Endocarditis + Cardiac failure ( )
46. Arrhythmia ( )
47. New valvular dysfunction ( )
48. Pleurisy/Pericarditis ( )
49. Cardiac tamponade ( )
50. Pleural effusion with dyspnoea ( )
51. Pulmonary haemorrhage/vasculitis ( )
52. Interstitial alveolitis/pneumonitis ( )
53. Shrinking lung syndrome ( )
54. Aortitis ( )
55. Coronary vasculitis ( )

<u>GASTROINTESTINAL</u>
56. Lupus peritonitis ( )
57. Abdominal serositis or ascites ( )
58. Lupus enteritis/colitis ( )
59. Malabsorption ( )
60. Protein losing enteropathy ( )
61. Intestinal pseudo-obstruction ( )
62. Lupus hepatitis ( )
63. Acute lupus cholecystitis ( )
64. Acute lupus pancreatitis ( )

<u>OPHTHALMIC</u>
65. Orbital inflammation/myositis/proptosis ( )
66. Keratitis - severe ( )
67. Keratitis - mild ( )
68. Anterior uveitis ( )
69. Posterior uveitis/retinal vasculitis - severe ( )
70. Posterior uveitis/retinal vasculitis - mild ( )
71. Episcleritis ( )
72. Scleritis - severe ( )
73. Scleritis - mild ( )
74. Retinal/choroidal vaso-occlusive disease ( )
75. Isolated cotton-wool spots (cytoid bodies) ( )
76. Optic neuritis ( )
77. Anterior ischaemic optic neuropathy ( )

<u>RENAL</u>
78. Systolic blood pressure (mm Hg) value ( ) Y/N*
79. Diastolic blood pressure (mm Hg) value ( ) Y/N*
80. Accelerated hypertension Yes/No ( )
81. Urine dipstick protein (+=1, ++=2, +++=3) ( ) Y/N*
82. Urine albumin-creatinine ratio mg/mmol ( ) Y/N*
83. Urine protein-creatinine ratio mg/mmol ( ) Y/N*
84. 24 hour urine protein (g) value ( ) Y/N*
85. Nephrotic syndrome Yes/No ( )
86. Creatinine (plasma/serum) µmol/l ( ) Y/N*
87. GFR (calculated) ml/min/1.73 m^2 ( ) Y/N*
88. Active urinary sediment Yes/No ( )
89. Active nephritis Yes/No ( )

<u>HAEMATOLOGICAL</u>
90. Haemoglobin (g/dl) value ( ) Y/N*
91. Total white cell count (x 10^9/l) value ( ) Y/N*
92. Neutrophils (x 10^9/l) value ( ) Y/N*
93. Lymphocytes (x 10^9/l) value ( ) Y/N*
94. Platelets (x 10^9/l) value ( ) Y/N*
95. TTP ( )
96. Evidence of active haemolysis Yes/No ( )
97. Coombs' test positive (isolated) Yes/No ( )

Revision: 1/Sep/2009

22.5 Disease Activity Score-28 (DAS-28):

DAS28 form

Patient name……………………………………………. Date of Birth ……-……-…….

Observer name………………………………………… Date ……-……-……

	LEFT Swollen	tender	RIGHT Swollen	Tender
Shoulder				
Elbow				
Wrist				
MCP 1				
2				
3				
4				
5				
PIP 1				
2				
3				
4				
5				
Knee				
Subtotal				
Total	swollen		Tender	

No disease activity high disease activity

Swollen (0-28)	
Tender (0-28)	
ESR	
VAS disease activity (0-100mm)	

DAS28 = 0.56*√(t28) + 0.28*√(sw28) + 0.70*Ln(ESR) + 0.014*VAS

22.6 The Cutaneous Lupus Erythematosus Disease Area and Severity Index (CLASI):

Cutaneous LE Disease Area and Severity Index (CLASI)

Select the score in each anatomical location that describes the most severely affected cutaneous lupus-associated lesion

Extent

	activity		damage		
Anatomical Location	Erythema	Scale/ Hypertrophy	Dyspigmentation	Scarring/ Atrophy/ Panniculitis	Anatomical Location
	0-absent<br>1-pink; faint erythema<br>2- red;<br>3-dark red; purple/violaceous/ crusted/ hemorrhagic	0-absent;<br>1-scale<br>2-verrucous/ hypertrophic	0-absent,<br>1-dyspigmentaton	0 – absent<br>1 – scarring<br>2 – severely atrophic scarring or panniculitis	
Scalp				See below	Scalp
Ears					Ears
Nose (incl. malar area)					Nose (incl. malar area)
Rest of the face					Rest of the face
V-area neck (frontal)					V-area neck (frontal)
Post. Neck &/or shoulders					Post. Neck &/or shoulders
Chest					Chest
Abdomen					Abdomen
Back, buttocks					Back, buttocks
Arms					Arms
Hands					Hands
Legs					Legs
Feet					Feet

Mucous membrane

Mucous membrane lesions (examine if patient confirms involvement)	
0-absent;<br>1-lesion or ulceration	

Dyspigmentation

Report duration of dyspigmentation after active lesions have resolved (verbal report by patient – tick appropriate box)
☐ Dyspigmentation usually lasts less than 12 months (dyspigmentation score above remains)<br>☐ Dyspigmentation usually lasts at least 12 months (dyspigmentation score is doubled)

Alopecia

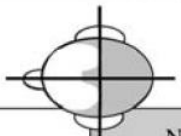

Recent Hair loss (within the last 30 days / as reported by patient)	
1-Yes<br>0-No	

NB: if scarring and non-scarring aspects seem to coexist in one lesion, please score both

Divide the scalp into four quadrants as shown. The dividing line between right and left is the midline. The dividing line between frontal and occipital is the line connecting the highest points of the ear lobe. A quadrant is considered affected if there is a lesion within the quadrant.

Alopecia (clinically not obviously scarred)		Scarring of the scalp (judged clinically)	
0-absent<br>1-diffuse; non-inflammatory<br>2-focal or patchy in one quadrant;<br>3-focal or patchy in more than one quadrant		0- absent<br>3- in one quadrant<br>4- two quadrants<br>5- three quadrants<br>6- affects the whole skull	

Total Activity Score<br>(For the activity score please add up the scores of the left side i.e. for Erythema, Scale/Hypertrophy, Mucous membrane involvement and Alopecia)<br>©		Total Damage Score<br>(For the damage score, please add up the scores of the right side, i.e. for Dyspigmentation, Scarring/Atrophy/Panniculitis and Scarring of the Scalp)	

22.7 36-item Short Form Survey:

Medical Outcomes Study: 36-Item Short Form Survey Instrument

1. In general, would you say your health is:	
Excellent	1
Very good	2
Good	3
Fair	4
Poor	5

2. Compared to one year ago, how would your rate your health in general now?	
Much better now than one year ago	1
Somewhat better now than one year ago	2
About the same	3
Somewhat worse now than one year ago	4
Much worse now than one year ago	5

The following items are about activities you might do during a typical day. Does your health now limit you in these activities? If so, how much?

(Circle One Number on Each Line)

	Yes, Limited a Lot	Yes, Limited a Little	No, Not limited at All
3. Vigorous activities, such as running, lifting heavy objects, participating in strenuous sports	[1]	[2]	[3]
4. Moderate activities, such as moving a table, pushing a vacuum cleaner, bowling, or playing golf	[1]	[2]	[3]

5. Lifting or carrying groceries	[1]	[2]	[3]
6. Climbing several flights of stairs	[1]	[2]	[3]
7. Climbing one flight of stairs	[1]	[2]	[3]
8. Bending, kneeling, or stooping	[1]	[2]	[3]
9. Walking more than a mile	[1]	[2]	[3]
10. Walking several blocks	[1]	[2]	[3]
11. Walking one block	[1]	[2]	[3]
12. Bathing or dressing yourself	[1]	[2]	[3]

During the past 4 weeks, have you had any of the following problems with your work or other regular daily activities as a result of your physical health?

(Circle One Number on Each Line)

	Yes	No
13. Cut down the amount of time you spent on work or other activities	1	2
14. Accomplished less than you would like	1	2
15. Were limited in the kind of work or other activities	1	2
16. Had difficulty performing the work or other activities (for example, it took extra effort)	1	2

During the past 4 weeks, have you had any of the following problems with your work or other regular daily activities as a result of any emotional problems (such as feeling depressed or anxious)?

(Circle One Number on Each Line)

	Yes	No
17. Cut down the amount of time you spent on work or other activities	1	2
18. Accomplished less than you would like	1	2
19. Didn't do work or other activities as carefully as usual	1	2

20. During the past 4 weeks, to what extent has your physical health or emotional problems interfered with your normal social activities with family, friends, neighbors, or groups?

(Circle One Number)

Not at all 1

Slightly 2

Moderately 3

Quite a bit 4

Extremely 5

21. How much bodily pain have you had during the past 4 weeks?

(Circle One Number)

None 1

Very mild 2

Mild 3

Moderate 4

Severe 5

Very severe 6

22. During the past 4 weeks, how much did pain interfere with your normal work (including both work outside the home and housework)?

(Circle One Number)

Not at all 1

A little bit 2

Moderately 3

Quite a bit 4

Extremely 5

These questions are about how you feel and how things have been with you during the past 4 weeks. For each question, please give the one answer that comes closest to the way you have been feeling.

How much of the time during the past 4 weeks . . .

(Circle One Number on Each Line)

	All of the Time	Most of the Time	A Good Bit of the Time	Some of the Time	A Little of the Time	None of the Time
23. Did you feel full of pep?	1	2	3	4	5	6
24. Have you been a very nervous person?	1	2	3	4	5	6
25. Have you felt so down in the dumps that nothing could cheer you up?	1	2	3	4	5	6
26. Have you felt calm and peaceful?	1	2	3	4	5	6
27. Did you have a lot of energy?	1	2	3	4	5	6
28. Have you felt downhearted and blue?	1	2	3	4	5	6
29. Did you feel worn out?	1	2	3	4	5	6
30. Have you been a happy person?	1	2	3	4	5	6
31. Did you feel tired?	1	2	3	4	5	6

32. During the past 4 weeks, how much of the time has your physical health or emotional problems interfered with your social activities (like visiting with friends, relatives, etc.)?

(Circle One Number)

All of the time 1

Most of the time 2

Some of the time 3

A little of the time 4

None of the time 5

How TRUE or FALSE is <u>each</u> of the following statements for you.

(Circle One Number on Each Line)

	Definitely True	Mostly True	Don't Know	Mostly False	Definitely False
33. I seem to get sick a little easier than other people	1	2	3	4	5
34. I am as healthy as anybody I know	1	2	3	4	5
35. I expect my health to get worse	1	2	3	4	5
36. My health is excellent	1	2	3	4	5

22.8 Multidimensional Assessment of Fatigue:

MULTIDIMENSIONAL ASSESSMENT OF FATIGUE (MAF) SCALE

Instructions: These questions are about fatigue and the effect of fatigue on your activities.

For each of the following questions, circle the number that most closely indicates how you have been feeling during the past week.

For example, suppose you really like to sleep late in the mornings. You would probably circle the number closer to the "a great deal" end of the line. This is where I put it:

Example: To what degree do you usually like to sleep late in the mornings?

1 2 3 4 [illegible] 6 7 8 9 1

Not at all — A great deal

Now please complete the following items based on the past week.

---

1. To what degree have you experienced fatigue?

1 2 3 4 5 6 7 8 9 1

Not at all — A great deal

If no fatigue, stop here.

2. How severe is the fatigue which you have been experiencing?

1 2 3 4 5 6 7 8 9 1

Mild — Severe

3. To what degree has fatigue caused you distress?

1 2 3 4 5 6 7 8 9 1

No distress — A great deal of distress

MULTIDIMENSIONAL ASSESSMENT OF FATIGUE (MAF) SCALE (Continued)

Circle the number that most closely indicates to what degree fatigue has interfered with your ability to do the following activities in the past week. For activities you don't do, for reasons other than fatigue (e.g. you don't work because you are retired), check the box.In the past week, to what degree has fatigue interfered with your ability to:

(NOTE: Check box to the left of each number if you don't do activity)

☐ 4. Do household chores

1 2 3 4 5 6 7 8 9 1

Not at all A great deal

☐ 5. Cook

1 2 3 4 5 6 7 8 9 1

Not at all A great deal

☐ 6. Bathe or wash

1 2 3 4 5 6 7 8 9 1

Not at all A great deal

☐ 7. Dress

1 2 3 4 5 6 7 8 9 1

Not at all A great deal

☐ 8. Work

1 2 3 4 5 6 7 8 9 1

Not at all A great deal

☐ 9. Visit or socialize with friends or family

1 2 3 4 5 6 7 8 9 1

Not at all A great deal

MULTIDIMENSIONAL ASSESSMENT OF FATIGUE (MAF) SCALE (Continued)

(NOTE: Check box to the left of each number if you don't do activity)

☐ 10. Engage in sexual activity

1 2 3 4 5 6 7 8 9 1

Not at all A great deal

☐ 11. Engage in leisure and recreational activities

1 2 3 4 5 6 7 8 9 1

Not at all A great deal

☐ 12. Shop and do errands

1 2 3 4 5 6 7 8 9 1

Not at all A great deal

☐ 13. Walk

1 2 3 4 5 6 7 8 9 1

Not at all A great deal

☐ 14. Exercise, other than walking

1 2 3 4 5 6 7 8 9 1

Not at all A great deal

15. Over the past week, how often have you been fatigued?

- 4 Every day
- 3 Most, but not all days
- 2 Occasionally, but not most days
- 1 Hardly any days

16. To what degree has your fatigue changed during the past week?

- 4 Increased
- 3 Fatigue has gone up and down
- 2 Stayed the same
- 1 Decreased

22.9 NIH Clinical Center guidelines for the management of allergic reactions:

ANAPHYLAXIS TREATMENT MEDICATION DOSE GUIDELINES – PRIMARY THERAPY			
**DRUG**	**CONCENTRATION**	**ADULT DOSE [1]**	**PEDIATRIC DOSE [1,2,3]**
**First-line Treatment**			
**EPINEPHRINE AUTO-INJECTOR** (EpiPen)	1:1,000 (0.3 MG Fixed Dose Inj)	0.3 mg<br>> 25 Kg [3]<br>**IM** [4]<br>MAY REPEAT q 5 to 15 mins	0.3 mg<br>> 25 Kg [3]<br>**IM** [4]<br>MAY REPEAT q 5 to 15 mins
**EPINEPHRINE AUTO-INJECTOR Jr** (EpiPen Jr.)	1:2,000 (0.15 MG Fixed Dose Inj)	N/A	0.15 mg<br>10 to 25 Kg[3]<br>**IM** [3] or SQ<br>MAY REPEAT q 5 to 15 mins
**EPINEPHRINE AMPULE**	1:1,000 (1 mg/mL)	0.2 to 0.5 mg per dose<br>**IM** [4] or Subcutaneous<br>MAY REPEAT q 5 to 15 mins	0.01 mg/Kg per dose<br>**IM** [4] or Subcutaneous<br>MAY REPEAT q 5 to 15 mins<br><br>MAX SINGLE DOSE 0.5 mg (0.5 mL)

1. The diagnosis and management of anaphylaxis practice parameter: 2010 Update. J Allergy Clin Immunol 2010;126: 477-80.
2. The Harriet Lane Handbook, 18th Edition
3. This differs from the package insert recommendation as per Guidelines for the Diagnosis and Management of Food Allergy in the United States: Report of the NIAID-Sponsored Expert Panel. J Allergy Clin Immunol 2010;126: S1 – S58.
4. The intramuscular (IM) route is preferred. Epinephrine absorption in adults: Intramuscular versus subcutaneous injection. J Allergy Clin Immunol 2001;108:871-3.

SEE REVERSE SIDE FOR ADJUNCTIVE THERAPY➔

Approved by P&T Committee on February 24, 2011. Revised on XX/XX/2011

ANAPHYLAXIS TREATMENT MEDICATION DOSE GUIDELINES – PRIMARY THERAPY			
**DRUG**	**CONCENTRATION**	**ADULT DOSE (1)**	**PEDIATRIC DOSE (1,2,3)**
**First-line Treatment**			
**EPINEPHRINE AUTO-INJECTOR** (EpiPen)	1:1,000 (0.3 MG Fixed Dose Inj)	0.3 mg > 25 Kg (3) **IM** (4) MAY REPEAT q 5 to 15 mins	0.3 mg > 25 Kg (3) **IM** (4) MAY REPEAT q 5 to 15 mins
**EPINEPHRINE AUTO-INJECTOR Jr** (EpiPen Jr.)	1:2,000 (0.15 MG Fixed Dose Inj)	N/A	0.15 mg 10 to 25 Kg(3) **IM** (3) or SQ MAY REPEAT q 5 to 15 mins
**EPINEPHRINE AMPULE**	1:1,000 (1 mg/mL)	0.2 to 0.5 mg per dose **IM** (4) or Subcutaneous MAY REPEAT q 5 to 15 mins	0.01 mg/Kg per dose **IM** (4) or Subcutaneous MAY REPEAT q 5 to 15 mins<br><br>MAX SINGLE DOSE 0.5 mg (0.5 mL)

1. The diagnosis and management of anaphylaxis practice parameter: 2010 Update. J Allergy Clin Immunol 2010;126: 477-80.
2. The Harriet Lane Handbook, 18th Edition
3. This differs from the package insert recommendation as per Guidelines for the Diagnosis and Management of Food Allergy in the United States: Report of the NIAID-Sponsored Expert Panel. J Allergy Clin Immunol 2010;126: S1 – S58.
4. The intramuscular (IM) route is preferred. Epinephrine absorption in adults: Intramuscular versus subcutaneous injection. J Allergy Clin Immunol 2001;108:871-3.

SEE REVERSE SIDE FOR ADJUNCTIVE THERAPY➔

Approved by P&T Committee on February 24, 2011. Revised on XX/XX/2011

22.10 **XELJANZ (tofacitinib) patient prescribing information:**

Xeljanz prescribing information

22.11 **NCI Common Toxicity Criteria, Version 4.0**:

http://evs.nci.nih.gov/ftp1/CTCAE/CTCAE_4.03_2010-06-14_QuickReference_5x7.pdf

22.12 Physician Global Assessment (PGA):

Mark an X on the line below to indicate disease activity (independent of patient's self assessment):

Very good |————————————————————| Very bad

22.13 Consent Forms: Attached as a separate file.

CLINICAL RESEARCH PROTOCOL

NATIONAL INSTITUTE OF ARTHRITIS AND MUSCULOSKELETAL AND SKIN DISEASES

PROTOCOL NUMBER: 15-AR-0185
PROTOCOL VERSION: CR/2019 IRB Approved Date: 04/23/2019

PROTOCOL TITLE: Safety of tofacitinib, an oral Janus kinase inhibitor, in Systemic Lupus Erythematosus; a Phase Ib clinical trial and associated mechanistic studies

SHORT TITLE: JAK-IN-LUPUS

IDENTIFYING WORDS: JAK-IN-LUPUS

Principal Investigator:

Name, Degree	Branch/Institute	Bldg/Rm	Phone	E-mail
Sarfaraz Hasni, M.D.	SAB/NIAMS	10/3-2340	301-451-1599	hasnisa@mail.nih.gov

Associate Investigators:

Name, Degree	Branch/Institute	Bldg/Rm	Phone	E-mail
Mariana Kaplan, *M.D.	SAB/NIAMS	10/6D47C	301-496-0517	Mariana.kaplan@nih.gov
John J. O'Shea, M.D.	MIIB, NIAMS	10/13C103	301-496-6026	osheajo@mail.nih.gov
James Balow, M.D	NIDDK	10/5-2551	301-496-4181	jmb@mail.nih.gov
Massimo Gadina, Pharm D, Ph.D.	TIS/OST/NIAMS	10/6D47A	301-443-5457	gadinama@mail.nih.gov
Meryl Waldman, M.D.	NIDDK	10/ 5-2551	301-451-6990	waldmanm@niddk.nih.gov
Betty Diamond M.D.	FIMR	-	516-562-3830	BDiamond@NSHS.edu
Meggan MacKay M.D.	FIMR	-	516- 562-3838	mmackay@nshs.edu
Peter Gregersen M.D.	FIMR	-	516-562-1542	pgregers@nshs.edu
Cynthia Aranow M.D.	FIMR	-	516-562-3837	caranow@nshs.edu
Nehal Mehta M.D.	NHLBI	10/5-3142	301-827-0483	nehal.mehta@nih.gov
Alan Remaley M.D.	NHLBI	10/2C433	301-402-9796	Alan.remaley@nih.gov
Daniella Schwartz* M.D.	OCD/NIAMS	10/13C103	301-443-8544	Daniella.schwartz@nih.gov
Yenealem Temesgen-	OCD/NIAMS	10/3-2340	301-451-4990	Yenealem.temesgen-oyelakin@nih.gov

Oyelakin*, BSN, RN				
Michael Davis*	OCD/NIAMS	10/3-2340	301-496-4644	Michael.davis@nih.gov
Elaine Poncio*	OCD/NIAMS	10/3-2340	301-435-4489	Elaine.poncio@nih.gov
Sarthak Gupta*, M.D.	NIAMS/NIAID	10/6N216	301-443-8541	Sarthak.gupta@nih.gov
Donald Thomas Jr., M.D.	-	-	301-345-5600	donthomasj@aol.com
Mohammad Naqi, M.D.	NIAMS/IRP	10/12N248	301-402-6409	mohammad.naqi@nih.gov
Isabel Ochoa-Navas	OCD/NIAMS	10/3-2340	301-451-4650	ochoai@mail.nih.gov

Key Research Personnel:

Name, Degree	Branch/Institute	Bldg./Rm	Phone	E-mail
Zerai Manna, MSc	SAB/NIAMS	10/4-1341	301-827-0546	zerai.manna@nih.gov
Yinghui Shi	SAB/NIAMS	10/6D46	301-435-8044	yinghui.shi@nih.gov

Associate Investigators with * after their names will be obtaining informed consents under this protocol

Research Contact:

Name, Degree	Branch/Institute	Bldg/Rm	Phone	E-mail
Yenealem Temesgen-Oyelakin, BSN, RN	OCD/NIAMS	10/3-2340	301-451-4990	Yenealem.temesgen-oyelakin@nih.gov

ESTIMATED DURATION OF STUDY: 5 years

START DATE: 08/17/2015 END DATE:

NUMBER AND TYPE OF PATIENTS: 30 Lupus Patients
Accrual Ceiling: 38

Number	Sex	Age Range

Lupus Patients: 30 Females & Males ≥18 ages

PROJECT USES IONIZING RADIATION:
__X__ Medically indicated:

IND/IDE: IND exempt Sponsor: NIAMS
Signature Page:

The signature below constitutes approval of this protocol and attachments, and provides the necessary assurances that this study will be conducted according to all stipulations of the protocol, including all statements regarding confidentiality, and according to local laws and regulatory requirements, applicable U.S. federal regulations, and guidelines established by the International Conference on Harmonization.

____

Principal Investigator (Signature) Date

Table of Contents

CLINICAL RESEARCH PROTOCOL ... 1
NATIONAL INSTITUTE OF ARTHRITIS AND MUSCULOSKELETAL AND SKIN DISEASES ... 1
TABLE OF CONTENTS ... 4
ABBREVIATIONS ... 8
PRÉCIS ... 10
INTRODUCTION/SCIENTIFIC RATIONALE: ... 12
1.1 Overview: ... 12
1.2 Systemic lupus erythematosus; description and epidemiology: ... 12
1.3 Current Treatment Paradigms in SLE: ... 13
1.4 Disease pathogenesis; cytokines in SLE: ... 13
Tofacitinib; description and summary of clinical studies: ... 15
2.1 Tofacitinib; mechanism of action: ... 17
2.2 Rationale for treatment with tofacitinib: ... 17
3. STUDY OBJECTIVES: ... 21
3.1 *Primary Objective:* ... 21
3.2 *Secondary Objectives:* ... 21
4. SUBJECTS: ... 22
4.1 Inclusion Criteria: ... 22
4.2 Exclusion Criteria: ... 23
4.3 Subject Completion and Replacement: ... 25
5 STUDY DESIGN AND METHODS: ... 25
5.1 Description of Study Design: ... 25
5.2 Description of Endpoints: ... 26
5.2.1 Primary Endpoint ... 26
5.2.2 Secondary Endpoints ... 26
5.3 Recruitment: ... 28
5.4 Screening Methods ... 28
5.5 Study Phases ... 28
5.6 Duration of Study Participation ... 29
5.7 Clinical Assessments ... 29
Vital Signs Measurement ... 30
5.8 Adverse event assessment ... 30
5.9 Assessment of lupus disease activity ... 30
5.10 Subject questionnaires ... 30
5.11 Clinical Laboratory Assessments ... 31
5.12 Laboratory Research Studies ... 31
Mechanistic studies ... 31
5.13 Visit Procedures: ... 33
5.13.2 Screening Period (—45 days to Day 1): ... 33
5.13.3 Day 1 +/- 7 days Study Visit: ... 34

5.13.4 Day 7+/- 7days, Day 21+/- 7days, Day 35 +/- 7days and Day 49 +/- 7days Telephone Calls ±7days : 36
5.13.5 Day 14 +/- 7days Study Visit: 36
5.13.6 Day 28 +/- 7 days Study Visit: 37
5.13.7 Day 42 +/- 7 days Study Visit: 38
5.13.8 Day 56 +/- 7 days Study Visit (end of treatment period): 39
5.13.9 Day 84 +/- 7 days (follow up visit, end of study) : 40
5.13.10 Unscheduled Visits: 42
5.14 Follow-up/Termination Procedures: 43
5.15 Study Drug, Dosing and Administration: 43
5.15.1 Tofacitinib: 43
5.15.2 Tofacitinib dosing: 44
5.15.3 Placebo: 44
5.16 Treatment Compliance: 44
5.17 Treatment Allocation: 45
5.17.1 Genotyping and Blinding: 45
5.18 Concomitant medications: 46
5.18.1 Restricted medications: 46
5.18.2 Disallowed medications: 46
5.18.3 Corticosteroids: 47
5.19 Treatment of flares: 47
5.20 Withdrawal criteria: 48
6 Risks and Discomforts: 48
6.1 Unexpected Adverse Events: 48
6.2 Risks/discomfort associated with tofacitinib: 49
6.3 Flare of SLE: 52
6.4 Risks associated with study procedures: 52
6.4.1 Blood draws 52
6.4.2 Electrocardiogram (ECG) 53
6.4.3 Vascular Function Studies 53
7 Safety Monitoring (see section 15) : 55
8 OUTCOME MEASURES: 55
9 STATISTICAL ANALYSIS 56
9.1 Analysis Populations: 56
9.1.1 Safety Population: 56
9.1.2 Efficacy Population: 56
9.2 Data Analysis: 57
Demographic and Baseline Characteristics 57
9.3 Primary outcome: 57
9.4 Secondary outcomes: 57
9.5 Exploratory Analyses: 57
9.6 Criteria for Significance: 58
9.7 Power Analysis: 58
9.8 Interim Analysis: 58
9.9 Accrual Number Request: 58
10 HUMAN SUBJECTS PROTECTION 58

10.1 Ethics and Good Clinical Practice, Data Collection and Data Quality Assurance: 58
10.4 Storage of Data and Samples: 60
10.4.1 Samples: 60
10.4.2 Data: 60
10.4.3 Record Retention: 61
10.4.4 Access to Source Data and Documents: 61
10.4.5 Data Collection, Quality Control and Quality Assurance Monitoring: 62
10.5 Institutional Review Board: 64
10.6 Recruitment of women, children and minorities: 65
10.7 Subject selection: 65
10.8 Qualification of Investigators: 65
11 BENEFITS: 67
12 SIGNIFICANCE TO BIOMEDICAL RESEARCH: 67
13 SUMMARY/CLASSIFICATION OF RISK: 68
14 INFORMED CONSENT DOCUMENTS AND PROCESS: 68
15 GCP COMPLIANCEAND DATA AND SAFETY MONITORING AND REPORTING: 69
15.1 Clinical Monitoring: 70
15.2 Data and Safety Monitoring Committee Oversight: 71
15.3 Suspension Guidelines: 71
16 ADVERSE EVENT AND UNANTICIPATED PROBLEM REPORTING: 72
16.1 Definitions: 72
16.1.1 Adverse Event: 72
16.1.2 Unanticipated Problem: 72
16.1.3 Serious Adverse Event: 74
16.1.4 Medical Events Not Qualifying as Adverse Events or Serious Adverse Events: 74
16.1.5 Clinical Laboratory Test Results Not Qualifying as Adverse Events or Serious Adverse Events: 74
16.1.6 Lupus Flare: 75
16.1.7 Reporting of Adverse Events, Unanticipated Problems and Protocol Deviations: 76
16.1.7.1 Intensity of Adverse Event: 76
16.1.7.2 Relationship to Study Drug and Procedures: 76
16.1.7.3 Expectedness of Adverse Events: 77
16.1.8 Procedures for Reporting 78
16.1.9 Reporting Timeline: 78
16.2 Adverse Event, Protocol deviation and Unanticipated Problem Assessment and Follow-up: 80
16.2.1 Adverse Event Recording: 80
16.2.2 Clinically Significant Laboratory Abnormalities: ......**Error! Bookmark not defined.**
16.2.3 Pregnancy Reporting and Follow-up: 81
16.2.4 Lost to follow up patient reporting: 82
17 ALTERNATIVES TO PARTICIPATION OR ALTERNATIVE THERAPIES: 82
18 CONFIDENTIALITY: 82
19 CONFLICT OF INTEREST/TECHNOLOGY TRANSFER: 82
20 RESEARCH AND TRAVEL COMPENSATION: 83
21 REFERENCES: 84

22 APPENDICES: 90
22.1 Schedule of Events 90
22.2 American College of Rheumatology Revised Classification Criteria for Systemic Lupus Erythematosus 91
22.3 SLEDAI 2K 92
SLEDAI-2K: DATA COLLECTION SHEET 92
22.4 BILAG 2004: 94
22.5 Disease Activity Score-28 (DAS-28): 95
22.6 The Cutaneous Lupus Erythematosus Disease Area and Severity Index (CLASI): 96
22.7 36-item Short Form Survey: 97
22.8 Multidimensional Assessment of Fatigue: 102
55 102
22.9 NIH Clinical Center guidelines for the management of allergic reactions: 106
22.10 XELJANZ (tofacitinib) patient prescribing information: 108
22.11 NCI Common Toxicity Criteria, Version 4.0: 108
22.12 Physician Global Assessment (PGA): 109

Abbreviations

AE	adverse event
AU	arbitrary unit
Anti-ds-DNA	Anti-double stranded DNA antibody
Anti-Sm	Anti-Smith antibody
Anti-SSA	Anti-Sjögren’s Syndrome A antibody
Anti-SSB	Anti-Sjögren’s Syndrome B antibody
ANA	Antinuclear Antibody
CBC	complete blood count
C3	Complement component 3
CD	Cluster of Differentiation
C4	Complement component 4
CTDB	Clinical Trials Data Base
CTSS	Clinical Trials Survey System
CRP	C-reactive protein
DAS-28	Disease Activity Score of the 28 joints
DSMC	Data and Safety Monitoring Committee
ESSDAI	EULAR Sjögren’s Syndrome Disease Activity Index
ESSPRI	EULAR Sjögren’s Syndrome Patient Reported Index
eCRF	electronic case report form
EKG	Electrocardiogram
EDC	electronic data capture
FIMR	Feinstein Institute for Medical Research
ESR	Erythrocyte sedimentation rate
FDA	Food and Drug Administration
GCP	Good Clinical Practice
HLA	Human Leukocyte Antigen
Ig G	Immunoglobulin subtype G
IgE	Immunoglobulin subtype E

IL-4	Interleukin 4
IU	International Units
ICF	Informed Consent Form
ICH	International Conference on Harmonization
IRB	Institutional Review Board
MHC	Major Histocompatibility Complex
MI	Myocardial Infarction
NIAMS	National Institute of Arthritis and Musculoskeletal and Skin Diseases
NIH	National Institutes of Health
NSAID	nonsteroidal anti-inflammatory drug
PK	Pharmacokinetics
PD	Pharmacodynamics
PT/PTT	Prothrombin time/Partial thromboplastin time
BILAG 2004	British Isles Lupus Assessment Group index
SLENA-SLEDAI	Safety of Estrogen in Lupus Erythematosus National Assessment-modification of Systemic Lupus Erythematosus Disease Activity Index
SLEDAI 2K	Systemic Lupus Erythematosus Disease Activity Index 2000
SAE	serious adverse event
SD	standard deviation
SF-36	Short Form-36
SLE	Systemic Lupus Erythematosus
SOP	Standard Operating Procedure
TBNK	T lymphocytes, B lymphocytes and Natural Killer cells
TNF-α	Tumor Necrosis Factor alpha
WBC	White Blood Cells

Précis

Background:

Systemic Lupus Erythematosus (SLE) is an autoimmune disease with variegated clinical presentation resulting from involvement of multiple biologic pathways. The pathways that lead to loss of tolerance in SLE include: multiple autoreactive cell types (B, T, dendritic, Th17 and regulatory T cells) and abnormal cytokine milieus, genetic factors, environmental and hormonal influences, all of which can influence cell differentiation patterns and reset tolerance checkpoints (1, 2). In addition, recent studies indicate a putative role for neutrophils in lupus pathogenesis and associated end-organ damage(3). Currently available therapeutics are frequently inadequate to treat disease flares and simultaneously expose patients to potentially serious toxicities. Further, premature cardiovascular disease not explained by the Framingham risk equation has become one of the most important causes of morbidity and mortality in this patient population. To date, no treatment used in lupus appears to significantly decrease cardiovascular risk. Identifying a drug that has immunomodulatory effects and is also vasculoprotective is an unmet need in this disease.

Tofacitinib is an orally administered Janus kinase (JAK) inhibitor that has recently been approved by the Food and Drug Administration for the treatment of moderate to severe rheumatoid arthritis (RA).

The JAKs are a family of intracellular enzymes (JAK1, 2 and 3 and TYK2) that mediate signaling via a broad range of cytokine receptors (4, 5). Targeting JAKs is an attractive therapeutic possibility for SLE for many reasons. Many of the inflammatory cytokines implicated in the pathogenesis of SLE signal via the JAK-STAT pathways. JAK inhibitors have been found to have efficacy in various murine models of lupus (6). Mice treated with a JAK2 inhibitor exhibited reduced serum levels of IL-6, and IL-17 along with reduced numbers of long-lived autoantibody producing plasma cells in the spleen and bone marrow (7). Furthermore, we have found that administration of tofacitinib reduced serum levels of ANA, IL-6, and IFN-γ; and ameliorated glomerulonephritis (unpublished data).

This study therefore represents an innovative investigative measure of the safety and efficacy of JAK inhibition in SLE that is predicted by genetic predisposition. We will also

investigate effects of tofacitinib on vascular function in SLE subjects and identify biomarkers that may be useful as endpoints in future studies.

Primary Objective:

To determine the safety and tolerability of tofacitinib in patients with SLE and mild to moderate disease activity.

Study Design:

This is a Phase Ib, randomized, double blind, placebo controlled clinical trial of orally administered tofacitinib, 5 mg twice daily, for the treatment of SLE subjects with mild to moderate disease activity stratified by the presence or absence of STAT4 risk alleles.

INTRODUCTION/SCIENTIFIC RATIONALE:

1.1 *Overview:*

The proposed research is an exploratory Phase Ib clinical trial, the primary focus of which will be to test the safety of tofacitinib, a JAK inhibitor, in SLE. A secondary goal will be to do exploratory mechanistic studies as a prologue to future studies and to identify candidate surrogate markers that might relate to clinical efficacy. The patient population for this trial will be stratified by the presence or absence of STAT4 risk alleles to investigate the effect(s) of these genetic haplotypes on response to tofacitinib. While the numbers of patients may be too small to discern significant effects, STAT4 genotyping will be performed as an exploratory effort. This clinical trial seeks to address an important medical problem: SLE is a chronic autoimmune disease that has no cure and current therapeutic strategies are limited in their efficacy and by their significant toxicities.

1.2 *Systemic lupus erythematosus; description and epidemiology:*

SLE is characterized by anti-nuclear antibody production and pathological findings of inflammation, vasculitis, vasculopathy and immune complex deposition in multiple target organs. Lupus occurs throughout the world and susceptibility is clearly modulated by ethnicity and gender. Although it affects both males and females of all age groups, it most commonly presents in women of reproductive age with a striking female to male ratio of approximately 9:1 (8). This ratio is approximately 2-3:1 in younger and older populations, supporting a role for hormonal factors in the induction of disease. Incidence rates reported during the last 25 years in North America vary from 2 to 7 per 100,000; rates in African-American, Afro-Caribbean, Hispanic and Asian populations are approximately three times greater than in Caucasian populations. The worldwide prevalence of lupus ranges from 17 to 48 per 100,000, but has been reported as high as 207/100,000 in an Afro-Caribbean population in the United Kingdom. While a precise etiology of SLE is not known, combinations of genetic, hormonal and environmental factors are thought to contribute to the loss of self-tolerance. In rare individuals, single gene mutations play a major role, e.g., 98% of individuals with complete deficiency of C1q develop SLE. For the vast majority of individuals with SLE the genetic contribution appears to be polygenetic. The importance of non-genetic contributors to disease factors (i.e., hormonal and environmental factors) is also apparent since disease discordance in monozygotic twins is at least 60%.

1.3 *Current Treatment Paradigms in SLE:*

The current management of patients with SLE is usually stratified by the degree of internal organ involvement; however, most treatment strategies include a variety of immunosuppressive medications that are limited both in their efficacy and by their potential toxicities (reviewed in (9)). FDA-approved treatments for SLE include only hydroxychloroquine, corticosteroids, aspirin, and most recently, belimumab. NSAIDs are relatively contraindicated in patients with renal disease and potential adverse effects on photosensitivity, aseptic meningitis and the gastrointestinal tract also limit their use in SLE. Potentially devastating side effects of corticosteroids are well-known and include infection, avascular necrosis, weight gain, osteoporosis, cataracts and development of diabetes. The use of immunosuppressive drugs for musculoskeletal symptoms refractory to NSAIDs or requiring continued steroid therapy has become “standard of care” by the rheumatologic community. Although often beneficial for treatment of active disease, these immunosuppressive medications (ex: azathioprine, methotrexate, mycophenolic acid, leflunomide, cyclophosphamide, cyclosporine) are associated with multiple toxicities; most commonly infection (with potentially fatal outcomes), hepatic and renal impairment and infertility. Despite the addition of these potentially toxic agents, SLE patients usually require continued treatment with corticosteroids. Thus, lupus patients are typically dependent indefinitely on corticosteroids and/or immunosuppressive agents for disease control even while developing cumulative toxicities from exposure to these drugs. Clearly there is an unmet need for improved treatment of inflammation in this patient population. Further, premature cardiovascular disease has become one of the most important causes of morbidity and mortality in this patient population and it is not explained by the Framingham risk equation. To this date, no drug used in lupus appears to significantly decrease cardiovascular risk. Therefore, identifying a drug that has both immunomodulatory and vasculoprotective effects would fill an important unmet need in this disease.

1.4 *Disease pathogenesis; cytokines in SLE:*

End organ damage in SLE is the consequence of a series of events characterized by loss of tolerance resulting in autoantibody production and ending in an inflammatory cascade that leads

to tissue destruction. Auto-reactive B and T cells as well as abnormally primed dendritic cells all contribute to the abnormal immune response in SLE. However, at a cellular level, much of the tissue destruction appears to be cytokine mediated. A variety of cytokines, including: IL-1, IL-2, IL-6, IL-10, IL-18, IL-21, IFN α, IFN γ, and TNF α, have been implicated in the immunopathogenesis of SLE. These molecules mediate tissue destruction and contribute to breaking tolerance through effects on B cell activation, immunoglobulin production and expression of costimulatory molecules on lymphocytes.(10-12) Immune complexes from SLE stimulate peripheral blood mononuclear cells (PBMCs) to express the highly pro-inflammatory cytokines TNF α and IFNα via Fc gamma receptor and TLR 9 mechanisms(13-15). Interestingly, deficiencies in Fc gamma receptors or the inability to produce high levels of IFN γ are protective against acute glomerulonephritis in murine models of SLE.(16, 17) IFN γ, IL-1β, TNF α, TGFβ, IL-18 and IL-6 are all expressed at high levels in the kidneys of mice with active glomerulonephritis leading to increased inflammation, hypercellularity, fibrosis and renal dysfunction (18-20). The same array of cytokines has also been identified in kidney biopsy tissue from SLE patients with active glomerulonephritis (11, 21, 22) and some of these cytokines have also been found in the urine of patients with active nephritis (11, 23, 24). Skin biopsies from SLE patients have yielded increased expression of IFN α and IL-6 in active sites (25-27). There are conflicting reports on circulating levels of all of these cytokines in SLE patients; it is assumed that the tissue levels (understandably difficult to measure) are truly reflective of the pathologic process and reports of cytokine levels in tissue specimens in murine models and humans have been reasonably consistent.

The idea of uncoupling autoantibody/immune complex formation from cytokine production and directing therapy at known cytokines is not without precedent; TNF α blockade has revolutionized the treatment of rheumatoid arthritis. Thus far, in SLE, anti- IL-10 (28, 29) and anti-TNF α (30-32) have been used successfully in murine models. Clinical trials of TNFα inhibitors in human SLE have been halted due to safety concerns. In addition, recent reports from clinical trials indicate that anti-IFNα and anti-IL-10 therapy (33), though well-tolerated, may not be effective in human disease. These data suggest that a multi-targeted approach to this disease using a JAK inhibitor may be beneficial.

Tofacitinib; description and summary of clinical studies:

Tofacitinib (CP-690550/XELJANZ; Pfizer) is an orally administered JAK inhibitor that has recently been approved for the treatment of rheumatoid arthritis (RA) and is currently being developed for use in inflammatory bowel disease, psoriasis, ankylosing spondylitis, juvenile arthritis and renal allograft transplantation. Clinical trials of tofacitinib in RA demonstrate rapid onset of drug efficacy with an acceptable safety profile (34-38). These clinical trials have used tofacitinib as 1) monotherapy in patients failing non-biologic or biologic disease modifying drugs (DMARDS), 2) in combination with methotrexate in patients failing methotrexate or TNF inhibitors and the combined results are shown in Table 1.

Table 1: Proportion of Patients with an ACR Response Percent of Patients									
Monotherapy in Nonbiologic or Biologic DMARD Inadequate Responders[c]				MTX Inadequate Responders[d]			TNF Inhibitor Inadequate Responders[e]		
Study I				Study IV			Study V		
N[a]	PBO 122	XELJANZ 5 mg Twice Daily 243	XELJANZ 10 mg Twice Daily 245	PBO + MTX 160	XELJANZ 5 mg Twice Daily + MTX 321	XELJANZ 10 mg Twice Daily + MTX 316	PBO + MTX 132	XELJANZ 5 mg Twice Daily + MTX 133	XELJANZ 10 mg Twice Daily + MTX 134
ACR20 Month 3 Month 6	26% NA[b]	59% 69%	65% 70%	27% 25%	55% 50%	67% 62%	24% NA	41% 51%	48% 54%
ACR50 Month 3 Month 6	12% NA	31% 42%	36% 46%	8% 9%	29% 32%	37% 44%	8% NA	26% 37%	28% 30%
ACR70 Month 3 Month 6	6% NA	15% 22%	20% 29%	3% 1%	11% 14%	17% 23%	2% NA	14% 16%	10% 16%

[a] N is number of randomized and treated patients.
[b] NA Not applicable, as data for placebo treatment is not available beyond 3 months in Studies I and V due to placebo advancement.
[c] Inadequate response to at least one DMARD (biologic or nonbiologic) due to lack of efficacy or toxicity.
[d] Inadequate response to MTX defined as the presence of sufficient residual disease activity to meet the entry criteria.
[e] Inadequate response to a least one TNF inhibitor due to lack of efficacy and/or intolerance.

Improvement with doses of 5 mg twice daily was noted as early as two weeks, with ACR 20 responses reported in 41-59% and 51-69% of subjects at 3 and 6 months respectively. Similarly, ACR 50 responses were reported in 26-31% and 32-42% of subjects at 3 and 6 months respectively. In general, tofacitinib was well tolerated; the most common toxicity reported was infection with an overall frequency of 20% in the patients who received tofacitinib 5 mg twice daily and 18% in the patients who received placebo. The most commonly reported infections were upper respiratory infections, throat infections and urinary tract infections.

Serious infections, pneumonia, cellulitis, herpes zoster, and urinary tract infections due to bacterial or opportunistic organisms (Herpes simplex, Cytomegalovirus, Cryptococcus, Pneumocystis and Candida) were observed in 0.4% of subjects who received drug for 3 months or less. Other toxicities, including malignancy and gastrointestinal perforation were rare. Laboratory abnormalities, including lymphopenia, neutropenia, anemia, increased transaminases and elevated high density lipoprotein (HDL) and low density lipoprotein (LDL) were also considered mild and reversible. The lymphopenia (absolute lymphocyte count <500 cells/mm3) was associated with an increased risk of infection but the neutropenia was not. Pooled data from 2 open-label long-term extension studies involving 4102 patients treated for 5963 patient-years reported a cardiovascular events incidence rate range from 0.05-0.3 per 100 patient-years (39) . Increase in serum creatinine of more than 50% from baseline was observed in 3.3 % of the patients(39).

2.1 Tofacitinib; mechanism of action:

The JAKs are a family of intracellular enzymes (JAK1, 2 and 3 and TYK2) that facilitate signaling between inflammatory cytokines bound to surface receptors and subsequent gene transcription that regulates immune response (4). Specifically, JAKs phosphorylate cytoplasmic tails of cytokine receptors once the receptor has been bound by its cognate cytokine. This leads to recruitment of appropriate Signal Transduction and Transcription (STAT) proteins, which are in turn phosphorylated. This leads to dimerization and disassociation from the receptor-JAK complex. STATs translocate to the nucleus where they regulate gene transcription. Specific cytokine receptor subunits selectively associate with different JAKS and STAT (Fig. 1). JAKs play a critical role in mediating inflammatory responses and pharmacologic intervention that modulates JAK function represents a novel approach to the treatment of autoimmune disease.

Figure 1. Associations between JAK kinases, cytokines and STAT proteins

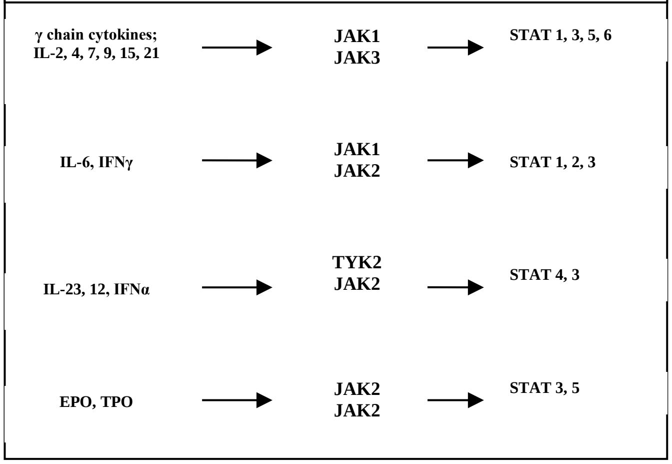

Tofacitinib has been shown to inhibit JAK1, JAK3 and JAK2, however, cellular assays that measure cytokine-induced STAT phosphorylation have demonstrated partial selectivity of tofacitinib for JAK 1 and 3 resulting in greater inhibition of JAK 1 and 3 compared to JAK 2(40). JAK1 and JAK3 mediate intracellular responses for cytokines that use the gamma chain, which are critical for the development and function of T, B, and NK cells. Other in vitro studies suggest that the effectiveness of tofacitinib in RA may be attributable to its suppressive effects on the generation of Th1 and pathogenic Th17 cells (41-43). Tofacitinib also inhibits cytokines that provide B cell help such as IL-4, IL-6, and IL-21. In addition, tofacitinib inhibits the effect of IL-6 and IFN-α on innate immune cells.

2.2 Rationale for treatment with tofacitinib:

2.2.1 Cytokine signaling: Targeting JAKs is an attractive therapeutic possibility for SLE for many reasons. Many of the inflammatory cytokines implicated in SLE

pathogenesis, in particular IFNα, IL-6, IL-23, IL-12, IL-21 and IFNγ, signal through JAK/STAT pathways. These molecules mediate tissue destruction and contribute to breaking tolerance through effects on B cell activation, immunoglobulin production and expression of costimulatory molecules on lymphocytes (10-12). Immune complexes from SLE stimulate peripheral blood mononuclear cells (PBMCs) to express the highly pro-inflammatory cytokines TNFα and IFNα via Fc gamma receptor and TLR 9 mechanisms (13-15). Interestingly, deficiencies in Fc gamma receptors or the inability to produce high levels of IFNγ are protective against acute glomerulonephritis in murine models of SLE (16, 17) and IFN γ genetic polymorphisms are associated with disease susceptibility (44). IFNγ, IL-1β, TNFα, TGFβ, IL-18 and IL-6 are all expressed at high levels in the kidneys of mice with active glomerulonephritis leading to increased inflammation, hypercellularity, fibrosis and renal dysfunction (18-20). The same array of cytokines has also been identified in kidney biopsy tissue from SLE patients with active glomerulonephritis (11, 21, 22) and some of these cytokines have also been found in the urine of patients with active nephritis (11, 23, 24). Skin biopsies from SLE patients show increased expression of IFNα and IL-6 in active sites (25-27). Following engagement of its receptor, IFNα activates JAKs and STATs leading to increased expression of MHC Class I, dendritic cell maturation, T cell survival and autoantibody production (45). There are currently several monoclonal antibodies directed against different isoforms of IFNα in clinical trials for SLE treatment. Known functions of IL-6 are to enhance B cell differentiation into immunoglobulin secreting plasma cells and promote antibody production through stimulation of IL-21 by CD4+ T cells (46, 47). IL-6 is an important factor that induces naïve CD4+ cells to differentiate into Th17 cells through activation of STAT3 and induction of RORγt (48). Additionally, anti-IL-6 monoclonal antibodies are currently being tested in clinical trials for a number of autoimmune diseases including SLE. Positive anti-inflammatory effects of anti-IL-6 therapy are attributable, in part, to diminished support of long lived plasma cells by IL-6. IL-17 is recognized as an important contributor to lupus pathogenesis through its effects on neutrophils and monocytes and T cell

migration into tissues. Increased numbers of IL-17-producing T cells have been identified in SLE patients and in renal biopsies from lupus nephritis patients. Positive correlations between disease activity and IL-17 producing T cells have also been reported (49-51). A selective JAK2 inhibitor has been used to successfully treat established SLE and nephritis in murine models of SLE. These mice exhibited reduced serum levels of IL-6 and IL-17 along with reduced numbers of long-lived autoantibody producing plasma cells in the spleen and bone marrow (at higher doses) (7). IL-23 is mainly secreted by antigen presenting cells and signals through JAKs and STATs to promote expansion of IL-17 producing cells (including Th17 cells), increase IFNγ production, activate memory T cell responses and increase production of pro-inflammatory cytokines (rev in (52)). Elevated mRNA expression of IL-23 has been correlated with disease activity and renal disease in human SLE (53-55) and IL-23 receptor-deficient MRL/lpr mice do not develop renal disease (56). Given the importance of these pro-inflammatory cytokines that signal through JAKs and STAT proteins to the inflammatory response in lupus, use of tofacitinib has the potential to block effects of several cytokines known to exacerbate inflammatory responses in SLE rather than singling out one cytokine target at a time.

2.2.2 T cell pathology in SLE and JAK inhibition: The importance of T cell contributions to pathology in SLE is well established (57). The MHC locus remains the strongest genetic association with SLE, supporting the role of T cell-driven auto-reactivity. The plethora of high affinity, class-switched IgG autoantibodies characteristic of SLE reflect the role of follicular T helper (T_{fh}) cells in B cell proliferation and differentiation giving rise to autoantibody producing plasma cells (58). T_{fh} cells are generated from naïve CD4+ T cells in the presence of IL-6, IL-21 and ICOS stimulation. Inflammation is locally driven in target organ tissue in response to cytokines that regulate vascular permeability and enhance local extravasation of inflammatory cells into tissue. Th17 cells are generated from naïve CD4+ T cells in the presence of TGFβ, IL-1β, IL-6, IL-21 and IL-23 and they moderate inflammatory responses by releasing the pro-inflammatory cytokines IL-17 and IL-21. Engagement of the IL-17 receptor on

target cells leads to chemokine production and results in leukocyte recruitment and production of other inflammatory cytokines such as IL-1β and TNFα. Th17-related pathology is described in multiple autoimmune diseases including RA, psoriasis, inflammatory bowel disease, and SLE (59). High levels of IL-17 from Th17 cells and CD4-, CD8- (double negative, DN) T cells correlate with disease activity in SLE (60) and both of these cell types have been identified within inflammatory infiltrates in human lupus nephritis (49, 61) and murine models of lupus nephritis. DN T cells, though rare in healthy individuals, are expanded in SLE, produce pro-inflammatory cytokines (IFNγ, IL-17, IL-1β) and target organ tissue.

Ghoreschi et al have demonstrated, through a series of in vitro cellular assays and in vivo animal models, the range of tofacitinib's effects on T cell function and inflammatory cytokines (41). Tofacitinib inhibits JAK3 dependent γ_c cytokine receptor signaling and other JAK1 dependent cytokine receptor signaling. These blocking effects manifest as decreased production of inflammatory IL-23 dependent Th17 cells. Additionally, tofacitinib blockade of JAK1 and JAK3 inhibited Th1 and Th2 differentiation and blocked IL-6 signaling. In the animal model of collagen-induced arthritis, mice experienced a significant reduction in established arthritis within 48 hours of treatment with tofacitinib and circulating inflammatory markers decreased within 4 hours; demonstrating a remarkably fast onset of action. These immediate results were not associated with leukocyte depletion; histologic confirmation of leukocyte depletion took 7 days. Others have demonstrated that treatment of human subjects with tofacitinib for prevention of graft rejection after kidney transplant results in a differential effect of tofacitinib on Treg cells and Th1 effector cells where the Th1 effector cells proved more susceptible to JAK blockade (62). These studies have direct implications for the use of JAK inhibition in SLE where, similar to the positive results demonstrated in RA, we predict that inhibition of signaling molecules with broad effects on inflammatory responses will prove to be beneficial.

The reason for a Phase 1b study of tofacitinib in SLE is that tofacitinib also inhibits cytokines that may be protective in SLE. IL-2 is an important immunoregulatory cytokine that promotes immune responses but it is also important for peripheral tolerance. IL-2 promotes expression of

Foxp3 in regulatory T (Treg) cells. It also inhibits IL-17 production and Bcl6 expression; Bcl6 is important for T_{fh} cells and germinal center formation. While there is no evidence in mouse models or in humans treated with tofacitinib that this drug promotes autoimmunity, this remains a theoretical possibility. Regulatory T cells (Tregs), identified by the presence of the FOXP3 intracellular transcription factor, help modulate the duration and intensity of inflammatory responses. Decreased numbers of circulating Tregs have been correlated with disease activity in human SLE (63, 64) although other studies report normal levels in active disease (65). It is not clear whether Tregs are intrinsically less effective at suppression of inflammation in SLE or whether target cells are more resistant. Some studies have demonstrated inhibition of immune complex-mediated glomerulonephritis in murine models with adoptive transfer of Tregs (66, 67).

In addition, tofacitinib can inhibit JAK2 and thereby interfere with the action of erythropoietin, GM-CSF and IL-7. Consequently, tofacitinib could theoretically exacerbate the anemia and lymphopenia associated with SLE.

Tofacitinib treatment is also associated with higher rates of BK virus nephropathy. Patients with SLE were found to be asymptomatic career of BK virus in urine (32%) in one cross sectional cohort study. Hence there is a possibility of BK virus activation leading to clinically significant disease after starting patients on tofacitinib. We will include assessment of BK virus infection at baseline and throughout the study for an additional safety measure.

3. STUDY OBJECTIVES:

3.1 *Primary Objective:*

- To determine the safety and tolerability of tofacitinib in patients with Systemic Lupus Erythematosus and mild to moderate disease activity.

3.2 *Secondary Objectives:*

- To assess clinical improvement after treatment with tofacitinib as measured by improvement in the Systemic Lupus Erythematosus Disease Activity Index (SLEDAI 2K) and no worsening on the physician's global assessment scale.
- To demonstrate that treatment with tofacitinib is more effective clinically and biologically in a subset of SLE subjects with STAT4 risk alleles than those without.

- To demonstrate that treatment with tofacitinib is more effective clinically and biologically in a subset of SLE subjects with mild to moderate disease as compared to placebo.
- To demonstrate that oral administration of tofacitinib does not result in increased disease activity as measured by the SLEDAI 2K disease activity index.
- To investigate the effects of tofacitinib on intracellular signaling molecules, serum cytokines and peripheral blood gene expression as a measure of biological effects that can be used as outcome measures to power a Phase II Clinical Trial.
- To investigate the effects of tofacitinib on patient quality of life measures as assessed by the patients' global assessment of disease activity, the Multidimensional Assessment of Fatigue questionnaire and the Short Form 36 health survey.
- To investigate the effects of tofacitinib in modulation of endothelial responses and markers of vascular risk in SLE.

4. SUBJECTS:

Adult SLE patients with mild to moderate disease activity will be eligible for the study. These patients can be naïve or failed immunosuppressive therapy beyond anti-malarials and glucocorticoids. We plan to enroll immunosuppressive therapy naïve patients as well as patients who have failed past therapies so as not to bias the cohort to patients with more recalcitrant disease. We expect that tofacitinib is a potential second line therapy, in addition to anti-malarials and glucocorticoids, depending on the patient's initial presentation and response and thus would like to include such patients in this study. Patients on the study will be followed weekly and thus any indication of worsening disease will be assessed promptly and patients will be withdrawn from the study accordingly for standard of care treatment as detailed in study withdrawal criteria. The following are the inclusion and exclusion criteria:

4.1 Inclusion Criteria:

Subjects who meet all of the following criteria are eligible for enrollment into the study:

1. Subject is capable of providing written informed consent.
2. Subject is ≥ 18 years old.
3. Meets at least 4 of 11 modified American College of Rheumatology (ACR) (1997) Revised Criteria for the Classification of Systemic Lupus Erythematosus

4. Has mild to moderate disease activity defined as SLEDAI-2K ≥ 2 and ≤14 at the screening visit.
5. If on glucocorticoids, the dose must be ≤20 mg daily and stable for the 4 weeks prior to screening visit.
6. If on hydroxychloroquine or other antimalarials such as chloroquine or quinacrine, the dose must have been stable for the 12 weeks prior to screening visit. The maximum allowed dose is hydroxychloroquine up to 400 mg/day or 6.5 mg/kg/day if more than 400 mg/day. The maximum allowed dose for chloroquine phosphate is up to 500 mg daily and for quinacrine up to100 mg daily.
7. Males and females with potential for reproduction must agree to practice effective birth control measures. **Females should be on adequate contraception if they are of child-bearing potential documented by a clinician, unless patients or spouse have previously undergone a sterilization procedure. Adequate will be considered intrauterine device (IUD) alone or hormone implants, hormone patches, injectables, or oral contraceptives plus a barrier method (male condom, female condom or diaphragm), abstinence or a vasectomized partner**
8. If patients are on ACE inhibitors or ARB medications, dose of this medication must be stable for 4 weeks prior to study entry.
9. Patients may be on lipid lowering medications if initiated at least 6 months prior to the screening visit.

4.2 Exclusion Criteria:

Subjects who meet any of the following criteria are disqualified from enrollment in the study:

1. Pregnant or lactating women. Women of childbearing potential are required to have a negative pregnancy test at screening.
2. Current or prior treatment with rituximab, belimumab or any other biologic agent in the 6 months prior to screening.
3. Current treatment with immunosuppressive drugs (methotrexate, azathioprine, mycophenolate mofetil, cyclosporine, tacrolimus). Glucocorticoids are allowed as per

the inclusion criteria. At the investigator's discretion, glucocorticoids may be tapered during the study. This should not be in here.

4. Patients previously on methotrexate, azathioprine, mycophenolate mofetil, cyclosporine or tacrolimus should have stopped it for at least 8 weeks at the time of screening.
5. Treatment with cyclophosphamide, pulse methylprednisolone or IVIG within the 6 months prior to screening.
6. Increase in glucocorticoid dose within 4 weeks of screening.
7. A history of drug or alcohol abuse within the 6 months prior to screening.
8. History of chronic liver disease or elevated LFTs:
   - ALT or AST ≥ 2x upper limit of normal at screening
   - serum unconjugated bilirubin > 2mg/dL at screening
9. Dialysis or serum creatinine >1.5mg/dL.
10. Protein to creatinine ratio of more than 1. mg/mg or 24 hours urine protein of more than 1000 mg.
11. Active urinary sediment (WBC, RBC or mixed cellular casts 1+ or more /hpf).
12. Hypercholesterolemia: Values after an 8-12 hour fasting blood specimen: total cholesterol >250 mg/dL or LDL >180 mg/dl or hypertriglyceridemia (triglyceride >300 mg/dL) within +/- 45 days of screening visit.
13. Active infection that requires the use of oral or intravenous antibiotics and has not resolved at least 2 weeks prior to the administration of the first dose of study medication.
14. Active chronic infections including but not limited to HIV, Hepatitis B, Hepatitis C, and BK viremia at screening visit.
15. History of cancers.
16. Known active tuberculosis or untreated latent tuberculosis.
17. History of opportunistic infections.
18. Subjects with active renal or central nervous system disease or a BILAG A in any organ system.
19. WBC <2500/µL or ANC <1,000/µL, Hgb <9.0 g/dL or platelets <70,000/µL or absolute lymphocyte count < 500/µL.
20. Current treatment with potent inhibitors of Cytochrome P450 3A4 (CYP3A4) (e.g., ketoconazole) or receiving one or more concomitant medications that result in both

moderate inhibition of CYP3A4 and potent inhibition of CYP2C19 (e.g., fluconazole) that would increase serum availability of tofacitinib. Past treatment with the above mentioned agent is allowed if it was more than a week prior to the administration of the first dose of study medication.

21. Significant impairment of major organ function (lung, heart, liver, kidney) or any condition that, in the opinion of the Investigator, would jeopardize the subject's safety following exposure to the study drug.

4.3 Subject Completion and Replacement:

A subject is considered to have completed the study when he/she has completed 56 days of treatment with tofacitinib and the Day 84 follow-up assessments.

Enrolled subjects who withdraw from the trial prior to starting on study drug will be replaced. Subjects who discontinue study treatment and/or withdraw from the trial for any reason other than drug related adverse events or lack of efficacy after initiating the first dose of tofacitinib and before completing 38 doses of tofacitinib will also be replaced in order to maintain the sample size of 10 subjects per group and a total of 30 subjects. Based on experience with studies in SLE patients, we expect a withdrawal rate of approximately 25% and therefore plan for a recruitment ceiling of 38 subjects.

5 STUDY DESIGN AND METHODS:

5.1 Description of Study Design:

This is a Phase Ib, randomized, double blinded, placebo controlled clinical trial of orally administered tofacitinib, 5 mg twice daily, for treatment of SLE subjects with mild to moderate disease activity stratified by the presence or absence of STAT4 risk alleles. There will be 3 arms in the study with 10 subjects in each arm and a recruitment ceiling of 38 subjects. Twenty subjects will be randomized to receive treatment with tofacitinib 5 mg twice daily; 10 of these subjects will be heterozygous or homozygous for the STAT 4 risk alleles and 10 will not. An additional 10 SLE subjects with variable genotypes will receive placebo twice daily. Both the investigators and study subjects will be blinded to the treatment allocation. The study duration is a maximum of 16 weeks; with 45 days screening period followed by an 8 week treatment period

and a 4 week follow-up period. The proposed clinical trial is an exploratory study designed to yield preliminary data about the safety, clinical and biologic efficacy of tofacitinib in SLE subjects as well as information about the influence of genotype on drug efficacy. Provided the drug is well tolerated in the SLE subjects, the data can be used to design and power a larger Phase II study of clinical efficacy and safety.

5.2 Description of Endpoints:

5.2.1 Primary Endpoint

The primary endpoint is safety of tofacitinib in SLE subjects. In order to assess safety, toxicity is defined as any study drug-related Grade 3 adverse event or higher (as measured by the National Cancer Institute (NCI), Common Terminology Criteria for Adverse Events (CTCAE), Version 4.0). Grade 3 adverse events will include measures of standard laboratory tests including serum chemistries, urinalysis, complete blood counts and lipid profiles at screening, baseline and conclusion of the treatment period and at the end of the study. Ideally, lipid studies will be performed on a 8-12 hour fasting state, however if fasting samples are not available we may still analyze the samples noting this limitation. In addition to the routine lipid profile and lipoprotein panel, we may also include measurement

s of proinflammatory HDL and oxidized HDL that has been associated with increased atherosclerotic risk in SLE (68).

5.2.2 Secondary Endpoints

- Preliminary assessments of clinical response will be measured by:
    - the change in SLEDAI 2K between baseline and week 8 (end of treatment)
    - the change in the Physicians Global Assessment scores between baseline and week 8 (end of treatment).
- Changes in anti-dsDNA autoantibody titers, complement proteins C3 and C4, markers of systemic inflammation such as ESR and CRP between baseline and Day 56/week 8 (end of treatment).
- Assessment of biologic effect(s) will be measured by changes in the following mechanistic endpoints between baseline and Day 56/ week 8 (end of treatment):
    - Alteration in peripheral blood immune cell populations by phosphoflow cytometry with special attention to NK, CD8+, CD4+, CD25+, Foxp3+ regulatory subsets and Th17 cells.

  - Ligand-induced STAT phosphorylation in circulating T cells and monocytes using phosphoflow assessment of single cells.
  - Expression of STAT-related target genes; expression of pro-inflammatory and immunoregulatory cytokines in T cells and monocytes using RNAseq.
  - Alteration in the "interferon signature" and the "granulocyte signature" in PBMCs using nanostring; measures of CD3+ T cell expression of IFN regulatory factor-related genes using RNAseq and nanostring.
  - Alteration in peripheral blood immune cell populations with special attention to a subset of aberrant neutrophils present in lupus patients (low-density granulocytes-LDG) (17). With regards to the latter, assessments will include quantification of LDG levels in peripheral blood and their propensity to form neutrophil extracellular traps (NETs) in the absence of added exogenous stimuli.
  - Measures of serum cytokines (IL-2, 4, 6, 7, 9, 15, 12,17, 23, IFNα and IFNγ)
  - Changes in vascular function, as assessed by the reactive hyperemia index (RHI) using Endopat device, arterial stiffness using cardio-ankle vascular index (CAVI), and by using SphygmoCor device to determine central blood pressures and arterial stiffness. These are surrogate markers of vascular damage and future atherosclerosis development which are amenable to change within this treatment timeframe.
  - Measurements of lipoproteins, including proinflammatory HDL and oxidized LDL that has been associated with increased atherosclerotic risk in SLE (68). Additional studies may be performed to assess modifications in the proteome of HDL, reverse cholesterol transport as well as lipoprotein particles.
- Assessment of the effect of STAT4 risk alleles on drug efficacy will be measured by a comparison of changes in all of the above clinical and biologic measures between Days 1 and 56 between the SLE subjects with the STAT4 risk alleles and those without.
- Assessment of durability of the clinical and biologic effects will be measured by changes in all of the above clinical and biologic measures between Day 56/week 8 (end of treatment) and Day 84/week 12 (end of study).

- Patient reported outcomes for clinical efficacy will be measured by changes in the SF-36, the Multidimensional Assessment of Fatigue questionnaire and the Patient Global Assessment scores between baseline and Day 56/week 8 (end of treatment).

5.3 Recruitment:

Patients may be recruited:

- From the Outpatient Rheumatology Clinic of the Clinical Center at the NIH or the NIAMS Community Health Center;
- From the Lupus Clinics associated with the Feinstein Institute for Medical Research (FIMR), Manhasset, NY;
- From patients referred for treatment and/or second opinion;
- From local area rheumatology and nephrology practices and university hospital clinics;
- By advertising the study to the rheumatologists and nephrologists;
- By direct advertising to patients through publications of patient advocate organizations, such as the Arthritis Foundation, the Lupus Foundation, Lupus Research Institute, Alliance for Lupus Research, and in the news outlets.

5.4 Screening Methods

All potential subjects will have preliminary screening done under the "Studies of the Pathogenesis and Natural History of Systemic Lupus Erythematosus" protocol (94-AR-0066) at the NIH Clinical Research Center. Subjects who are eligible for the study will be asked to sign the informed consent document.

Potentially eligible patients identified in the Lupus Clinics at the FIMR will be approached by the investigators at FIMR to discuss the study. If interested, an appointment will be made at the NIH Clinical Research Center to review and sign the informed consent document. A subject is considered enrolled in the study when a signed informed consent is obtained.

5.5 Study Phases

5.1.1 Pre-Screening and Screening:

Subjects will be pre-screened on the "Natural History of SLE" protocol (94-AR-0066). Those with mild-moderate active lupus and no obvious exclusion criteria will be offered the chance to enroll in the study. Interested subjects will sign the

consent form and undergo formal screening. This will take place within 45 days of the first treatment. Routine laboratory data obtained within 21 days of the pre-screening visit can be used for screening. All patients will have their screening visit at the NIH Clinical Research Center.

5.1.2 Treatment Period:

All subjects who fulfill eligibility criteria will be randomized to one of 2 dosing arms; tofacitinib 5 mg twice daily (n=20 subjects, 10 of whom are homozygous or heterozygous for STAT4 risk allele) or placebo twice daily (n= 10 subjects) for 56 days. Each subject will be seen at the NIH Clinical Research Center Day hospital or Outpatient clinic at the NIH at baseline. After this baseline visit each subject will be seen in the either at the Clinical Research Center Day Hospital or outpatient clinic at the NIH for days 14, 28, 42, 56 and 84 (all visits are +/- 7 days). They will have a physical exam, blood draw and be assessed for adverse events and compliance with medications and study drug at each of these visits (see Appendix 21.1 for the schedule of events). Each subject will receive a telephone call on days 7, 21, 35 and 49(all days are +/-7 days) to assess adverse events and compliance. Day 56 (+/-7 days) will be the end of the treatment period.

5.1.3 Follow-Up Period:

There will be a follow up safety visit at Day 84(+/- 7 days). End of follow up (EFU) end-points will be collected at this visit.

5.6 Duration of Study Participation

Study participation will be a maximum of 16 weeks, comprised of a 45 day screening period 8 weeks treatment period, and 4 week follow up period. All study time periods are inclusive of a window of +/- 7 days to accommodate any potential variability in subject scheduling (see Appendix 21.1; Schedule of Events).

5.7 Clinical Assessments

Clinical assessment will take place in the outpatient clinics or the day hospital of the Clinical Research Center of the NIH. Vascular assessments will be performed in the Vascular Lab at the NIH Clinical Research Center.

Vital Signs Measurement

Vital signs measurements will include pulse rate, systolic and diastolic blood pressure, respiratory rate, and temperature. These measurements will be performed at all study visits.

5.8 Adverse event assessment

Adverse events will be assessed by reviewing the interim medical history, performing a physical evaluation, asking open-end question of how the patient's feeling and reviewing laboratory results at each visit. Adverse events will be collected after the subjects receive their first dose of study medication until their last study visit. Abnormalities detected at the screening visit will be noted as baseline but not considered adverse events.

5.9 Assessment of lupus disease activity

Lupus disease activity will be assessed by completing the following measures:

- SLEDAI 2K (Appendix 22.3): This validated disease activity measure reflects clinical events that occurred within 28 days, and includes physical findings and laboratory measures.
- BILAG 2004
- Physician Global Assessment (PGA) (Appendix 21.4): Global disease activity will be rated by the PGA
- Joint count: A 28 tender and swollen joint count (DAS-28) (Appendix 22.5) will be done.
- Cutaneous Lupus Erythematosus Disease Area and Severity Index (CLASI) (Appendix 22.6): The severity of cutaneous involvement (when applicable) will be documented on the CLASI

5.10 Subject questionnaires

The following assessments will be utilized to assess patient reported outcomes:

1. Fatigue will be measured by the Multidimensional Assessment of Fatigue questionnaire (Appendix 21.8).
2. Quality of life by the Short Form 36 (SF36) (Appendix 21.7) questionnaire.
   Both of these tools are validated and widely used in SLE.
3. Subjects will also be asked to indicate overall assessment of disease activity using the Patient Global Assessment Scale.

5.11 Clinical Laboratory Assessments

The CLIA certified clinical laboratories at the NIH Clinical Center (Department of Laboratory Medicine) will be the central laboratories for all routine clinical laboratory parameters including CBC, chemistries, urinalysis, PT/PTT, ESR, CRP, hepatitis B surface antigen, hepatitis C antibody, HIV antibody, Quantiferon Gold tuberculosis testing, complement components 3 and 4, anti-ds DNA, ANA and anti-ENA (Sm, RNP, SSA, SSB), anticardiolipin antibodies, lupus anticoagulant, and anti-beta2-glycoprotein antibodies. A serum or urine pregnancy test will be performed for female subjects at screening, before dosing at baseline and again on days 56 and 84, unless they are postmenopausal (no menstrual period for 2 years or more), had a previous hysterectomy or removal of the ovaries. Testing for BK virus quantitative PCR serum and urine levels will be performed at Screening, day 28, day 56 and day 84. Subjects with positive serum BK virus PCR results at screening will be excluded from the study. Subjects with positive BK virus in urine with undetectable BK virus in serum will be allowed to participate in the study. However, an increase in BK viruria to greater than 10 million copies/ml or development of BK viremia at any of the post randomization visits will lead to study medication discontinuation. Subjects will be asked to complete safety visits and further follow-up will be done under the Natural History Protocol or by their local physicians.

The hematology, chemistry, and urine parameters will be assayed (see Appendix 21.1; Schedule of Events) and are described in detail under Section 4.9 Study Visit Procedures.

5.12 Laboratory Research Studies

Mechanistic studies

Mechanistic studies will be conducted to investigate effects of tofacitinib on intracellular signaling molecules, cytokine expression, circulating immune cell types and autoreactive B cells. Results of these mechanistic studies will be used to compare responses to tofacitinib in those patients with the STAT4 risk alleles to those without STAT4 risk alleles; as well as to the SLE subjects receiving placebo.

All samples for the mechanistic assays will be collected at baseline (prior to treatment), on Day 14, on Day 56 (end of treatment), on Day 84 (end of study) and Unscheduled Visits. These assays will be performed in different laboratories at the NIH and FIMR. To diminish confounding from variability between multiple assays, all of the samples will be treated

appropriately, frozen and stored until all samples can be run simultaneously for each assay at the end of the study.

- The following is a list of the planned mechanistic studies:
  - Ligand-induced STAT phosphorylation in circulating T cells and monocytes using phosphoflow assessment of single cells;
  - Expression of STAT-related target genes; expression of pro-inflammatory and immunoregulatory cytokines in T cells and monocytes using RNA seq;
  - Alteration in the “interferon signature” and the “granulocyte signature” in PBMCs using nanostring; measures of CD3+ T cell expression of IFN regulatory factor-related genes using RNAseq and/or NanoString;
  - Alteration in peripheral blood immune cell populations with special attention to CD4+, CD25+, Foxp3+ regulatory subsets,Th17 cells, and a subset of aberrant neutrophils present in lupus patients (low-density granulocytes)(69). With regards to the latter, assessments will include quantification of LDG levels in peripheral blood and their propensity to form neutrophil extracellular traps (NETs) in the absence of added exogenous stimuli;
  - Measures of serum cytokines (IL-2, 4, 6, 7, 9,12, 15, 17, 23, IFNα and IFNγ);
  - Measurement of pro-inflammatory HDL (piHDL) will be identified with a cell free assay based on the ability of HDL to prevent oxidation.
  - Changes in vascular function, as assessed by the reactive hyperemia index (RHI) using Endopat device, and arterial stiffness using cardio-ankle vascular index (CAVI). Both are surrogate markers of vascular damage and future atherosclerosis development which are amenable to change within this duration of treatment.
  - Additional biomarker studies: Left over whole blood, plasma and serum from the planned mechanistic studies will be frozen and stored for future use.

5.13 Visit Procedures:

5.13.1 ***<u>Pre-screening:</u>***

NIH subjects with SLE who are followed in Protocol 94-AR-0066 will be offered participation in the study if they are found to have mild to moderate disease activity and no obvious exclusion criteria. Pre-screening assessments will be done during routine clinical follow up or initial evaluations and will include review of medical history, physical examination and laboratory assessment and concomitant medications. All of the subjects referred to this study will be enrolled in protocol 94-AR-0066 first.

Patients followed in the Lupus Clinics associated with the FIMR will be offered participation in the study if they are found to have mild to moderate disease activity and no obvious exclusion criteria. These patients will be referred to the NIH for their initial screening and subsequent study-related visits. A summary of the study procedures is in Appendix 21.1.

5.13.2 Screening Period (—45 days to Day 1):

Subjects may be scheduled for screening visits at the NIH Outpatient 9 Clinic or the 5SWS Day Hospital. After informed consent is provided, subjects will undergo screening assessments that include:

- Review of inclusion and exclusion criteria,
- Vital sign measurements,
- Medical history and physical examination,
- EKG,
- Concomitant medications review,
- Assessment of lupus activity (SLEDAI 2K, BILAG 2004, PGA) Laboratory studies:
    - Complete blood count with differential,
    - Serum chemistry panel (creatinine, glucose, urea nitrogen, total bilirubin, alanine aminotransferase, aspartate aminotransferase, alkaline phosphatase),
    - Pregnancy test (serum or urine; for females with reproductive potential only),

- Urinalysis and random urine protein/creatinine ratio,
- Lipid panel
- Lipoprotein Profile
- ANA and anti-ENA (Sm, RNP, SSA, SSB), anticardiolipin antibodies, lupus anticoagulant, and anti-beta2-glycoprotein antibodies,
- Anti-dsDNA antibodies, serum complement components C_3 and C_4,
- Quantitative Immunoglobulins,
- Screening serologies for hepatitis B, hepatitis C and HIV,
- Tuberculosis screening using the Quantiferon Gold test.
- BK virus serum and urine quantitative PCR level
- Albumin

After confirmation of eligibility, the PI or designated AI will notify the statistician associated with the study, who will work in conjunction with the Clinical Center Research Pharmacy to randomize the patient based on presence or absence of STAT 4 risk allele.

5.13.3 Day 1 +/- 7 days Study Visit:

All subjects will be seen in the Clinical Center outpatient clinic or the Clinical Center Day Hospital at the NIH for their visits.

The following procedures will be performed at the Day 1(+/-7 days) visit:

- Concomitant medication review
- Drug Accountability- Dispense 35 day supply of study drug
- Vital sign assessments
- Presenting symptoms, abbreviated medical history, and physical examination

- Laboratory studies:
    - Complete blood count with differential,
    - Serum chemistry panel (creatinine, glucose, urea nitrogen, total bilirubin, alanine aminotransferase, aspartate aminotransferase, alkaline phosphatase),
    - Pregnancy test (urine or serum; for females of reproductive potential only),
    - Urinalysis, random urine protein/creatinine ratio,
    - Lipid panel
    - Lipoprotein Profile
    - Lymphocyte pheno-TBNK
    - Inflammatory markers (hs-CRP, ESR),
    - Anti-dsDNA antibodies, serum complement components C_3 and C_4,
    - Research labs
- Assessment of SLE disease activity (SLEDAI 2K, PGA, DAS-28, CLASI [if indicated]),
- Vascular function studies,
- Patient questionnaires (Multidimensionnel fatigue, SF-36, Patient Global Assessment),
- Study drug administration: A 35 day supply of study drug will be dispensed to subjects on Day 1 with instructions regarding the twice daily dosing regimen and a review of potential side effects. Subjects will take the first dose of study drug in the Clinical Research Center at the NIH. No drug will be dispensed to a female subject of reproductive potential until negative pregnancy test results (serum or urine) have been obtained within 24 hours.
- If a patient is found to have an acute infection at the baseline visit, their next visit will be scheduled for no more than 45 days later, at which time if the infections has cleared up they will start the study medication. We will repeat only baseline clinical labs at this extra visit; we will not repeat vascular function studies or research sample collection.

- If a patient has an abnormal lab result at the baseline visit that requires a repeat, the patients next visit will be scheduled no more than 45 days later, at which time if the abnormal lab has improved or normalized they will start the study medication. We will not repeat vascular function studies or research sample collection.

5.13.4 Day 7 (+/- 7days), Day 21 (+/- 7days), Day 35 (+/- 7days) and Day 49 (+/- 7days) Telephone Calls:

All subjects will receive a telephone call from the study coordinator on Days 7, 21, 35 and 49 (all +/- 7days). The purpose of the calls will be to ascertain any adverse events, review compliance with study drug and with concomitant medications.

5.13.5 Day 14 +/- 7days Study Visit:

All subjects will return to the Clinical Center at the NIH on Day 14 (+/- 7 days) for a brief visit. The following procedures will be performed at this visit:

- Adverse event assessment
- Concomitant medication review
- Vital sign measurements
- Presenting symptoms abbreviated medical history and, physical examination Research labs
- Laboratory studies:
    - Complete blood count with differential,
    - Serum chemistry panel (creatinine, glucose, urea nitrogen, total bilirubin, alanine aminotransferase, aspartate aminotransferase, alkaline phosphatase),
    - Anti-dsDNA antibodies, serum complement components C3 and C4,

- Pregnancy test (urine or serum; for females of reproductive potential only),
- Urinalysis, random urine protein/creatinine ratio,

- Assessment of SLE disease activity (SLEDAI 2K, PGA,CLASI [if indicated], DAS-28)
- Patient questionnaires (Multidimensionnel fatigue, SF-36, Patient Global Assessment)

5.13.6 Day 28 +/- 7 days Study Visit:

All subjects will return to the Clinical Center at the NIH on Day 28 (+/- 7 days) for a brief visit and they will be asked to bring their bottles of study drug with them. The following procedures will be performed at this visit:

- Adverse event assessment
- Concomitant medication review
- Drug accountability-Returning used drug bottle
- Drug accountability- Dispense 35 day supply of study drug
- Vital sign measurements
- Presenting symptoms abbreviated medical history
- Physical examination
- Laboratory studies:
    - Complete blood count with differential,
    - Serum chemistry panel (creatinine, glucose, urea nitrogen, total bilirubin, alanine aminotransferase, aspartate aminotransferase, alkaline phosphatase),
    - Pregnancy test (urine or serum; for females of reproductive potential only),
    - Urinalysis, random urine protein/creatinine ratio,

  - Anti-dsDNA antibodies, serum complement components C_3 and C_4 quantitative Immunoglobulins,
  - BK virus serum and urine quantitative PCR level
  - Albumin
  - Inflammatory markers (ESR)

- Assessment of SLE disease activity (SLEDAI 2K, BILAG 2004, PGA, CLASI [if indicated], DAS-28)
- Patient questionnaires (Multidimensionnel fatigue, SF-36, Patient Global Assessment).

5.13.7 Day 42 +/- 7 days Study Visit:

All subjects will return to the Clinical Center at the NIH on Day 42 (+/- 7 days) for a brief visit. The following procedures will be performed at this visit:

- Adverse event assessment
- Concomitant medication review
- Vital sign measurements
- Presenting symptoms abbreviated medical history
- Physical examination
- Laboratory studies:
  - Complete blood count with differential,
  - Serum chemistry panel (creatinine, glucose, urea nitrogen, total bilirubin, alanine aminotransferase, aspartate aminotransferase, alkaline phosphatase),
  - Pregnancy test (urine or serum; for females of reproductive potential only),
  - Urinalysis, random urine protein/creatinine ratio,

  - Anti-dsDNA antibodies, serum complement components C3 and C4,
  - Quantitative Immunoglobulins,

- Assessment of SLE disease activity (SLEDAI 2K, PGA, CLASI [if indicated], DAS-28)
- Patient questionnaires (Multidimensionnel fatigue, SF-36, Patient Global Assessment)

5.13.8 Day 56 +/- 7 days Study Visit (end of treatment period):

All subjects will be seen again in the Clinical Center Day hospital or outpatient clinic at the NIH for this visit and they will be asked to bring their bottles of study drug with them. Subjects will be asked to fast after midnight the night prior for the fasting lipid profile and the following procedures will be performed at this visit:

- Adverse event assessment
- Concomitant medication review
- Drug accountability-Returning used drug bottle
- Vital sign measurements
- Presenting symptoms, abbreviated medical history, and physical examination Laboratory studies:
  - Complete blood count with differential,
  - Serum chemistry panel (creatinine, glucose, urea nitrogen, , total bilirubin, alanine aminotransferase, aspartate aminotransferase, alkaline phosphatase),
  - Pregnancy test (urine or serum; for females of reproductive potential only),
  - Urinalysis, random urine protein/creatinine ratio,
  - Inflammatory markers (hsCRP, ESR),
  - Anti-dsDNA antibodies, serum complement components C_3 and C_4,
  - Quantitative Immunoglobulins,

        - Lipid panel,
        - Lipoprotein Profile
        - Lymphocyte pheno-TBNK
        - BK virus serum and urine quantitative PCR level
        - Research labs,
        - Albumin

    - EKG,

- Assessment of SLE disease activity (SLEDAI 2K, BILAG 2004, PGA, CLASI [if indicated], DAS-28)
- Patient questionnaires (Multidimensionnel fatigue, SF-36, Patient Global Assessment)
- Vascular function studies.

5.13.9 Day 84 +/- 7 days (follow up visit, end of study):

All subjects will return to the Clinical Center outpatient clinic or Day hospital at the NIH for the Day 84(+/- 7 days) visit that will serve as the final visit to evaluate for any toxicity and/or increased disease activity after discontinuation of study drug. Subjects will be asked to fast after midnight the previous night for the fasting lipid profile and the following procedures will be performed at this visit:

- Adverse event assessment
- Concomitant medication review
- Vital sign measurements
- Presenting symptoms, abbreviated medical history, and physical examination
- Laboratory studies:
    - Complete blood count with differential,

  - Serum chemistry panel (creatinine, glucose, urea nitrogen, total bilirubin, alanine aminotransferase, aspartate aminotransferase, alkaline phosphatase),
  - Pregnancy test (urine or serum; for females of reproductive potential only),
  - Urinalysis, random urine protein/creatinine ratio,
  - Inflammatory markers (hsCRP, ESR),
  - Anti-dsDNA antibodies, serum complement components C_3 and C_4,
  - Quantitative Immunoglobulins,
  - Lipid panel,
  - Lipoprotein Profile
  - Lymphocyte pheno-TBNK
  - BK virus serum and urine quantitative PCR level
  - Research labs
  - Albumin

- EKG,
- Assessment of SLE disease activity (SLEDAI 2K, BILAG 2004, PGA, CLASI [if indicated], DAS-28)
- Patient questionnaires (Multidimensionnel fatigue, SF-36, Patient Global Assessment).
- Vascular function studies.

5.13.10 Unscheduled Visits:

In the event of an adverse event or other reason that results in an unscheduled visit, reasonable measures should be taken to try and obtain the following information:

- Adverse event assessment (AE)
- Concomitant medication review
- Vital sign measurements
- Presenting symptoms, abbreviated medical history, and physical examination Laboratory studies:
    - Complete blood count with differential,
    - Serum chemistry panel (creatinine, glucose, urea nitrogen, total bilirubin, alanine aminotransferase, aspartate aminotransferase, alkaline phosphatase),
    - Pregnancy test (urine or serum; for females of reproductive potential only),
    - Urinalysis, random urine protein/creatinine ratio,
    - Inflammatory markers (hsCRP, ESR),
    - Anti-dsDNA antibodies, serum complement components C_3 and C_4,
    - Quantitative Immunoglobulins,
    - Lipid panel,
    - Lipoprotein Profile
    - Lymphocyte pheno-TBNK
    - Albumin
- EKG,
- Research labs,

- Assessment of SLE disease activity (SLEDAI 2K, BILAG 2004, PGA, CLASI [if indicated], DAS-28)
- Patient questionnaires (Multidimensionnel fatigue, SF-36, Patient Global Assessment).

5.14 Follow-up/Termination Procedures:

During the study, subjects will continue to receive regular medical care from their primary physicians. Any AEs reported will be followed by the investigators as described in Section 0. Following completion of the study, subjects will return to the care of their referring physicians. Subjects who received treatment under protocol 94-AR-0066 may continue to be managed under that protocol. A summary of the subjects' course in the study will be sent to the referring physician, if applicable.

Subjects who withdraw or are withdrawn from the study after receiving study treatment will be asked to complete the Day 56 (end of treatment) and the Day 84 follow-up assessments. Subjects who withdraw or are withdrawn before Day 1 will resume care with their referring physician with no further evaluation in this protocol.

5.15 Study Drug, Dosing and Administration:

5.15.1 Tofacitinib:

Tofacitinib (XELJANZ®; Pfizer) is a Janus kinase (JAK) inhibitor. JAKs are intracellular enzymes that transmit signals arising from cytokine or growth factor-receptor interactions on immune cell membranes to influence cellular processes of hematopoiesis and immune cell function. Within the signaling pathway, JAKs phosphorylate and activate Signal Transducers and Activators of Transcription (STATs) which modulate intracellular activity including gene expression. Tofacitinib modulates the signaling pathway at the point of JAKs, preventing the phosphorylation and activation of STATs. XELJANZ® is the citrate salt of tofacitinib. Tofacitinib citrate is a white to off-white powder with the following chemical name: (3R,4R)-4-methyl-3-(methyl-7H-pyrrolo [2,3-d]pyrimidin-4-ylamino)-β-oxo-1-piperidinepropanenitrile, 2-hydroxy-1,2,3-propanetricarboxylate (1:1) It is freely soluble in water and has a molecular weight of 504.5 Daltons. XELJANZ® is supplied for oral administration as 5 mg tofacitinib

(equivalent to 8 mg tofacitinib citrate) white round, immediate-release film-coated tablet. Each tablet of XELJANZ® contains the appropriate amount of XELJANZ ®as a citrate salt and the following inactive ingredients: microcrystalline cellulose, lactose monohydrate, croscarmellose sodium, magnesium stearate, HPMC 2910/Hypromellose 6cP, titanium dioxide, macrogol/PEG3350, and triacetin.

5.15.2 Tofacitinib dosing:

The package insert with the recommended dosing is attached as Appendix 22.10. Each subject will be dosed with 5 mg (1 tablet) twice daily for 56 days as is recommended for the treatment of rheumatoid arthritis. Tofacitinib tablets will be supplied by Pfizer Incorporated to the NIH Clinical Center Research Pharmacy.

5.15.3 Placebo:

Placebo tablets will be supplied by Pfizer Incorporated to the NIH Clinical Center Research Pharmacy.

5.16 Treatment Compliance:

Compliance with study drug dosing will be assessed at the Day 28 and 56 (+/- 7 days) visits. Subjects will be asked to bring their bottles for a pill count that will be recorded. Subjects who have demonstrated less than 80% compliance at the Day 28 and 56 (+/- 7 days) visits will be withdrawn from the study. The compliance with study medication will be determined by the principal investigator based on multiple factors including but not limited to pill count of the returning bottles, subject's overall compliance with study procedures, subject's self-reporting of compliance and subject's reliability based on prior interactions. Subjects withdrawn from the study after receiving study treatment will be asked to follow up to complete the Day 56 (+/-7 days) and Day 84(+/-7 days) follow-up assessments. Study treatment compliance will be assessed on telephone calls on day 7, 21, 35, and 49 and during clinic visits on day 14, 28, 42, and 56. Missing study medication doses are a possible occurrence during the 56-day dosing period and this will be documented at each compliance check, but will not result in a protocol deviation. However, if the subject is determined to be noncompliant in the opinion of the principal investigator, then the subject will be withdrawn from the study.

5.17 Treatment Allocation:

5.17.1 Genotyping and Blinding:

Most of the SLE patients followed in the Lupus Clinics at the FIMR have agreed to participate in the “Rheumatology Clinical Database and Specimen Bank” protocol at the FIMR. As part of this protocol, blood samples have been collected and stored for DNA analysis. Similarly, NIH subjects followed in the "Studies of the Pathogenesis and Natural History of Systemic Lupus Erythematosus" protocol (94-AR-0066) have also provided consent for DNA analysis. These blood samples will be analyzed for STAT4 risk alleles in Dr. Peter Gregersen’s laboratory at the FIMR.

The results indicating the presence or absence of STAT 4 risk alleles will be communicated to the NIH Clinical Center Research Pharmacy. As subjects who are followed in these observational cohorts experience disease flares and become eligible for participation in this study, investigators will contact the statistician associated with the study who will work in conjunction with the NIH Clinical Center Research Pharmacy during the screening process to determine eligibility based on STAT4 risk alleles. Clinical Center Research Pharmacy will work in conjunction with the statistician associated with the study to ensure that a minimum of 10 eligible subjects are either heterozygous or homozygous for the STAT4 risk allele and that a minimum of 10 eligible subjects do not have the risk allele. The placebo group may or may not have STAT4 risk alleles. This will help to maintain the blind for the investigators and study subjects.

5.17.2 Randomization and Blinding:

Randomization will be done by the NIH Clinical Research Center Pharmacy in conjunction with the statistician associated with the study. This is a two arm study and subjects will be randomized to either tofacitinib or placebo in a 2:1 ratio so that 10 subjects with STAT 4 risk alleles and 10 subjects without STAT 4 risk alleles are in the tofacitinib treatment group. The genotype for the placebo group (n=10) is not pertinent to the randomization as these subjects may or may not have the risk alleles. Both subjects and investigators will be blinded to treatment allocation during the first phase of the study. Unblinding will occur for the analyses after the last subject has completed Day 84 of the study. We will inform the subjects of their

treatment allocation after the last subject has completed Day 84 of the study. We will send subjects a notice of treatment allocation letter through certified mail. See Appendix 22.14.

The blind will be held by the NIH Clinical Center Research Pharmacy as well as the statistician associated with the study. In cases where breaking the blind is necessary to provide clinical care to the patient (as determined by the physician involved in patient's care and principal investigator), the principal investigator will contact the NIH Clinical Center Research Pharmacy and provide the treatment allocation information to the treating physician. Such subject(s) would be withdrawn from the study and followed up as described in section 4.10 above. The Data and Safety Monitoring Committee (DSMC) may also request unblinding of treatment allocation or group assignment. These requests will be transmitted to the NIH Clinical Center Research Pharmacy by the principal investigator. The data will be provided directly to the Chair of DSMC without unblinding the investigators before the DSMC determines a plan of action.

5.18 Concomitant medications:

5.18.1 Restricted medications:

The following medications are allowed provided that they are administered at stable doses during the study:

- Antimalarial (hydroxychloroquine 400 mg/day or ≤6.5 mg/kg/day, if more than 400 mg/day, chloroquine phosphate 500 mg daily, quinacrine 100 mg daily); must already be on this stable dose of this medication 12 weeks prior to study entry.
- Prednisone or equivalent steroid dose up to 20 mg /day must already be on this stable dose of this medication 4 weeks prior to study entry.
- ACE inhibitors or ARB medications, must already be on this stable dose of this medication 4 weeks prior to study entry.
- Lipid lowering medications if initiated at least 6 months prior to the screening visit. The dose of any of these medications can be decreased (temporally or for the duration of the study) if clinically indicated for toxicity.

5.18.2 Disallowed medications:

The medications and vaccines listed below will not be allowed during treatment. Any clinical indication requiring treatment with medications listed below will lead to cessation of study

treatment. Withdrawn subjects will be asked to return according to the follow up phase of the study and to complete all study procedures.

- Rituximab.
- Any other biologic agent including TNF-α blockers, IL-1 blocking agents, anti-IL-6 agents, belimumab, abatacept.
- Cyclophosphamide.
- Azathioprine.
- Mycophenolate mofetil.
- Rapamycin.
- Cyclosporine.
- Tacrolimus.
- Methotrexate.
- Administration of any live virus vaccines.

5.18.3 **Corticosteroids**:

Subjects may be on prednisone ≤ 20 mg/day at study entry. Prednisone may be increased as indicated after the end of the treatment period on Day 56 (+/- 7 days). At the investigator's discretion, glucocorticoids may be tapered during the study.

5.19 Treatment of flares:

Subjects entering the study will have mild to moderate disease activity. Based on the known response to tofacitinib in RA, animal studies and in cellular assays, we expect a similar rapid response in SLE subjects. Nonetheless, flares that escalate despite treatment with tofacitinib to merit treatment with corticosteroids, increasing current dose of corticosteroid, and/or the addition of another DMARD will meet criteria for subject termination from the study.

5.20 Withdrawal criteria:

Subjects will be informed that they may withdraw or be excluded from the study at any time. The following conditions will require the discontinuation of study drug:

1. Request by subject to be withdrawn from the study.
2. Flares not responding to the treatment above or if, in the opinion of the responsible investigator, the subject needs immediate immunosuppressive therapy that is not allowed in the protocol.
3. Any Grade 4 adverse event that is unexpected and at least possibly related to study drug.
4. More than 45 days delay in treatment after the screening visit.
5. More than 1 "no-show" for a visit.
6. Persistent elevation of LFTs of > 2 times upper limit of normal persistently present on repeated samples 2 weeks +/- 7 days apart.
7. >30% increase in serum creatinine from baseline at enrollment, persistently present on repeated samples 2 weeks +/- 7 days apart.
8. Increase in proteinuria at the time of screening visit to protein to creatinine ratio of more than 1.000 mg/mg or more than 1000 mg in a 24 hours urine collection, persistently present on repeated samples 2 weeks +/- 7 days apart.
9. Cellular casts ≥ 2+ persistently present on repeated samples 2 weeks +/- 7 days apart, (Criteria #7, 8, and 9 were chosen as withdrawal criteria as they would be indicative of active lupus nephritis requiring aggressive immunosuppressive therapy).
10. Any other reason that, in the opinion of the responsible Investigator, poses unacceptable risk to the subject.
11. Infections requiring intravenous antibiotic treatment.
12. Development of any malignancy.
13. urine BK virus level of more than 10 million copies/ml by quantitative PCR or detection of BK virus in serum by quantitative PCR.

6 Risks and Discomforts:

6.1 Unexpected Adverse Events:

Unforeseen adverse effects are a risk with every new drug treatment, including new indications for approved drugs. The risks of participating in this study are reasonable relative to the potential health benefits and generalizable medical knowledge that may be obtained.

6.2 Risks/discomfort associated with tofacitinib:

Tofacitinib is approved by the Food and Drug Administration (FDA) for treatment of rheumatoid arthritis. It is also being tested for use in other diseases such as psoriasis and inflammatory bowel disease and to prevent transplant rejection. Though generally well-tolerated in patients with Rheumatoid Arthritis, tofacitinib has potential risks associated with its use. The data on adverse events associated with exposure to tofacitinib are gathered from two Phase II and 5 Phase III multi-center, double-blind, placebo controlled clinical trials where subjects were randomized to tofacitinib 5 mg twice daily, 10 mg twice daily or placebo, with or without concomitant disease modifying drugs (DMARDS); most commonly methotrexate. Adverse events associated with tofacitinib included the following:

6.2.1 Infections

In the rheumatoid arthritis (RA) studies, the overall frequency of infections was 20% in the patients who received tofacitinib 5 mg twice daily and 18% in the patients who received placebo. The most commonly reported infections were upper respiratory infections, throat infections and urinary tract infections.

The most serious side effects reported with tofacitinib in RA patients were serious infections due to bacterial, viral or fungal organisms. These occurred in 11 out of 2685 patients (0.4%) who received the drug for 3 months or less. For patients who experienced 0-12 months of exposure to tofacitinib, serious infections were reported in 34 patients on the 5 mg twice daily dose and in 33 subjects on the 10 mg twice daily dose. The most common serious infections were pneumonia, cellulitis, herpes zoster, and urinary tract infections. Tuberculosis was not reported in patients on any dose of tofacitinib for less than 3 months. During 0-12 months of exposure to tofacitinib, tuberculosis cases were reported in 0 patients receiving the 5 mg twice daily dose and 6 patients who received the 10 mg twice daily dose. Opportunistic infections were not reported in patients receiving any dose of tofacitinib for less than 3 months. During 0-12 months of exposure, opportunistic infections were reported in 4 patients receiving 5 mg twice daily dosing and 4 patients receiving 10 mg twice daily dosing; the median range for tofacitinib exposure prior to diagnosis of an opportunistic infection was 8 months. These opportunistic infections included

cryptococcus, pneumocystis, herpes virus, cytomegalovirus (CMV) and BK virus and most occurred in patients that were also taking corticosteroids and methotrexate.

6.2.2 Gastrointestinal Perforation

Rare events of gastrointestinal perforation have been reported in clinical studies of tofacitinib in RA patients although the role of JAK inhibition in these events is not known.

6.2.3 Malignancy

Malignancies were observed in clinical studies of tofacitinib. In the seven controlled RA clinical studies, 11 solid cancers and one lymphoma were diagnosed in 3328 patients receiving tofacitinib with or without methotrexate, compared to 0 solid cancers and 0 lymphomas in 809 patients in the placebo with or without methotrexate group during the first 12 months of exposure. Lymphomas and solid cancers have also been observed in the long-term extension studies in RA patients treated with tofacitinib. Two of these 11 malignancies occurred in the first 3 months of exposure to tofacitinib. The most common types of malignancies were lung and breast cancer followed by gastric, colorectal, renal cell, prostate cancer and malignant melanoma. In Phase 2B, controlled dose-ranging trials in de-novo renal transplant patients, all of whom received induction therapy with basiliximab, high dose corticosteroids, and mycophenolic acid products, Epstein Barr virus-associated post-transplant lymphoproliferative disorder was observed in 5 out of 218 patients treated with tofacitinib (2.3%) compared to 0 out of 111 patients treated with cyclosporine.

6.2.4 Laboratory Abnormalities

6.2.4.1 **Lymphopenia:** Lymphopenia below 500 cells/mm^3 occurred in0.04 % of patients in both dosing groups of tofacitinib within the first 3 months. Low lymphocyte counts (<500 cells/mm^3) were associated with increased risk for serious infections.

6.2.4.2 Neutropenia: Neutropenia with an ANC less than 1000 cells/mm^3 occurred in 0.07% of patients in both dosing groups of tofacitinib during the first 3 months of exposure. No ANCs less than 500 cells/mm3 were reported and

there was no clear association between neutropenia and risk of infection. The neutropenia was dose related and reversible.

6.2.4.3 Anemia: Although JAK inhibition has potential to affect hematopoiesis through interruption of cytokine-induced regulation of hematopoiesis, minimal effects of tofacitinib 5 mg twice daily have been observed in the clinical trials in RA. Mild fluctuations in hemoglobin were not different from those observed in the placebo group.

6.2.4.4 Liver Function Tests: Increased liver enzyme tests greater than 3 times the upper limit of normal were seen in approximately 1.2% of patients overall. No differences in the incidence of AST or ALT elevations were observed between the placebo, 5 mg and 10 mg twice daily groups in the first three months.

6.2.4.5 Lipids: Dose related elevations of lipid parameters were observed at one month of exposure and remained stable thereafter. These were:

- a mean increase of LDL by 15% in the 5 mg twice daily arm and 19% in the 10 mg twice daily arm, and
- a mean increase in HDL by 10 % in the 5 mg twice daily arm and 12% in the 10 mg twice daily arm.

However, mean LDL/HDL ratios were unchanged in patients treated with tofacitinib.

6.2.4.6 Serum Creatinine: In a pooled analysis of 5 phase 3 and 2 long term extension studies of tofacitinib in subjects with RA increase in serum creatinine were seen predominantly in the first 3 months of treatment. The serum creatinine increases at Month 3 were 0.07 and 0.08 mg/dl for 5 and 10 mg BID tofacitinib doses, respectively, compared with 0.04 mg/dl in the placebo. In the tofacitinib 5 mg BID group, 17/1,220 (1.4%) patients had a confirmed serum creatinine increase of ≥33% from baseline in Months 0 to 3. Of these patients, only 2 patients had serum creatinine elevation above the reference range for normal. In the tofacitinib 10 mg BID group, 23/1,217 (1.9%) patients had a confirmed serum creatinine increase ≥33% from baseline in

Months 0 to 3. Of these 23 patients, only 4 had serum creatinine above the reference range for normal. No continued worsening of serum creatinine was noted during the long term follow-up studies. The published data revealed that these changes plateaued or reversed in long term follow up.

6.2.5 Other adverse reactions:

Other adverse reactions reported with tofacitinib at doses of 5 mg twice a day include diarrhea (4%), nasopharyngitis 3.8%), upper respiratory infections (4.5%, headache (4.3%), high blood pressure (1.6%). None of these occurred significantly more frequently than in the patients who were treated with placebo.

6.2.6 Medication interactions

Tofacitinib exposure is increased when tofacitinib is co-administered with moderate and potent inhibitors of cytochrome P450 (CYP) 3A4 (e.g., ketoconazole) or CYP2C19 (e.g., fluconazole). Potent CYP3A4 inducers (e.g., rifampin) will decrease exposure to tofacitinib.
The risk of increased immunosuppression is enhanced if tofacitinib is co-administered with potent immunosuppressive drugs such as azathioprine, tacrolimus, cyclosporine.

6.3 Flare of SLE:

There are no data about the use of tofacitinib in patients with SLE. Tofacitinib may be ineffective or may even worsen lupus. The potential impact of flares is minimized in this study by close monitoring of subjects and strict withdrawal criteria for flares.

6.4 Risks associated with study procedures:

6.4.1 Blood draws

Subjects may experience discomfort, bleeding, or bruising at the venipuncture site, which should resolve with time. There is also a small risk of fainting. Infection at the site of needle insertion may also occur but is rare with the use of sterile disposable needles and aseptic technique.

The amount of blood drawn for clinical care indication and research purposes will be kept within the NIH guidelines of 10.5 mL/kg or 550 mL, whichever is smaller, over any 8 week period for adults.

6.4.2 Electrocardiogram (ECG)

There is no clinically significant risk associated with this procedure. There may be minor discomfort, when the ECG electrodes taped to chest are removed. Rarely, a reaction to the electrodes may cause redness or swelling of the skin.

6.4.3 Vascular Function Studies

Explanation of procedures and risks:

6.4.3.1 Pulse wave analysis (SphygmoCor):

SphygmoCor is a set of non-invasive tools used to determine central blood pressures and arterial stiffness. It (1) derives the pressure wave from the ascending aorta to the carotid artery and (2) gives an accurate measurement of pressure at the heart, brain, and kidneys. However, it cannot be used on patients who may suffer from heart arrhythmias or arterial stenosis. The SphygmoCor consists of the following:

SphygmoCor Px Pulse Wave Analysis System – a diagnostic tool to measure central blood pressure

SphygmoCor Pulse Wave Analysis System – an algorithm used to determine central aortic pressure and visualize ventricular- vascular interactions

SphygmoCor Pulse Wave Velocity System- a tool that derives a pressure pulse waveform using the pressure tonometer and an ECG signal simultaneously. Arterial tonometry uses a pressure sensor to detect the speed of a pulse wave and may indicate a problem in the arteries.

SphygmoCor Pulse Wave Monitoring System– a tool that provides an estimated pressure waveform from the ascending aorta.

6.4.3.2 Cardio-ankle vascular index (CAVI):

For this procedure, placement of ECG electrodes on both wrists and a microphone for phonocardiograph on second intercostal space and 4 blood pressure cuffs wrapped around 4 extremities. Arterial stiffness is calculated followed specified formulas. Main advantage of this procedure versus other procedures to measure arterial stiffness is that it is not altered by blood pressure. In addition, it is simple to perform. All vasodilators, antihypertensives and statins will be held the morning of the test and will be restarted after the procedures are completed. This is in order to avoid having additional variables interfering with the readings of the vascular measurements. The test takes approximately 20 minutes to be performed.

6.4.3.3 **Peripheral arterial tonometry (Endopat):**

EndoPAT™ quantifies the endothelium-mediated changes in vascular tone, elicited by a 5-minute occlusion of the brachial artery (using a standard blood pressure cuff). When the cuff is released, the surge of blood flow causes an endothelium-dependent flow mediated dilatation (FMD). The dilatation, manifested as Reactive Hyperemia, is captured by EndoPAT as an increase in the PAT Signal amplitude. A post-occlusion to pre-occlusion ratio is calculated by the EndoPAT software, providing the EndoPAT index. The test takes approximately 15 minutes to complete, is very easy to perform, and is both operator and interpreter independent. It is a noninvasive test, providing automatic analysis, office-based procedure. The five-minute blood pressure cuff inflation is an accepted standard test to cause reactive hyperemia (the increase of blood flow after a temporary restriction in blood supply) for the assessment of endothelial function. While the occlusion may cause some minor discomfort and tingling in the fingers, the test is absolutely harmless. It is recommended that the patient fast 3 to 8 hours before the test. Subjects will be requested to hold all medications including but not limited to all vasodilator, antihypertensive and statin on the morning of the test. Subjects will be requested to bring their medication with them on the day of the procedure they

will resume their medications right after the completion of procedures. This is in order to avoid any possible confounding variables interfering with the readings of the vascular measurements.

Vascular function studies risk (SphygmoCor, CAVI and Endopat): These procedures are very well tolerated. Other than potential transient minimal discomfort with blood pressure cuff, no side effects are expected.

7 Safety Monitoring (see section 15) :

All subjects will be followed from the time of informed consent until the end of study. At each study visit, subjects will be evaluated for AEs and will be followed until resolution or stabilization of any AEs. All AEs will be graded according to a descriptive severity scale based on the National Cancer Institute Common Toxicity Criteria Version 4.0 (Appendix 21.15). Abnormal findings at the screening visit will be recorded but not considered as adverse events. Concomitant medications will be recorded at the screening visit and assessed at each subsequent visit and telephone monitoring calls.

8 OUTCOME MEASURES:

- Ligand-induced STAT phosphorylation in circulating T cells and monocytes using phosphoflow assessment of single cells measured at baseline, study days 56 and 84.
- Reduced expression of STAT-related target genes; expression of pro-inflammatory and immunoregulatory cytokines in PBMCs, T cells, and monocytes using RNAseq and/or NanoString measured at baseline, study days 7, 56 and 84.
- Alteration in the "interferon signature"; measures of CD3+ T cell expression of IFN regulatory factor-related genes using RNAseq and NanoString between subjects with STAT4 risk allele present and subjects with STAT4 risk allele absent measured at baseline, study days 56 and 84.
- Flow cytometry immunophenotyping to analyze alteration in peripheral blood immune cell populations with special attention to CD4+, CD25+, Foxp3+ regulatory subsets and Th17 cells between subjects with STAT4 risk allele present and subjects with STAT4 risk allele absent at baseline, study days 56 and 84.
- Measures of serum cytokines (IL-2, 4, 6, 7, 9, 12, 15,17, 23, IFNα and IFNγ) at baseline,

study days 56 and 84.

- Reduced IgG autoantibody levels: a statistically significant difference in the change in IgG autoantibody (ANA, anti-dsDNA, anti-Ro, anti-La, anti-Sm, anti-RNP and anticardiolipin antibodies levels) between the subjects with STAT4 risk allele present and subjects with STAT4 risk allele absent at study days 56 and 84.
- Clinical Efficacy: the difference in scores on patient reported outcome measures (SF-36, Fatigue scale and Patient Global Assessment) between subjects on study medications vs. subjects receiving placebo at study days 56 (8 weeks) and 84 (12 weeks).
- Clinical response will be defined as:
    - An improvement in SLEDAI 2K scores of 4 or more from baseline without a worsening (defined as an increase > 0.3) in PGA (physician's global assessment).
- SLE disease flares will be defined as:
    - Mild to moderate flare:
        - an increase in SLEDAI 2K score ≥ 3 but the total score is < 12 or
        - an increase in the PGA > 1 but the total score is < 2.5
    - Severe Flare:
        - a SLEDAI 2K score > 12
        - a PGA>2.5

9 STATISTICAL ANALYSIS

9.1 Analysis Populations:

9.1.1 Safety Population:

The Safety Population will consist of all enrolled subjects receiving at least one dose of study treatment.

9.1.2 Efficacy Population:

The Efficacy Population (EP) will consist of subjects who receive at least 3 doses of study treatment.

9.2 Data Analysis:

Demographic and Baseline Characteristics

Demographic and baseline characteristics will be summarized in tables. Continuous demographic and baseline variables will be summarized as means, medians, standard deviations, minimum values, and maximum values. Categorical demographic (e.g., race) and baseline variables will be summarized as frequencies and percentages.

9.3 Primary outcome:

The primary outcome is to evaluate the safety and tolerance of tofacitinib in patients with SLE. This analysis will include a comparison of rates of adverse events (serious adverse events, Grade 3 and 4 toxicities not fulfilling the criteria for SAE, and non-serious adverse events) and rates of SLE disease flares between the tofacitinib group and the placebo group.

9.4 Secondary outcomes:

Secondary outcomes will be analyzed by comparisons between the treatment (all SLE subjects receiving tofacitinib) and placebo groups and by comparisons between the STAT4 + and STAT4 – groups once the last subject completes the 84 days of study medication.

- Reduced expression of STAT-related target genes; expression of pro-inflammatory and immunoregulatory cytokines in T cells and monocytes using microarray analysis.
- A change in clinical efficacy will be analyzed using the chi-square test comparing the proportions of subjects in the treatment/placebo and STAT4+/STAT4- groups that achieve clinical response at Week 12.
- Differences between the treatment/placebo groups and the STAT4+/STAT4- groups in changes in individual disease activity measures (SLEDAI 2K, PGA) at the end of week 12 will be analyzed as repeated measures with change from baseline as the dependent variable.

9.5 Exploratory Analyses:

Data generated from the exploratory mechanistic studies may not have sufficient power for significance, given the limited number of subjects in this study. Nonetheless, the data generated from differential expression of STAT4 regulated genes in the subjects with STAT4 risk allele present and STAT4 risk allele absent as well as interferon signature in the two groups will be

significant. This approach is likely to be hypothesis generating and serves to seed future studies toward a mechanistic understanding of SLE and the effect of JAK inhibition treatment on the dysregulation of the immune system seen in people with SLE.

9.6 Criteria for Significance:

The number of subjects in this study is unlikely to lead to a statistically significant difference in adverse events between subjects on study medication vs. placebo. Therefore, no formal statistical analysis will be performed for the primary outcome.

Hypothesis tests and confidence intervals of secondary analyses will be either 1- or 2-sided. Results will be considered significant at the $\alpha = 0.05$ level.

9.7 Power Analysis:

This is a pilot, Phase Ib study intended to study predominantly the tolerability/toxicity of tofacitinib. The number of subjects for this study is arbitrary and is based primarily on the conventional numbers in Phase I studies and our experience with similar studies in the past. The primary endpoint is safety and tolerability and 15-20 subjects are commonly used as the sample size for open label safety studies. Our plan to have 20 subjects dosed with tofacitinib to evaluate for safety and a placebo group of additional 10 subjects for comparison is consistent with Phase 1 studies.

9.8 Interim Analysis:

No interim analysis will be performed.

9.9 Accrual Number Request:

We plan to treat 30 subjects with at least 112 doses of study medication each (56 days). Subjects withdrawn before receiving 38 doses of study drug (19 days) for reasons other than drug related adverse events or lack of efficacy will be replaced. Assuming a 25% attrition rate we would like to accrue up to 38 subjects.

10 HUMAN SUBJECTS PROTECTION

10.1.1 Ethics and Good Clinical Practice, Data Collection and Data Quality Assurance:

The study will be conducted according to Good Clinical Practice (GCP) guidelines, the Manual of Procedures (MOP), U.S. 21 CFR Part 50 – Protection of Human Subjects, 21CFR312 subpart D and Part 56 – Institutional Review Boards.

10.1.2 Compliance with Good Clinical Practices:

This trial will be conducted in compliance with the protocol, current GCPs recommended by the International Conference on Harmonization (ICH) and the applicable regulatory requirements for participating institutions. These include the tenets of the Declaration of Helsinki and review and approval by the appropriate ethics review committee or IRBs of participating organizations.

10.2 Data Collection:

Study staff will complete electronic case report forms (eCRFs) that will be compiled and stored in a computerized central database Clinical Trial Data Base (CTDB). Subject electronic medical records in the Clinical Research Information System (CRIS) at the NIH will be used as source documents for these eCRFs. These source documents will be made available upon request for review during site monitoring visits. The eCRF data will be validated via a series of manual edit checks, and all relevant data queries will be raised and resolved on an ongoing basis. Complete, clean data will be locked to prevent further inadvertent modifications. All discrepancies will be reviewed and any resulting queries will be resolved with the investigators and amended in the database. All elements of data entry (i.e., time, date, verbatim text, and the person performing the data entry) will be recorded in an electronic audit trail to allow all data changes in the database to be monitored and maintained in accordance with federal regulations. Security of the database system is maintained through an application firewall, military grade encryption and SSL certificates, with removal of personal identifiers consistent with HIPAA requirements (45 C.F.R. 164.514(a), (b)&(c)).

10.3 Storage of Data and Samples:

10.3.1 Samples:

Research samples collected from subjects consenting to this protocol will be stored in locked secure freezers belonging to NIAMS. The freezers are located in Building 10 at the NIH. The freezers for DNA samples in Dr. Gregersen's lab are located on the 2nd floor of the FIMR and freezers for the Clinical Research Center at the FIMR are located on the first floor. Only study investigators and participating research personnel will have access to the samples. Samples will be kept indefinitely unless there is a significant justification for destroying them. The Principal Investigator will report the loss or destruction of samples collected under this protocol to the Institutional Review Board (IRB).

All samples will be coded and will not have personal identifiers. The codes for identifiers will be contained in a secure electronic database (CTDB) and a subject code log that is maintained in secure research files. An electronic record log with identifiers of all collected research specimens will be kept. These will be stored in secure NIH computers.

Coded samples may be shared with collaborators within and outside the NIH and FIMR. Any remaining samples will be stored in the NIAMS and FIMR locked freezers. Stored samples may be used for studies related to systemic lupus erythematosus. Approval from the IRB will be obtained prior to any research use of stored samples beyond the scope of this study.

All patient samples will be coded and used for research purposes without sharing identifying information and all collaborators will follow federal rules for clinical research.

10.3.2 Data:

Research records and all source documents will be kept in locked cabinets or rooms, and computer research databases will be stored in a secure, password-protected environment, per standard NIH policies. Only study investigators and participating research personnel will have access to the data. The studies done at FIMR will be done on coded samples with no identifiable patient information. Results of the tests will be transmitted to NIH and any data analysis on de-identified samples will be done only at the NIH.

If applicable:

In the future, other investigators (at the NIH) may wish to study these samples and/or data. In that case, IRB approval must be sought prior to any sharing of samples. Any clinical information shared about the sample without patient identifiers would similarly require prior IRB approval.

If applicable:

The research use of stored, unlinked or unidentified samples may be exempt from the need for prospective IRB review and approval. Exemption requests will be submitted in writing to the NIH Office of Human Subjects Research, which is authorized to determine whether a research activity is exempt.

If applicable:

At the completion of the protocol (termination), samples will be retained, and after IRB approval, may be transferred to another existing protocol or a repository.

10.3.3 Record Retention:

The investigators will retain all study-related records for at least 3 years after discontinuation of the study. Some of the research data might be maintained indefinitely for research purposes.

10.3.4 Access to Source Data and Documents:

Medical and research records will be maintained in the strictest confidence. However, as a part of the quality assurance and legal responsibilities of an investigation, the Principal Investigator must permit authorized representatives of the IND sponsor (NIAMS), Leidos Biomedical Research, Inc., CTDB at the NIH, and health authorities to examine (and when required by applicable law, to copy) clinical records for the purposes of quality assurance reviews, audits, and evaluation of the study safety and progress. Unless required by the laws permitting copying of records, only the coded identity associated with documents or other subject data may be copied (obscuring any personally identifying information). Authorized representatives as noted above are bound to maintain the strict confidentiality of medical and research information that may be linked to identifiable individuals. Participating sites will normally be notified in advance of auditing visits.

All subject records and study documentation will be maintained after the protocol is completed. This will include all documentation of AEs, records of study drug receipt and dispensation, and all IRB correspondence. All study records will be kept for at least 3 years after the investigation is completed.

10.3.5 Data Collection, Quality Control and Quality Assurance Monitoring:

The site investigators are required to keep accurate records to ensure the conduct of the study is fully documented. The period of record retention should be consistent with the record retention policies of the sponsoring agency or applicable regulatory agencies.

The site investigators will report all protocol deviations (including those found by study monitors) to their local IRBs as per their policies. The Principal Investigator will review each protocol deviation for potential impact on evaluations of safety and efficacy and subsequent reports will be submitted to the NIAMS/NIDDK IRB as appropriate.

Clinical monitoring for this study will be based on a clinical monitoring plan developed by Leidos Biomedical Research Inc. Clinical Monitoring Research Program (CMRP), Clinical Trials Management (CTM) team in collaboration with the Principal Investigator. The purposes of the clinical monitoring activities are to: 1) to verify the existence of signed informed consent documents and documentation of the ICF process for each monitored subject; 2) to verify the prompt and accurate recording of all monitored data points, and prompt reporting of all SAEs; 3) to compare abstracted information in CTDB with individual subjects' records and source documents (subjects' charts, laboratory analyses and test results, physicians' progress notes, nurses' notes, and any other relevant original subject information);4) to help ensure investigators are in compliance with the protocol, and 5) protocol drug accountability . The monitors will also inspect the clinical site regulatory files to ensure that regulatory requirements (Office for Human Research Protections-OHRP) and applicable guidelines (ICH GCP) are being followed. The clinical monitoring plan will specify the frequency, procedures, and levels of monitoring activities. Some monitoring activities will be performed remotely (e.g., review of regulatory documents), while others will take place on site (e.g., verification of study databases against source documentation). Staff from Leidos Biomedical Research, Inc. CMRP CTM will conduct the monitoring activities and provide follow-up letters describing the findings. The frequency of

reporting for monitoring activities will be specified in the monitoring plan. The Principal Investigator will receive copies of the final follow-up letters.

Study staff will complete electronic case report forms (eCRFs) via a web-based electronic data capture (EDC) system (Clinical Trials Database, CTDB) that is compliant with Part 11 Title 21 of the Code of Federal Regulations. Subject electronic medical records in the Clinical Research Information System (CRIS) will be used as source documents for these eCRFs.

Subjects will also complete periodic questionnaires as outlined in the study procedures through the Clinical Trial Survey System (CTSS) – an ancillary web-based application associated with CTDB. These patient completed questionnaires in CTSS will be used as source documents for the study. The CTSS is accessible outside the NIH and allows subjects to remotely respond to clinical questionnaires with secure passwords administered by CTDB. Security of the database system is maintained through an application firewall, military grade encryption and SSL certificates, removal of personal identifiers consistent with HIPAA requirements (45 C.F.R. 164.514(a),(b)&(c)), and the incorporation of audit trails. The servers are physically located in a secured NIH data center with controlled limited access.

CRFs and patient questionnaire data will be kept in the CTDB database.

The data will be further validated via a series of manual edit checks, and all relevant data queries will be raised and resolved on an ongoing basis. Complete, clean data will be frozen to prevent further inadvertent modifications. All discrepancies will be reviewed and any resulting queries will be resolved with the investigators and amended in the database. All elements of data entry (i.e., time, date, verbatim text, and the person performing the data entry) will be recorded in an electronic audit trail to allow all data changes in the database to be monitored and maintained in accordance with federal regulations.

10.3.6 Review Schedule:

The Leidos Biomedical Research, Inc., will provide monitoring services to assess performance at :

- Initial site monitoring visit: Prior to study start-up for GCP and protocol overview

- Follow-up monitoring visits: Will occur approximately 2-3 times a year based on subject enrollment and follow up visits. The first 3 enrolled subjects will be monitored at 100%. Approximately 10-30% of the remaining enrolled subjects will be randomly selected for monitoring of eligibility criteria, AE/SAE/UP reporting and key data points per PI. All enrolled subject's Informed Consent Documents will be monitored.
- Final monitoring visit: Study close out once all subjects are off study or other study closure event occurs.

10.3.7 Reporting:

Regular monitoring reports will be generated by the Leidos Biomedical Research, Inc., after each visit and will be provided to Principal Investigator (PI). Resolution of queries and outstanding issues or concerns will be the responsibility of the PI.

The PI will be responsible for reporting incidents of IRB non-compliance to the NIH IRB (in compliance with regulations on the protection of human subjects and institutional policy and procedures) and responsible for securing compliance.

10.4 Institutional Review Board:

The principal investigator must provide for the review and approval of this protocol and associated informed consent documents by NIAMS/NIDDK IRB at the NIH. Any amendments to the protocol or consent materials must be approved by the NIAMS/NIDDK IRB before they are placed into use.

The principal investigator will inform the IRB of serious or unexpected AEs that might occur during the study and are likely to affect the safety of the subjects, or the conduct of the study according to the NIH HRPP SOP 16 v2(10/11/13): "Reporting Requirements for Unanticipated Problems, adverse Events and Protocol Deviations". The principal investigator will comply fully with all IRB requirements for both the reporting of AEs, protocol or consent form changes, as well as any new information pertaining with the use of the study medication that might affect the conduct of the study.

10.5 Recruitment of women, children and minorities:

Minors will not be included in this study because this is a Phase Ib study that involves greater than a minor increment over minimal risk without the prospect of direct benefit to subjects based on currently available data.

The sex distribution (female:male) for SLE is 9:1. Lupus is more prevalent in African-Americans and Hispanics. We expect to recruit subjects indicative of this population distribution. If subject enrollment is not indicative of the Lupus population of the United States, then alternative methods for recruitment will be considered such as:

- contacting our referring physicians and requesting the appropriate study subjects and/or
- contacting health professionals in urban tertiary referral centers for African-, Hispanic-, or Asian-American subject recruitment.

10.6 Subject selection:

Pregnant and lactating women will be excluded from the study because the safety of tofacitinib during pregnancy/lactation has not been established. All subjects of childbearing potential will be required to use an effective method of contraception during the study. A pregnancy test will also be performed at pre-screening, during the Screening Period, before each study treatment, and on the follow up visits. Other vulnerable populations (minors and prisoners) will also be excluded from participating in this study. Eligible NIH employees may participate in the study, the specific protections for the NIH employees as vulnerable population is described under section 14 Informed Consent documents and process.

10.7 Qualification of Investigators:

- Dr. Sarfaraz Hasni MD, will be the Principal Investigator (PI) for the NIH site. He is board certified in internal medicine and rheumatology. He is involved with clinical research at the NIH and is currently PI for another SLE treatment protocol.
- Dr. Meggan Mackay MD, will be the Associate Investigator. She is a clinical investigator with experience in the design and conduct of clinical trials and translational studies in lupus.

- Dr. John O'Shea MD, will be the Associate Investigator. He has made significant contributions to the discovery of JAK-STAT pathway and subsequent development of JAK inhibitors.

- Dr. Betty Diamond MD, will be the Associate Investigator. She is a physician-scientist with significant experience in clinical research involving SLE patients.

- Dr. Mariana Kaplan MD, will be lead medical Investigator. She is a physician-scientist with significant experience in clinical research involving SLE patients. She will be one of the investigators responsible for informed consent process.

- Dr. Nehal Mehta, cardiologist at the NHLBI and will be evaluating and interpreting vascular studies.

- Dr. Alan Remaley, is a senior investigator at the NHLBI and will be respponsible for the lipoprotein assays and analysis

- Dr. Peter Gregerson, MD is a collaborator at the FIMR and head of the Center for Genomics. He will supervise the genotyping and microarray studies.

- Dr. Cynthia Aranow will be the associate investigator at the FIMR. She is a clinical investigator with experience in the design and conduct of clinical trials and translational studies.

- Daniella Schwartz M.D. will be the associate investigator at the NIAMS. She will work on the mechanistic studies in the lab of Dr. John O'Shea and will also perform protocol related visits requiring a medical evaluation. She will be one of the investigators responsible for informed consent process.

- Yenealem Temesgen-Oyelakin, BSN, RN, will be the study coordinator. She will be one of the investigators responsible for informed consent process.

- Massimo Gadina, PhD, will be supervising the mechanistic studies.

- Michael Davis, MSN will be the nurse practitioner on the study and will perform the majority of protocol related visits requiring a medical evaluation. He will be

one of the investigators responsible for informed consent process and will be performing vascular function studies.

- Elaine Poncio, BSN, RN, will be the back–up study coordinator. She will be one of the investigators responsible for informed consent process and will be performing vascular function studies.

- Sarthak Gupta M.D., will be the associate investigator at the NIAMS. He will perform protocol related visits requiring medical evaluation. He will be one of the investigators responsible for informed consent process.

- Donald Thomas Jr., M. D., will be an Associate Investigator. He is board certified rheumatologist with special interest in SLE clinical research. He will be referring eligible subjects to the NIH.

- Mohammad Naqi, M.D, will be performing vascular function studies.

- Isabel Ochoa-Navas, is the patient care coordinator at NIAMS. She will schedule patient appointments and help recruit patients.

11 BENEFITS:

There may be no direct benefits to participants. Subjects may benefit from a thorough evaluation by experts in SLE.

12 SIGNIFICANCE TO BIOMEDICAL RESEARCH:

If this regimen is shown to be devoid of any significant toxicities, further efficacy studies will be planned. This agent is not expected to be associated with the most common toxicities of therapies commonly used in the treatment of SLE, such as severe immunosuppression, myelosuppression, amenorrhea and osteoporosis. This study will provide important preliminary information about the effect of JAK-STAT pathway inhibition in SLE patients and may contribute to better understanding of the pathophysiology of SLE.

13 SUMMARY/CLASSIFICATION OF RISK:

This study involves more than minimal risk without the prospect of direct benefit to subjects based on currently available data. There is the prospect of generalizable knowledge and better understanding of the disease process.

14 INFORMED CONSENT DOCUMENTS AND PROCESS:

The principles of informed consent in the current edition of the Declaration of Helsinki, as well as compliance with all IRB requirements, will be implemented in the study, before any protocol-specified procedures are carried out. A standard consent form for subject participation will be provided with the protocol to the IRB and Office of Protocol Services (OPS) of the NIH. Any modifications to the standard information in the template will require review and approval by the IRB. All subjects will receive a consent form that will include the purposes, procedures, benefits, and potential hazards of the study. This information will be reviewed with the subject by either the principal or a qualified associate investigator. All prospective subjects will be given ample time to read the consent form, and ask questions, before signing.

The consent documents will be translated into Spanish. For Spanish speaking subjects the consent will be explained by the PI/AI through an interpreter. All subjects will be informed of their right to withdraw from the study. Translated documents must be certified to contain the complete descriptions provided in the English version of the document. Should we enroll a subject who speaks a language other than Spanish, we will request IRB approval to use the Short Form Consent Process as outlined in SOP 12.9.1, under the provisions of 45 CFR 46.117(b)(2). In accordance with IRB guidance we will obtain IRB approval prior to obtaining informed consent from the potential study participant/s.

For inclusion in the study, each subject will be required to sign the consent form. The original forms will become part of the permanent medical record and kept on file in the subject's study chart, available for inspection by regulatory authorities, both federal and institutional. Copies will be provided to the subjects. The fact that informed consent was obtained prior to the initiation of study procedures will be documented in the subject's medical records.

The principal investigator (PI) will be responsible to ensure that informed consent is obtained consistent with the requirements as outlined in the document HRPP SOP 12, *REQUIREMENTS*

FOR INFORMED CONSENT. The informed consent will be obtained by the PI or designated investigators as listed under section 10.8 Qualification of Investigators.

All the relevant research information necessary to make an informed decision will be disclosed to the prospective subjects. The individual obtaining the informed consent will facilitate the understanding of what has been discussed. The individual obtaining the informed consent will make every effort to minimize any possibility of coercion and undue influence.

All subjects must provide written informed consent to participate and consent will be documented using the most current IRB-approved consent form.

Eligible NIH employees may participate in this study with the protections as outlined below:

Participation of NIH employees:

We anticipate eligible NIH employees may participate in this study. We will follow the Guidelines for the Inclusion of Employees in NIH Research Studies and will give each employee a copy of the "NIH information sheet on Staff Research Participation" as per NIH SOP 14F, "Research Involving NIH Staff as Subjects." If the employee is within the same branch, section, or unit such that the individual obtaining consent is a supervisor then independent monitoring of the consent process through Clinical Center Department of Bioethics Consultation Service will be requested. Study staff will be trained that communication of any personal or medical information about an NIH employee), including the fact that they are participating in this study, should be restricted to those investigators who need to know this information, and such information will not be discussed with anyone outside of the study without permission from the subject.

We will discuss the following applicable safeguards to the eligible NIH employee:

- Unbiased participation for protocol integrity and participant risk assessment.
- Ensure there is no perceived workplace pressure or expectation on either participation or deciding not to participate on the protocol in regards to a benefit or adverse effect on their NIH employment or staff position.
- Protection of privacy and confidentiality will be maintained, but also with acknowledgement of the limits due to sensitive information that may be in their NIH file.
- Discussion of time commitments of the study and compensation in accordance with NIH policy 2300-630-3, *Leave Policy for NIH Employees Participating in NIH Medical Research Studies*.

15 GCP COMPLIANCEAND DATA AND SAFETY MONITORING AND REPORTING:

This section defines the types of safety data that will be collected under this protocol and outlines the procedures for appropriately collecting, grading, recording and reporting that data. Serious adverse events must be reported promptly to the IND sponsor and NIAMS/NIDDK Institutional Review Board (IRB). IRB reporting of AEs will be done as mandated by NIH policies

Information in this section complies with ICH Guideline E2A: Clinical Safety Data Management: Definitions and Standards for Expedited Reporting, ICH Guideline E-6: Guideline for Good Clinical Practice and applies the standards set forth in the NCI CTCAE, version 4 and NIH HRPP SOP 16 v2 (10/11/13): “Reporting Requirements for Unanticipated Problems, adverse Events and Protocol Deviations”. Data for this study will be collected on electronic Case Report Forms (eCRFs) that will be transmitted to the NIH CTDB. The Principal Investigator, Dr. Hasni, will oversee the conduct of the study.

15.1 Clinical Monitoring:

Study procedures will be subject to audits and/or monitoring visits to ensure compliance with the protocol and applicable regulatory requirements consistent with the NIAMS quality assurance program plan. Audit and/or monitoring visit results will be reported to the Principal Investigator for further reporting as appropriate. Study documents and pertinent hospital or clinical records will be reviewed to verify that the conduct of the study is consistent with the protocol plan. The clinical monitoring plan will be developed by the Leidos Biomedical Research, Inc. Clinical Monitoring Research Program (CMRP), Clinical Trials Management Team (CTM) in collaboration with the Principal Investigator. The purposes of the clinical monitoring activities are:

1. to verify the existence of signed informed consent documents and documentation of the ICF process for each monitored subject;
2. to verify the prompt and accurate recording of all monitored data points, and prompt reporting of all SAEs;
3. to compare abstracted information in the electronic database with individual subjects’ records and source documents (subjects’ charts, laboratory analyses and test results, physicians’ progress notes, nurses’ notes, and any other relevant original subject information);
4. to help ensure investigators are in compliance with the protocol.

The monitors will also inspect the clinical site regulatory files to ensure that regulatory requirements (Office for Human Research Protections-OHRP) and applicable guidelines (ICH-GCP) are being followed. Some monitoring activities may be performed remotely (e.g., review of regulatory documents), while others will take place on site (e.g., verification of study databases against source documentation). Staff from Leidos Biomedical Research Inc., will

conduct the monitoring activities and provide follow-up letters describing the findings. The frequency of reporting for monitoring activities will be specified in the monitoring plan.

15.2 Data and Safety Monitoring Committee Oversight:

The NIAMS Data and Safety Monitoring Committee (DSMC) will have safety oversight responsibilities for the study. A separate committee will be formed specifically for this protocol and will include members with expertise in a broad range of areas, including human subjects' protection, research ethics, clinical trial implementation, immunology, and rheumatology. Approximately semi-annually, the DSMC will review data related to enrollment progress, study implementation, subject safety, and protocol violations. The DSMC will primarily review blinded data but the committee chair may also request un-blinding of treatment allocation or group assignment of individual subjects on as needed basis. These requests will be transmitted to the NIH Clinical Center Research Pharmacy by the principal investigator. The data will be provided directly to the Chair of DSMC without un-blinding the investigators before the DSMC determines a plan of action.

The CTDB will generate reports that compile all newly submitted and accumulated AEs, SAEs, toxicities, pregnancies, and concomitant medications. Subsequent review of periodic reports will be performed by the Principal Investigator.

The DSMC will also consider current information from other sources on the biology of the disease and the subject population under study. Based on these reviews, the DSMC will make recommendations to the Principal Investigator and the NIAMS Clinical Director concerning the continuation, modification, or termination of the study. The DSMC will also meet ad hoc if relevant issues arise that require committee review. The roles and responsibilities of committee members and meeting procedures are formally described in the NIAMS Charter.

15.3 Suspension Guidelines:

The DSMC chair will be alerted if any of the following situations occur:

- An unexpected fatal or life-threatening event assessed as related to the use of study drug.
- Three or more subjects with similar severe AEs assessed as related to the use of study drug.

While the DSMC has ultimate authority to establish safety, we have established additional pre-determined stopping rules as defined by the number and type of AEs (Table 2). The Principal Investigator will be responsible for monitoring the accruing safety data related to suspension guidelines and for alerting the DSMC chair when a criterion is met. The DSMC chair will be alerted by email within 7 calendar days of determination that a criterion has been met. The DSMC will issue a recommendation on study continuation to the NIAMS Clinical Director after reviewing data related to the suspension guideline. If the study is stopped, subjects will receive conventional care for any study-related AEs and continue to be followed for clinical and safety outcomes. Otherwise, the study will continue per the DSMC recommendations. The Principal Investigator and the Clinical Director will provide the recommendations of the DSMC to the IRB.

Table 2: Suspension Guidelines for DSMC	
First 5 subjects	Three grade 3 or 4 AEs of same type<br>Five grade 3 or 4 AEs of any type
First 10 subjects	Four grade 3 or 4 AEs of same type<br>Five grade 3 or 4 AEs of any type
All 20 subjects	Eight grade 3 or 4 AEs of same type<br>Ten grade 3 or 4 AEs of any type

16 ADVERSE EVENT AND UNANTICIPATED PROBLEM REPORTING:

The Principal Investigator will be responsible for detecting, documenting, and reporting AEs and SAEs in accordance with the protocol, IRB requirements, and federal regulations.

16.1 Definitions:

16.1.1 Adverse Event:

An adverse event (AE) is any unfavorable and unintended diagnosis, symptom, sign (including an abnormal laboratory finding), syndrome, or disease that either occurs during the study, having been absent at baseline, or if present at baseline, appears to worsen. All AEs will be graded for intensity (severity) and relationship to study drug.

16.1.2 Unanticipated Problem:

The Office for Human Research Protections considers unanticipated problems to be any incident, experience, or outcome that meets all of the following criteria:

- Is unexpected in terms of nature, severity, or frequency given a) the research procedures that are described in the IRB-approved research protocol and informed consent, and b) the characteristics of the subject population being studied;
- Is related or possibly related to participation in the research (possibly related means there is a reasonable possibility that the incident, experience, or outcome may have been caused by the procedures involved in the research); and
- Places subjects or others at a greater risk for physical, psychological, economic, or social harm than was previously known or recognized.

An incident, experience, or outcome that meets the 3 criteria above will generally warrant consideration of substantive changes in order to protect the safety, welfare, or rights of subjects or others. Examples of corrective actions or substantive changes that might need to be considered in response to an unanticipated problem include the following:

- Changes to the research protocol initiated by the investigator prior to obtaining IRB approval to eliminate apparent immediate hazards to subjects.
- Modification of inclusion or exclusion criteria to mitigate the newly identified risks.
- Implementation of additional procedures for monitoring subjects.
- Suspension of enrollment of new subjects.
- Suspension of research procedures in currently enrolled subjects.
- Modification of informed consent documents to include a description of newly recognized risks.
- Provision of additional information about newly recognized risks to previously enrolled subjects.

Per the definition, only a subset of AEs would be further characterized as unanticipated problems. Additionally, there are other sorts of events that, while not AEs, would also be characterized as unanticipated problems (e.g., contaminated study drug).

16.1.3 Serious Adverse Event:

A serious adverse event (SAE) is defined as any untoward medical occurrence that:

- Results in death,
- Is life-threatening (defined as a subject at immediate risk of death at the time of the event; it does not apply to an AE which hypothetically might have caused the death if it were more severe),
- Requires or prolongs hospitalization (i.e. the AE required at least a 24-hour inpatient hospitalization or prolonged a hospitalization beyond the expected length of stay; hospitalizations for elective medical/surgical procedures, scheduled treatments, or routine check-ups are not SAEs by this criterion),
- Results in a congenital anomaly or birth defect (i.e., an adverse outcome in a child or fetus of a patient exposed to the trial drug prior to conception or during pregnancy),
- Causes a persistent or significant disability/incapacity (i.e. the AE resulted in a substantial disruption of the patient's ability to carry out normal life functions), or
- Is any other condition that, in the judgment of the investigator, represents a significant hazard or it does not meet any of the above serious criteria but may jeopardize the patient and may require medical or surgical intervention to prevent one of the outcomes listed above.

16.1.4 Medical Events Not Qualifying as Adverse Events or Serious Adverse Events:

Signs and symptoms of pre-existing medical conditions will not be recorded or reported as AEs or SAEs, unless they represent a clinically significant change from the baseline disease status documented at the Pre-screening Visit. In addition, hospitalization for elective procedures or surgeries will not be considered SAEs, nor will inpatient hospitalizations for convenience.

16.1.5 Clinical Laboratory Test Results Not Qualifying as Adverse Events or Serious Adverse Events:

A clinically significant laboratory result that is present at baseline and does not change significantly during the study will not be reported as an AE or SAE. The clinical significance of a change in a laboratory result will be determined by the investigator.

The Principal Investigator or designated AI will evaluate all clinical laboratory and imaging results for clinically significant abnormalities and document the evaluation in the medical record and case report form. A laboratory abnormality will be documented as an adverse event using the following criteria:

- The abnormality is not already encompassed by a reported adverse event (e.g., elevated AST need not be reported as an AE if Liver Failure has already been reported as an AE).
- The abnormality is considered clinically significant by the Investigator. Clinically significant lab abnormality is defined as meeting the following:
  - Necessitates study drug dosing modification (i.e., dose reduction, interruption or discontinuation); and/or
  - Requires a therapeutic intervention (e.g., concomitant medication, blood transfusion or dialysis); and
  - Is unexplainable by the patient's current and past medical conditions

The Principal Investigator will follow significant abnormalities until they return to baseline or stabilize.

16.1.6 Lupus Flare:

A lupus flare is any significant worsening of the signs, symptoms and laboratory test abnormalities associated with lupus. Any increase in the SLEDAI 2K index of 3 or more will be considered as a SLE flare.

16.1.7 Reporting of Adverse Events, Unanticipated Problems and Protocol Deviations:

16.1.7.1 Intensity of Adverse Event:

The intensity (severity) of AEs and SAEs will be graded according to a descriptive scale based on the National Cancer Institute Common Terminology Criteria for Adverse Events (CTCAE) Version 4.0.

16.1.7.2 Relationship to Study Drug and Procedures:

For all AEs and SAEs, the investigator will provide his best estimate of the causal relationship between the event and study drug, and the causal relationship between the event and study procedures. The degree of certainty about causality will be graded according to the criteria in Table 3.

Table 3. Relatedness of Adverse Event to Intervention

Causality	Description
Not Related Category	
Unrelated	Adverse event is clearly due to extraneous causes (e.g., underlying disease, environment)
Related Category	
Unlikely (must have at least 2)	1) does not have temporal relationship to intervention<br>2) could readily have been produced by the subject's clinical state<br>3) could have been due to environmental or other interventions<br>4) does not follow a known pattern of response to intervention<br>5) does not reappear or worsen with reintroduction of intervention
Possible (must have at least 2)	1) has a reasonable temporal relationship to intervention<br>2) could not readily have been produced by the subject's clinical state<br>3) could not readily have been due to environmental or other interventions<br>4) follows a known pattern of response to intervention
Probable (must have at least 3)	1) has a reasonable temporal relationship to intervention<br>2) could not readily have been produced by the subject's clinical state or have been due to environmental or other interventions<br>3) follows a known pattern of response to intervention<br>4) disappears or decreases with reduction in dose or cessation of intervention
Definite (must have all 4)	1) has a reasonable temporal relationship to intervention<br>2) could not readily have been produced by the subject's clinical state or have been due to environmental or other interventions<br>3) follows a known pattern of response to intervention<br>4) disappears or decreases with cessation of intervention and recurs with re-exposure

16.1.7.3 Expectedness of Adverse Events:

For purposes of regulatory reporting, the medically responsible investigator will determine whether an AE or SAE is expected or unexpected. Expected adverse events are those adverse events that are listed or characterized in the Package Insert or in the Physicians' Desk Reference.

Unexpected adverse events are those not listed in the Package Insert (P.I.) or Physicians' Desk Reference (PDR), published medical literature, protocol, informed consent document or not identified. This includes adverse events for which the specificity or severity is not

consistent with the description in the P.I. or PDR. For example, under this definition, hepatic necrosis would be unexpected if the P.I. or I.B. only referred to elevated hepatic enzymes or hepatitis.

For consistency of labeling and categorizing adverse events, the National Cancer Institute (NCI) Common Terminology Criteria for Adverse Events (CTCAE), Version 4.0 will be used in this study.

Procedures for Reporting

Serious adverse events, unexpected AEs, and unanticipated problems will be reported to the IRB, NIAMS Clinical Director, DSMC and FDA according to the NIH-OHSRP SOP 16, "Reporting Requirements for Unanticipated Problems, Adverse Events and Protocol Deviations.". All patients who receive at least one dose or part of a dose of the trial medication (tofacitinib) and complete a safety follow-up, whether withdrawn prematurely or not, will be included in the safety analyses. All data relating to safety will be listed and summarized separately for the treatment period and for the entire study. All safety reports will be reviewed by the Principal Investigator.

16.1.8 Reporting Timeline:

Adverse events, protocol deviations, unanticipated problems (UP), Unanticipated Adverse Device Effects (UADEs), serious adverse events, sponsor and serious, are defined as described in NIH HRPP SOP 16 ("Reporting Requirements for Unanticipated Problems, Adverse Events and Protocol Deviations."). All adverse events occurring during the study, including those observed by or reported to the research team, will be recorded. Serious unanticipated problems and serious protocol deviations, will be reported to the IRB and CD as soon as possible but not more than 7 days after the PI first learns of the event. Not serious unanticipated problems will be reported to the IRB and CD as soon as possible but not more than 14 days after the PI first learns of the event. Not serious protocol deviations will be reported to the IRB as soon as possible but not more than 14 days after the PI first learns of the event.

Deaths will be reported to the Clinical Director and IRB within 7 days after the PI first learns of the event.

16.1.9 Reporting of Non-Serious Protocol Deviations:

Non-serious protocol deviations will only be reported to the IRB (within 14 days after the PI first learns of the event) if they represent a departure from NIH policies for the conduct of human

subjects research, adversely affect the health care of the subject(s) or compromise the interpretation or integrity of the research. Non-serious protocol deviations that result from normal subject scheduling variations or technical issues associated with sampling that does not impact the health of the subject or the interpretation of the study data will not be reported.

16.1.10 <u>Reporting of Adverse Events</u>:

The PI is responsible for summarizing all serious adverse events and adverse events at least possibly related to the research procedure and interventions at the time of Continuing Review.

16.1.11 <u>Reporting of Deaths:</u>

All deaths that have occurred among study participants since the previous review will be summarized at the time of continuing review.

16.1.12 <u>Reporting Waivers:</u>

Waiver of Reporting to the IRB of anticipated minor protocol deviations, adverse events and deaths due to underlying disease or population under study unless determined to be an Unanticipated Problem.

- The following anticipated minor deviations in the conduct of the protocol will not be reported to the IRB unless they occur at a rate greater than that which is anticipated to occur: Deviation in blood draw or visit dates due to any unexpected closure of federal government due to inclement weather or otherwise, a single missed time-point for blood draw in the mechanistic studies. If the rate of these events exceeds the rate specified by the protocol, the events will be classified and reported as though they are Unanticipated Problems.
- The anticipated non-UP adverse events will not be reported to the IRB unless they occur at a rate greater than that known to occur in patients with SLE. If events are occurring substantially more frequent that would be anticipated in typically treated patients with SLE, they will also be reported to IRB. The following anticipated adverse events will not be reported to the IRB unless they occur at a severity greater than that known to occur in patients taking tofacitinib: mild infections of upper respiratory tract, oropharynx and urinary tract treated with or without oral antibiotics, lymphopenia with absolute lymphocyte count of more than 500 cells/mm^3, neutropenia with an absolute neutrophil count of more than 1000 cells/mm^3, mild anemia with drop in hemoglobin of less than 1.5g/dl,

increased liver enzyme tests which are not greater than 2 times the upper limit of normal, a mean increase in LDL by less than 15 %, and a mean increase in HDL by less than 10%. If the rate of these events exceeds the rate specified in the protocol or investigator's brochure the events will be classified and reported as though they are Unanticipated Problems.

16.2 Adverse Event, Protocol deviation and Unanticipated Problem Assessment and Follow-up:

In the event of an adverse event, protocol deviation and unanticipated problem the first concern will be for the safety of the patients. Investigators are required to collect and document all adverse events (AEs), protocol deviations and unanticipated problems. At each study visit, the Principal Investigator will inquire about the occurrence of AE/SAEs since the last visit, and review any protocol deviations and unanticipated problems. Adverse events (including SAEs), protocol deviations, and unanticipated problems may be discovered through any of these methods:

- Observing the subject.
- Questioning the subject in an objective manner.
- Receiving an unsolicited complaint from the subject.
- Review of all source documentation related to study procedures; abnormal values or results from clinical or laboratory evaluations (including, but not limited to, radiographs, ultrasounds, or electrocardiograms) can also indicate adverse events.

Events will be followed for outcome information until they return to baseline or stabilize. Study-related AEs will be followed and/or treated at the NIH until resolution or stabilization of the AE, after which the subject will be referred to a physician(s) outside of the NIH for care and follow-up.

16.2.1 Adverse Event Recording:

Adverse events will be monitored throughout this study, and these events will be recorded on the appropriate AE eCRF at each visit. The record for each event will include the following information:

- Description of the event.
- Onset and stop dates of the event.

- Seriousness of event.
- Intensity (or severity) of the event.
- Action taken because of the event.
- Relationship of the event to study drug and/or study procedure.
- Outcome of the event.
- Expectedness of the event.

16.2.2 **Pregnancy Reporting and Follow-up:**

This study includes pregnancy information as safety data and pregnancies will be recorded if they begin any time after enrollment. Information about any pregnancy should be reported promptly to the NIH NIAMS/NIDDK IRB, NIAMS Clinical Director, and DSMC on the same timeline as a SAE. All pregnancies identified during the study must be followed to conclusion and the outcome of each must be reported. The investigator should be informed immediately of any pregnancy in a study subject or a partner of a study subject. A pregnant subject should be instructed to stop taking study medication. The investigator should counsel the subject and discuss the risks of continuing with the pregnancy and the possible effects on the fetus. Tofacitinib has a pregnancy risk factor Category C; there are no adequate and well controlled studies in pregnant women. Tofacitinib is fetocidal and teratogenic in rats and rabbits when given at exposures 146 times and 13 times, respectively, the maximum recommended human dose. Monitoring of the pregnant subject should continue until the conclusion of the pregnancy, and a follow-up Pregnancy Monitoring form detailing the outcome of the pregnancy should be submitted to the IRB. When possible, similar information should be obtained for a pregnancy occurring in a partner of a study subject. Information requested about the delivery will include:

- Subject's enrollment ID
- Gestational age at delivery
- Birth weight, length, and head circumference
- Gender
- Appearance, pulse, grimace, activity, and respiration (APGAR) score at 1 minute, 5 minutes, and 24 hours after birth, if available

- Any abnormalities.

  Should the pregnancy result in a congenital abnormality or birth defect, an SAE also must be submitted to the NIH NIAMS/NIDDK IRB using the SAE reporting procedures described above.

16.2.3 Lost to follow up patient reporting:

After three attempts to contact the patient via phone, a certified letter will be sent to notify him or her that they have been withdrawn from the study.

17 ALTERNATIVES TO PARTICIPATION OR ALTERNATIVE THERAPIES:

The alternative to participating in this study is to receive conventional treatment. There are several treatment options available for SLE patients with mild to moderate disease activity. The options include: higher doses of corticosteroids; switching to or adding another immunosuppressant; or using a biologic, such as belimumab, which was recently approved for SLE.

18 CONFIDENTIALITY:

A unique coded study number will be assigned to each subject for data collection. The number will not contain any personal information (dates, age) to further ensure protection. Research records will be kept in locked cabinets or rooms, and computer research databases will be stored on NIH computers, which are password protected and encrypted. Only members of the study staff and monitors will have access to study samples and data. Clinical data will be stored in CRIS at the NIH and will be protected by standard measures.

19 CONFLICT OF INTEREST/TECHNOLOGY TRANSFER:

The NIH guidelines on conflict of interest have been distributed to all investigators.

The NIH and Dr. John O'Shea have a patent related to JAK inhibitors and receive royalties. The NIH and Dr. O'Shea have had a collaborative agreement and development award (CRADA) with Pfizer that pertains to JAK inhibition and tofacitinib. The NIH and Dr. O'Shea have an ongoing CRADA for new JAK inhibitors. None of the other investigators have any financial conflicts of interest to report.

For the purpose of this trial, we have a clinical trial agreement (CTA) with Pfizer Incorporated.

The Principal Investigator will seek prospective and continuing NIH IRB review and approval for research collaborations in which coded samples (for which the investigators maintain the key) are sent to non-NIH investigator(s). He will identify the names of the collaborating researchers and their affiliated institutions.

20 RESEARCH AND TRAVEL COMPENSATION:

Travel reimbursement will be provided in accordance with NIH regulations. Subjects will receive financial compensation for time required to participate in the research as per the guidelines provided in HRPP SOP 13, RECRUITMENT, SELECTION AND COMPENSATION OF RESEARCH SUBJECTS. The subjects will be paid $ 60 at each outpatient visit. In addition subjects will be paid an additional $100 at the end of study visit Day 84 as a study completion bonus. Any subject who drops off the study or withdrawn from the study will be paid for the number of visits completed.

21 REFERENCES:

1. GC T. - Systemic lupus erythematosus. N Engl J Med. 2011;365(22):2110-21. - eng.

2. PJ M. - The JAK-STAT signaling pathway: input and output integration. J Immunol. 2007;178(5):2623-9. - eng.

3. MJ K. - Neutrophils in the pathogenesis and manifestations of SLE. Nat Rev Rheumatol. 2011;7(12):691-9. - eng.

4. Murray PJ. The JAK-STAT signaling pathway: input and output integration. J Immunol. 2007 Mar 1;178(5):2623-9. PubMed PMID: 17312100. Epub 2007/02/22. eng.

5. JJ OS, Holland Sm Fau - Staudt LM, LM S. - JAKs and STATs in immunity, immunodeficiency, and cancer. N Engl J Med. 2013;368(2):161-70. - eng.

6. S W, Yang N Fau - Zhang L, Zhang L Fau - Huang B, Huang B Fau - Tan H, Tan H Fau - Liang Y, Liang Y Fau - Li Y, et al. - Jak/STAT signaling is involved in the inflammatory infiltration of the kidneys in MRL/lpr mice. Lupus. 2010;19(10):1171-80. - eng.

7. Lu LD, Stump KL, Wallace NH, Dobrzanski P, Serdikoff C, Gingrich DE, et al. Depletion of autoreactive plasma cells and treatment of lupus nephritis in mice using CEP-33779, a novel, orally active, selective inhibitor of JAK2. J Immunol. 2011 Oct 1;187(7):3840-53. PubMed PMID: 21880982. Epub 2011/09/02. eng.

8. O'Neill S, Cervera R. Systemic lupus erythematosus. Best Pract Res Clin Rheumatol. 2010 Dec;24(6):841-55. PubMed PMID: 21665130. Epub 2011/06/15. eng.

9. Lo MS, Tsokos GC. Treatment of systemic lupus erythematosus: new advances in targeted therapy. Ann N Y Acad Sci. 2012 Jan;1247:138-52. PubMed PMID: 22236448. Epub 2012/01/13. eng.

10. Aringer M, Smolen JS. Tumour necrosis factor and other proinflammatory cytokines in systemic lupus erythematosus: a rationale for therapeutic intervention. Lupus. 2004;13(5):344-7. PubMed PMID: 15230290. Epub 2004/07/03. eng.

11. Aringer M, Smolen JS. Cytokine expression in lupus kidneys. Lupus. 2005;14(1):13-8. PubMed PMID: 15732282. Epub 2005/03/01. eng.

12. Dean GS, Tyrrell-Price J, Crawley E, Isenberg DA. Cytokines and systemic lupus erythematosus. Ann Rheum Dis. 2000 Apr;59(4):243-51. PubMed PMID: 10733469. Pubmed Central PMCID: PMC1753117. Epub 2000/03/25. eng.

13. Banchereau J, Steinman RM. Dendritic cells and the control of immunity. Nature. 1998 Mar 19;392(6673):245-52. PubMed PMID: 9521319. Epub 1998/04/01. eng.

14. Bennett L, Palucka AK, Arce E, Cantrell V, Borvak J, Banchereau J, et al. Interferon and granulopoiesis signatures in systemic lupus erythematosus blood. J Exp Med. 2003 Mar 17;197(6):711-23. PubMed PMID: 12642603. Pubmed Central PMCID: PMC2193846. Epub 2003/03/19. eng.

15. Vallin H, Blomberg S, Alm GV, Cederblad B, Ronnblom L. Patients with systemic lupus erythematosus (SLE) have a circulating inducer of interferon-alpha (IFN-alpha) production acting on leucocytes resembling immature dendritic cells. Clin Exp Immunol. 1999

Jan;115(1):196-202. PubMed PMID: 9933442. Pubmed Central PMCID: PMC1905199. Epub 1999/02/05. eng.

16. Balomenos D, Rumold R, Theofilopoulos AN. Interferon-gamma is required for lupus-like disease and lymphoaccumulation in MRL-lpr mice. J Clin Invest. 1998 Jan 15;101(2):364-71. PubMed PMID: 9435308. Pubmed Central PMCID: PMC508575. Epub 1998/02/07. eng.

17. Clynes R, Dumitru C, Ravetch JV. Uncoupling of immune complex formation and kidney damage in autoimmune glomerulonephritis. Science. 1998 Feb 13;279(5353):1052-4. PubMed PMID: 9461440. Epub 1998/03/07. eng.

18. Boswell JM, Yui MA, Burt DW, Kelley VE. Increased tumor necrosis factor and IL-1 beta gene expression in the kidneys of mice with lupus nephritis. J Immunol. 1988 Nov 1;141(9):3050-4. PubMed PMID: 3262676. Epub 1988/11/01. eng.

19. Brennan DC, Yui MA, Wuthrich RP, Kelley VE. Tumor necrosis factor and IL-1 in New Zealand Black/White mice. Enhanced gene expression and acceleration of renal injury. J Immunol. 1989 Dec 1;143(11):3470-5. PubMed PMID: 2584702. Epub 1989/12/01. eng.

20. Lemay S, Mao C, Singh AK. Cytokine gene expression in the MRL/lpr model of lupus nephritis. Kidney Int. 1996 Jul;50(1):85-93. PubMed PMID: 8807576. Epub 1996/07/01. eng.

21. Herrera-Esparza R, Barbosa-Cisneros O, Villalobos-Hurtado R, Avalos-Diaz E. Renal expression of IL-6 and TNFalpha genes in lupus nephritis. Lupus. 1998;7(3):154-8. PubMed PMID: 9607638. Epub 1998/06/02. eng.

22. Malide D, Russo P, Bendayan M. Presence of tumor necrosis factor alpha and interleukin-6 in renal mesangial cells of lupus nephritis patients. Hum Pathol. 1995 May;26(5):558-64. PubMed PMID: 7750940. Epub 1995/05/01. eng.

23. Chan RW, Lai FM, Li EK, Tam LS, Chow KM, Li PK, et al. The Effect of immunosuppressive therapy on the messenger RNA expression of target genes in the urinary sediment of patients with active lupus nephritis. Nephrol Dial Transplant. 2006 Jan 31. PubMed PMID: 16449281.

24. Iwano M, Dohi K, Hirata E, Kurumatani N, Horii Y, Shiiki H, et al. Urinary levels of IL-6 in patients with active lupus nephritis. Clin Nephrol. 1993 Jul;40(1):16-21. PubMed PMID: 8358870. Epub 1993/07/01. eng.

25. Blomberg S, Eloranta ML, Cederblad B, Nordlin K, Alm GV, Ronnblom L. Presence of cutaneous interferon-alpha producing cells in patients with systemic lupus erythematosus. Lupus. 2001;10(7):484-90. PubMed PMID: 11480846. Epub 2001/08/02. eng.

26. Nurnberg W, Haas N, Schadendorf D, Czarnetzki BM. Interleukin-6 expression in the skin of patients with lupus erythematosus. Exp Dermatol. 1995 Feb;4(1):52-7. PubMed PMID: 7757333. Epub 1995/02/01. eng.

27. Pelton BK, Hylton W, Denman AM. Activation of IL-6 production by UV irradiation of blood mononuclear cells from patients with systemic lupus erythematosus. Clin Exp Immunol. 1992 Aug;89(2):251-4. PubMed PMID: 1638768. Pubmed Central PMCID: PMC1554444. Epub 1992/08/01. eng.

28. Ishida H, Muchamuel T, Sakaguchi S, Andrade S, Menon S, Howard M. Continuous administration of anti-interleukin 10 antibodies delays onset of autoimmunity in NZB/W F1

mice. J Exp Med. 1994 Jan 1;179(1):305-10. PubMed PMID: 8270873. Pubmed Central PMCID: PMC2191319. Epub 1994/01/01. eng.

29. Llorente L, Zou W, Levy Y, Richaud-Patin Y, Wijdenes J, Alcocer-Varela J, et al. Role of interleukin 10 in the B lymphocyte hyperactivity and autoantibody production of human systemic lupus erythematosus. J Exp Med. 1995 Mar 1;181(3):839-44. PubMed PMID: 7869046. Pubmed Central PMCID: PMC2191898. Epub 1995/03/01. eng.

30. Edwards CK, 3rd, Zhou T, Zhang J, Baker TJ, De M, Long RE, et al. Inhibition of superantigen-induced proinflammatory cytokine production and inflammatory arthritis in MRL-lpr/lpr mice by a transcriptional inhibitor of TNF-alpha. J Immunol. 1996 Aug 15;157(4):1758-72. PubMed PMID: 8759766. Epub 1996/08/15. eng.

31. Segal R, Dayan M, Zinger H, Mozes E. Suppression of experimental systemic lupus erythematosus (SLE) in mice via TNF inhibition by an anti-TNFalpha monoclonal antibody and by pentoxiphylline. Lupus. 2001;10(1):23-31. PubMed PMID: 11243506. Epub 2001/03/13. eng.

32. Su X, Zhou T, Yang P, Edwards CK, 3rd, Mountz JD. Reduction of arthritis and pneumonitis in motheaten mice by soluble tumor necrosis factor receptor. Arthritis Rheum. 1998 Jan;41(1):139-49. PubMed PMID: 9433879. Epub 1998/01/20. eng.

33. Llorente L, Richaud-Patin Y, Garcia-Padilla C, Claret E, Jakez-Ocampo J, Cardiel MH, et al. Clinical and biologic effects of anti-interleukin-10 monoclonal antibody administration in systemic lupus erythematosus. Arthritis Rheum. 2000 Aug;43(8):1790-800. PubMed PMID: 10943869. Epub 2000/08/16. eng.

34. Fleischmann R, Kremer J, Cush J, Schulze-Koops H, Connell CA, Bradley JD, et al. Placebo-controlled trial of tofacitinib monotherapy in rheumatoid arthritis. N Engl J Med. 2012 Aug 9;367(6):495-507. PubMed PMID: 22873530. Epub 2012/08/10. eng.

35. Kremer JM, Cohen S, Wilkinson BE, Connell CA, French JL, Gomez-Reino J, et al. A phase IIb dose-ranging study of the oral JAK inhibitor tofacitinib (CP-690,550) versus placebo in combination with background methotrexate in patients with active rheumatoid arthritis and an inadequate response to methotrexate alone. Arthritis Rheum. 2012 Apr;64(4):970-81. PubMed PMID: 22006202. Epub 2011/10/19. eng.

36. Riese RJ, Krishnaswami S, Kremer J. Inhibition of JAK kinases in patients with rheumatoid arthritis: scientific rationale and clinical outcomes. Best Pract Res Clin Rheumatol. 2010 Aug;24(4):513-26. PubMed PMID: 20732649. Epub 2010/08/25. eng.

37. van Vollenhoven RF, Fleischmann R, Cohen S, Lee EB, Garcia Meijide JA, Wagner S, et al. Tofacitinib or adalimumab versus placebo in rheumatoid arthritis. N Engl J Med. 2012 Aug 9;367(6):508-19. PubMed PMID: 22873531. Epub 2012/08/10. eng.

38. JJ OS, Laurence A Fau - McInnes IB, IB M. - Back to the future: oral targeted therapy for RA and other autoimmune diseases. Nat Rev Rheumatol. 2013;9(3):173-82. - eng.

39. J W, Silverfield J Fau - Lee EB, Lee Eb Fau - Curtis JR, Curtis Jr Fau - Wood SP, Wood Sp Fau - Soma K, Soma K Fau - Nduaka CI, et al. - Safety and Efficacy of Tofacitinib, an Oral Janus Kinase Inhibitor, for the Treatment of Rheumatoid Arthritis in Open-label, Longterm Extension Studies. J Rheumatol. 2014;41(5):837-52. - eng.

40. Meyer DM, Jesson MI, Li X, Elrick MM, Funckes-Shippy CL, Warner JD, et al. Anti-inflammatory activity and neutrophil reductions mediated by the JAK1/JAK3 inhibitor, CP-690,550, in rat adjuvant-induced arthritis. Journal of inflammation (London, England). 2010;7:41. PubMed PMID: 20701804. Pubmed Central PMCID: PMC2928212. Epub 2010/08/13. eng.

41. Ghoreschi K, Jesson MI, Li X, Lee JL, Ghosh S, Alsup JW, et al. Modulation of innate and adaptive immune responses by tofacitinib (CP-690,550). J Immunol. 2011 Apr 1;186(7):4234-43. PubMed PMID: 21383241. Pubmed Central PMCID: PMC3108067. Epub 2011/03/09. eng.

42. Maeshima K, Yamaoka K, Kubo S, Nakano K, Iwata S, Saito K, et al. The JAK inhibitor tofacitinib regulates synovitis through inhibition of interferon-gamma and interleukin-17 production by human CD4+ T cells. Arthritis Rheum. 2012 Jun;64(6):1790-8. PubMed PMID: 22147632. Epub 2011/12/08. eng.

43. Tanaka Y, Maeshima K, Yamaoka K. In vitro and in vivo analysis of a JAK inhibitor in rheumatoid arthritis. Ann Rheum Dis. 2012 Apr;71 Suppl 2:i70-4. PubMed PMID: 22460142. Epub 2012/04/04. eng.

44. Kim K, Cho SK, Sestak A, Namjou B, Kang C, Bae SC. Interferon-gamma gene polymorphisms associated with susceptibility to systemic lupus erythematosus. Ann Rheum Dis. 2010 Jun;69(6):1247-50. PubMed PMID: 19919944. Epub 2009/11/19. eng.

45. Ronnblom L. The type I interferon system in the etiopathogenesis of autoimmune diseases. Ups J Med Sci. 2011 Nov;116(4):227-37. PubMed PMID: 22066971. Pubmed Central PMCID: PMC3207297. Epub 2011/11/10. eng.

46. Dienz O, Eaton SM, Bond JP, Neveu W, Moquin D, Noubade R, et al. The induction of antibody production by IL-6 is indirectly mediated by IL-21 produced by CD4+ T cells. J Exp Med. 2009 Jan 16;206(1):69-78. PubMed PMID: 19139170. Pubmed Central PMCID: PMC2626667. Epub 2009/01/14. eng.

47. Naka T, Nishimoto N, Kishimoto T. The paradigm of IL-6: from basic science to medicine. Arthritis Res. 2002;4 Suppl 3:S233-42. PubMed PMID: 12110143. Pubmed Central PMCID: PMC3240141. Epub 2002/07/12. eng.

48. Acosta-Rodriguez EV, Napolitani G, Lanzavecchia A, Sallusto F. Interleukins 1beta and 6 but not transforming growth factor-beta are essential for the differentiation of interleukin 17-producing human T helper cells. Nat Immunol. 2007 Sep;8(9):942-9. PubMed PMID: 17676045. Epub 2007/08/07. eng.

49. Crispin JC, Oukka M, Bayliss G, Cohen RA, Van Beek CA, Stillman IE, et al. Expanded double negative T cells in patients with systemic lupus erythematosus produce IL-17 and infiltrate the kidneys. J Immunol. 2008 Dec 15;181(12):8761-6. PubMed PMID: 19050297. Pubmed Central PMCID: PMC2596652. Epub 2008/12/04. eng.

50. Dolff S, Quandt D, Wilde B, Feldkamp T, Hua F, Cai X, et al. Increased expression of costimulatory markers CD134 and CD80 on interleukin-17 producing T cells in patients with systemic lupus erythematosus. Arthritis Res Ther. 2010;12(4):R150. PubMed PMID: 20653937. Pubmed Central PMCID: PMC2945048. Epub 2010/07/27. eng.

51. Yang J, Chu Y, Yang X, Gao D, Zhu L, Wan L, et al. Th17 and natural Treg cell population dynamics in systemic lupus erythematosus. Arthritis and rheumatism. 2009 May;60(5):1472-83. PubMed PMID: 19404966. Epub 2009/05/01. eng.

52. Kastelein RA, Hunter CA, Cua DJ. Discovery and biology of IL-23 and IL-27: related but functionally distinct regulators of inflammation. Annu Rev Immunol. 2007;25:221-42. PubMed PMID: 17291186. Epub 2007/02/13. eng.

53. Edelbauer M, Kshirsagar S, Riedl M, Billing H, Tonshoff B, Haffner D, et al. Activity of childhood lupus nephritis is linked to altered T cell and cytokine homeostasis. J Clin Immunol. 2012 Jun;32(3):477-87. PubMed PMID: 22228566. Epub 2012/01/10. eng.

54. Mok MY, Wu HJ, Lo Y, Lau CS. The relation of interleukin 17 (IL-17) and IL-23 to Th1/Th2 cytokines and disease activity in systemic lupus erythematosus. J Rheumatol. 2010 Oct;37(10):2046-52. PubMed PMID: 20682672. Epub 2010/08/05. eng.

55. Rana A, Minz RW, Aggarwal R, Anand S, Pasricha N, Singh S. Gene expression of cytokines (TNF-alpha, IFN-gamma), serum profiles of IL-17 and IL-23 in paediatric systemic lupus erythematosus. Lupus. 2012 Sep;21(10):1105-12. PubMed PMID: 22759859. Epub 2012/07/05. eng.

56. Kyttaris VC, Zhang Z, Kuchroo VK, Oukka M, Tsokos GC. Cutting edge: IL-23 receptor deficiency prevents the development of lupus nephritis in C57BL/6-lpr/lpr mice. J Immunol. 2010 May 1;184(9):4605-9. PubMed PMID: 20308633. Pubmed Central PMCID: PMC2926666. Epub 2010/03/24. eng.

57. Tsokos GC. Systemic lupus erythematosus. N Engl J Med. 2011 Dec 1;365(22):2110-21. PubMed PMID: 22129255. Epub 2011/12/02. eng.

58. Crotty S. Follicular helper CD4 T cells (TFH). Annu Rev Immunol. 2011;29:621-63. PubMed PMID: 21314428. Epub 2011/02/15. eng.

59. Waite JC, Skokos D. Th17 response and inflammatory autoimmune diseases. Int J Inflam. 2012;2012:819467. PubMed PMID: 22229105. Pubmed Central PMCID: PMC3249891. Epub 2012/01/10. eng.

60. Doreau A, Belot A, Bastid J, Riche B, Trescol-Biemont MC, Ranchin B, et al. Interleukin 17 acts in synergy with B cell-activating factor to influence B cell biology and the pathophysiology of systemic lupus erythematosus. Nat Immunol. 2009 Jul;10(7):778-85. PubMed PMID: 19483719. Epub 2009/06/02. eng.

61. Shah K, Lee WW, Lee SH, Kim SH, Kang SW, Craft J, et al. Dysregulated balance of Th17 and Th1 cells in systemic lupus erythematosus. Arthritis Res Ther. 2010;12(2):R53. PubMed PMID: 20334681. Pubmed Central PMCID: PMC2888202. Epub 2010/03/26. eng.

62. Sewgobind VD, Quaedackers ME, van der Laan LJ, Kraaijeveld R, Korevaar SS, Chan G, et al. The Jak inhibitor CP-690,550 preserves the function of CD4CD25FoxP3 regulatory T cells and inhibits effector T cells. Am J Transplant. 2010 Aug;10(8):1785-95. PubMed PMID: 20626385. Epub 2010/07/16. eng.

63. Crispin JC, Martinez A, Alcocer-Varela J. Quantification of regulatory T cells in patients with systemic lupus erythematosus. J Autoimmun. 2003 Nov;21(3):273-6. PubMed PMID: 14599852. Epub 2003/11/06. eng.

64. Lee JH, Wang LC, Lin YT, Yang YH, Lin DT, Chiang BL. Inverse correlation between CD4+ regulatory T-cell population and autoantibody levels in paediatric patients with systemic lupus erythematosus. Immunology. 2006 Feb;117(2):280-6. PubMed PMID: 16423064. Pubmed Central PMCID: PMC1782210. Epub 2006/01/21. eng.

65. Lourenco EV, La Cava A. Natural regulatory T cells in autoimmunity. Autoimmunity. 2011 Feb;44(1):33-42. PubMed PMID: 21091291. Pubmed Central PMCID: PMC3057884. Epub 2010/11/26. eng.

66. La Cava A. T-regulatory cells in systemic lupus erythematosus. Lupus. 2008 May;17(5):421-5. PubMed PMID: 18490420. Epub 2008/05/21. eng.

67. La Cava A, Ebling FM, Hahn BH. Ig-reactive CD4+CD25+ T cells from tolerized (New Zealand Black x New Zealand White)F1 mice suppress in vitro production of antibodies to DNA. J Immunol. 2004 Sep 1;173(5):3542-8. PubMed PMID: 15322219. Epub 2004/08/24. eng.

68. McMahon M, Grossman J, Skaggs B, Fitzgerald J, Sahakian L, Ragavendra N, et al. Dysfunctional proinflammatory high-density lipoproteins confer increased risk of atherosclerosis in women with systemic lupus erythematosus. Arthritis Rheum. 2009 Aug;60(8):2428-37. PubMed PMID: 19644959. Pubmed Central PMCID: PMC2753974. Epub 2009/08/01. eng.

69. E V, Yalavarthi S Fau - Berthier CC, Berthier Cc Fau - Hodgin JB, Hodgin Jb Fau - Khandpur R, Khandpur R Fau - Lin AM, Lin Am Fau - Rubin CJ, et al. - Netting neutrophils induce endothelial damage, infiltrate tissues, and expose immunostimulatory molecules in systemic lupus erythematosus. J Immunol. 2011;187(1):538-52. - eng.

22 APPENDICES:

22.1 Schedule of Events

Study Procedures and Visits

**Procedures**	**SCR** 45 **days**	**D1**	**D7 t/call**	**D14**	**D21 t/call**	**D28**	**D35 t/call**	**D42**	**D49 t/call**	**D56 -Last dosing day**	**D84 Final f/u**	**UNSCH visit for AE or other reason**
Informed Consent	**X**											
Review of inclusion and exclusion criteria,	**X**											
vital sign measurements	**X**	**X**		**X**		**X**		**X**		**X**	**X**	**X**
Medical history	**X**											
Randomization	**X**											
Presenting Symptoms-Abbreviated Medical History		**X**		**X**		**X**		**X**		**X**	**X**	**X**
Physical examination	**X**									**X**	**X**	**X**
EKG,	**X**	**X**		X		X		X		**X**	**X**	**X**
Concomitant medications review.	**X**	**X**	**X**	**X**	**X**	**X**	**X**	**X**	**X**	**X**	**X**	**X**
Study Drug Administration<br>D1-D56, Dose BID. 1st dose at NIH on D1		**X**	**X**	**X**	**X**	**X**	**X**	**X**	**X**	**X**		
Drug Accountability-Dispensing Study Drug (35 days' supply)		**x**				**x**						
Compliance with study drug- Returning bottle.						**X**				**X**		
Adverse event review (assessed by reviewing medical hx, Physical exam, lab results)<br>from D1-D84.		**X**	**X**	**X**	**X**	**X**	**X**	**X**	**X**	**X**	**X**	**X**
Screening serologies for hepatitis B, hepatitis C and HIV,	**X**											
Screening Tuberculosis using the Quantiferon Gold test.	**X**											
BK virus quantitative PCR- urine and blood	**X**					X				X	X	
**Serologies:** antinuclear antibodies, anti-ENA panel (anti-RNP, anti-SmRNP, anti-SSA, anti-SSB **)** anti-dsDNA antibodies, anticardiolipin antibodies, lupus anticoagulant, anti-beta2-glycoprotein antibodies, C3 complement, C4 complement, Quantitative immunoglobulin IgA, IgG, and IgM	**X**											
anti-dsDNA antibodies, C3 complement, C4 complement,		**x**		**x**		**x**		**x**		**x**	**x**	**x**
Quantitative immunoglobulin IgA, IgG, and IgM						**x**		**x**		**x**		**x**
**complete blood count** with differential	**X**	**X**		**X**		**X**		**X**		**X**	**X**	**X**
**serum chemistry panel (**creatinine, glucose, urea nitrogen, total bilirubin, alanine aminotransferase, aspartate aminotransferase, alkaline phosphatase)	**X**	**X**		**X**		**X**		**X**		**X**	**X**	**X**
**Pregnancy test** (serum or urine; for females with reproductive potential only)	**X**	**X**		**X**		**X**		**X**		**X**	**X**	**X**
**Urinalysis and Random urine Protein/Creatinine ratio**	**X**	**X**		**x**		**X**		**x**		**X**	**X**	**X**
**Lipid Panel** (low density lipoprotein (LDL), high density lipoprotein (HDL), triglycerides (TG) and total cholesterol.	**X**	**X**								**X**	**X**	**X**
**Lipoprotein Profile**	**X**	**X**								**X**	**X**	**X**
**Lymphocyte pheno-TBNK**		**X**								**X**	**X**	**X**
**Inflammatory markers:**<br>(hs-CRP all visits except D28, ESR)		**X**				**X**				**X**	**X**	**X**
**Mineral Panel:**<br>(Albumin)	**X**					**X**				**X**	**X**	**X**
Assessment of biologic effect(s)		*x*								*x*		
Assessment of the effect of STAT4 risk alleles on drug efficacy.		*x*								*x*		
Assessment of durability of the clinical and biologic effects.										*x*	*x*	
Mechanistic studies: (intracellular signaling molecules, cytokine expression, circulating immune cell types and autoreactive B cells.) All samples can be run simultaneously for each assay at the end of the study.		**x**								**x**	**x**	
Assessment of lupus activity (SLEDAI-2K , BILAG 2004, PGA)	**X**											
6. Patient questionnaires<br>1. SF-36,<br>2. Multidimensional Assessment of Fatigue questionnaire and<br>3. Patient Global Assessment Scale. P.16, 31,39		**X**		**X**		**X**		**X**		**X**	**X**	**X**
**Assessment of lupus disease activity**<br>1. SLEDAI-2K<br>2. Physician Global Assessment (PGA)<br>3. Joint count: A 28 tender and swollen joint count (DAS-28)<br>4. Cutaneous Lupus Erythematosus Disease Area and Severity Index (CLASI)		**X**		**X**		**X**		**X**		**X**	**X**	**X**
5. BILAG 2004	**X**					**X**				**X**	**X**	**X**
Vascular Function Studies		**X**								**X**	**X**	
Research labs		**X**		X						**X**	**X**	**X**

22.2 American College of Rheumatology Revised Classification Criteria for Systemic Lupus Erythematosus

Criteria	Definition
Malar rash	Fixed erythema, flat or raised, over the malar eminences, tending to spare the nasolabial folds
Discoid rash	Erythematous raised patches with adherent keratotic scaling and follicular plugging; atrophic scarring occurs in older lesions
Photosensitivity	Skin rash as a result of unusual reaction to sunlight, by patient history or physician observation
Oral ulcers	Oral or nasopharyngeal ulceration, usually painless, observed by a physician
Arthritis	Nonerosive arthritis involving two or more peripheral joints, characterized by tenderness, swelling, or effusion
Serositis	a. Pleuritis—convincing history of pleuritic pain or rub heard by a physician or evidence of pleural effusion or<br>b. Pericarditis—documented by ECG or rub or evidence of pericardial effusion
Renal disorder	a. Persistent proteinuria >0.5 g/day >3+ if quantitation is not performed or<br>b. Cellular casts—may be red blood cell, hemoglobin, granular tubular, or mixed
Neurologic disorder	a. Seizures—in the absence of offending drugs or known metabolic derangements (e.g., uremia, acidosis, or electrolyte imbalance) or<br>b. Psychosis—in the absence of offending drugs or known metabolic derangements (e.g., uremia, acidosis, or electrolyte imbalance)
Hematologic disorder	a. Hemolytic anemia with reticulocytosis, or<br>b. Leukopenia—<4000/mm^3, or<br>c. Lymphopenia—<1500/mm^3, or<br>d. Thrombocytopenia—<100,000/mm^3 in the absence of offending drugs
Immunologic disorder	a. Anti-DNA—antibody to native DNA in abnormal titer, or<br>b. Anti-Sm—presence of antibody to Sm nuclear antigen, or<br>c. Positive finding of antiphospholipid antibodies based on (1) abnormal serum concentration of IgG or IgM anticardiolipin antibodies, (2) positive test result for lupus anticoagulant using a standard method, or (3) false-positive serologic test for syphilis

Criteria	Definition
	known to be positive for at least 6 mo and confirmed by Treponema pallidum immobilization or fluorescent treponemal antibody absorption test
ANA	Abnormal titer of ANA by immunofluorescence or equivalent assay at any point in time and in the absence of drugs known to be associated with drug-induced lupus syndrome

Adapted from Hochberg MC: Updating the American College of Rheumatology revised criteria for the classification of systemic lupus erythematosus. Arthritis Rheum 40:1725, 1997.

22.3 SLEDAI 2K

SLEDAI-2K: DATA COLLECTION SHEET

SLEDAI 2K Weight	SCORE	Descriptor	Definition
8	________	Seizure	Recent onset, exclude metabolic, infectious or drug causes.
8	________	Psychosis	Altered ability to function in normal activity due to severe disturbance in the perception of reality. Include hallucinations, incoherence, marked loose associations, impoverished thought content, marked illogical thinking, bizarre, disorganized, or catatonic behavior. Exclude uremia and drug causes
8	________	Organic brain syndrome	Altered mental function with impaired orientation, memory, or other intellectual function, with rapid onset and fluctuating clinical features, inability to sustain attention to environment, plus at least 2 of the following: perceptual disturbance, incoherent speech, insomnia or daytime drowsiness, or increased or decreased psychomotor activity. Exclude metabolic, infectious, or drug causes.
8	________	Visual disturbance	Retinal changes of SLE. Include cytoid bodies, retinal hemorrhages, serous exudate or hemorrhages in the choroid, or optic neuritis. Exclude hypertension, infection, or drug causes.
8	________	Cranial nerve disorder	New onset of sensory or motor neuropathy involving cranial nerves.
8	________	Lupus headache	Severe, persistent headache; may be migrainous, but must be nonresponsive to narcotic analgesia.
8	________	CVA	New onset of cerebrovascular accident(s). Exclude arteriosclerosis.

8	________	Vasculitis	Ulceration, gangrene, tender finger nodules, periungual infarction, splinter hemorrhages, or biopsy or angiogram proof of vasculitis.
4	________	Arthritis	$\geq$ 2 joints with pain and signs of inflammation (i.e., tenderness, swelling or effusion).
4	________	Myositis	Proximal muscle aching/weakness, associated with elevated creatine phosphokinase/aldolase or electromyogram changes or a biopsy showing myositis.
4	________	Urinary casts	Heme-granular or red blood cell casts.
4	________	Hematuria	>5 red blood cells/high power field. Exclude stone, infection or other cause.
4	________	Proteinuria	>0.5 gram/24 hours
4	________	Pyuria	>5 white blood cells/high power field. Exclude infection.
2	________	Rash	Inflammatory type rash.
2	________	Alopecia	Abnormal, patchy or diffuse loss of hair.
2	________	Mucosal ulcers	Oral or nasal ulcerations.
2	________	Pleurisy	Pleuritic chest pain with pleural rub or effusion, or pleural thickening.
2	________ P	Pericarditis	Pericardial pain with at least 1 of the following: rub, effusion, or electrocardiogram or echocardiogram confirmation.
2	________	Low complement	Decrease in CH50, C3, or C4 below the lower limit of normal for testing laboratory
2	________	Increased DNA binding	Increased DNA binding by Farr assay above normal range for testing laboratory.
1	________	Fever	>38° C. Exclude infectious cause.
1	________	Thrombocytopenia	<100,000 platelets / x10^9/L, exclude drug causes.
1	________	Leukopenia	< 3,000 white blood cells / x10^9/L, exclude drug causes.

TOTAL SCORE: ______________

22.4 **BILAG 2004:**

BILAG-2004 INDEX **Centre:** **Date:** **Initials/Hosp No:**

♦ **Only record manifestations/items due to SLE Disease Activity**
♦ **Assessment refers to manifestations occurring in the last 4 weeks (compared with the previous 4 weeks)**
♦ **TO BE USED WITH THE GLOSSARY**

Record: **ND** **Not Done**
0 **Not present**
1 **Improving**
2 **Same**
3 **Worse**
4 **New**
Yes/No OR Value (where indicated)
***Y/N Confirm this is due to SLE activity (Yes/No)**

CONSTITUTIONAL
1. Pyrexia - documented > 37.5°C ( )
2. Weight loss - unintentional > 5% ( )
3. Lymphadenopathy/splenomegaly ( )
4. Anorexia ( )

MUCOCUTANEOUS
5. Skin eruption - severe ( )
6. Skin eruption - mild ( )
7. Angio-oedema - severe ( )
8. Angio-oedema - mild ( )
9. Mucosal ulceration - severe ( )
10. Mucosal ulceration - mild ( )
11. Panniculitis/Bullous lupus - severe ( )
12. Panniculitis/Bullous lupus - mild ( )
13. Major cutaneous vasculitis/thrombosis ( )
14. Digital infarcts or nodular vasculitis ( )
15. Alopecia - severe ( )
16. Alopecia - mild ( )
17. Peri-ungual erythema/chilblains ( )
18. Splinter haemorrhages ( )

NEUROPSYCHIATRIC
19. Aseptic meningitis ( )
20. Cerebral vasculitis ( )
21. Demyelinating syndrome ( )
22. Myelopathy ( )
23. Acute confusional state ( )
24. Psychosis ( )
25. Acute inflammatory demyelinating polyradiculoneuropathy ( )
26. Mononeuropathy (single/multiplex) ( )
27. Cranial neuropathy ( )
28. Plexopathy ( )
29. Polyneuropathy ( )
30. Seizure disorder ( )
31. Status epilepticus ( )
32. Cerebrovascular disease (not due to vasculitis) ( )
33. Cognitive dysfunction ( )
34. Movement disorder ( )
35. Autonomic disorder ( )
36. Cerebellar ataxia (isolated) ( )
37. Lupus headache - severe unremitting ( )
38. Headache from IC hypertension ( )

MUSCULOSKELETAL
39. Myositis - severe ( )
40. Myositis - mild ( )
41. Arthritis (severe) ( )
42. Arthritis (moderate)/Tendonitis/Tenosynovitis ( )
43. Arthritis (mild)/Arthralgia/Myalgia ( )

**Weight (kg):**	**Serum urea (mmol/l):**
**African ancestry: Yes/No**	**Serum albumin (g/l):**

CARDIORESPIRATORY
44. Myocarditis - mild ( )
45. Myocarditis/Endocarditis + Cardiac failure ( )
46. Arrhythmia ( )
47. New valvular dysfunction ( )
48. Pleurisy/Pericarditis ( )
49. Cardiac tamponade ( )
50. Pleural effusion with dyspnoea ( )
51. Pulmonary haemorrhage/vasculitis ( )
52. Interstitial alveolitis/pneumonitis ( )
53. Shrinking lung syndrome ( )
54. Aortitis ( )
55. Coronary vasculitis ( )

GASTROINTESTINAL
56. Lupus peritonitis ( )
57. Abdominal serositis or ascites ( )
58. Lupus enteritis/colitis ( )
59. Malabsorption ( )
60. Protein losing enteropathy ( )
61. Intestinal pseudo-obstruction ( )
62. Lupus hepatitis ( )
63. Acute lupus cholecystitis ( )
64. Acute lupus pancreatitis ( )

OPHTHALMIC
65. Orbital inflammation/myositis/proptosis ( )
66. Keratitis - severe ( )
67. Keratitis - mild ( )
68. Anterior uveitis ( )
69. Posterior uveitis/retinal vasculitis - severe ( )
70. Posterior uveitis/retinal vasculitis - mild ( )
71. Episcleritis ( )
72. Scleritis - severe ( )
73. Scleritis - mild ( )
74. Retinal/choroidal vaso-occlusive disease ( )
75. Isolated cotton-wool spots (cytoid bodies) ( )
76. Optic neuritis ( )
77. Anterior ischaemic optic neuropathy ( )

RENAL
78. Systolic blood pressure (mm Hg) value ( ) Y/N*
79. Diastolic blood pressure (mm Hg) value ( ) Y/N*
80. Accelerated hypertension Yes/No ( )
81. Urine dipstick protein (+=1, ++=2, +++=3) ( ) Y/N*
82. Urine albumin-creatinine ratio mg/mmol ( ) Y/N*
83. Urine protein-creatinine ratio mg/mmol ( ) Y/N*
84. 24 hour urine protein (g) value ( ) Y/N*
85. Nephrotic syndrome Yes/No ( )
86. Creatinine (plasma/serum) μmol/l ( ) Y/N*
87. GFR (calculated) ml/min/1.73 m^2 ( ) Y/N*
88. Active urinary sediment Yes/No ( )
89. Active nephritis Yes/No ( )

HAEMATOLOGICAL
90. Haemoglobin (g/dl) value ( ) Y/N*
91. Total white cell count (x 10^9/l) value ( ) Y/N*
92. Neutrophils (x 10^9/l) value ( ) Y/N*
93. Lymphocytes (x 10^9/l) value ( ) Y/N*
94. Platelets (x 10^9/l) value ( ) Y/N*
95. TTP ( )
96. Evidence of active haemolysis Yes/No ( )
97. Coombs' test positive (isolated) Yes/No ( )

Revision: 1/Sep/2009

22.5 Disease Activity Score-28 (DAS-28):

DAS28 form

Patient name……………………………………………. Date of Birth ……-……-…….

Observer name………………………………………… Date ……-……-……

	LEFT Swollen	tender	RIGHT Swollen	Tender
Shoulder				
Elbow				
Wrist				
MCP 1				
2				
3				
4				
5				
PIP 1				
2				
3				
4				
5				
Knee				
Subtotal				
Total	swollen		Tender	

No disease activity high disease activity

Swollen (0-28)	
Tender (0-28)	
ESR	
VAS disease activity (0-100mm)	

DAS28 = 0.56*√(t28) + 0.28*√(sw28) + 0.70*Ln(ESR) + 0.014*VAS

22.6 The Cutaneous Lupus Erythematosus Disease Area and Severity Index (CLASI):

Cutaneous LE Disease Area and Severity Index (CLASI)

Select the score in each anatomical location that describes the most severely affected cutaneous lupus-associated lesion

activity ⟷ damage

Extent

Anatomical Location	Erythema	Scale/ Hypertrophy	Dyspigmentation	Scarring/ Atrophy/ Panniculitis	Anatomical Location
	0-absent<br>1-pink; faint erythema<br>2- red;<br>3-dark red; purple/violaceous/ crusted/ hemorrhagic	0-absent;<br>1-scale<br>2-verrucous/ hypertrophic	0-absent,<br>1-dyspigmentaton	0 – absent<br>1 – scarring<br>2 – severely atrophic scarring or panniculitis	
Scalp				See below	Scalp
Ears					Ears
Nose (incl. malar area)					Nose (incl. malar area)
Rest of the face					Rest of the face
V-area neck (frontal)					V-area neck (frontal)
Post. Neck &/or shoulders					Post. Neck &/or shoulders
Chest					Chest
Abdomen					Abdomen
Back, buttocks					Back, buttocks
Arms					Arms
Hands					Hands
Legs					Legs
Feet					Feet

Mucous membrane

Mucous membrane lesions (examine if patient confirms involvement)	
0-absent;<br>1-lesion or ulceration	

Dyspigmentation

Report duration of dyspigmentation after active lesions have resolved (verbal report by patient – tick appropriate box)
☐ Dyspigmentation usually lasts less than 12 months (dyspigmentation score above remains)<br>☐ Dyspigmentation usually lasts at least 12 months (dyspigmentation score is doubled)

Alopecia

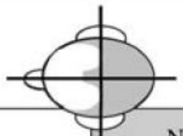

Recent Hair loss (within the last 30 days / as reported by patient)		NB: if scarring and non-scarring aspects seem to coexist in one lesion, please score both
1-Yes<br>0-No		

Divide the scalp into four quadrants as shown. The dividing line between right and left is the midline. The dividing line between frontal and occipital is the line connecting the highest points of the ear lobe. A quadrant is considered affected if there is a lesion within the quadrant.

Alopecia (clinically not obviously scarred)		Scarring of the scalp (judged clinically)	
0-absent<br>1-diffuse; non-inflammatory<br>2-focal or patchy in one quadrant;<br>3-focal or patchy in more than one quadrant		0- absent<br>3- in one quadrant<br>4- two quadrants<br>5- three quadrants<br>6- affects the whole skull	

Total Activity Score (For the activity score please add up the scores of the left side i.e. for Erythema, Scale/Hypertrophy, Mucous membrane involvement and Alopecia) ©		Total Damage Score (For the damage score, please add up the scores of the right side, i.e. for Dyspigmentation, Scarring/Atrophy/Panniculitis and Scarring of the Scalp)	

22.7 36-item Short Form Survey:

Medical Outcomes Study: 36-Item Short Form Survey Instrument

1. In general, would you say your health is:	
Excellent	1
Very good	2
Good	3
Fair	4
Poor	5

2. Compared to one year ago, how would your rate your health in general now?	
Much better now than one year ago	1
Somewhat better now than one year ago	2
About the same	3
Somewhat worse now than one year ago	4
Much worse now than one year ago	5

The following items are about activities you might do during a typical day. Does your health now limit you in these activities? If so, how much?

(Circle One Number on Each Line)

	Yes, Limited a Lot	Yes, Limited a Little	No, Not limited at All
3. Vigorous activities, such as running, lifting heavy objects, participating in strenuous sports	[1]	[2]	[3]
4. Moderate activities, such as moving a table, pushing a vacuum cleaner, bowling, or playing golf	[1]	[2]	[3]

5. Lifting or carrying groceries	[1]	[2]	[3]
6. Climbing several flights of stairs	[1]	[2]	[3]
7. Climbing one flight of stairs	[1]	[2]	[3]
8. Bending, kneeling, or stooping	[1]	[2]	[3]
9. Walking more than a mile	[1]	[2]	[3]
10. Walking several blocks	[1]	[2]	[3]
11. Walking one block	[1]	[2]	[3]
12. Bathing or dressing yourself	[1]	[2]	[3]

During the past 4 weeks, have you had any of the following problems with your work or other regular daily activities as a result of your physical health?

(Circle One Number on Each Line)

	Yes	No
13. Cut down the amount of time you spent on work or other activities	1	2
14. Accomplished less than you would like	1	2
15. Were limited in the kind of work or other activities	1	2
16. Had difficulty performing the work or other activities (for example, it took extra effort)	1	2

During the past 4 weeks, have you had any of the following problems with your work or other regular daily activities as a result of any emotional problems (such as feeling depressed or anxious)?

(Circle One Number on Each Line)

	Yes	No
17. Cut down the amount of time you spent on work or other activities	1	2
18. Accomplished less than you would like	1	2
19. Didn't do work or other activities as carefully as usual	1	2

20. During the past 4 weeks, to what extent has your physical health or emotional problems interfered with your normal social activities with family, friends, neighbors, or groups?

(Circle One Number)

Not at all 1

Slightly 2

Moderately 3

Quite a bit 4

Extremely 5

21. How much bodily pain have you had during the past 4 weeks?

(Circle One Number)

None 1

Very mild 2

Mild 3

Moderate 4

Severe 5

Very severe 6

22. During the past 4 weeks, how much did pain interfere with your normal work (including both work outside the home and housework)?

(Circle One Number)

Not at all 1

A little bit 2

Moderately 3

Quite a bit 4

Extremely 5

These questions are about how you feel and how things have been with you during the past 4 weeks. For each question, please give the one answer that comes closest to the way you have been feeling.

How much of the time during the past 4 weeks . . .

(Circle One Number on Each Line)

	All of the Time	Most of the Time	A Good Bit of the Time	Some of the Time	A Little of the Time	None of the Time
23. Did you feel full of pep?	1	2	3	4	5	6
24. Have you been a very nervous person?	1	2	3	4	5	6
25. Have you felt so down in the dumps that nothing could cheer you up?	1	2	3	4	5	6
26. Have you felt calm and peaceful?	1	2	3	4	5	6
27. Did you have a lot of energy?	1	2	3	4	5	6
28. Have you felt downhearted and blue?	1	2	3	4	5	6
29. Did you feel worn out?	1	2	3	4	5	6
30. Have you been a happy person?	1	2	3	4	5	6
31. Did you feel tired?	1	2	3	4	5	6

32. During the past 4 weeks, how much of the time has your physical health or emotional problems interfered with your social activities (like visiting with friends, relatives, etc.)?

(Circle One Number)

All of the time 1

Most of the time 2

Some of the time 3

A little of the time 4

None of the time 5

How TRUE or FALSE is each of the following statements for you.

(Circle One Number on Each Line)

	Definitely True	Mostly True	Don't Know	Mostly False	Definitely False
33. I seem to get sick a little easier than other people	1	2	3	4	5
34. I am as healthy as anybody I know	1	2	3	4	5
35. I expect my health to get worse	1	2	3	4	5
36. My health is excellent	1	2	3	4	5

22.8 Multidimensional Assessment of Fatigue:

MULTIDIMENSIONAL ASSESSMENT OF FATIGUE (MAF) SCALE

Instructions: These questions are about fatigue and the effect of fatigue on your activities.

For each of the following questions, circle the number that most closely indicates how you have been feeling during the past week.

For example, suppose you really like to sleep late in the mornings. You would probably circle the number closer to the "a great deal" end of the line. This is where I put it:

Example: To what degree do you usually like to sleep late in the mornings?

1 2 3 4 [illegible] 6 7 8 9 1

Not at all — A great deal

Now please complete the following items based on the past week.

---

1. To what degree have you experienced fatigue?

1 2 3 4 5 6 7 8 9 1

Not at all — A great deal

If no fatigue, stop here.

2. How severe is the fatigue which you have been experiencing?

1 2 3 4 5 6 7 8 9 1

Mild — Severe

3. To what degree has fatigue caused you distress?

1 2 3 4 5 6 7 8 9 1

No distress — A great deal of distress

MULTIDIMENSIONAL ASSESSMENT OF FATIGUE (MAF) SCALE (Continued)

Circle the number that most closely indicates to what degree fatigue has interfered with your ability to do the following activities in the past week. For activities you don't do, for reasons other than fatigue (e.g. you don't work because you are retired), check the box.In the past week, to what degree has fatigue interfered with your ability to:

(NOTE: Check box to the left of each number if you don't do activity)

☐ 4. Do household chores

☐1 ☐2 ☐3 ☐4 ☐5 ☐6 ☐7 ☐8 ☐9 ☐1

Not at all A great deal

☐ 5. Cook

☐1 ☐2 ☐3 ☐4 ☐5 ☐6 ☐7 ☐8 ☐9 ☐1

Not at all A great deal

☐ 6. Bathe or wash

☐1 ☐2 ☐3 ☐4 ☐5 ☐6 ☐7 ☐8 ☐9 ☐1

Not at all A great deal

☐ 7. Dress

☐1 ☐2 ☐3 ☐4 ☐5 ☐6 ☐7 ☐8 ☐9 ☐1

Not at all A great deal

☐ 8. Work

☐1 ☐2 ☐3 ☐4 ☐5 ☐6 ☐7 ☐8 ☐9 ☐1

Not at all A great deal

☐ 9. Visit or socialize with friends or family

☐1 ☐2 ☐3 ☐4 ☐5 ☐6 ☐7 ☐8 ☐9 ☐1

Not at all A great deal

MULTIDIMENSIONAL ASSESSMENT OF FATIGUE (MAF) SCALE (Continued)

(NOTE: Check box to the left of each number if you don't do activity)

☐ 10. Engage in sexual activity

1 2 3 4 5 6 7 8 9 1

Not at all A great deal

☐ 11. Engage in leisure and recreational activities

1 2 3 4 5 6 7 8 9 1

Not at all A great deal

☐ 12. Shop and do errands

1 2 3 4 5 6 7 8 9 1

Not at all A great deal

☐ 13. Walk

1 2 3 4 5 6 7 8 9 1

Not at all A great deal

☐ 14. Exercise, other than walking

1 2 3 4 5 6 7 8 9 1

Not at all A great deal

15. Over the past week, how often have you been fatigued?

- 4 Every day
- 3 Most, but not all days
- 2 Occasionally, but not most days
- 1 Hardly any days

16. To what degree has your fatigue changed during the past week?

- 4 Increased
- 3 Fatigue has gone up and down
- 2 Stayed the same
- 1 Decreased

22.9 NIH Clinical Center guidelines for the management of allergic reactions:

ANAPHYLAXIS TREATMENT MEDICATION DOSE GUIDELINES – PRIMARY THERAPY			
**DRUG**	**CONCENTRATION**	**ADULT DOSE** (1)	**PEDIATRIC DOSE** (1,2,3)
**First-line Treatment**			
**EPINEPHRINE AUTO-INJECTOR** (EpiPen)	1:1,000 (0.3 MG Fixed Dose Inj)	0.3 mg > 25 Kg (3) **IM** (4) MAY REPEAT q 5 to 15 mins	0.3 mg > 25 Kg (3) **IM** (4) MAY REPEAT q 5 to 15 mins
**EPINEPHRINE AUTO-INJECTOR Jr** (EpiPen Jr.)	1:2,000 (0.15 MG Fixed Dose Inj)	N/A	0.15 mg 10 to 25 Kg(3) **IM** (3) or SQ MAY REPEAT q 5 to 15 mins
**EPINEPHRINE AMPULE**	1:1,000 (1 mg/mL)	0.2 to 0.5 mg per dose **IM** (4) or Subcutaneous MAY REPEAT q 5 to 15 mins	0.01 mg/Kg per dose **IM** (4) or Subcutaneous MAY REPEAT q 5 to 15 mins<br>MAX SINGLE DOSE 0.5 mg (0.5 mL)

1. The diagnosis and management of anaphylaxis practice parameter: 2010 Update. J Allergy Clin Immunol 2010;126: 477-80.
2. The Harriet Lane Handbook, 18th Edition
3. This differs from the package insert recommendation as per Guidelines for the Diagnosis and Management of Food Allergy in the United States: Report of the NIAID-Sponsored Expert Panel. J Allergy Clin Immunol 2010;126: S1 – S58.
4. The intramuscular (IM) route is preferred. Epinephrine absorption in adults: Intramuscular versus subcutaneous injection. J Allergy Clin Immunol 2001;108:871-3.

SEE REVERSE SIDE FOR ADJUNCTIVE THERAPY➔

Approved by P&T Committee on February 24, 2011. Revised on XX/XX/2011

ANAPHYLAXIS TREATMENT MEDICATION DOSE GUIDELINES – PRIMARY THERAPY			
**DRUG**	**CONCENTRATION**	**ADULT DOSE** (1)	**PEDIATRIC DOSE** (1,2,3)
**First-line Treatment**			
**EPINEPHRINE AUTO-INJECTOR** (EpiPen)	1:1,000 (0.3 MG Fixed Dose Inj)	0.3 mg<br>> 25 Kg (3)<br>**IM** (4)<br>MAY REPEAT q 5 to 15 mins	0.3 mg<br>> 25 Kg (3)<br>**IM** (4)<br>MAY REPEAT q 5 to 15 mins
**EPINEPHRINE AUTO-INJECTOR Jr** (EpiPen Jr.)	1:2,000 (0.15 MG Fixed Dose Inj)	N/A	0.15 mg<br>10 to 25 Kg(3)<br>**IM** (3) or SQ<br>MAY REPEAT q 5 to 15 mins
**EPINEPHRINE AMPULE**	1:1,000 (1 mg/mL)	0.2 to 0.5 mg per dose<br>**IM** (4) or Subcutaneous<br>MAY REPEAT q 5 to 15 mins	0.01 mg/Kg per dose<br>**IM** (4) or Subcutaneous<br>MAY REPEAT q 5 to 15 mins<br><br>MAX SINGLE DOSE 0.5 mg (0.5 mL)

1. The diagnosis and management of anaphylaxis practice parameter: 2010 Update. J Allergy Clin Immunol 2010;126: 477-80.
2. The Harriet Lane Handbook, 18th Edition
3. This differs from the package insert recommendation as per Guidelines for the Diagnosis and Management of Food Allergy in the United States: Report of the NIAID-Sponsored Expert Panel. J Allergy Clin Immunol 2010;126: S1 – S58.
4. The intramuscular (IM) route is preferred. Epinephrine absorption in adults: Intramuscular versus subcutaneous injection. J Allergy Clin Immunol 2001;108:871-3.

SEE REVERSE SIDE FOR ADJUNCTIVE THERAPY➔

Approved by P&T Committee on February 24, 2011. Revised on XX/XX/2011

22.10 XELJANZ (tofacitinib) patient prescribing information:

Xeljanz prescribing information

22.11 NCI Common Toxicity Criteria, Version 4.0:

http://evs.nci.nih.gov/ftp1/CTCAE/CTCAE_4.03_2010-06-14_QuickReference_5x7.pdf

22.12 Physician Global Assessment (PGA):

Mark an X on the line below to indicate disease activity (independent of patient's self assessment):

Very good |———————————————————| Very bad

22.13 Consent Forms: Attached as a separate file.

22.14 Notice of treatment allocation letter to be attached:

National Institute of Arthritis and Musculoskeletal and Skin Diseases

Sarfaraz Hasni, M.D.
Building 10, Room 3-2340
10 Center Drive
Bethesda, MD 20892

(Date)

Dear (Subject Name)

This letter is to inform you that the clinical trial you participated in, *Safety of Tofacitinib, an Oral Janus Kinase Inhibitor, in Systemic Lupus Erythematosus; a Phase Ib Clinical Trial and Associated Mechanistic Studies,* is complete. Thank you for your participation in our clinical treatment study at the National Institutes of Health.

It was revealed that you received (the study medication, Tofacitnib or Placebo, non-active substance) during the study.

To date, there are no major safety concerns identified during the study. We will continue to analyze data we have collected and evaluate the information gathered accordingly.

Again, thank you for your participation is research studies with NIH. Your contribution is invaluable in our ongoing efforts to better understand Lupus and develop better treatments for patients.

If you have any questions or concerns please feel free to contact me or the research nurse, Elaine Poncio, at 301-435-4489.

Sincerely,

Sarfaraz A. Hasni, M.D.
Director, Lupus Clinical Research Program
Phone: (301) 451-1599

Amendment Log for Protocol 15-AR-0185

Updated 12/30/2019

Amendment (# or Letter)	Date Submitted to the IRB	Date IRB Approved	Consent changes (Yes/No)	Requires Reconsent (Yes/No)	Consent Version (if applicable)
**Initial Review**	5/20/2015	8/17/2015	N/A	N/A	N/A
**Expedited Amendment A**	3/16/2016	3/29/2016	Yes	No	A
1. Removed associate investigators, Alice Fike and Elizabeth Joyal, because they are no longer at NIAMS.					
2. Added associate investigator, Michael Davis, as the nurse practitioner on the study and will perform majority of protocol related visits requiring a medical evaluation. Elaine Poncio added as the back–up study coordinator. Both investigators responsible for informed consent process.					
3. Added FIMR in the list of abbreviations.					
4. We reviewed the inclusion and exclusion criteria to improve recruitment and other logistical issues. See updated below:<br>a. Inclusion criteria # 4: Has mild to moderate disease activity defined as SLEDAI-2K score ≥ 2 and ≤14 at the screening visit.<br>b. Males and females with potential for reproduction must agree to practice effective birth control measures. Females should be on adequate contraception if they are of child-bearing potential documented by a clinician, unless patients or spouse have previously undergone a sterilization procedure. Adequate will be considered intrauterine device (IUD) alone or hormone implants, injectables, or oral contraceptives plus a barrier method (male condom, female condom or diaphragm), abstinence or a vasectomized partner.<br>c. Added Inclusion criteria # 8: If patients are on ACE inhibitors or ARB medications, dose of this medication must be stable for 4 weeks prior to study entry.<br>d. Added inclusion criteria # 9: Patients may be on lipid lowering medications if initiated at least 6 months prior to the screening visit.<br>e. Deleted exclusion criteria # 2 and clarified with inclusion #7.<br>f. Modified exclusion criteria # 3: Current or prior treatment with rituximab, belimumab or any other biologic agent in the 6 months prior to screening.<br>g. Added methotrexate to the list of medications in exclusion criteria # 5<br>h. Corrected the unit of measurement of protein to creatinine ratio in exclusion criteria # 11: Protein to creatinine ratio of more than 1. mg/mg or 24 hours urine protein of more than 1000 mg.<br>i. Modified exclusion criteria # 15: Hypercholesterolemia: Values after an 8-12 hour fasting blood specimen: total cholesterol >250mg/dL or LDL >180 mg/dl or hypertriglyceridemia (triglyceride >300 mg/dL) within +/- 30 days of screening visit.					
5. Section 5.2.1: Primary endpoint modified as follows to clarify the AE relatedness to the drug. Lipid studies were all clarified that ideally, lipid studies will be performed on a 8-12 hour fasting state, however if fasting samples are not available we may still analyze the samples noting this limitation. In addition to the routine lipid profile and lipoprotein panel, we may also include measurements of lipoproteins such as; proinflammatory HDL and oxidized HDL that has been associated with increased atherosclerotic risk in SLE.					
6. Clarification that screening will occur within 30 days of first treatment.					
7. Treatment allocation clarified to a n=20 subjects, 10 of whom are homozygous or heterozygous for STAT4 risk allele or placebo twice daily (n= 10 subjects) for 56 days.					
8. Clarification that BK viruria titers defined as an increase in BK viruria greater than 10 million copies/ml.					

9. Randomization and blinding process has to be modified due to the changes in personal and procedures at the NIH Research Pharmacy. We have revised the process and made changes as follows:<br>a. Section 5.13.2: After confirmation of eligibility, the PI or designated AI will notify the statistician associated with the study, who will work in conjunction with the Clinical Center Research Pharmacy to randomize the patient based on presence or absence of STAT 4 risk allele.<br>b. Section 5.17.1: The results indicating the presence or absence of STAT 4 risk alleles will be communicated to the NIH Clinical Center Research Pharmacy. As subjects who are followed in these observational cohorts' experience disease flares and become eligible for participation in this study, investigators will contact the statistician associated with the study who will work in conjunction with the NIH Clinical Center Research Pharmacy during the screening process to determine eligibility based on STAT4 risk alleles. Clinical Center Research Pharmacy will work in conjunction with the statistician associated with the study to ensure that a minimum of 10 eligible subjects are either heterozygous or homozygous for the STAT4 risk allele and that a minimum of 10 eligible subjects do not have the risk allele. The placebo group may or may not have STAT4 risk alleles. This will help to maintain the blind for the investigators and study subjects.<br>c. Section 5.17.2: Randomization will be done by the NIH Clinical Research Center Pharmacy in conjunction with the statistician associated with the study. This is a two-arm study and subjects will be randomized to either tofacitinib or placebo in a 2:1 ratio so that 10 subjects with STAT 4 risk alleles and 10 subjects without STAT 4 risk alleles are in the tofacitinib treatment group. The genotype for the placebo group (n=10) is not pertinent to the randomization as these subjects may or may not have the risk alleles. Both subjects and investigators will be blinded to treatment allocation during the first phase of the study. Unblinding will occur for the analyses after the last subject has completed Day 84 of the study. We will inform the subjects of their treatment allocation after the last subject has completed Day 84 of the study.<br>The blind will be held by the NIH Clinical Center Research Pharmacy as well as the statistician associated with the study. In cases where breaking the blind is necessary to provide clinical care to the patient (as determined by the physician involved in patient's care and principal investigator), the principal investigator will contact the NIH Clinical Center Research Pharmacy and provide the treatment allocation information to the treating physician. Such subject(s) would be withdrawn from the study and followed up as described in section 4.10 above. The Data and Safety Monitoring Committee (DSMC) may also request unblinding of treatment allocation or group assignment. These requests will be transmitted to the NIH Clinical Center Research Pharmacy by the principal investigator. The data will be provided directly to the Chair of DSMC without unblinding the investigators before the DSMC determines a plan of action.
10. We have made some changes in the study visit procedures in view of logistical concerns:<br>a. Protocol page 35-36, section 5.13.3 Day 1 +/- 7 days Study Visit we would not review the inclusion /exclusion criteria as the SLEDAI 2K cannot be calculated due to non-availability of the lab results also the immunoglobulins will not be measured on this visit.<br>b. Protocol page 36-37, section 5.13.5 Day 14 +/- 7days Study Visit we would not do a pill count and not measure the quantitative immunoglobulins.<br>c. Protocol page 46, section 5.16 Treatment Compliance the procedure was clarified as follows: Compliance with study drug dosing will be assessed at the Day 28 and 56 (+/- 7 days) visits. Subjects will be asked to bring their bottles for a pill count that will be recorded. Subjects who have demonstrated less than 80% compliance at the Day 28 and 56 (+/- 7 days) visits will be withdrawn from the study.<br>d. Protocol page 49, section 5.20 withdrawal criteria and the following changes were made to maintain internal consistency:<br>1. More than 30 days delay in treatment after the screening visit.<br>2. Urine BK virus level of more than 10 million copies/ml by quantitative PCR or detection of BK virus in serum by quantitative PCR.

11. Medication and Placebo will no longer be supplied by NIH Research Pharmacy due to issues with PDS. We have approached Pfizer incorporated and they have agreed to supply the medication and placebo under their investigator-initiated research program. We are in process of finalizing a clinical trials agreement with them. The clinical trial is still being sponsored by NIAMS. We made the following changes based on this change:<br>a. Protocol page 46, section 5.15.2 Tofacitinib dosing the following sentence was added: Tofacitinib tablets will be supplied by Pfizer Incorporated to the NIH Clinical Center Research Pharmacy.<br>b. Protocol page 46, section 5.15.3 Placebo the sentence was modified as follows: Placebo tablets will be supplied by Pfizer Incorporated to the NIH Clinical Center Research Pharmacy.
12. Editorial changes on Protocol page 56, section 6.4.3.3 Peripheral arterial tonometry (Endopat).
13. The Clinical Monitoring of the trial is done through Leidos Biomedical Research Inc. Clinical Monitoring Research Program (CMRP), Clinical Trials Management (CTM) team. After site initiation visit the Leidos team requested some modification in the monitoring language to more accurately reflect their monitoring procedures. We modified the following in Protocol pages 64 and 65, section 10.3.5 Data Collection, Quality Control and Quality Assurance Monitoring:<br>a. Clinical monitoring for this study will be based on a clinical monitoring plan developed by Leidos Biomedical Research Inc. Clinical Monitoring Research Program (CMRP), Clinical Trials Management (CTM) team in collaboration with the Principal Investigator. The purposes of the clinical monitoring activities are to: 1) to verify the existence of signed informed consent documents and documentation of the ICF process for each monitored subject; 2) to verify the prompt and accurate recording of all monitored data points, and prompt reporting of all SAEs; 3) to compare abstracted information in CTDB with individual subjects' records and source documents (subjects' charts, laboratory analyses and test results, physicians' progress notes, nurses' notes, and any other relevant original subject information);4) to help ensure investigators are in compliance with the protocol, and 5) protocol drug accountability . The monitors will also inspect the clinical site regulatory files to ensure that regulatory requirements (Office for Human Research Protections-OHRP) and applicable guidelines (ICH GCP) are being followed. The clinical monitoring plan will specify the frequency, procedures, and levels of monitoring activities. Some monitoring activities will be performed remotely (e.g., review of regulatory documents), while others will take place on site (e.g., verification of study databases against source documentation). Staff from Leidos Biomedical Research, Inc. CMRP CTM will conduct the monitoring activities and provide follow-up letters describing the findings. The frequency of reporting for monitoring activities will be specified in the monitoring plan. The Principal Investigator will receive copies of the final follow-up letters.<br>b. Protocol pages 65 and 66, section 10.3.6 Review Schedule is modified as follows at:<br>• Initial site monitoring visit: Prior to study start-up for GCP and protocol overview<br>• Follow-up monitoring visits: Will occur approximately 2-3 times a year based on subject enrollment and follow up visits. The first 3 enrolled subjects will be monitored at 100%. Approximately 10-30% of the remaining enrolled subjects will be randomly selected for monitoring of eligibility criteria, AE/SAE/UP reporting and key data points per PI. All enrolled subject's Informed Consent Documents will be monitored.<br>• Final monitoring visit: Study close out once all subjects are off study or other study closure event occurs.
14. To keep adverse events reporting language in one place we moved section 16.2.2 Clinically Significant Laboratory Abnormalities from protocol page 82 to section 16.1.5 Clinical Laboratory Test Results Not Qualifying as Adverse Events or Serious Adverse Events on protocol page 76.
15. Miscellaneous formatting and minor editorial changes made through-out the protocol were highlighted.
16. We request IRB to approve study flyer.
17. The following changes were made to the informed consent document:<br>a. Informed Consent page 3, section study design, paragraph 6 the following was deleted: Please bring your bottles of study drug with you to the visit.

b. Informed consent page 11, section Conflict of Interest the following was added: The medication and placebo for this study are being supplied by Pfizer incorporated. However, they have no role in the study design and conduct. We will share a summary of results with Pfizer upon completion of the study.<br>c. Informed consent page 6, study procedure table was updated to reflect that inclusion/exclusion criteria will not be reviewed on Day 1.<br>d. Informed consent page 12, changes were made to reflect updated contact information: Deleted: Elizabeth Joyal, MSN and Added: Elaine Poncio, BSN					
**Amendment (# or Letter)**	**Date Submitted to the IRB**	**Date IRB Approved**	**Consent changes (Yes/No)**	**Requires Reconsent (Yes/No)**	**Consent Version (if applicable)**
**Continuing Review**	4/4/2016	6/15/2016	N/A	N/A	N/A
**Expedited Amendment B**	5/3/2016	7/7/2016	Yes	No	B
1. We request IRB to approve the Spanish translation of the latest version of the consent form. Translated consent and certificate from NIH Translations office was submitted with this memo.					
**Amendment (# or Letter)**	**Date Submitted to the IRB**	**Date IRB Approved**	**Consent changes (Yes/No)**	**Requires Reconsent (Yes/No)**	**Consent Version (if applicable)**
**Expedited Amendment C**	8/31/2016	9/22/2016	Yes	No	C
1. The changes are for staff changes, contraception update, and informed consent clarification as follows to protocol:<br>a. Inclusion criteria #7 add: hormone patches.<br>b. Removed Simantini Sakhardande. Added Elaine Poncio and Michael Davis to perform vascular function studies. Added Sarthak Gupta as an associate investigator.<br>c. Consent changes are to add dosing of tofa: 5mg twice daily for 56 days and to change the language from 2 forms to an effective method of birth control.					
**Amendment (# or Letter)**	**Date Submitted to the IRB**	**Date IRB Approved**	**Consent changes (Yes/No)**	**Requires Reconsent (Yes/No)**	**Consent Version (if applicable)**
**Expedited Amendment D**	10/17/2016	10/26/2016	Yes	No	D
1. We request IRB to approve the Spanish translation of the latest version of the consent form. Translated consent and certificate from NIH Translations office is submitted with this memo.					
**Amendment (# or Letter)**	**Date Submitted to the IRB**	**Date IRB Approved**	**Consent changes (Yes/No)**	**Requires Reconsent (Yes/No)**	**Consent Version (if applicable)**
**Expedited Amendment E**	3/1/2017	3/13/2017	Yes	Yes	E
1. Added Associate Investigators: Donald Thomas Jr. added as a referring rheumatologist. Mohammad Naqi added as staff performing vascular function studies.					
2. Protocol face page study start date updated and we clarified that we have a CTA with Pfizer on page 82, section 19.					
3. Informed consent section updated, and redundancy of information removed.					
4. Inclusion and exclusion section 2.1 updated to improve recruitment and other logistical issues.<br>a. Inclusion #6- Antimalarials such as chloroquine phosphate (up to 250mg daily) and quinacrine (up to 100mg daily) added					

<table>
<tr><td colspan="6">5. Screening window increased to 45 days to improve scheduling that is conducive to patient's availability. Screening sections updated to reflect this change in table of contents, section 4.2, section 5.2, section 5.1.1, section 5.6, section 5.13.2, section 5.20, and section 22.1</td></tr>
<tr><td colspan="6">6. Time window for routine labs for pre-screening increased to 21 days to improve the possibility of including patients seen under 94-AR-0066. Section 5.1.1 updated.</td></tr>
<tr><td colspan="6">7. To minimize patient discomfort and unnecessary blood draw, we added the following to section 5.13.3:
a. If a patient is found to have an acute infection at the baseline visit, their next visit will be scheduled for no more than 45 days later, at which time if the infections has cleared up, they will start the study medication. We will repeat only baseline clinical labs at this extra visit; we will not repeat vascular function studies or research sample collection.
b. If a patient has an abnormal lab result at the baseline visit that requires a repeat, the patients next visit will be scheduled no more than 45 days later, at which time if the abnormal lab has improved or normalized, they will start the study medication. We will not repeat vascular function studies or research sample collection.</td></tr>
<tr><td colspan="6">8. Based on recommendations from our outside monitors, we clarified language in the protocol to determine that missed study medication doses will be not be considered as a deviation. Section 5.16 was updated to say that we are not reporting each missed dose if the patient is within the compliancy range. Missed doses are an expected occurrence under the protocol.</td></tr>
<tr><td colspan="6">9. Restricted medication section 5.18.1 was updated to reflect the changes to inclusion criteria #6</td></tr>
<tr><td colspan="6">10. Labs (albumin and ESR were added in order to assess SLE disease activity for BILAG 2004. The following sections were updated to reflect this: 5.13.2, 5.13.6, 5.13.8, 5.13.9, 5.13,10, and 22.1</td></tr>
<tr><td colspan="6">11. We anticipate participation of eligible NIH employees, so language was added to clarify this, and protections were described in section 14 and section 10.6.</td></tr>
<tr><td colspan="6">12. Informed consent was updated to reflect the changes in this amendment. Chloroquine and quinacrine allowance and dose level were updated in informed consent page 2, page 3, and page 4. Screening window was also updated in informed consent page 5 and page 6.</td></tr>
<tr><td>Amendment (# or Letter)</td><td>Date Submitted to the IRB</td><td>Date IRB Approved</td><td>Consent changes (Yes/No)</td><td>Requires Reconsent (Yes/No)</td><td>Consent Version (if applicable)</td></tr>
<tr><td>Continuing Review</td><td>3/1/2017</td><td>4/24/2017</td><td>N/A</td><td>N/A</td><td>N/A</td></tr>
<tr><td>Expedited Amendment F</td><td>5/6/2017</td><td>5/10/2017</td><td>Yes</td><td>No</td><td>F</td></tr>
<tr><td colspan="6">1. We request IRB to approve the Spanish translation of the latest version of the consent form. Translated consent and certificate from NIH Translations office is submitted with this memo.</td></tr>
<tr><td>Amendment (# or Letter)</td><td>Date Submitted to the IRB</td><td>Date IRB Approved</td><td>Consent changes (Yes/No)</td><td>Requires Reconsent (Yes/No)</td><td>Consent Version (if applicable)</td></tr>
<tr><td>Expedited Amendment G</td><td>6/2/2017</td><td>7/21/2017</td><td>Yes</td><td>Yes</td><td>G</td></tr>
<tr><td colspan="6">This amendment includes modifications to the inclusion, exclusion criteria and language has been updated to clarify how dosing compliance will be determined.</td></tr>
<tr><td colspan="6">1. To improve recruitment, we reviewed the allowed concomitant medication chloroquine phosphate and we increased the dose allowance to 500mg daily from 250mg. There are no contraindications cited for this increase.
a. Inclusion #6, the maximum allowed dose for chloroquine phosphate is up to 500 mg daily from 250mg daily.
b. Restricted medications section updated to reflect the change of the chloroquine phosphate to 500 mg daily from 250mg daily.</td></tr>
</table>

2. Due to a SUSAR report from the Pfizer drug safety team about a clinical trial using tofacitinib we updated the exclusion criteria. In one such report a patient was diagnosed with malignant melanoma after a biopsy of the patient's finger and death occurred approximately a month later. The patient had prior history of basal cell carcinoma and it was concluded that there is reasonable possibility that this event is related to tofacitinib. This patient was taking tofacitinib 10mg BID for more than 3 years as oppose to our clinical trial that is investigating tofacitinib at 5mg BID for 56 days. Although it is unlikely that a brief exposure of 56 days of a lower dose tofacitinib will increase the risk of malignancy to current or future patients in our trial, we have modified our exclusion criteria to exclude patients with any history of cancer.<br>a. Exclusion #15: History of cancers
3. We clarified how dosing compliance will be assessed based on a discovery from a pill count. It appeared that a patient was non-compliant although the patient is reliable and overall compliant with study requirements and instructions. Upon further investigation, it was discovered that there were discrepancies with the quantity of pills in medication bottles in storage and there was extra overfill in some bottles. Extra overfill in the medication bottles could falsely withdraw patients from the study. So, pill count on day 28 and day 56 alone will not be the only determining factor to assess for dosing compliancy. We will also consider the patient's report of missed doses and patient reliability in addition to the pill count on day 28 and day 56.<br>a. Language was updated in the treatment compliance section to say the following: The compliance with study medication will be determined by the principal investigator based on multiple factors including but not limited to pill count of the returning bottles, subject's overall compliance with study procedures, subject's self-reporting of compliance and subject's reliability based on prior interactions. However, if the subject is determined to be noncompliant in the opinion of the principal investigator, then they the subject will be withdrawn from the study.
4. The informed consent was also updated to reflect the amendment changes.<br>a. Page 4 of informed consent updated to reflect the exclusion any history of cancer.<br>b. Page 4 of informed consent updated to reflect that chloroquine 500mg daily dose level is accepted.

**Amendment (# or Letter)**	**Date Submitted to the IRB**	**Date IRB Approved**	**Consent changes (Yes/No)**	**Requires Reconsent (Yes/No)**	**Consent Version (if applicable)**
**Expedited Amendment H**	9/26/2017	10/23/2017	Yes	No	H
1. We request IRB to approve the Spanish translation of the latest version of the consent form. Translated consent and certificate from NIH Translations office is submitted with this memo.					

**Amendment (# or Letter)**	**Date Submitted to the IRB**	**Date IRB Approved**	**Consent changes (Yes/No)**	**Requires Reconsent (Yes/No)**	**Consent Version (if applicable)**
**Continuing Review**	3/6/2018	4/23/2018	N/A	N/A	N/A
**Expedited Amendment I**	3/6/2018	3/16/2018	No	No	I
1. Removed associate investigators, Yasuko Furumoto, Shubhasree Choudhury, and Ann Biehl because they are no longer at NIAMS.					
2. Added associate investigator, Isabel Ochoa-Navas, to the protocol.					
3. Protocol was updated to add how patients will be notified of their treatment allocation during the study.<br>a. Page 45, Randomization and Blinding Section:<br>We will inform the subjects of their treatment allocation after the last subject has completed Day 84 of the study. We will send subjects a notice of treatment allocation letter through certified mail. See Appendix 22.14.<br>b. We added the Notice of Treatment Allocation Letter template in the appendices section of the protocol.					

Amendment Log for Protocol 15-AR-0185

Updated 12/30/2019

Amendment (# or Letter)	Date Submitted to the IRB	Date IRB Approved	Consent changes (Yes/No)	Requires Reconsent (Yes/No)	Consent Version (if applicable)
**Continuing Review**	3/21/2019	4/23/2019	N/A	N/A	N/A

STATISTICAL ANALYSIS PLAN

1. Analysis Populations:

A. Safety Population:

The Safety Population will consist of all enrolled subjects receiving at least one dose of study treatment.

B. Efficacy Population:

The Efficacy Population (EP) will consist of subjects who receive at least 3 doses of study treatment.

2. Data Analysis:

Demographic and Baseline Characteristics

Demographic and baseline characteristics will be summarized in tables. Continuous demographic and baseline variables will be summarized as means, medians, standard deviations, minimum values, and maximum values. Categorical demographic (e.g., race) and baseline variables will be summarized as frequencies and percentages.

3. Primary outcome:

The primary outcome is to evaluate the safety and tolerance of tofacitinib in patients with SLE. This analysis will include a comparison of rates of adverse events (serious adverse events, Grade 3 and 4 toxicities not fulfilling the criteria for SAE, and non-serious adverse events) and rates of SLE disease flares between the tofacitinib group and the placebo group.

4. Secondary outcomes:

Secondary outcomes will be analyzed by comparisons between the treatment (all SLE subjects receiving tofacitinib) and placebo groups and by comparisons between the STAT4 + and STAT4 – groups once the last subject completes the 84 days of study medication.

- Reduced expression of STAT-related target genes; expression of pro-inflammatory and immunoregulatory cytokines in T cells and monocytes using microarray analysis.
- A change in clinical efficacy will be analyzed using the chi-square test comparing the proportions of subjects in the treatment/placebo and STAT4+/STAT4- groups that achieve clinical response at Week 12.

- Differences between the treatment/placebo groups and the STAT4+/STAT4- groups in changes in individual disease activity measures (SLEDAI 2K, PGA) at the end of week 12 will be analyzed as repeated measures with change from baseline as the dependent variable.

5. Exploratory Analyses:

Data generated from the exploratory mechanistic studies may not have sufficient power for significance, given the limited number of subjects in this study. Nonetheless, the data generated from differential expression of STAT4 regulated genes in the subjects with STAT4 risk allele present and STAT4 risk allele absent as well as interferon signature in the two groups will be significant. This approach is likely to be hypothesis generating and serves to seed future studies toward a mechanistic understanding of SLE and the effect of JAK inhibition treatment on the dysregulation of the immune system seen in people with SLE.

6. Criteria for Significance:

The number of subjects in this study is unlikely to lead to a statistically significant difference in adverse events between subjects on study medication vs. placebo. Therefore, no formal statistical analysis will be performed for the primary outcome.

Hypothesis tests and confidence intervals of secondary analyses will be either 1- or 2-sided. Results will be considered significant at the $\alpha = 0.05$ level.

7. Power Analysis:

This is a pilot, Phase Ib study intended to study predominantly the tolerability/toxicity of tofacitinib. The number of subjects for this study is arbitrary and is based primarily on the conventional numbers in Phase I studies and our experience with similar studies in the past. The primary endpoint is safety and tolerability and 15-20 subjects are commonly used as the sample size for open label safety studies. Our plan to have 20 subjects dosed with tofacitinib to evaluate for safety and a placebo group of additional 10 subjects for comparison is consistent with Phase 1 studies.

8. Interim Analysis:

No interim analysis will be performed.

9. Accrual Number Request:

We plan to treat 30 subjects with at least 112 doses of study medication each (56 days). Subjects withdrawn before receiving 38 doses of study drug (19 days) for reasons other than drug related adverse events or lack of efficacy will be replaced. Assuming a 25% attrition rate we would like to accrue up to 38 subjects.
