# Supplementary material for: Phase 1 double-blind randomized safety trial of the Janus kinase inhibitor tofacitinib in systemic lupus erythematosus
Source: Nat Commun. 2021 Jun 7;12:3391. doi: 10.1038/s41467-021-23361-z (PMC8185103; doi:10.1038/s41467-021-23361-z)
Supplement: Supplementary file 3 — Reporting Summary [file 41467_2021_23361_MOESM3_ESM.pdf]

nature research

Corresponding author(s): Sarfaraz Hasni

Last updated by author(s): Apr 7, 2021

Reporting Summary

Nature Research wishes to improve the reproducibility of the work that we publish. This form provides structure for consistency and transparency in reporting. For further information on Nature Research policies, see Authors & Referees and the Editorial Policy Checklist .

Statistics

For all statistical analyses, confirm that the following items are present in the figure legend, table legend, main text, or Methods section.

n/a	Confirmed	
☐	☒	The exact sample size (*n*) for each experimental group/condition, given as a discrete number and unit of measurement
☐	☒	A statement on whether measurements were taken from distinct samples or whether the same sample was measured repeatedly
☐	☒	The statistical test(s) used AND whether they are one- or two-sided<br>*Only common tests should be described solely by name; describe more complex techniques in the Methods section.*
☐	☒	A description of all covariates tested
☐	☒	A description of any assumptions or corrections, such as tests of normality and adjustment for multiple comparisons
☐	☒	A full description of the statistical parameters including central tendency (e.g. means) or other basic estimates (e.g. regression coefficient) AND variation (e.g. standard deviation) or associated estimates of uncertainty (e.g. confidence intervals)
☐	☒	For null hypothesis testing, the test statistic (e.g. *F*, *t*, *r*) with confidence intervals, effect sizes, degrees of freedom and *P* value noted<br>*Give P values as exact values whenever suitable.*
☒	☐	For Bayesian analysis, information on the choice of priors and Markov chain Monte Carlo settings
☐	☒	For hierarchical and complex designs, identification of the appropriate level for tests and full reporting of outcomes
☒	☐	Estimates of effect sizes (e.g. Cohen's *d*, Pearson's *r*), indicating how they were calculated

Our web collection on statistics for biologists contains articles on many of the points above.

Software and code

Policy information about availability of computer code

Data collection	No software was used
Data analysis	SAS software (version 9.4), Microsoft Excel Office 365 Microsoft (version 1908), JMP (version 14),Partek Genomics Suite 6.6, BD FACSDiva software (v.8.0.1), Prism (v.8.0.1), FlowJo Software Version 10, Illumina's HiSeq 35 2500 or 3000 system,CASAVA (version 1.8.2), TopHat 37 (version2.1.1), nSolver software(version 2.0.134), Bio-Plex Manager software (version 6), VaSera VS-1000 software

For manuscripts utilizing custom algorithms or software that are central to the research but not yet described in published literature, software must be made available to editors/reviewers. We strongly encourage code deposition in a community repository (e.g. GitHub). See the Nature Research guidelines for submitting code & software for further information.

Data

Policy information about availability of data

All manuscripts must include a data availability statement. This statement should provide the following information, where applicable:

- Accession codes, unique identifiers, or web links for publicly available datasets
- A list of figures that have associated raw data
- A description of any restrictions on data availability

RNA-Seq results have been deposited and released in the Gene Expression Omnibus (GEO) database; Geo Series Entry GSE139940 https://www.ncbi.nlm.nih.gov/geo/query/acc.cgi? acc=GSE139940. The source data underlying Figures, Supplementary Figures, Tables and Supplementary Tables are provided as a Source Data file. In addition the source data for fluorescent cell barcoding is provided as a sperate file and can be accessed as referenced(54). All the data is available after executing material transfer and sharing agreement as applicable under laws of US Government. Raw Data is submitted with the manuscript and data availability statement is added to the manuscript.

Field-specific reporting

Please select the one below that is the best fit for your research. If you are not sure, read the appropriate sections before making your selection.

- [x] Life sciences
- [ ] Behavioural & social sciences
- [ ] Ecological, evolutionary & environmental sciences

For a reference copy of the document with all sections, see nature.com/documents/nr-reporting-summary-flat.pdf

Life sciences study design

All studies must disclose on these points even when the disclosure is negative.

Sample size

This was an early phase study focused on safety and mechanistic studies where determination of sample size is not feasible. The sample size chosen was based on what is commonly being used in similar studies and consistent with our experiences in early phase safety studies. No formal samples size calculations were done.
References:
1. Huang J, Su Q, Yang J, Lv Y, He Y, Chen J, Xu L, Wang K, Zheng Q. Sample sizes in dosage investigational clinical trials: a systematic evaluation. Drug Des Devel Ther. 2015;9:305-312
https://doi.org/10.2147/DDDT.S76135
2. Hasni S, Gupta S, Davis M, Poncio E, Temesgen-Oyelakin Y, Joyal E, et al. Safety and Tolerability of Omalizumab: A Randomized Clinical Trial of Humanized Anti-IgE Monoclonal Antibody in Systemic Lupus Erythematosus. Arthritis Rheumatol. 2019;71(7):1135-40.
3. Hartmann S, Biliouris K, Naik H, Rabah D, Stevenson L, Shen C, et al. A clinical population pharmacokinetic/pharmacodynamic model for BIIB059, a monoclonal antibody for the treatment of systemic and cutaneous lupus erythematosus. J Pharmacokinet Pharmacodyn. 2020.

Data exclusions

No data was excluded from analyses.

Replication

Clinical trial data was obtained at the time of each visit , hence could can not be replicated. All the lab experiments were replicated as mentioned in the methods section of the main manuscript and supplementary file.

Randomization

This was a two arm study and subjects were randomized to either tofacitinib or placebo in a 2:1 ratio so that 10 subjects with STAT 4 risk alleles and 10 subjects without STAT 4 risk alleles are in the tofacitinib treatment group. Similarly, 5 subjects with STAT 4 risk alleles and 5 subjects without STAT 4 risk alleles are in the placebo group.

Blinding

Both subjects and investigators were blinded to treatment allocation during the study. Unblinding occurred for the analyses after the last subject has completed Day 84 of the study. All data was generated while the investigators were still blinded to the treatment allocation. To make inferences from the data and understand treatment effects, unblinding was needed to report results.

Reporting for specific materials, systems and methods

We require information from authors about some types of materials, experimental systems and methods used in many studies. Here, indicate whether each material, system or method listed is relevant to your study. If you are not sure if a list item applies to your research, read the appropriate section before selecting a response.

Materials & experimental systems

n/a	Involved in the study	
x		Antibodies
x		Eukaryotic cell lines
x		Palaeontology
x		Animals and other organisms
	x	Human research participants
	x	Clinical data

Methods

n/a	Involved in the study	
x		ChIP-seq
	x	Flow cytometry
x		MRI-based neuroimaging

Human research participants

Policy information about studies involving human research participants

Population characteristics	Details about population characteristics are provided in Table 1 of the manuscript.
Recruitment	Participants were recruited from pre-exisiting SLE Natural History Cohort , self referral, and referral from the local area physicians. All eligible subjected who presented consecutively were invited to participate in research. Possibility for potential self-selection bias or other biases were minimized with the strict adherence to the study protocol inclusion, exclusion criteria, and recruitment procedures.
Ethics oversight	Institutional Review Board of the National Institutes of Health, Bethesda, Maryland, USA.

Note that full information on the approval of the study protocol must also be provided in the manuscript.

Clinical data

Policy information about clinical studies

All manuscripts should comply with the ICMJEguidelines for publication of clinical research and a completedCONSORT checklist must be included with all submissions.

Clinical trial registration	ClinicalTrials.gov NCT02535689
Study protocol	Full Protocol included in file uploaded as Supplemental Material
Data collection	Subject recruitment and data collection was done at the outpatient rheumatology clinics at the Clinical Center of the National Institutes of Health in Bethesda, MD, USA. Recruitment started April 22,2016. First patient screened on July 28,2016. Last patient completed last study visit on December 17th,2018.
Outcomes	The primary outcome of the study was defined as comparing rates of adverse events and rates of SLE disease flares between the tofacitinib group and the placebo group. The SLE disease flare was defined as an increase in SLEDAI 2K score of ≥ 3 or an increase in PGA >1. The secondary outcomes included assessment of clinical response, and effects on quality of life measures. The rates of adverse events (AEs, as defined by National Cancer Institute (NCI), Common Terminology Criteria for Adverse Events (CTCAE), Version 4.0) were recorded weekly. The SLE disease activity was determined using SLEDAI 2K, BILAG 2004, Disease Activity Score 28-Erythrocyte Sedimentation Rate (DAS-28-ESR), Physician Global Assessment (PGA) (Likert scale 0-3), and patient-reported outcomes (SF-36, Multidimensional Assessment of Fatigue questionnaire).

Flow Cytometry

Plots

Confirm that:

- [x] The axis labels state the marker and fluorochrome used (e.g. CD4-FITC).
- [x] The axis scales are clearly visible. Include numbers along axes only for bottom left plot of group (a 'group' is an analysis of identical markers).
- [x] All plots are contour plots with outliers or pseudocolor plots.
- [x] A numerical value for number of cells or percentage (with statistics) is provided.

Methodology

Sample preparation	The following FCB dyes were used: DyLight 350 NHS ester and Pacific Orange NHS ester (Thermo Fisher Scientific, Waltham, MA, USA). Antibodies used for surface staining were: mouse anti-human CD3-PerCP-Cy5.5 (clone SK7), mouse anti-human CD4-PE-Cy7 (clone SK3), mouse anti-human CD8-FITC (clone RPA-T8), and mouse anti-human CD20-APC-H7 (clone H1) (BD Biosciences, San Jose, CA, USA); and CD14-PE (clone M5E2) from BioLegend (San Diego, CA). Antibodies used for phosphoproteins were: pSTAT1(pY701)-Alexa Fluor 647 (clone 4a), pSTAT3(pY705)- Alexa Fluor 647 (clone 4/P-STAT3), and pSTAT5(pY694)- Alexa Fluor 647 (clone 47/Stat5 pY694) (BD Biosciences). Phosflow Lyse/Fix Buffer 5×, Phosflow Perm Buffer III, and Phosflow Barcoding Wash Buffer 4× buffers (BD Biosciences) were prepared and used according to manufacturer's instructions. Phosflow Perm Buffer II (BD Biosciences) was diluted 1:1 with cold PBS and kept on ice before use. For PBMC stimulation, the following cytokines were used: recombinant human IL-10 (PeproTech, Rocky Hill, NJ); human Interferon-α (Cell Signaling Technology, Boston, MA); recombinant human IL-2 (Hoffmann-La Roche Inc., Nutley, NJ). The CTL Anti-Aggregate (CTL) wash buffer was from Cellular Technology Limited (Shaker Heights, OH).<br>Heparinized fresh whole blood was obtained from healthy donors of the NIH Clinical Center Department of Transfusion Medicine.<br>Peripheral blood mononuclear cells (PBMCs) were isolated by Ficoll-Paque gradient centrifugation (GE Healthcare, Chicago, IL) with Leucosep centrifuge tubes (VWR, Radnor, PA), according to manufacturer's instructions. Cells were frozen in medium containing 90% FCS and 10% dymethyl-sulfoxide (DMSO, Sigma-Aldrich, St. Louis, MO, USA) and stored in liquid nitrogen until use.<br>Each dye was dissolved in DMSO at a final concentration of 500 µg/mL and stored at −80 °C. Using the stock solution, FCB dyes were diluted with DMSO to have the following concentrations: 0, 30, and 250 µg/mL for DyLight 350 and 0, 15, and 250 µg/mL for Pacific Orange. A final volume of 40 µL/well was used for barcoding by combining 30 µL of sample and 5 µL of each dye or

DMSO in order to have the final concentrations of 0, 6.25 (for DyLight 350) or 3.125 (for Pacific Orange), and 31.25 μg/mL. At the end of stimulation, untreated and treated PBMCs were fixed with 550 μL of 1× Lyse/Fix Buffer for 10 min at 37 °C, centrifuged at 500 g for 5 min, and then permeabilized with 600 μL of Phosflow Perm Buffer III on ice for 30 min. Subsequently, cells were washed twice with 600 μL of Phosflow Perm Buffer II (1:1 with PBS), resuspended in 35 μL of cold Phosflow Perm Buffer II and 30 μL were added to appropriate wells (1.29 × 106 cells/ 30 μL/ well) with various concentrations of FCB dyes previously prepared in a U-bottom 96 well plate. After incubation on ice for 20 min in the dark, samples within each matrix were combined and washed twice with BD Phosflow Barcoding Wash Buffer (3 mL) by centrifugation at 500 g for 5 min, followed by resuspension with BD Phosflow Barcoding Wash Buffer (50 μL) for antibody staining. Cells were stained with 20 μL of CD14-PE (20 μg/mL), 40 μL of CD8-FITC (1.25 μg/mL), 40 μL of CD3-PerCP-Cy5.5 (0.6 μg/mL), 40 μL of CD20-APC-H7 (2.4 μg/mL), 10 μL of CD4-PE-Cy7 (0.3 μg/mL), and 40 μL of appropriate pSTAT-Alexa Fluor 647 (0.3 μg/mL for pSTAT1 and pSTAT3; 0.6 μg/mL for pSTAT5) according to manufacturer's instructions. Cells were incubated for 1 h at room temperature in the dark with gentle shaking, washed with BD Phosflow Barcoding Wash Buffer (3 mL), and resuspended in 300 μL of the same buffer for acquisition. As non-barcoded controls, 1.5 × 106 cells were directly stained without fixation, permeabilization and barcoding. This control was prepared in order to exclude that methanol used for membrane permeabilization might disrupt epitopes and influence antibody fluorescence (Behbehani et al., 2014). For each treated and untreated donor, 1.5 × 106 fixed and permeabilized cells were stained without barcoding. After staining, all samples were washed and acquired using BD Phosflow Barcoding Wash Buffer.

Instrument

LSR Fortessa (BD Biosciences) equipped with 6 lasers (ultraviolet, 355nm; violet, 405nm; blue, 488nm; green, 552nm; orange, 592nm; and red laser, 628nm) and BD FACSDiva software (v.8.0.1, BD Biosciences) were used for sample acquisition

Software

FlowJo software v.10.0.7b

Cell population abundance

We did not assess cell numbers in our analysis but only the pSTATs in the different cell types. Our study was not aimed at assessing cell abundance but rather to the intracellular signaling events.

Gating strategy

After post-acquisition compensation using FlowJo, lymphocytes and monocytes were identified using linear parameters (FSC-A vs SSC-A) and double cells were excluded (FSC-A vs FSC-H) (Supplementary Fig. 1). Single cells were deconvoluted by plotting the FCB dye channels (Pacific Orange vs DyLight 350), identifying nine populations. On each barcoded lymphocyte sample, CD3 and CD20 expression were first investigated, and CD4 and CD8 expression was further studied on CD3+ cells. CD14+ cells were identified (CD14 vs FSC-A) on barcoded monocyte populations. pSTAT1, pSTAT3, or pSTAT5 expression was calculated on CD3+, CD4+, CD8+, CD20+, and CD14+ cells as median fluorescence intensity (MFI) values.

☒ Tick this box to confirm that a figure exemplifying the gating strategy is provided in the Supplementary Information.
